# Computational approaches for discovery of common immunomodulators in fungal infections: towards broad-spectrum immunotherapeutic interventions

**DOI:** 10.1186/1471-2180-13-224

**Published:** 2013-10-07

**Authors:** Yared H Kidane, Christopher Lawrence, T M Murali

**Affiliations:** 1 Genetics, Bioinformatics, and Computational Biology PhD Program, Virginia Tech, Blacksburg, VA 24061, USA; 2 Virginia Bioinformatics Institute, Virginia Tech, Blacksburg, VA 24061, USA; 3 Department of Biology, Virginia Tech, Blacksburg, VA 24061, USA; 4 Department of Computer Science, Virginia Tech, Blacksburg, VA 24061, USA; 5 ICTAS Center for Systems Biology of Engineered Tissues, Virginia Tech, Blacksburg, VA 24061, USA; 6, Universities Space Research Association, Houston, TX 77058, USA

**Keywords:** Host-oriented therapy, Broad-spectrum target, Immunomodulation, Drug-resistance, Drug-target discovery, Immunotherapy

## Abstract

**Background:**

Fungi are the second most abundant type of human pathogens. Invasive fungal pathogens are leading causes of life-threatening infections in clinical settings. Toxicity to the host and drug-resistance are two major deleterious issues associated with existing antifungal agents. Increasing a host’s tolerance and/or immunity to fungal pathogens has potential to alleviate these problems. A host’s tolerance may be improved by modulating the immune system such that it responds more rapidly and robustly in all facets, ranging from the recognition of pathogens to their clearance from the host. An understanding of biological processes and genes that are perturbed during attempted fungal exposure, colonization, and/or invasion will help guide the identification of endogenous immunomodulators and/or small molecules that activate host-immune responses such as specialized adjuvants.

**Results:**

In this study, we present computational techniques and approaches using publicly available transcriptional data sets, to predict immunomodulators that may act against multiple fungal pathogens. Our study analyzed data sets derived from host cells exposed to five fungal pathogens, namely, *Alternaria alternata*, *Aspergillus fumigatus*, *Candida albicans*, *Pneumocystis jirovecii*, and *Stachybotrys chartarum*. We observed statistically significant associations between host responses to *A. fumigatus* and *C. albicans*. Our analysis identified biological processes that were consistently perturbed by these two pathogens. These processes contained both immune response-inducing genes such as MALT1, SERPINE1, ICAM1, and IL8, and immune response-repressing genes such as DUSP8, DUSP6, and SPRED2. We hypothesize that these genes belong to a pool of common immunomodulators that can potentially be activated or suppressed (agonized or antagonized) in order to render the host more tolerant to infections caused by *A. fumigatus* and *C. albicans*.

**Conclusions:**

Our computational approaches and methodologies described here can now be applied to newly generated or expanded data sets for further elucidation of additional drug targets. Moreover, identified immunomodulators may be used to generate experimentally testable hypotheses that could help in the discovery of broad-spectrum immunotherapeutic interventions. All of our results are available at the following supplementary website: http://bioinformatics.cs.vt.edu/~murali/supplements/2013-kidane-bmc

## Background

Fungi are the second most abundant pathogens, accounting for 307 of the 1,407 species of recognized human infectious pathogens [1]. Health-related impacts of fungal pathogens have increased over the past several years. For instance, in the years from 2000 to 2005, the fraction of people who were admitted to hospitals primarily due to the presence of *Candida* species in their blood increased by 52% [2]. Furthermore, mortality rates from invasive fungal infections has increased by 320% from 1980 through 1997 in the United States alone [3]. An increase in immunocompromised individuals, prevalence of cancer, chemotherapy treatments, organ transplantation, and autoimmune diseases are major factors that have contributed to the rise in opportunistic fungal infections [4].

Despite the medical importance of fungal pathogens, the discovery and development of new antifungal agents has been very slow. There are only four major classes of antifungals for the treatment of systemic infections, namely, fluoropyrimidine analogs, polyenes, azoles, and echinocandins [5]. Fungal and human cells have similar cellular structure and molecular machinery. Consequently, the number of fungal-specific targets are few, partly contributing to the shortage of antifungal drugs [6]. Even for approved drugs, e.g., polyenes, toxicity to the host has been a major problem [7]. Over the past two decades, drug resistant fungal pathogens have emerged, resistance of *Candida* species to azoles being the most common type [8]. Recent examples of drug-resistant fungal pathogens include multidrug resistant *C. glabrata* and azole-resistant central nervous system infections by *A. fumigatus*[9]

There are two potential immunomodulatory mechanisms for the treatment of fungal infections: (1) decreasing pathogen load by enhancing clearance of the pathogen from the host and (2) increasing the ability of the host to limit the health impacts of pathogens by making the host more tolerant to infections [10]. The host’s defense capacity primarily constitutes the sum of these two mechanisms. Therapeutic strategies that merely focus on directly killing or eradicating pathogens from host cells are prone to the development of drug resistance. On the contrary, strategies that target host tolerance were shown to be viable approaches to circumvent the problem of drug resistance [11].

A host’s tolerance to pathogens can be improved by modulating the immune system so that it responds rapidly and robustly in all facets, ranging from the recognition of pathogens to their clearance from the host. For example, this strategy may involve the use of granulocyte colony stimulating factor (G-CSF) to increase the activation of immune cells such as neutrophils, macrophages, and dendritic cells or the activation of toll-like receptors to promote the recognition of pathogens. Strategies may also involve the use of vaccines to increase the host’s humoral immunity [12], which is an antibody-mediated immunity, but this approach may have limitations in the context of immunosuppression. For instance, mice that are deficient in pentraxin 3, a gene involved in pathogen recognition, have been shown to be more susceptible to *A. fumigatus* infection [13]. In another study, the administration of pentraxin 3 improved survival rate of immunocompromised rats infected with *A. fumigatus* and decreased the overall fungal burden [14]. Another example is the use of thymosin *α*1 (T *α*1), a naturally occurring thymic peptide that was shown to facilitate the induction of interleukin 12 (IL-12) and the functional maturation of dendritic cells [15]. Following this study, chemically synthesized molecules have become available e.g., Thymalfasin which mimics the human Thymosin *α*1. The benefit of immunomodulatory agents may be maximized by designing them so that they can be effective against multiple pathogens. For instance, activation of antifungal *CD*4^+^*T*_*h*_1 immunity using epitope *p41* of *A. fumigatus* extracellular cell wall glucanase *Crf1* succeeded in improving outcomes in both invasive aspergillosis and candidiasis [16].

An understanding of biological processes and genes that are perturbed during fungal exposure, colonization, and/or invasion will help guide identification and development of therapies that are targeted to enhance the hosts’ tolerance against fungal pathogens. In this study, we present computational techniques to predict immunomodulators that can act against multiple fungal pathogens, based on publicly available transcriptional data sets.

## Results and discussion

We obtained genome-wide transcriptional data sets of host responses upon exposure to fungal pathogens from the NCBI’s Gene Expression Omnibus (GEO) [17] and ArrayExpress [18]. We filtered data using the criterion described in Section Methods, after which we retained nine data sets. These data sets involved five fungal pathogens, namely, *Alternaria alternata*, *Aspergillus fumigatus*, *Candida albicans*, *Pneumocystis jirovecii*, and *Stachybotrys chartarum*[19-24]. They covered seven target cell/tissue types including macrophages, epithelial cells, dendritic cells, monocytes, neutrophils, endothelial cells and lung cells, totaling 107 samples (Table 1).

**Table 1:** Description of gene expression data sets used in the study

**ID**	**Author(s)**	**Acc #**	**Pathogen**	**Platform**	**Target cell/tissue**
	***H. sapiens***				
D1	Babiceanu *et al.* [*]	GSE32893	*A. alternata*	HG-U133_Plus_2	Epithelial
D2	Mezger *et al.*[19]	GSE6965	*A. fumigatus*	HG-U133_Plus_2	Dendritic
D3	Sharon *et al.*[21]	GSE24983	*A. fumigatus*	HG-U133A_2	Epithelial
D4	Mattingsdal *et al.* [*]	E-MEXP-1103	*A. fumigatus*	HG-U133_Plus_2	Monocytes
D5	Rubin-Bejerano *et al.* [*]	E-MEXP-914	*C. albicans*	HG-U133A_2	Neutrophils
D6	Rizzetto *et al.*[22]	E-MTAB-135	*C. albicans*	Illumina HumanHT-12 v3.0	Dendritic
D7	Müller *et al.*[23]	GSE7355	*C. albicans*	HG-U133A	Endothelial
	***R. norvegicus***				
D8	Cheng *et al.*[24]	GSE20149	*P. jirovecii*	RG_U34A	Macrophages
	***M. musculus***				
D9	Shimodaira *et al.* [*]	GSE23178	*S. chartarum*	Mouse430_2	Lung

The columns from left to right are (i) data set id, (ii) authors and the publication associated with the data set, (iii) GEO or ArrayExpress accession number, (iv) pathogen name, (v) microarray platform, and (vi) cell/tissue type from which gene expression measurements were taken. Data sets are categorized by the host, namely, *H. sapiens*, *R. norvegicus* and *M. musculus.* [*] indicates the data set that does not have an associated publication.

We computed biclusters using the procedure described in Section Methods. Briefly, first, we computed gene sets up- and down- regulated by each fungal pathogen using Gene Set Enrichment Analysis (GSEA) [25]. Then, we created two binary matrices for up- and down- regulated gene sets, respectively. These matrices captured if a gene set was perturbed or unperturbed by a pathogen to a statistically significant extent. We biclustered these matrices using Bimax biclustering software [26] in order to identify subsets of fungal pathogens that commonly up- or down- regulated subsets of gene sets. We obtained 27 up-regulated and 13 down-regulated biclusters (see http://www.biomedcentral.com/content/supplementary/1471-2180-13-224-S1.zipAdditional file 1: Table S1 and Table S2 on supplementary website for details on these biclusters). We assessed the statistical significance of the biclusters by comparing their sizes to biclusters found in randomized data sets. After multiple hypothesis correction using the method of Benjamini and Hochberg [27], we retained three significant biclusters (two up-regulated and one down-regulated) at a 0.05 *p*-value cutoff (Table 2). All the significant biclusters contained two pathogens, namely, *A. fumigatus* and *C. albicans*. Among the two significantly up-regulated biclusters, we noticed that there were more up-regulated gene sets in bicluster B1 than in bicluster B2. We reasoned that this difference arose from the variation in the target cell type used in *A. fumigatus* infection, since the *C. albicans* data sets in the two biclusters were identical. The *A. fumigatus* data in bicluster B1 was derived from lung epithelial cells whereas the one in bicluster B2 was derived from dendritic cells (Table 1). Epithelial cells of the lung are the primary entry points for *A. fumigatus* infection. *A. fumigatus* has been shown to adhere to and enter epithelial cells of the lung in order to escape the hosts’ resident phagocytic cells [28]. We reasoned this fact may explain why *A. fumigatus* perturbed more genes in epithelial cells as compared to in dendritic cells. Hence, in this paper we decided to focus our discussion on up-regulated bicluster B1 and down-regulated bicluster B3.

**Table 2:** Statistically significant biclusters

**ID**	**Pathogens**	**Bicluster** ***p*-value**	**Number of** **gene sets**
	**Up-regulated**		
B1	*A. fumigatus* (D3) and *C. albicans* (D6)	<10^−5^	204
B2	*A. fumigatus* (D2) and *C. albicans* (D6)	<10^−5^	174
	**Down-regulated**		
B3	*A. fumigatus* (D3) and *C. albicans* (D6)	<10^−5^	133

This table shows the bicluster that are statistically significant at a 0.05 *p*-value cutoff. The columns from left to right are: (i) bicluster identification code, (ii) list of pathogens contained in the biclusters. The text in parentheses indicate the gene expression data identification code (see Table 1), (iii) a *p*-value indicating the statistical significance of the bicluster, (iv) the number of gene sets in the bicluster. The table shows up- and down- regulated biclusters.

Pathogens may commonly perturb a gene set without perturbing a single gene in common. Therefore, we started our analysis by detecting if gene sets perturbed by *A. fumigatus* and *C. albicans* share common genes. To this end, we considered the leading edge genes of each gene set perturbed by each pathogen. As computed by GSEA, the leading edge genes for a gene set-pathogen pair constitute those genes that contribute the most to perturbation of a gene set by a pathogen [25]. We computed the intersection of leading edge genes for the two pathogens for each gene set in biclusters B1 and B3. We retained 115 gene sets in up-regulated bicluster B1 and 49 gene sets in down-regulated bicluster B3 whose leading edge in the two pathogens have a non-empty intersection.

Next, we ranked the remaining gene sets in increasing order of McNemar’s Chi-squared statistic. We used this statistic to measure whether or not the two pathogens perturbed different numbers of genes in each gene set. We focused on gene sets where both pathogens perturbed the same number of genes; i.e with small value of this statistic. We called such gene sets *consistently perturbed gene sets* (see Methods).

We hypothesize that such consistently perturbed gene sets represent common host responses against the pathogens *A. fumigatus* and *C. albicans*, and that they may contain genes with the potential to serve as immunomodulators. We selected 19 gene sets from bicluster B1 and three gene sets from bicluster B3 that have a McNemar’s test statistic value of zero (Table 3). Then, we re-ranked these gene sets in increasing order of the number of genes commonly perturbed by *A. fumigatus* and *C. albicans*. Among these gene sets, we selected the top-ten gene set from up-regulated bicluster B1 and all three gene sets from down-regulated bicluster B3 (a total of 13 gene sets) for discussion in this paper (Table 4).

**Table 3:** Consistently perturbed gene sets

**Gene set**	**| *A *∩ *C *|**	**| *A *∖ *C *|**	**| *C *∖ *A *|**	**|*A***^***′***^**∩*C***^***′***^**|**	**| *n *|**
**Up-regulated**					
Adaptive immune response (MsigDB)	1	4	4	15	24
Dissolution of fibrin clot (NCI)	2	1	2	3	8
Granulocytes pathway (BIOCARTA)	3	0	1	10	14
Inactivation of MAPK activity (MsigDB)	3	1	1	9	14
Viral genome replication (MsigDB)	3	2	2	14	21
Hedgehog pathway up (NETPATH)	3	2	1	16	22
Positive regulation of immune response (MsigDB)	3	3	2	21	29
CD28 dependent PI3K AKT signaling (REACTOME)	4	2	3	10	19
Peptidyl tyrosine phosphorylation (MsigDB)	4	5	6	12	27
CDMAC pathway (BIOCARTA)	5	2	3	6	16
CARDIACEGF pathway (BIOCARTA)	5	3	3	7	18
CHUK NFKB2 REL IKBKG SPAG9 NFKB1 NFKBIE COPB2 TNIP1 NFKBIA RELA TNIP2 complex (CORUM)	6	1	2	3	12
CD40 pathway (BIOCARTA)	7	1	1	6	15
EGFR1 pathway down (NETPATH)	8	13	13	64	98
NFKAPPABcanonicalpathway (NCI)	9	2	3	10	24
TRAF6 mediated induction of the antiviral cytokine IFN alpha beta cascade (REACTOME)	10	7	6	30	53
Negative regulation of apoptosis (MsigDB)	19	18	19	91	147
MAPK signaling pathway (KEGG)	24	34	34	175	267
Signaling in immune system (REACTOME)	28	47	47	244	366
**Down-regulated**					
LSM1-7 complex (CORUM)	4	2	1	0	7
Respiratory chain complex I (incomplete intermediate) mitochondrial (CORUM)	4	3	4	0	11
Mitochondrial respiratory chain (MsigDB)	8	7	7	2	24

The columns from left to right are: (i) gene set name, (ii) |*A****∩****C*|: the number of genes in the gene set perturbed by both *A. fumigatus* and *C. albicans*, (iii) |*A****∖****C*|: the number of genes perturbed by *A. fumigatus* but not by *C. albicans*, (iv) |*C****∖****A*|: the number of genes perturbed by *C. albicans* but not by *A. fumigatus*, (v) |*A*^′^***∩****C*^′^|: the number of genes unperturbed by both pathogens, and (vi) |*n*|: the total number of genes in a gene set. For each gene set, we have indicated the source database in parentheses. This table shows gene sets that have a McNemar’s test statistic value of zero.

**Table 4:** Top-ten consistently perturbed gene sets

**Gene set**	**Genes**
**Up-regulated**	
Adaptive immune response (MsigDB)	MALT1
Dissolution of fibrin clot (NCI)	SERPINE1, PLAUR
Granulocytes pathway (BIOCARTA)	ICAM1, IL8, IL1A
Inactivation of MAPK activity (MsigDB)	DUSP8, DUSP6, SPRED2
Viral genome replication (MsigDB)	TNIP1, CCL2, IL8
Hedgehog pathway up (NETPATH)	PMP22, THBD, MYC
Positive regulation of immune response (MsigDB)	FYN, EREG, MALT1
CD28 dependent PI3K AKT signaling (REACTOME)	MAP3K14, FYN, TRIB3, MAP3K8
Peptidyl tyrosine phosphorylation (MsigDB)	CLCF1, STAT1, IL12A, LYN
CDMAC pathway (BIOCARTA)	NFKB1, NFKBIA, FOS, MYC, RELA
**Down-regulated**	
LSM1-7 complex (CORUM)	LSM4, LSM6, LSM2, LSM7
Respiratory chain complex I incomplete intermediate mitochondrial (CORUM)	NDUFS6, NDUFV2, NDUFS4, NDUFS7
Mitochondrial respiratory chain (MsigDB)	UQCRC1, NDUFAB1, BCS1L, NDUFS4, NDUFS7, NDUFS3, NDUFS8, SURF1

Top-ten consistently perturbed gene sets, ranked by the number of genes they contain for up- and down- regulated gene sets.

We organize our results into two sections: 

1. We evaluated the putative immunomodulatory activity of the 13 selected gene sets and the genes they contain, based on evidence found in the literature.

2. We grouped gene sets/genes depending on whether they induced or repressed immune response, based on the observation made in Step 1. Immune response-inducing gene sets/genes represent up- or down- regulated gene sets/genes that would make the host more tolerant to infections by *A. fumigatus* and *C. albicans*, while immune response-repressing gene sets/genes are those that would have an opposite effect when up- or down- regulated. We generated a network of these gene sets/genes and analyzed their combined immunomodulatory roles.

### Predicted immunomodulatory activity

Most immunocompetent humans are immune to infections caused by *A. fumigatus* and *C. albicans*. The innate and adaptive immune systems of the host are versatile enough to prevent infection by these microorganisms. The host becomes prone to infection only when one or more of the immune system components such as physical barriers, cellular or humoral components are compromised [29,30]. Consistently and commonly perturbed gene sets represent biological processes that are important for pathogen survival or for defending the host from being invaded by a pathogen. In other words, these gene sets are important for maintaining the balance between pathogen clearance, colonization and invasion. Identification of these host responses is the first step in the search for immunotherapies that are effective against both *A. fumigatus* and *C. albicans*. Augmenting these host responses may assist an immunocompromised individual in the fight against these pathogens. Guided by these hypotheses, we examined the 13 highly consistently and commonly perturbed gene sets identified earlier (Table 4). These gene sets contain a total of 41 unique genes. Below we discuss the potential immunomodulatory activity of these genes in relation to the gene sets to which they belong, based on evidence found in the literature.

#### Adaptive immune response

In the “Adaptive immune response” (GO:0002460) gene set, *A. fumigatus* and *C. albicans* commonly up-regulated the mucosa-associated lymphoid tissue lymphoma translocation gene 1 (MALT1) (Table 4). MALT1 is involved in the activation of Th-17 based adaptive anti-fungal immunity. This process involves the following steps: Zymosan, a component of the fungal cell wall, activates dectin-1, a pattern recognition receptor in the host. This interaction induces a protein scaffold consisting of caspase recruitment domain 9 (Card9), B cell lymphoma 10 (Bcl10) and Malt1 [31]. Specifically, the activation of Malt1 is responsible for the activation of the c-Rel component of NF- *κ*B, which then induces Th-17 polarizing cytokines such as IL-1 *β* and IL-23 [32].

**Figure 1:**
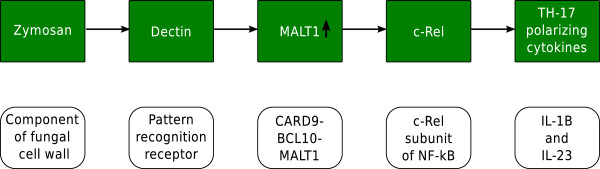
**Immunomodulation of Th-17 adaptive immunity.** Immunomodulation of Th-17 adaptive immunity using MALT1. The figure shows the cascade of events in the malt1 dependent activation of Th-17 type adaptive immunity.

Inhibition of MALT1 has been shown to prevent c-Rel activation and Th-17 immunity in human primary dendritic cells infected with *Candida* species [33]. Th-17 cells secrete IL-17, which is important for mobilizing neutrophils against fungal infections [32]. Gringhuis *et al.*[32] have experimentally verified the importance of Malt1 in the induction of adaptive immunity against various species of *Candida*. In addition, they pointed out that a similar mechanism may have the same effect in *A. fumigatus* infection, due to the presence of glucans in both the cell wall of *C. albicans* and *A. fumigatus*. Our analysis supports their premise that up-regulation of MALT1 might be a common host response mechanism against *A. fumigatus* and *C. albicans*. Hence, we hypothesize that increasing the expression of MALT1 might help to promote pathogen recognition by dectin-1 and may serve as a viable strategy for immune response modulation (Figure 1).

#### Dissolution of fibrin clot

Dissolution of fibrin clot (also known as fibrinolysis) refers to the degradation of fibrin. The main enzyme in this process is plasmin. Plasmin is obtained when the precursor plasminogen is converted to plasmin by plasminogen activators. However, the conversion of plasminogen to plasmin can be inhibited by plasminogen-activator inhibitors [34].

Previous studies have linked the activation of plasmin and hence fibrinolysis to increased pathogenicity of both *A. fumigatus* and *C. albicans*. The binding of *Candida* to the host’s plasmin via its surface cell receptors resulted in an increased ability to cross an *in-vitro* blood-brain barrier [35]. Urokinase plasminogen activator (uPA) and urokinase plasminogen activator receptor (uPAR), agents that convert plasminogen to plasmin, were up-regulated when human monocytes were co-cultured with *A. fumigatus*[36].

**Figure 2:**
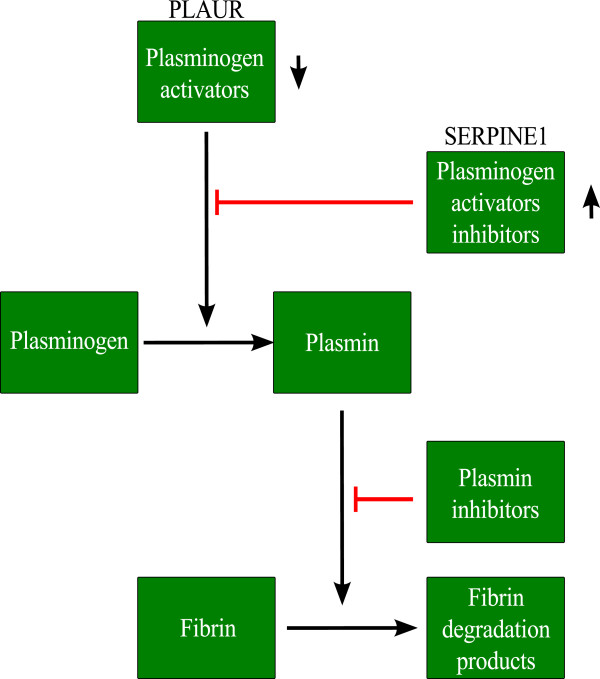
**Immunomodulation of the dissolution of fibrin clot.** Immunomodulation of the dissolution of fibrin clot by using SERPINE1 and PLAUR genes. The figure shows a simplified model of the dissolution of fibrin clot and the involvement of the genes SERPINE1 and PLAUR in this process.

In our study, *A. fumigatus* and *C. albicans* up-regulated the genes serpin peptidase inhibitor clade E member 1 (SERPINE1) and plasminogen activator urokinase receptor (PLAUR) in the “Dissolution of fibrin clot” (NCI-PID) gene set (Table 4). PLAUR encodes for the receptor of uPA and SERPINE1 is an inhibitor of tissue plasminogen activator (tPA) and uPA. The genes SERPINE1 and PLAUR counteract each other, in that PLAUR tends to increase the level of plasmin while SERPINE1 tends to inhibit plasmin formation. The up-regulation of these two genes in our analysis might indicate competition between the host and the pathogen where *A. fumigatus* and *C. albicans* attempted to create a favorable environment for their pathogenicity, while the host reacts to overcome this process. This observation led us to speculate that up-regulating SERPINE1 and/or down-regulating PLAUR may help an immunocompromised individual to become more tolerant to infections caused by either of these pathogens (Figure 2).

#### Granulocytes pathway

*A. fumigatus* and *C. albicans* up-regulated three genes, namely, intercellular adhesion molecule 1 (ICAM1), interleukin 8 (IL-8) and interleukin 1 alpha (IL-1 *α*) in the “Granulocytes pathway” (BIOCARTA) gene set (Table 4). Adhesion molecules play an important role in the host defense mechanism against pathogens. Oral epithelial cells have been shown to induce ICAM1 in order to recruit and maintain neutrophils during infection by *Actinobacillus actinomycetemcomitans* and *Porphyromonas gingivalis*[37]. Inhibition of ICAM1 has been shown to hinder adherence of *C. albicans* to human gingival epithelial cells (HGECs) and resulted in decreased secretion of IL-8, an important pro-inflammatory molecule secreted during *A. fumigatus* and *C. albicans* infections [38-40]. Another study linked the up-regulation of IL-1 *α* with the secretion of IL-8 in oral epithelial cells infected with *C. albicans*[41]. All these studies indicated the common role of ICAM1, IL-8 and IL-1 *α* in the host defense against pathogens. Our analysis further supports published studies showing that the up-regulation of the granulocytes pathway, in particular of these three genes, may be an important aspect of the host’s defense against infections caused by *A. fumigatus* and *C. albicans*. We suggest that ICAM1, IL-8 and IL-1 *α* as candidates for a host-oriented therapy that exploits the importance of granulocytes pathway.

#### Inactivation of mapk activity

Mitogen-activated protein kinases (MAPKs) encompass a group of protein kinases that regulate a number of cellular processes ranging from cellular differentiation and proliferation to apoptosis. Extracellular signal-regulated kinases (ERKs) such as ERK1, ERK2, and ERK3 were among the first recognized MAPKs in mammals [42]. Inactivation of MAPKs has a negative effect on the normal functioning of host systems including immune responses. For instance, Dubourdeau *et al.* indicated that inactivation of MAPK/ERK correlates with a decrease in the activation of innate immunity against *A. fumigatus* in a mouse model [43].

In our analysis, *A. fumigatus* and *C. albicans* commonly up-regulated three members of the “Inactivation of mapk activity” (GO:0000188) gene set, namely, dual specificity phosphatase 6 (DUSP 6), dual specificity phosphatase 8 (DUSP8), and sprouty-related EVH1 domain-containing 2 (SPRED2) genes (Table 4). DUSP6 and DUSP8 are known negative regulators of MAPK activity [44]. Another study indicated that SPRED2 negatively regulates growth factor-mediated ERK signaling and hematopoiesis [45]. Given these observations, we hypothesize that the up-regulation of the “Inactivation of mapk activity” gene set might make the host more vulnerable to infections caused by *A. fumigatus* and *C. albicans*, and that the genes DUSP6, DUSP8 and SPRED2 might play a vital role in this aspect. Hence, modulating the expression or activities (phosphorylation status) of these gene products or downstream targets may work to maintain cellular signaling via MAPKs.

#### Viral genome replication

The “Viral genome replication” (GO:0019079) gene set annotates 21 genes. Among these genes, *A. fumigatus* and *C. albicans* commonly up-regulated the genes monocyte chemotactic protein-1 (MCP-1), TNFAIP3 interacting protein 1 (TNIP1), and interleukin 8 (IL-8) (Table 4). We have discussed the positive role of up-regulation of IL-8 in the host defense against *A. fumigatus* and *C. albicans* under the “Granulocytes pathway” gene set. Likewise, activation of MCP-1 (CCL2) has been shown to have a positive role in the host defense against aspergillosis and candidiasis. Neutralizing the MCP-1 gene resulted in increased mortality and pathogen burden in the lungs of mice with invasive aspergillosis [46]. MCP-1 was also produced by oral and vaginal epithelial cells when challenged with *C. albicans*[47]. TNIP1, through its interaction with tumor necrosis factor *α*-induced protein 3 (TNFAIP), is able to inhibit NF- *κ*B [48,49]. The inhibition of NF- *κ*B is reported to decrease the number of neutrophils as well as the phagocytic and microbicide capacity against *C. albicans*[50]. Hence, in this gene set, while the up-regulation of IL-8 and MCP-1 might help the host to better tolerate the damage caused by *A. fumigatus* and *C. albicans*, down-regulation of TNIP1 might be advantageous to the host.

#### Hedgehog pathway up

The hedgehog (Hh) signaling pathway (NETPATH) regulates the expression of genes that are important for various cellular processes including growth, cell cycle regulation and embryogenesis [51]. In this pathway, *A. fumigatus* and *C. albicans* up-regulated the genes peripheral myelin protein 22 (PMP22), thrombomodulin (THBD), and MYC (Table 4). We did not find evidence in the literature that linked the Hh signaling pathway to fungal infection. However, a recent study showed that up-regulation of this biological pathway increased cellular permissiveness in hepatitis C infection [52]. Paya *et al.*[53] indicated that fungal infections such as those caused by *Candida* spp. and hepatitis infections reoccur in liver transplant patients, which may suggest an indirect association between the up-regulation of the Hh signaling pathway and the pathogenesis of *C. albicans*. Guided by this observation, we propose that the up-regulation of this pathway might result in increasing the pathogenesis of opportunistic infections such as those caused by *A. fumigatus* and *C. albicans*. Thus, down-regulation of this pathway may be a plausible therapeutic approach to test.

#### Positive regulation of immune response

In the “Positive regulation of immune response” (GO:0050778) gene set, *A. fumigatus* and *C. albicans* commonly up-regulated the genes tyrosine-protein kinase FYN, epiregulin (EREG) and mucosa associated lymphoid tissue lymphoma translocation gene 1 (MALT1) (Table 4). We have discussed the role of MALT1 in the activation of Th-17 host immunity (see the section on the “Adaptive immune response” gene set). Experimental evidence has suggested that the up-regulation of FYN and EREG is associated with an increase in the host’s defense against *A. fumigatus* and *C. albicans*. FYN is involved in the control of Th1-Th2 type cellular differentiation. Kudlacz *et al.*[54] showed that FYN-knockout mice exhibited an increase in Th2-type immune response and a decrease in allergic airway inflammation. A shift in the host’s immune response towards Th2-type will generally aggravate invasive aspergillosis as well as invasive candidiasis [16]. This observation suggests that the up-regulation of FYN might be linked to the maintenance of the host’s Th1 type immunity. Th1 type immune response is an integral component of host response in the protection against both *A. fumigatus* and *C. albicans*[55,56]. In addition EREG, which encodes for epiregulin (a protein involved in the formation of epidermal growth factor receptor agonist), plays a vital role in the proliferation of immune cells such as macrophages [57]. EREG is up-regulated when alveolar macrophages are challenged with *A. fumigatus*[58]. The up-regulation of FYN and EREG by *A. fumigatus* and *C. albicans* in our analysis, might indicate the hosts attempt to shift towards a Th1 type immune response and an increase in the proliferation of phagocytic cells.

#### Cd28 dependent Pi3K Akt signaling

Phosphatidylinositol 3-kinase (PI3K) signaling is among the first signal transduction events that occurs when an antigen interacts with host cell surface receptors. Activated PI3K will recruit pleckstrin homology domain-containing proteins such as Akt to initiate the PI3K/Akt signaling cascade. PI3K/Akt signaling plays an important role in various cellular activities ranging from cellular differentiation to motility [59].

The “Cd28 dependent Pi3K Akt signaling” (REACTOME) gene set contains 19 annotated genes. Among these, the genes mitogen-activated protein 3 kinase 8 (MAP3K8), mitogen-activated protein 3 kinase 14 (MAP3K14), tyrosine-protein kinases FYN and tribbles homolog 3 (Drosophila) (TRIB3) were commonly up-regulated by *A. fumigatus* and *C. albicans* (Table 4). We have discussed the role of FYN in the regulation of Th1-Th2 type immune response earlier (see section on “Positive regulation of immune response” gene set).

MAP3K8 is known to activate extracellular signal-regulated kinases (ERKs). MAP3K8-deficient mice exhibited low levels of TNF- *α* and ERK production [60]. Similarly, MAP3K14 also participates in ERK signaling [61]. The activation of MAPK/ERK is important in the recognition of *A. fumigatus* by innate immunity [43]. The fourth gene perturbed in this gene set is TRIB3. Schwarzer *et al.*[62] have shown that TRIB3 is up-regulated when there is a shortage of nutrient supplies in cells. With this observation in mind, we reasoned that the the up-regulation of the “Cd28 dependent Pi3K Akt signaling” gene set is part of the host defense mechanism against the two pathogens. An immunomodulation strategy might focus on the four genes discussed above.

#### Peptidyl tyrosine phosphorylation

The “Peptidyl tyrosine phosphorylation” (GO:0018108) gene set annotates 27 genes. Among these genes, *A. fumigatus* and *C. albicans* commonly up-regulated the genes signal transducer and activator of transcription 1 (STAT1), cardiotrophin-like cytokine factor 1 (CLCF1), interleukin 12A (IL12A), and v-yes-1 Yamaguchi sarcoma viral related oncogene homolog (LYN). STAT1 plays a vital role in the induction of Th17 and Th1 type host immune responses. It encodes for an adapter molecule that is important for the activation of the IL-23 (IL23R) and IL-12 (IL12R) receptor pathways. IL23R and IL12R are vital in inducing Th-17 and Th-1 type immune responses. Previous studies indicated that mutation in STAT1 resulted in defective Th-17-type and Th-1-type responses [63,64]. Hence, this gene set may have a positive role in the host defense mechanism.

#### Cdmac pathway

The cadmium-induced DNA synthesis and proliferation in macrophages (CDMAC) pathway (BIOCARTA) contains 16 annotated genes that were dysregulated when macrophages were exposed to cadmium ions (*Cd*2^+^). Among these genes, *A. fumigatus* and *C. albicans* commonly up-regulated five genes namely, NFKB1, RELA, NFKBIA, FOS, and MYC. Cadmium induces both cellular proliferation promoting and immune response inhibiting genes. The cellular proliferation genes include NFKB1, REL1, FOS, and MYC. NFKB1 and REL1 are involved in the formation of NF- *κ*B complexes and NFKBIA is an inhibitor of NF- *κ*-B/REL complexes [65]. Taken together, the up-regulation of this gene set supports the common observation regarding the proliferation of macrophages in response to attempted infection.

#### Lsm1-7 complex

The “Lsm1-7 complex” (CORUM) gene set annotates seven genes that are involved in mRNA degradation [66]. Among these genes *A. fumigatus* and *C. albicans* commonly down-regulated four genes, namely, LSM4, LSM6, LSM2, and LSM7 (Table 4). Unstable mRNA degradation has been linked to a number of diseases including the inflammatory disease, arthritis [67]. Previous studies also indicated the potential regulation of mRNA stability in the treatment of a wide range of diseases including those caused by infectious pathogens [68,69]. The down-regulation of genes related to mRNA degradation in our analysis might be linked to the pathogenesis of *A. fumigatus* and *C. albicans*. The genes LSM4, LSM6, LSM2, and LSM7 are potential candidates for an immunological strategy that targets the LSM1-7 complex in countering these two pathogens.

#### Mitochondrial respiratory chain and respiratory chain complex I mitochondrial

About 90% of cellular energy, in the form of adenosine-tri-phosphate (ATP), is produced inside mitochondria. Mitochondria also play a vital role in cellular processes such as the regulation of reactive oxygen, calcium homeostasis, programmed cell death and metabolic processes. The classical mitochondrial respiratory chain is composed of four complexes (Complex I-IV) [70]. *A. fumigatus* and *C. albicans* down-regulated 10 unique genes in the “Mitochondrial Respiratory Chain” (GO:0005746) and “Respiratory Chain Complex I Mitochondrial” (CORUM) gene sets. Studies have shown that mitochondrial respiratory chain has an important role in antiviral processes. A decrease in Coxsackievirus B3 (CVB3) viral load was observed with an increase in mitochondrial complexes I in a mouse model [71]. Although we did not find literature evidence describing the role of the mitochondrial respiratory chain in bacterial and fungal infections, the perturbation of this gene set, in our analysis, may shed some light on its importance. The down-regulation of the mitochondrial respiratory chain gene set might favor the pathogenesis of *A. fumigatus* and *C. albicans*.

### Immune response-inducing and -repressing gene sets

In the preceding section, we discussed the immunological relevance of 13 consistently and commonly perturbed gene sets. Among these gene sets, we observed that the up-regulation of seven gene sets namely, “Adaptive immune response”, “Granulocytes pathway”, “Viral genome replication”, “Positive regulation of immune response”, “Cd28 dependent Pi3K Akt signaling”, “Peptidyl tyrosine phosphorylation”, and “Cdmac pathway” favor the host immune response. On the other hand, the up-regulation of gene sets such as “Dissolution of fibrin clot”, “Inactivation of mapk activity” and “Hedgehog pathway up” and the down-regulation of “Lsm1-7 complex”, “Respiratory Chain Complex I Mitochondrial”, and “Mitochondrial Respiratory Chain” disfavor the host immune response. In the same way, the perturbed genes also fall into two groups. For instance, genes such as MALT1, SERPINE1, ICAM1, and IL8 have a positive impact on the immune response when up-regulated, while the up-regulation of genes such as DUSP8, DUSP6 and SPRED2 may negatively impact the host immune response. We refer to the first set of gene sets/genes as immune response-inducing and to the second set as immune response-repressing gene sets/genes.

**Figure 3:**
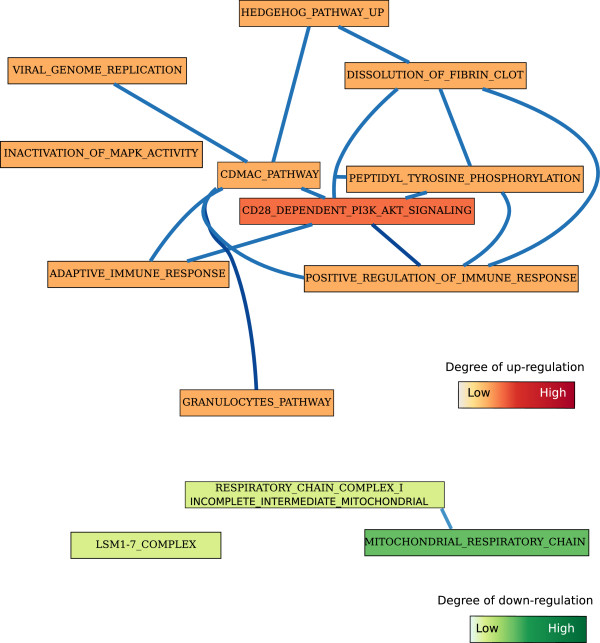
**Network of gene sets.** A network of immune response-inducing and -repressing gene sets. Gene sets are represented with rectangles. Gene sets are connected by an edge if they are interacting.

**Figure 4:**
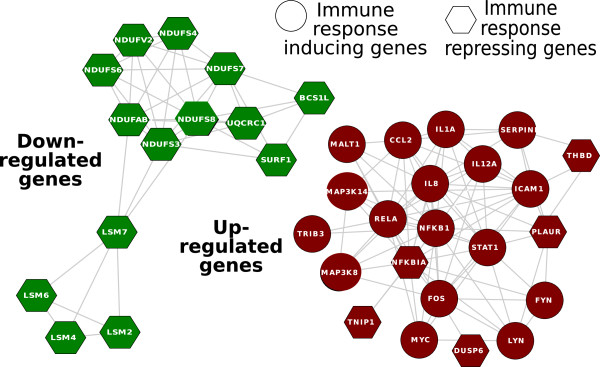
**Network of genes.** A network of immune response-inducing and -repressing genes. In this diagram, circles represent immune response-inducing genes and hexagons represent immune response-repressing genes. Genes are connected by an edge if they are functionally interacting (interaction information was obtained from the STRING database [74]).

We generated a network of these gene sets using the Markov Chain Monte Carlo-Biological Process Networks (MCMC-BPN) method developed by Lasher *et al.*[72] (Figure 3). Given gene expression data, protein-protein interactions, functional annotations of genes, and a set of biological processes, MCMC-BPN first creates a network among all pairs of processes that cross annotate at least one gene. Then, it seeks to find the smallest number of process links that explain as many as perturbed interactions as possible. We also created a functional interaction network among the genes using Cytoscape [73] and data obtained from the STRING database [74] (Figure 4). The goal is to identify highly interacting gene sets and genes, upon which we can prioritize immunomodulatory strategies. We noticed that “Cd28 dependent Pi3K Akt signaling” and “Cdmac pathway” are the top-two highly connected gene sets (Figure 3). In addition, genes such as such as NF- *κ*B1 and LSM-7 have many interactors. In general, an immunomodulatory strategy against *A. fumigatus* and *C. albicans* might focus on increasing the expression level of genes in the immune response-inducing category and/or suppressing immune response-repressing genes.

## Methods

### Gene expression data sets

We obtained 307 distinct taxonomic names of fungal pathogens from Woolhouse and Gowtage-Sequeria [1]. We queried the GEO meta database [17] and ArrayExpress [18]) using these taxonomic names as keywords. We filtered the data using the following criterion. In short, we removed time-course data, we excluded data sets with less than six samples, we retained data only from three host species namely, *Homo sapiens*, *Mus musculus* and *Rattus norvegicus*, and we removed data that did not involve healthy and infected samples. We obtained nine data sets. The data sets spanned across five fungal pathogens, namely, *A. alternata*, *A. fumigatus*, *C. albicans*, *P. jirovecii*, and *S. chartarum*, and seven target cell/tissue types including macrophages, epithelial, dendritic cells, monocytes, neutrophils, endothelial cells, and lung (Table 1). We downloaded gene expression data and normalized using the GC Robust Multi-array Average (GCRMA) procedure.

### Functional annotations

Functional annotation data sets were collected from four sources namely, National Cancer Institute-Nature Pathway Interaction Database (NCI-PID), NetPath database (NetPath), CORUM Database of Mammalian Protein Complexes (CORUM), and Molecular Signature Database (MsigDB). Collectively these were referred to as gene sets in our analysis.

### Computation of bicluster genes

In GSEA, the leading edge genes for a gene set-pathogen pair (*GS*_*i*_,*P*_*j*_) is defined as the set of genes that contribute the most to the perturbation of a gene set by a pathogen. The leading edge genes for a gene set constitute those genes that appear in the ranked list of genes at or before the point where the running sum reaches its maximum deviation from zero [25]. On the basis of the leading edge genes, we intended to define *bicluster genes* which can be interpreted as the core of gene sets that accounts for the perturbation of the gene sets by the pathogens in a bicluster.

Consider a bicluster *b* that consists of *m* gene sets and *n* pathogens, and let *L*_*i*,*j*_ be the set of leading edge genes for the *i*^*th*^ gene set and *j*^*th*^ pathogen in *b*. We computed the set of *bicluster genes* in the following way: First, found the intersection of the sets *L*_*i*,*j*_ across all the pathogens for each gene set in a bicluster, and we denoted the resulting set by *L*_*i*_. Then, we found the union of the sets *L*_*i*_*s* across all member gene sets which gave us *bicluster genes* denoted by *BG*_*b*_. Table 5 demonstrates this process for a bicluster that contains two gene sets and two pathogens.

**Table 5:** **Computation of*bicluster genes***

	***P***_**1**_		***P***_**2**_	
*GS*_1_	*L*_11_	∩	*L*_12_	*L*_1_
				∪
*GS*_2_	*L*_21_	∩	*L*_22_	*L*_2_
				*BG*_*b*_

Computation of *bicluster genes* for a 2*X*2 bicluster. *L*_*i,j*_ represents the leading-edge genes for a pathogen-gene set pair. *L*_*i*_ represents leading edge genes for the *i*^*th*^ gene set over all the pathogens in the bicluster. *BG*_*b*_ represents the *bicluster genes* for bicluster.

### Computation of consistently and commonly perturbed gene sets

**Figure 5:**
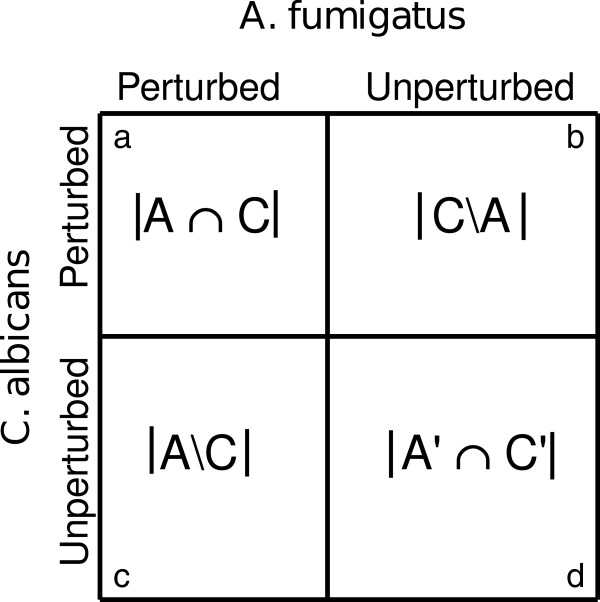
**Contingency table of genes.** Contingency table of perturbed/unperturbed genes by *A. fumigatus* and *C. albicans*, **(a)** |*A*∩*C*|: the number of genes in the gene set perturbed by both *A. fumigatus* and *C. albicans*, **(b)** |*C*∖*A*|: the number of genes perturbed by *C. albicans* but not by *A. fumigatus*, **(c)** |*A*∖*C*|: the number of genes perturbed by *A. fumigatus* but not by *C. albicans*, and **(d)** |*A*^′^∩*C*^′^|: the number of genes unperturbed by both pathogens.

Pathogens may not perturb gene sets in a bicluster in the same manner, i.e., the number of perturbed genes may be skewed towards one of the pathogens, or the perturbation may be similar across the two pathogens, assuming two pathogens in a bicluster. We intended to discover gene sets where the number of genes perturbed by two pathogens remained similar. We converted this into a contingency table as shown in Figure 5. We used the McNemar’s chi-square test statistic as an assessment criterion [75]. A smaller test statistic result indicated that the number of perturbed genes was similar for the two pathogens whereas a larger test statistic indicated that the number of genes perturbed by the two pathogens are dissimilar. For a contingency table such as that shown in Figure 5, the McNemar’s chi-square test statistic is given by the formula shown below. According to R Statistical Package, a McNemar’s chi-squared value of less than one is rounded to zero.

$$\chi^{2}=\frac{{\left(|b-c|-1\right)}^{2}}{b+c}$$

## Conclusions

In this study, we used a combination of gene set enrichment analysis and biclustering in the prediction of common immunomodulators that can potentially be activated or repressed in order to make the host more tolerant to fungal infections. Although this work leveraged on existing computational techniques, to the best of our knowledge, it is the first one to use similar computational approaches in the prediction of broad-spectrum immunomodulators. In this study, we tried to address one of the most important problems in the treatment of infectious diseases, *i.e.* drug resistance. The current paradigm in the fight against drug-resistance is targeting host genes and proteins instead of pathogen targets. This involves manipulating or subverting biological processes in the host that pathogens utilize. We have developed a computational approach for the identification of common host response mechanisms, which is an important step in host-oriented treatment paradigms.

Our approach is based on detecting consistently perturbed gene sets among gene sets commonly perturbed by pathogens guided by McNemar’s chi-square test of dependence. The hypothesis behind our approach is that such gene sets might capture biological processes that are commonly and consistently involved in the host-pathogen interaction, and that such gene sets may contain potential putative broad-spectrum immunomodulators. Using this approach, we produced a ranking of gene sets containing immunomodulators, although we focused our analysis on ten highly consistently perturbed gene sets.

In this study, we identified genes that are positively and negatively correlated with the host immune response; we called them immune response-inducing and -repressing genes respectively (Table 6). Immune response -inducing genes were genes whose up-regulation increased the host’s immune response and immune response-repressing genes had an opposite effect. We believe that the perturbation of both groups of gene sets and genes are important in maintaining the balance between clearance, colonization and invasion in immunocompetent individuals.

**Table 6:** Predicted immunomodulatory activities

**Gene**	**Immunomodulatory Activity**	**Pathogen**	**Ref**
**Immune response-inducing genes**			
MALT1	Inhibition of MALT1 stopped c-Rel activation and Th-17 immunity to *Candida* species	*C. albicans*	[33]
SERPINE1	SERPINE1 Inhibits plasmin formation. Plasmin bound *C. albicans* showed increased invasiveness.	*C. albicans*	[35]
ICAM1	Inhibition of ICAM-1 stopped adherence of *C. albicans* to human gingival epithelial cells (HGECs) and resulted in a decreased activation of pro-inflammatory cytokine IL-8	*C. albicans*	[38]
IL8	see ICAM1		[38]
IL1A	IL-1 *α* from *C. albicans* infected oral epithelial cells up-regulated secretion IL-8 and granulocyte colony-stimulating factor (GM-CSF)	*C. albicans*	[41]
FYN	FYN knockout mice were shown to have increased Th2 type immune response		[54]
STAT1	Defects in STAT1 result in defective Th17-type and Th1-type responses		[63,64]
CCL2	Neutralizing CCL2 resulted in increased mortality and pathogen burden in the lungs of mice with invasive aspergillosis	*A. fumigatus*	[46]
MAP3K8	MAP3K8 deficient mice exhibited low-level of TNF- *α* and ERK production		[60]
MAP3K14	MAP3K14 Activated MAPKs		[61]
NFKB1	Component of NF- *κ*B		
RELA	Component of NF- *κ*B		
EREG	EREG is important in the proliferation and differentiation of macrophages. It was up-regulated when alveolar macrophages were challenged with conidia of *A. fumigatus*	*A. fumigatus*	[58]
IL12A	Central for the induction of TH1-type cytokines		[63,64]
TRIB3	TRIB3 senses the presence of cellular nutrient in PI3K/AKT signaling		[62]
CLCF1	Activates the Jak-STAT signaling cascade?		
LYN	Inhibiting the ICAM-1-binding activity?		
**Immune response-repressing genes**			
PLAUR	PLAUR promotes plasmin formation. Plasmin bound *C. albicans* showed increased invasiveness.	*C. albicans*	[35]
DUSP8	DUSP8 inhibits MAPK/ERK. Inactivation of MAPK/ERK correlated with a decrease in the activation of innate immunity against *A. fumigatus* in a mice model	*A. fumigatus*	[43]
DUSP6	DUSP6 inhibits MAPK/ERK (see DUSP8)		
SPRED2	SPRED2 inhibits MAPK/ERK (see DUSP8)		
NFKBIA	Inhibits NF- *κ*B. Inhibition of NF- *κ* decreased neutrophil phagocytosis and microbicide capacity	*C. albicans*	[50]
TNIP1	Inhibits NF- *κ*B		[49]
PMP22	Up-regulation of Hedgehog pathway increased cellular permissiveness for hepatitis C virus replication		[52]
THBD	Up-regulation of Hedgehog pathway increased cellular permissiveness for hepatitis C virus replication		[52]

Genes and their predicted immunomodulatory activities for up-regulated bicluster B1.

Our approach has enabled the identification of previously known immunomodulators such as MALT1, suggesting the validity of the computational techniques that we implemented in this study. Currently, our analysis is limited by the number of publicly available gene expression data sets pertaining to host-response to fungal infections. In the future, we hope to expand the approach to include more fungal pathogens as new data sets become available. Also, our analyses suffer from heterogeneity in the transcriptional data sets utilized in this study, e.g., microarray platforms, cell types, and temporal aspects. We believe that standardized experimental designs may improve the reliability and robustness of such analyses.

We acknowledge that tissue specificity is an important aspect of host response. However, we did not find an adequate number of data sets to investigate tissue specific responses. We found nine data sets that met our selection criterion. These data sets spanned across seven target cell/tissue types including macrophages, epithelial, dendritic cells, monocytes, neutrophils, endothelial cells, and lung. The number of data sets was very small and sparse to perform a statistically sound analysis pertaining to tissue-specific host responses. However, the computational method we developed is capable of detecting tissue-specific host responses by computing biclusters’ enrichment in tissue/cell types. We demonstrated this in our previous work on a large host response gene expression data set related to bacterial infection as shown on the supplementary website [76].

## Availability

Links to biclusters are available at http://bioinformatics.cs.vt.edu/~murali/supplements/2013-kidane-bmc.

## Competing interests

The authors declare that there are no competing interests.

## Authors’ contributions

All authors proposed and designed the study. YHK implemented the approach and analyzed the results. All authors contributed to the writing of the manuscript. All authors read and approved the final manuscript.

## Supplementary Material

Additional file 1 Details of up- and down- regulated biclusters. Click here for file
