# Supplementary material for: Computational approaches for discovery of common immunomodulators in fungal infections: towards broad-spectrum immunotherapeutic interventions
Source: BMC Microbiol. 2013 Oct 7;13:224. doi: 10.1186/1471-2180-13-224 (PMC3853472; doi:10.1186/1471-2180-13-224)
Supplement: Additional file 1 — Details of up- and down- regulated biclusters. [file 1471-2180-13-224-S1.zip › 2013-kidane-bmc/details-of-biclusters/dnreg-biclust-8.html]

**BICLUSTER\_ID** : DNREG-8  
**PATHOGENS** /1/ : a. fumigatus  
**KNOWN DRUG TARGETS** /4/ : CHRNA4, SSTR5, CHRM5, CHRNA2  

| Gene Set | Leading Edge Genes |
| --- | --- |
| NEUROTRANSMITTER RECEPTOR ACTIVITY | CHRNA4, SSTR5, CHRM5, CHRNA2 |
| NEUROTRANSMITTER BINDING | CHRNA4, SSTR5, CHRM5, CHRNA2 |
| ACETYLCHOLINE BINDING | CHRNA4, CHRM5, CHRNA2 |
| NEUROPEPTIDE BINDING | SSTR3, SSTR5 |
| NEUROPEPTIDE RECEPTOR ACTIVITY | SSTR3, SSTR5 |
| DETECTION OF CHEMICAL STIMULUS | UGT2A1 |

| Color legend | | | | | | | | | | | |
| --- | --- | --- | --- | --- | --- | --- | --- | --- | --- | --- | --- |
| q-value | -1 | -0.2 | -0.05 | -0.01 | -0.001 | -0.0001 |
| Color |  |  |  |  |  |  |

TABLE OF Q-VALUES

| aspergillus fumigatus conidia a549 | aspergillus fumigatus 16hbe14o | Gene Set |
| --- | --- | --- |
| -0.0041162646 | -0.0037597455 | NEUROTRANSMITTER\_RECEPTOR\_ACTIVITY |
| -0.029299565 | -0.00438641 | NEUROTRANSMITTER\_BINDING |
| -0.18649735 | -0.06708722 | ACETYLCHOLINE\_BINDING |
| -0.14514485 | -0.14210184 | NEUROPEPTIDE\_BINDING |
| -0.13338187 | -0.1392568 | NEUROPEPTIDE\_RECEPTOR\_ACTIVITY |
| -0.14939642 | -0.1644083 | DETECTION\_OF\_CHEMICAL\_STIMULUS |
