# Supplementary material for: Computational approaches for discovery of common immunomodulators in fungal infections: towards broad-spectrum immunotherapeutic interventions
Source: BMC Microbiol. 2013 Oct 7;13:224. doi: 10.1186/1471-2180-13-224 (PMC3853472; doi:10.1186/1471-2180-13-224)
Supplement: Additional file 1 — Details of up- and down- regulated biclusters. [file 1471-2180-13-224-S1.zip › 2013-kidane-bmc/details-of-biclusters/upreg-biclust-0.html]

**BICLUSTER\_ID** : UPREG-0  
**PATHOGENS** /2/ : c. albicans,a. fumigatus  
**KNOWN DRUG TARGETS** /43/ : ATP2C1, PPP2CB, SLC7A11, GFPT2, NFKB1, GPRC5A, PPIF, SLC22A4, PPARG, GSK3B, FYN, SERPINE1, MET, ACVR1, CANT1, ICAM1, ABCG1, CCL20, EPHA2, UAP1, CTPS, LDLR, PDE4B, CCL2, CASP7, AMPD3, SLC7A1, CD55, PIM1, TXNRD1, STAT1, WARS, THBD, INPP1, TOP1, TFPI, EGLN3, IL6, PLOD2, PTGS2, MAPKAPK2, IL8, PLAUR  

| Gene Set | Leading Edge Genes |
| --- | --- |
| NETPATH TGFBETA RECEPTOR PATHWAY UP | MAPK7, HBEGF, CLCF1, NFKBIA, EIF2C2, INHBA, RGS2, PDGFA, EDN1, MAPK6, TNFRSF12A, CYR61, SERPINE1, HIVEP2, ABCG1, IL1RAP, CTPS, RELA, FOSL1, AKAP12, TOM1, IER3, DUSP5, KLF10, SPRY2, TM4SF1, IL6, ATF3, PTGS2, PMP22, SLC7A5, SERPINB8, DDX21, TRIB3, MAP3K8, CHST11, PHLDA2, SLC7A11, TSPAN13, GFPT2, GTPBP2, EXT1, RELB, NEDD4, ENC1, ITGA2, PDK3, TOPORS, SERPINB9, FYN, RYBP, ACVR1, CRY1, ICAM1, TFPI2, RASAL2, MYC, RAI14, GADD45B, UAP1, ITGA6, SOX4, RP2, SLC7A1, PIM1, FHL2, KIAA0247, NRIP1, ATP1B1, ETS2, PLOD2, HMGCS1, TANK, MAPKAPK2, DAXX, PLAUR |
| SIGNAL TRANSDUCTION | MAPK7, RRAGC, EREG, HBEGF, DUSP6, HUS1, CLCF1, HOMER1, PMAIP1, OSMR, HIPK2, INHBA, CFLAR, RGS2, PDGFA, CXCL1, EDN1, PPARG, GSK3B, MAPK6, MOAP1, TNFSF9, TNFAIP3, MAP4K4, SOCS2, BNIP3, TTRAP, CCL20, EPHA2, DUSP10, CCL2, RELA, CASP7, SQSTM1, TRIP10, AKAP12, ALCAM, SPRED2, STAT1, REL, RGS12, KLF10, EIF2AK3, INPP1, RHEB, MALT1, TGFA, IL6, PSEN1, TICAM1, ATP2C1, OPN3, TRAF4, SIAH2, TP53BP2, GTPBP2, EXT1, RIPK2, CREM, GPRC5A, TRAF3, RIPK1, STK17A, RGS20, FYN, SERPINB9, GEM, ACVR1, CANT1, RASAL2, CD83, DUSP4, GADD45B, STAT4, TLE1, BIRC2, MAPRE2, DUSP8, VAPA, BIRC3, CXCL5, TANK, MAPKAPK2, DAXX, IL8, EPS8 |
| NETPATH IL 2 PATHWAY UP | DDX21, PFKFB3, ABCE1, SFPQ, NOLC1, BTG3, DUSP6, PMAIP1, RRAS2, FOS, CREM, CFLAR, NFKB1, IFIT1, MFHAS1, DENND3, FYN, SERPINE1, ICAM1, IRF1, MYC, SOCS2, SACS, DUSP4, LDLR, PDE4B, STAT4, CTPS, RELA, NR4A2, PIM1, TXNRD1, PPRC1, IER3, STAT1, KLF6, DUSP5, LIF, WARS, TOP1, TRIM21, ATP1B1, ETS2, IL6, MAPKAPK2, DAXX, IL8, SLC7A5, CHSY1, PLAUR |
| NEGATIVE REGULATION OF BIOLOGICAL PROCESS | RND1, EREG, TP53BP2, BTG3, ARID5B, CLCF1, OSGIN1, INHBA, CFLAR, NFKB1, TNIP1, CXCL1, FOSB, ZHX2, BTG1, GSK3B, IL1A, BCL2A1, TNFAIP8, SEMA4D, SERPINB9, RYBP, TNFAIP3, SERPINE1, ACVR1, SOCS2, MYC, ZBTB17, BNIP3, TLE1, CCL2, GADD45A, RELA, PLAGL1, PIM1, E2F6, IER3, BIRC3, ARHGEF2, LIF, KLF10, PPP1R15A, WARS, EIF2AK3, ANGPTL4, IL6, PSEN1, DAXX, IL8 |
| NETPATH IL 1 PATHWAY UP | NR4A3, CCL20, CCL2, PLK1, DUSP6, RELA, FOSL1, CXCL3, NFKBIA, AMPD3, UST, CXCL2, TXNRD1, SOD2, NFKB1, BIRC3, LIF, CXCL1, CXCL5, IL6, PTGS2, SERPINE1, IL8, MYC |
| RESPONSE TO EXTERNAL STIMULUS | ELF3, CCL20, IL1RAP, EREG, CCL2, RELA, FOSL1, RIPK2, FOS, CXCL2, ITGA2, NFKB1, CXCL1, THBD, PPARG, IL1A, TFPI, CXCL5, SERPINE1, IL8, PLAUR |
| NEGATIVE REGULATION OF CELLULAR PROCESS | RND1, EREG, TP53BP2, BTG3, ARID5B, CLCF1, OSGIN1, INHBA, CFLAR, NFKB1, CXCL1, FOSB, ZHX2, BTG1, GSK3B, IL1A, BCL2A1, TNFAIP8, SEMA4D, SERPINB9, RYBP, TNFAIP3, ACVR1, SOCS2, MYC, ZBTB17, TLE1, CCL2, GADD45A, RELA, PLAGL1, PIM1, E2F6, IER3, BIRC3, ARHGEF2, LIF, WARS, KLF10, PPP1R15A, EIF2AK3, ANGPTL4, IL6, PSEN1, DAXX, IL8 |
| NETPATH IL 5 PATHWAY UP | PLSCR1, PDE4B, SFPQ, CCL2, HBEGF, DUSP6, GADD45A, RELA, EGR1, CD55, PIM1, RELB, IER3, NFKB1, DUSP5, REL, PPIF, TRAF3, MAPK6, IL1A, RAB21, PMP22, ICAM1, IL8 |
| REACTOME SIGNALING IN IMMUNE SYSTEM | DUSP4, TRIB3, MAPK7, NFKB2, PPP2CB, ITGA6, MAP3K8, DUSP6, NRAS, MEF2A, RELA, MAP3K14, SLC7A11, NFKBIA, KRAS, RIPK2, FOS, NFKB1, RIPK1, THBD, MALT1, ATP1B1, FYN, PVRL2, MAPKAPK2, ICAM1, SLC7A5, TICAM1 |
| NETPATH IL 4 PATHWAY UP | CCL2, TP53BP2, ELL2, ARID5B, DUSP6, FJX1, RELA, MAP3K14, RIPK2, FOS, CREM, RGS2, NFKB1, GATA6, REL, SLC22A4, TFPI, EGLN3, IL6, MET, MYC |
| NEGATIVE REGULATION OF APOPTOSIS | CCL2, RELA, CLCF1, PIM1, IER3, CFLAR, NFKB1, BIRC3, IL1A, GSK3B, ANGPTL4, BCL2A1, SEMA4D, TNFAIP8, IL6, SERPINB9, ACVR1, PSEN1, SOCS2 |
| KEGG CYTOKINE CYTOKINE RECEPTOR INTERACTION | CCL20, CCL2, CLCF1, CXCL3, OSMR, INHBA, CXCL2, PDGFB, PDGFA, CXCL1, TNFRSF12A, IL1A, CXCL5, INHBC, TNFSF9, IL6, MET, ACVR1, IL8 |
| NEGATIVE REGULATION OF PROGRAMMED CELL DEATH | CCL2, RELA, CLCF1, PIM1, IER3, CFLAR, NFKB1, BIRC3, IL1A, GSK3B, ANGPTL4, BCL2A1, SEMA4D, TNFAIP8, IL6, SERPINB9, ACVR1, PSEN1, SOCS2 |
| RESPONSE TO WOUNDING | ELF3, CCL20, EREG, IL1RAP, RELA, RIPK2, FOS, CXCL2, ITGA2, NFKB1, CXCL1, THBD, TFPI, IL1A, SERPINE1, IL8 |
| PROTEIN KINASE CASCADE | ATP2C1, DUSP4, GADD45B, STAT4, DUSP10, CCL2, BIRC2, DUSP6, RELA, CLCF1, SQSTM1, HIPK2, DUSP8, RIPK2, SPRED2, VAPA, CFLAR, STAT1, REL, STK17A, MALT1, TGFA, FYN, TNFAIP3, MAPKAPK2, DAXX, CANT1, MAP4K4, TICAM1, SOCS2 |
| NETPATH TNF ALPHA PATHWAY DOWN | ABCG1, CCL20, IFIT5, MAP3K14, CXCL3, NFKBIA, EGR1, SDC4, CXCL2, IER3, NFKB1, KLF6, REL, CXCL1, KLF10, PPP1R15A, ZHX2, FOSB, MAPK6, GSK3B, DDX3X, TNFAIP2, MAFF, IL6, GEM, PTGS2, TNFAIP3, IRF1 |
| KEGG CHEMOKINE SIGNALING PATHWAY | NFKB1, STAT1, CCL20, CXCL1, CCL2, GSK3B, RELA, CXCL3, CXCL5, NFKBIA, IL8, CXCL2 |
| NEGATIVE REGULATION OF DEVELOPMENTAL PROCESS | EREG, CCL2, RELA, CLCF1, TXNDC5, INHBA, PIM1, IER3, CFLAR, NFKB1, BIRC3, ANGPTL4, IL1A, GSK3B, BCL2A1, TNFAIP8, SEMA4D, IL6, SERPINB9, ACVR1, PSEN1, SOCS2, BNIP3 |
| IMMUNE SYSTEM PROCESS | CD83, CCL20, EREG, CCL2, MALT1, SEMA4D, IL6, FYN, GEM, TNFAIP1, INHBA, IL8 |
| NCI REG GR PATHWAY | SGK, NFKB1, STAT1, GSK3B, RELA, EGR1, IL6, IL8, IRF1, FOS |
| NETPATH IL 4 PATHWAY DOWN | PDGFB, CCL20, GADD45B, CCL2, NFKBIA, IL6, GEM, PTGS2, CXCL2, IRF1, IL8 |
| NETPATH KIT RECEPTOR PATHWAY UP | DUSP4, PPP2CB, CCL2, RELA, TFAP2C, IL6, EGR1, PIM1, FOS, MYC, TXNRD1, IER3 |
| KEGG NOD LIKE RECEPTOR SIGNALING PATHWAY | NFKB1, CXCL1, CCL2, NFKBIA, IL6, TNFAIP3, RIPK2, CXCL2, IL8 |
| NCI IL23PATHWAY | NFKB1, NFKBIA, STAT1, IL6, CXCL1, STAT4, CCL2, RELA |
| EXTRACELLULAR SPACE | CCL20, CXCL1, EREG, CCL2, EDN1, FJX1, CXCL3, TNFAIP2, IL6, CXCL2, IL8 |
| NCI LYSOPHOSPHOLIPID PATHWAY | NFKB1, HBEGF, GSK3B, RELA, NFKBIA, IL6, PRKCD, IL8, FOS, GNAI1 |
| BEHAVIOR | CCL20, CXCL1, CCL2, FOSB, FOSL1, CXCL5, CXCL2, IL8, PLAUR |
| NETPATH B CELL RECEPTOR PATHWAY UP | RHOB, EPHA2, MAPK7, LDLR, EREG, CCL2, TP53BP2, NR4A2, EGR1, DUSP8, PIM1, FOS, CFLAR, BIRC3, ELF4, CSNK1A1, BCL2A1, RAB5A, TNFAIP3, BCAR3, SOCS2, MYC |
| IMMUNE RESPONSE | CD83, CCL20, EREG, CCL2, MALT1, IL6, FYN, GEM, TNFAIP1 |
| LOCOMOTORY BEHAVIOR | FOSL1, CXCL5, CCL20, CXCL1, CCL2, IL8, CXCL2, PLAUR |
| NETPATH EGFR1 PATHWAY UP | EMP1, NR4A3, DUSP4, EREG, PPP1R10, DUSP6, PHLDA2, GADD45A, EGR1, AKAP12, SDC4, ITGA2, IER3, GPRC5A, PHLDA1, LIF, SPRY2, EHD1, CXCL5, PTGS2, TNFAIP3, MET, TFPI2, MYC, PLAUR, DUSP1 |
| REACTOME CLASS A1 RHODOPSIN LIKE RECEPTORS | OPN3, CCL20, CXCL1, CCL2, EDN1, CXCL3, CXCL5, IL8, CXCL2 |
| REACTOME PEPTIDE LIGAND BINDING RECEPTORS | CCL20, CXCL1, CCL2, EDN1, CXCL3, CXCL5, IL8, CXCL2 |
| CYTOKINE ACTIVITY | CXCL3, CXCL5, CCL20, CXCL1, CCL2, IL8, CXCL2, SPRED2 |
| CHEMOKINE ACTIVITY | CXCL3, CXCL5, CCL20, CXCL1, CCL2, CXCL2, IL8 |
| DEFENSE RESPONSE | CD83, ELF3, CCL20, EREG, IL1RAP, RELA, FOSL1, VEZF1, INHBA, CXCL2, FOS, RIPK2, NFKB1, TNIP1, CXCL1, IL1A, IL8, BNIP3 |
| REACTOME CHEMOKINE RECEPTORS BIND CHEMOKINES | CXCL3, CXCL5, CCL20, CXCL1, CCL2, CXCL2, IL8 |
| NCI NFAT TFPATHWAY | FOSL1, EGR1, PTGS2, CBLB, IL8, FOS, PPARG |
| NCI AMB2 NEUTROPHILS PATHWAY | NFKB1, IL6, CYR61, PLAUR |
| KEGG T CELL RECEPTOR SIGNALING PATHWAY | NFKB1, NFAT5, MAP3K8, MALT1, GSK3B, RELA, MAP3K14, NFKBIA, FYN, PPP3CC, CBLB, FOS |
| NCI IL2 1PATHWAY | STAT1, FYN, MAPKAPK2, FOS, SOCS2, MYC |
| REACTOME GPCR LIGAND BINDING | BDKRB1, OPN3, CCL20, CXCL1, EDN1, CCL2, FZD6, CXCL3, CXCL5, IL8, CXCL2 |
| KEGG TOLL LIKE RECEPTOR SIGNALING PATHWAY | NFKB1, NFKBIA, IL6, TRAF3, IL8, MAP3K8, TICAM1, RELA |
| RECEPTOR BINDING | JMJD1C, SDCBP, CCL20, EREG, CCL2, HBEGF, CLCF1, CXCL3, SQSTM1, OSGIN1, INHBA, CXCL2, CSF2, ALCAM, SPRED2, CXCL1, EDN1, TGFA, CXCL5, SEMA4D, EFNA1, INHBC, DAXX, IL8, PLXNC1, SOCS2 |
| KEGG JAK STAT SIGNALING PATHWAY | STAT1, LIF, STAT4, SPRY2, CLCF1, OSMR, IL6, CBLB, PIM1, SOCS2, MYC, SPRY1, SPRED2 |
| REACTOME TOLL LIKE RECEPTOR 3 CASCADE | NFKB1, DUSP4, MAPK7, NFKB2, PPP2CB, RIPK1, DUSP6, MEF2A, RELA, NFKBIA, MAPKAPK2, FOS, TICAM1 |
| CHEMOKINE RECEPTOR BINDING | CXCL3, CXCL5, CCL20, CXCL1, CCL2, CXCL2, IL8 |
| KEGG EPITHELIAL CELL SIGNALING IN HELICOBACTER PYLORI INFECTION | NFKB1, CXCL1, HBEGF, RELA, TJP1, MAP3K14, NFKBIA, MET, IL8 |
| NETPATH IL 3 PATHWAY UP | CCL2, PIM1, IL8, SOCS2, MYC |
| KEGG LEISHMANIA INFECTION | NFKB1, STAT1, NFKBIA, PTGS2, FOS, IL1A, RELA |
| NETPATH IL 6 PATHWAY UP | GADD45B, CXCL1, LDLR, PPARG, MAFF, PIM1, FOS, IRF1, SOCS2 |
| G PROTEIN COUPLED RECEPTOR BINDING | CXCL3, CXCL5, CCL20, CXCL1, CCL2, CXCL2, IL8 |
| INFLAMMATORY RESPONSE | NFKB1, ELF3, CCL20, CXCL1, IL1RAP, IL1A, RELA, IL8, RIPK2, CXCL2, FOS |
| BIOCARTA STEM PATHWAY | IL6, IL8 |
| NETPATH IL 9 PATHWAY UP | CCL2, PIM1, MYC, SOCS2 |
| NCI TNFPATHWAY | NFKB1, STAT1, BIRC3, RIPK1, MAP3K7IP2, BIRC2, RELA, SQSTM1, TNFAIP3, MAP4K4 |
| BIOCARTA IL1R PATHWAY | MAP3K14, NFKB1, NFKBIA, IL6, IL1A, RELA |
| NCI DISSOLUTION OF FIBRIN CLOT | SERPINE1, PLAUR |
| VIRAL REPRODUCTION | TNIP1, CCL2, IL8 |
| BIOCARTA IL17 PATHWAY | IL6, IL8 |
| REACTOME G ALPHA I SIGNALLING EVENTS | BDKRB1, OPN3, CCL20, CXCL1, CXCL3, CXCL5, CXCL2, IL8, GNAI1 |
| NCI IL12 2PATHWAY | NFKB1, STAT1, GADD45B, STAT4, RELA, RIPK2, FOS, RELB |
| VIRAL INFECTIOUS CYCLE | TNIP1, CCL2, IL8 |
| KEGG MAPK SIGNALING PATHWAY | DUSP4, GADD45B, MAPK7, DUSP10, MAP3K8, DUSP6, GADD45A, RELA, MAP3K14, DUSP14, PPP3CC, RRAS2, DUSP8, RELB, FOS, PDGFB, NFKB1, DUSP5, PDGFA, IL1A, MAPKAPK2, DAXX, MYC, DUSP1 |
| VIRAL GENOME REPLICATION | TNIP1, CCL2, IL8 |
| BIOCARTA CYTOKINE PATHWAY | IL6, IL8, IL1A |
| BIOCARTA NTHI PATHWAY | MAP3K14, NFKB1, NFKBIA, IL8, DUSP1, RELA |
| JAK STAT CASCADE | STAT1, STAT4, CCL2, SOCS2, CLCF1 |
| REACTOME TRAF6 MEDIATED INDUCTION OF THE ANTIVIRAL CYTOKINE IFN ALPHA BETA CASCADE | NFKB1, DUSP4, MAPK7, PPP2CB, DUSP6, MEF2A, RELA, NFKBIA, FOS, TICAM1 |
| KEGG RIG I LIKE RECEPTOR SIGNALING PATHWAY | NFKB1, TRAF3, DDX3Y, RELA, DDX3X, NFKBIA, TANK, IL8, DDX58 |
| REGULATION OF RESPONSE TO STIMULUS | FYN, EREG, IL8, MALT1 |
| BIOCARTA INFLAM PATHWAY | IL6, PDGFA, IL8, CSF2, IL1A |
| BIOCARTA GRANULOCYTES PATHWAY | ICAM1, IL8, IL1A |
| BIOCARTA NFKB PATHWAY | MAP3K14, NFKB1, NFKBIA, TNFAIP3, RIPK1, IL1A, RELA |
| POSITIVE REGULATION OF CELLULAR PROTEIN METABOLIC PROCESS | IL6, EREG, CDC42EP2, SAMD4A, CLCF1 |
| NETPATH IL 7 PATHWAY UP | CXCL3, CXCL5, CXCL1, TRAF3, IL8, CXCL2, MYC |
| ST ERK1 ERK2 MAPK PATHWAY | NFKB1, DUSP4, TRAF3, MAP3K8, DUSP6 |
| BIOCARTA CD40 PATHWAY | MAP3K14, NFKB1, NFKBIA, TNFAIP3, TRAF3, DUSP1, RELA |
| BIOCARTA ERYTH PATHWAY | IL6, CSF2, IL1A |
| NCI CHEMOKINE RECEPTORS BIND CHEMOKINES | CCL20 |
| REGULATION OF IMMUNE SYSTEM PROCESS | FYN, EREG, INHBA, MALT1 |
| NETPATH HEDGEHOG PATHWAY UP | PMP22, THBD, MYC |
| POSITIVE REGULATION OF IMMUNE SYSTEM PROCESS | FYN, EREG, MALT1 |
| BIOCARTA CARDIACEGF PATHWAY | NFKB1, EDN1, FOS, MYC, RELA |
| PEPTIDYL TYROSINE MODIFICATION | STAT1, IL12A, LYN, CLCF1 |
| NCI TCRCALCIUMPATHWAY | FOSL1, PTGS2 |
| NCI ATF2 PATHWAY | IL6, ATF3, DUSP5, DUSP10, FOS, DUSP8, DUSP1 |
| NETPATH EGFR1 PATHWAY DOWN | IFIT1, EREG, BTG1, PPARG, ARID5B, SQSTM1, CLDN1, IER3 |
| BIOCARTA CCR5 PATHWAY | CCL2, FOS |
| BIOCARTA CDMAC PATHWAY | NFKB1, NFKBIA, FOS, MYC, RELA |
| REACTOME CD28 DEPENDENT PI3K AKT SIGNALING | MAP3K14, FYN, TRIB3, MAP3K8 |
| CORUM CHUK-NFKB2-REL-IKBKG-SPAG9-NFKB1-NFKBIE-COPB2-TNIP1-NFKBIA-RELA-TNIP2 COMPLEX | NFKB1, NFKBIA, SPAG9, REL, TNIP1, RELA |
| POSITIVE REGULATION OF MULTICELLULAR ORGANISMAL PROCESS | FYN, EREG, MALT1 |
| PEPTIDYL TYROSINE PHOSPHORYLATION | STAT1, IL12A, LYN, CLCF1 |
| POSITIVE REGULATION OF IMMUNE RESPONSE | FYN, EREG, MALT1 |
| NCI CD40 PATHWAY | NFKB1, TTRAP, BIRC3, TRAF3, BIRC2, RELA, MAP3K14, NFKBIA, TNFAIP3, CBLB, MYC |
| POSITIVE REGULATION OF TRANSLATION | IL6, EREG, SAMD4A |
| REGULATION OF IMMUNE RESPONSE | FYN, EREG, MALT1 |
| POSITIVE REGULATION OF CELL PROLIFERATION | PDGFA, LIF, EREG, EDN1, TBC1D8, FOSL1, TGFA, CXCL5, IL6, MYC |
| BIOCARTA KERATINOCYTE PATHWAY | MAP3K14, ETS2, NFKB1, NFKBIA, PRKCH, DAXX, FOS, RELA |
| BIOCARTA TNFR2 PATHWAY | NFKB1, TRAF3, RELA, MAP3K14, NFKBIA, TNFAIP3, TANK, DUSP1 |
| NCI NFKAPPABCANONICALPATHWAY | NFKB1, CYLD, BIRC2, MALT1, RELA, NFKBIA, TNFAIP3, RIPK2, TRAF6 |
| POSITIVE REGULATION OF BIOLOGICAL PROCESS |  |
| LEUKOCYTE ACTIVATION |  |
| NETPATH IL 1 PATHWAY |  |
| EXTRACELLULAR REGION PART |  |
| REACTOME AMINO ACID AND OLIGOPEPTIDE SLC TRANSPORTERS |  |
| NCI FGF PATHWAY |  |
| KEGG HYPERTROPHIC CARDIOMYOPATHY HCM |  |
| NETPATH T CELL RECEPTOR PATHWAY DOWN |  |
| NCI IL2 STAT5PATHWAY |  |
| BIOCARTA NKT PATHWAY | IL12A, CSF2 |
| I KAPPAB KINASE NF KAPPAB CASCADE |  |
| BIOCARTA LAIR PATHWAY |  |
| NCI IL2 PI3KPATHWAY |  |
| KEGG FOCAL ADHESION |  |
| REGULATION OF I KAPPAB KINASE NF KAPPAB CASCADE |  |
| REGULATION OF PROTEIN KINASE ACTIVITY | GADD45B, TRIB3, EREG, MALT1, DUSP6, GADD45A, TRIB1, TGFA, DAXX, DUSP8, SPRED2 |
| NCI HIF1 TFPATHWAY |  |
| CORUM AKAP250-PKA-PDE4D COMPLEX |  |
| BIOCARTA LYM PATHWAY |  |
| POSITIVE REGULATION OF PROTEIN METABOLIC PROCESS |  |
| ST GRANULE CELL SURVIVAL PATHWAY |  |
| POSITIVE REGULATION OF METABOLIC PROCESS |  |
| POSITIVE REGULATION OF RESPONSE TO STIMULUS |  |
| ADAPTIVE IMMUNE RESPONSE GO 0002460 | MALT1 |
| BIOCARTA ETS PATHWAY |  |
| REGULATION OF TRANSFERASE ACTIVITY | GADD45B, TRIB3, EREG, MALT1, DUSP6, GADD45A, TRIB1, TGFA, DAXX, DUSP8, SPRED2 |
| BIOCARTA WNT PATHWAY |  |
| BIOCARTA RANKL PATHWAY |  |
| CELL CELL SIGNALING |  |
| T CELL DIFFERENTIATION |  |
| NETPATH TNF ALPHA PATHWAY |  |
| REACTOME MAP KINASES ACTIVATION IN TLR CASCADE |  |
| REGULATION OF PEPTIDYL TYROSINE PHOSPHORYLATION | CLCF1 |
| INNATE IMMUNE RESPONSE |  |
| CORUM TNF-ALPHA/NF-KAPPA B SIGNALING COMPLEX CHUK KPNA3 NFKB2 NFKBIB REL IKBKG NFKB1 NFKBIE RELB NFKBIA RELA TNIP2 |  |
| POSITIVE REGULATION OF ANGIOGENESIS | BTG1, ANGPTL4 |
| CALCIUM MEDIATED SIGNALING |  |
| BIOCARTA HIVNEF PATHWAY |  |
| NCI P38ALPHABETAPATHWAY |  |
| NCI CD8TCRDOWNSTREAMPATHWAY | FOSL1, EGR1, STAT4, FOS |
| NETPATH TGFBETA RECEPTOR PATHWAY DOWN |  |
| POSITIVE REGULATION OF CELL MIGRATION |  |
| BIOCARTA 41BB PATHWAY |  |
| NEGATIVE REGULATION OF TRANSFERASE ACTIVITY |  |
| NCI P53DOWNSTREAMPATHWAY |  |
| CORUM TNF-ALPHA/NF-KAPPA B SIGNALING COMPLEX RPL6 RPL30 RPS13 CHUK DDX3X NFKB2 NFKBIB REL IKBKG NFKB1 MAP3K8 RELB GLG1 NFKBIA RELA TNIP2 GTF2I |  |
| REACTOME CD28 CO STIMULATION |  |
| NETPATH IL 2 PATHWAY |  |
| ST TUMOR NECROSIS FACTOR PATHWAY |  |
| NCI HIF2PATHWAY |  |
| ANATOMICAL STRUCTURE DEVELOPMENT |  |
| POSITIVE REGULATION OF SIGNAL TRANSDUCTION |  |
| NEGATIVE REGULATION OF MAP KINASE ACTIVITY | DUSP8, DUSP6, SPRED2 |
| CORUM NFKB1-NFKB2-REL-RELA-RELB COMPLEX |  |
| POSITIVE REGULATION OF CELLULAR PROCESS |  |
| POSITIVE REGULATION OF PHOSPHATE METABOLIC PROCESS |  |
| REGULATION OF CATALYTIC ACTIVITY |  |
| VIRAL REPRODUCTIVE PROCESS |  |
| POSITIVE REGULATION OF CELL DIFFERENTIATION |  |
| INACTIVATION OF MAPK ACTIVITY | DUSP8, DUSP6, SPRED2 |
| NCI EPHRINBREVPATHWAY |  |
| REGULATION OF KINASE ACTIVITY | GADD45B, TRIB3, EREG, MALT1, DUSP6, GADD45A, TRIB1, TGFA, DAXX, DUSP8, SPRED2 |
| NCI NFKAPPABATYPICALPATHWAY |  |
| REACTOME DOWNSTREAM EVENTS IN GPCR SIGNALING |  |
| BIOCARTA TALL1 PATHWAY |  |
| NETPATH IL 7 PATHWAY |  |
| ADAPTIVE IMMUNE RESPONSE | MALT1 |
| ANTI APOPTOSIS |  |
| KEGG SMALL CELL LUNG CANCER |  |
| POSITIVE REGULATION OF PHOSPHORYLATION |  |
| REACTOME AMINO ACID TRANSPORT ACROSS THE PLASMA MEMBRANE |  |
| NCI REGULATED PROTEOLYSIS OF P75NTR |  |
| BIOCARTA PPARA PATHWAY |  |
| REGULATION OF CYTOKINE PRODUCTION |  |
| MAPKKK CASCADE GO 0000165 |  |
| ORGAN DEVELOPMENT |  |
| POSITIVE REGULATION OF CELLULAR METABOLIC PROCESS |  |
| INTRACELLULAR SIGNALING CASCADE |  |
| SIG CD40PATHWAYMAP |  |
| REGULATION OF CELL ADHESION |  |
| BIOCARTA RNA PATHWAY |  |
| ST FAS SIGNALING PATHWAY |  |
| CELL SURFACE RECEPTOR LINKED SIGNAL TRANSDUCTION GO 0007166 |  |
| BIOCARTA DEATH PATHWAY |  |
| REACTOME DOWNSTREAM TCR SIGNALING |  |
| BIOCARTA STRESS PATHWAY |  |
| REACTOME SLC MEDIATED TRANSMEMBRANE TRANSPORT |  |
| KEGG GAP JUNCTION | PDGFB, PDGFA, MAPK7, TUBB2B, NRAS, TJP1, KRAS, TUBB2A, TUBB6, ADCY3, GNAI1 |
| PROTEIN KINASE ACTIVITY |  |
| REGULATION OF MULTICELLULAR ORGANISMAL PROCESS |  |
| REACTOME TOLL RECEPTOR CASCADES |  |
| KEGG HEMATOPOIETIC CELL LINEAGE |  |
| REACTOME P75NTR SIGNALS VIA NFKB |  |
| NCI CD28 DEPENDENT PI3K AKT SIGNALING |  |
| NCI IL12 STAT4PATHWAY |  |
| ORGAN MORPHOGENESIS |  |
| BIOCARTA TOLL PATHWAY |  |
| REACTOME MAPK **KNOWN DRUG TARGETS** NUCLEAR EVENTS MEDIATED BY MAP KINASES |  |
| REGULATION OF MAP KINASE ACTIVITY |  |
| KEGG PATHWAYS IN CANCER |  |
| NETPATH WNT PATHWAY UP |  |
| BIOCARTA ARENRF2 PATHWAY | MAFF, FOS |

| Color legend | | | | | | | | | | | |
| --- | --- | --- | --- | --- | --- | --- | --- | --- | --- | --- | --- |
| q-value | 1 | 0.2 | 0.05 | 0.01 | 0.001 | 0.0001 |
| Color |  | |  |  |  | |

TABLE OF Q-VALUES

| aspergillus fumigatus conidia a549 | candida albicans moddc135 | Gene Set |
| --- | --- | --- |
| 0.014604702 | 3.2457785E-4 | NETPATH\_TGFBETA\_RECEPTOR\_PATHWAY\_UP |
| 0.060973283 | 0.0151228625 | SIGNAL\_TRANSDUCTION |
| 2.9144692E-4 | 0.0 | NETPATH\_IL\_2\_PATHWAY\_UP |
| 0.05298526 | 0.047334336 | NEGATIVE\_REGULATION\_OF\_BIOLOGICAL\_PROCESS |
| 1.5328516E-5 | 0.0 | NETPATH\_IL\_1\_PATHWAY\_UP |
| 6.960372E-4 | 2.43421E-4 | RESPONSE\_TO\_EXTERNAL\_STIMULUS |
| 0.052566186 | 0.04382298 | NEGATIVE\_REGULATION\_OF\_CELLULAR\_PROCESS |
| 1.0097658E-4 | 2.7117665E-6 | NETPATH\_IL\_5\_PATHWAY\_UP |
| 0.10950888 | 0.002677061 | REACTOME\_SIGNALING\_IN\_IMMUNE\_SYSTEM |
| 2.0300957E-5 | 2.7103227E-4 | NETPATH\_IL\_4\_PATHWAY\_UP |
| 0.07917507 | 5.556078E-6 | NEGATIVE\_REGULATION\_OF\_APOPTOSIS |
| 2.5672338E-5 | 0.0 | KEGG\_CYTOKINE\_CYTOKINE\_RECEPTOR\_INTERACTION |
| 0.075307034 | 3.9515476E-6 | NEGATIVE\_REGULATION\_OF\_PROGRAMMED\_CELL\_DEATH |
| 0.0013995847 | 2.1165008E-6 | RESPONSE\_TO\_WOUNDING |
| 0.004826704 | 0.016131835 | PROTEIN\_KINASE\_CASCADE |
| 0.0 | 2.3453117E-6 | NETPATH\_TNF\_ALPHA\_PATHWAY\_DOWN |
| 0.028497338 | 6.846009E-5 | KEGG\_CHEMOKINE\_SIGNALING\_PATHWAY |
| 0.070169486 | 2.2477773E-4 | NEGATIVE\_REGULATION\_OF\_DEVELOPMENTAL\_PROCESS |
| 0.09567344 | 2.410459E-6 | IMMUNE\_SYSTEM\_PROCESS |
| 0.003923119 | 0.005379269 | NCI\_REG\_GR\_PATHWAY |
| 8.290948E-4 | 0.0 | NETPATH\_IL\_4\_PATHWAY\_DOWN |
| 0.003725802 | 3.7335965E-5 | NETPATH\_KIT\_RECEPTOR\_PATHWAY\_UP |
| 7.929928E-5 | 2.5522509E-6 | KEGG\_NOD\_LIKE\_RECEPTOR\_SIGNALING\_PATHWAY |
| 1.4370484E-5 | 0.0 | NCI\_IL23PATHWAY |
| 0.07053614 | 0.0 | EXTRACELLULAR\_SPACE |
| 0.009436423 | 0.0074755205 | NCI\_LYSOPHOSPHOLIPID\_PATHWAY |
| 1.642341E-5 | 2.2250392E-6 | BEHAVIOR |
| 0.005676588 | 0.0030833506 | NETPATH\_B\_CELL\_RECEPTOR\_PATHWAY\_UP |
| 0.039666753 | 0.0 | IMMUNE\_RESPONSE |
| 2.2720385E-5 | 3.2139455E-6 | LOCOMOTORY\_BEHAVIOR |
| 2.5547526E-5 | 0.0 | NETPATH\_EGFR1\_PATHWAY\_UP |
| 0.0015525026 | 1.4066127E-4 | REACTOME\_CLASS\_A1\_RHODOPSIN\_LIKE\_RECEPTORS |
| 1.9160645E-5 | 0.0 | REACTOME\_PEPTIDE\_LIGAND\_BINDING\_RECEPTORS |
| 2.0902522E-5 | 0.0 | CYTOKINE\_ACTIVITY |
| 2.8400484E-5 | 0.0 | CHEMOKINE\_ACTIVITY |
| 0.005702261 | 2.1694132E-6 | DEFENSE\_RESPONSE |
| 0.0 | 0.0 | REACTOME\_CHEMOKINE\_RECEPTORS\_BIND\_CHEMOKINES |
| 1.4434879E-4 | 5.8961764E-4 | NCI\_NFAT\_TFPATHWAY |
| 0.0056939363 | 2.0661078E-6 | NCI\_AMB2\_NEUTROPHILS\_PATHWAY |
| 0.0076145586 | 0.002180355 | KEGG\_T\_CELL\_RECEPTOR\_SIGNALING\_PATHWAY |
| 0.024652105 | 0.0128200315 | NCI\_IL2\_1PATHWAY |
| 0.06493268 | 0.0016598669 | REACTOME\_GPCR\_LIGAND\_BINDING |
| 0.017372232 | 2.892551E-6 | KEGG\_TOLL\_LIKE\_RECEPTOR\_SIGNALING\_PATHWAY |
| 0.096030585 | 0.0 | RECEPTOR\_BINDING |
| 1.768675E-5 | 2.9922942E-6 | KEGG\_JAK\_STAT\_SIGNALING\_PATHWAY |
| 0.008573098 | 0.0070938244 | REACTOME\_TOLL\_LIKE\_RECEPTOR\_3\_CASCADE |
| 0.0 | 0.0 | CHEMOKINE\_RECEPTOR\_BINDING |
| 0.044559825 | 0.002267632 | KEGG\_EPITHELIAL\_CELL\_SIGNALING\_IN\_HELICOBACTER\_PYLORI\_INFECTION |
| 2.8740968E-5 | 0.0 | NETPATH\_IL\_3\_PATHWAY\_UP |
| 0.044578478 | 3.0991619E-6 | KEGG\_LEISHMANIA\_INFECTION |
| 3.6444217E-5 | 0.0 | NETPATH\_IL\_6\_PATHWAY\_UP |
| 1.8933655E-5 | 0.0 | G\_PROTEIN\_COUPLED\_RECEPTOR\_BINDING |
| 6.0851693E-5 | 4.0434443E-6 | INFLAMMATORY\_RESPONSE |
| 0.02162187 | 3.7597125E-5 | BIOCARTA\_STEM\_PATHWAY |
| 1.0158219E-4 | 0.002537575 | NETPATH\_IL\_9\_PATHWAY\_UP |
| 0.112507604 | 0.013837767 | NCI\_TNFPATHWAY |
| 0.070430465 | 2.7992428E-6 | BIOCARTA\_IL1R\_PATHWAY |
| 0.01466968 | 0.034454864 | NCI\_DISSOLUTION\_OF\_FIBRIN\_CLOT |
| 0.10805145 | 0.019178493 | VIRAL\_REPRODUCTION |
| 0.045313723 | 1.6053132E-4 | BIOCARTA\_IL17\_PATHWAY |
| 0.017489815 | 0.009618234 | REACTOME\_G\_ALPHA\_I\_SIGNALLING\_EVENTS |
| 0.0054974956 | 0.0 | NCI\_IL12\_2PATHWAY |
| 0.100959316 | 0.01161826 | VIRAL\_INFECTIOUS\_CYCLE |
| 0.003983041 | 0.004631088 | KEGG\_MAPK\_SIGNALING\_PATHWAY |
| 0.06791781 | 0.001417204 | VIRAL\_GENOME\_REPLICATION |
| 0.007446698 | 0.0 | BIOCARTA\_CYTOKINE\_PATHWAY |
| 0.017831605 | 8.434533E-4 | BIOCARTA\_NTHI\_PATHWAY |
| 0.03835076 | 0.004624408 | JAK\_STAT\_CASCADE |
| 0.008140157 | 0.011636542 | REACTOME\_TRAF6\_MEDIATED\_INDUCTION\_OF\_THE\_ANTIVIRAL\_CYTOKINE\_IFN\_ALPHA\_BETA\_CASCADE |
| 0.08271712 | 1.6542599E-5 | KEGG\_RIG\_I\_LIKE\_RECEPTOR\_SIGNALING\_PATHWAY |
| 0.11097098 | 0.033170246 | REGULATION\_OF\_RESPONSE\_TO\_STIMULUS |
| 0.01884623 | 0.0 | BIOCARTA\_INFLAM\_PATHWAY |
| 0.04560868 | 0.029836837 | BIOCARTA\_GRANULOCYTES\_PATHWAY |
| 0.07733991 | 0.01410618 | BIOCARTA\_NFKB\_PATHWAY |
| 0.10709039 | 0.003457766 | POSITIVE\_REGULATION\_OF\_CELLULAR\_PROTEIN\_METABOLIC\_PROCESS |
| 3.284682E-5 | 0.0 | NETPATH\_IL\_7\_PATHWAY\_UP |
| 0.09501592 | 0.011223838 | ST\_ERK1\_ERK2\_MAPK\_PATHWAY |
| 0.019903738 | 0.009154281 | BIOCARTA\_CD40\_PATHWAY |
| 0.09741977 | 3.5301655E-5 | BIOCARTA\_ERYTH\_PATHWAY |
| 0.009898863 | 3.2052063E-4 | NCI\_CHEMOKINE\_RECEPTORS\_BIND\_CHEMOKINES |
| 0.09243208 | 4.633083E-4 | REGULATION\_OF\_IMMUNE\_SYSTEM\_PROCESS |
| 0.006852322 | 0.044520505 | NETPATH\_HEDGEHOG\_PATHWAY\_UP |
| 0.039703723 | 0.008807747 | POSITIVE\_REGULATION\_OF\_IMMUNE\_SYSTEM\_PROCESS |
| 0.012665072 | 0.0036709863 | BIOCARTA\_CARDIACEGF\_PATHWAY |
| 0.04039933 | 0.006842382 | PEPTIDYL\_TYROSINE\_MODIFICATION |
| 0.0031213323 | 0.0074842023 | NCI\_TCRCALCIUMPATHWAY |
| 2.2992774E-5 | 5.803015E-6 | NCI\_ATF2\_PATHWAY |
| 0.05835833 | 0.0025017355 | NETPATH\_EGFR1\_PATHWAY\_DOWN |
| 0.019676251 | 0.026470594 | BIOCARTA\_CCR5\_PATHWAY |
| 0.050761063 | 0.0035081438 | BIOCARTA\_CDMAC\_PATHWAY |
| 0.01756342 | 0.001492786 | REACTOME\_CD28\_DEPENDENT\_PI3K\_AKT\_SIGNALING |
| 0.02733804 | 3.231262E-4 | CORUM\_CHUK-NFKB2-REL-IKBKG-SPAG9-NFKB1-NFKBIE-COPB2-TNIP1-NFKBIA-RELA-TNIP2\_COMPLEX |
| 0.037143324 | 0.0138140945 | POSITIVE\_REGULATION\_OF\_MULTICELLULAR\_ORGANISMAL\_PROCESS |
| 0.0517779 | 0.0033218658 | PEPTIDYL\_TYROSINE\_PHOSPHORYLATION |
| 0.030143488 | 0.0127873905 | POSITIVE\_REGULATION\_OF\_IMMUNE\_RESPONSE |
| 3.4622008E-5 | 1.14572584E-4 | NCI\_CD40\_PATHWAY |
| 0.02029611 | 0.00871612 | POSITIVE\_REGULATION\_OF\_TRANSLATION |
| 0.013679031 | 0.022878889 | REGULATION\_OF\_IMMUNE\_RESPONSE |
| 0.08752097 | 4.1946248E-4 | POSITIVE\_REGULATION\_OF\_CELL\_PROLIFERATION |
| 0.031115418 | 9.770222E-4 | BIOCARTA\_KERATINOCYTE\_PATHWAY |
| 0.011185336 | 9.2645256E-5 | BIOCARTA\_TNFR2\_PATHWAY |
| 0.02079193 | 0.04540201 | NCI\_NFKAPPABCANONICALPATHWAY |
| 0.142593 | 0.025013098 | POSITIVE\_REGULATION\_OF\_BIOLOGICAL\_PROCESS |
| 0.12753585 | 0.029801909 | LEUKOCYTE\_ACTIVATION |
| 0.17969474 | 5.2014195E-5 | NETPATH\_IL\_1\_PATHWAY |
| 0.13260934 | 4.004787E-5 | EXTRACELLULAR\_REGION\_PART |
| 0.14679587 | 0.01380212 | REACTOME\_AMINO\_ACID\_AND\_OLIGOPEPTIDE\_SLC\_TRANSPORTERS |
| 0.017533444 | 0.104172826 | NCI\_FGF\_PATHWAY |
| 0.1734655 | 0.014559986 | KEGG\_HYPERTROPHIC\_CARDIOMYOPATHY\_HCM |
| 0.12003832 | 0.15405947 | NETPATH\_T\_CELL\_RECEPTOR\_PATHWAY\_DOWN |
| 0.17762682 | 0.013494399 | NCI\_IL2\_STAT5PATHWAY |
| 0.11278513 | 2.6295918E-6 | BIOCARTA\_NKT\_PATHWAY |
| 0.059626676 | 0.12882927 | I\_KAPPAB\_KINASE\_NF\_KAPPAB\_CASCADE |
| 0.118695535 | 0.0023013013 | BIOCARTA\_LAIR\_PATHWAY |
| 0.12671489 | 0.009630525 | NCI\_IL2\_PI3KPATHWAY |
| 0.027411686 | 0.098345995 | KEGG\_FOCAL\_ADHESION |
| 0.092734 | 0.17607056 | REGULATION\_OF\_I\_KAPPAB\_KINASE\_NF\_KAPPAB\_CASCADE |
| 0.07277688 | 0.035474196 | REGULATION\_OF\_PROTEIN\_KINASE\_ACTIVITY |
| 0.0037352245 | 0.13496065 | NCI\_HIF1\_TFPATHWAY |
| 0.1438923 | 0.15738921 | CORUM\_AKAP250-PKA-PDE4D\_COMPLEX |
| 0.12109292 | 0.033600047 | BIOCARTA\_LYM\_PATHWAY |
| 0.15894607 | 0.0034152507 | POSITIVE\_REGULATION\_OF\_PROTEIN\_METABOLIC\_PROCESS |
| 0.017768223 | 0.119654395 | ST\_GRANULE\_CELL\_SURVIVAL\_PATHWAY |
| 0.048240278 | 0.06924841 | POSITIVE\_REGULATION\_OF\_METABOLIC\_PROCESS |
| 0.01471888 | 0.06268975 | POSITIVE\_REGULATION\_OF\_RESPONSE\_TO\_STIMULUS |
| 0.038137298 | 0.002260311 | ADAPTIVE\_IMMUNE\_RESPONSE\_GO\_0002460 |
| 0.123622485 | 0.06369417 | BIOCARTA\_ETS\_PATHWAY |
| 0.0870416 | 0.044430036 | REGULATION\_OF\_TRANSFERASE\_ACTIVITY |
| 0.12842566 | 0.054095533 | BIOCARTA\_WNT\_PATHWAY |
| 0.00397121 | 0.080644235 | BIOCARTA\_RANKL\_PATHWAY |
| 0.12902527 | 4.9217895E-4 | CELL\_CELL\_SIGNALING |
| 0.19505428 | 0.075348035 | T\_CELL\_DIFFERENTIATION |
| 0.109485276 | 0.08797927 | NETPATH\_TNF\_ALPHA\_PATHWAY |
| 0.012436739 | 0.078599736 | REACTOME\_MAP\_KINASES\_ACTIVATION\_IN\_TLR\_CASCADE |
| 0.08275426 | 0.008728356 | REGULATION\_OF\_PEPTIDYL\_TYROSINE\_PHOSPHORYLATION |
| 0.19079636 | 0.07530166 | INNATE\_IMMUNE\_RESPONSE |
| 0.03207342 | 2.466239E-4 | CORUM\_TNF-ALPHA/NF-KAPPA\_B\_SIGNALING\_COMPLEX\_CHUK\_KPNA3\_NFKB2\_NFKBIB\_REL\_IKBKG\_\_NFKB1\_NFKBIE\_RELB\_\_NFKBIA\_RELA\_TNIP2 |
| 0.07091445 | 0.04790732 | POSITIVE\_REGULATION\_OF\_ANGIOGENESIS |
| 0.020051014 | 0.13157527 | CALCIUM\_MEDIATED\_SIGNALING |
| 0.029508216 | 0.19104342 | BIOCARTA\_HIVNEF\_PATHWAY |
| 0.10401403 | 0.12749368 | NCI\_P38ALPHABETAPATHWAY |
| 0.009731392 | 2.160868E-5 | NCI\_CD8TCRDOWNSTREAMPATHWAY |
| 0.05171527 | 0.052799206 | NETPATH\_TGFBETA\_RECEPTOR\_PATHWAY\_DOWN |
| 0.10655671 | 0.14632621 | POSITIVE\_REGULATION\_OF\_CELL\_MIGRATION |
| 0.14658783 | 0.08456892 | BIOCARTA\_41BB\_PATHWAY |
| 0.02807921 | 0.11711622 | NEGATIVE\_REGULATION\_OF\_TRANSFERASE\_ACTIVITY |
| 0.02839442 | 0.12501344 | NCI\_P53DOWNSTREAMPATHWAY |
| 0.07160611 | 2.3260359E-4 | CORUM\_TNF-ALPHA/NF-KAPPA\_B\_SIGNALING\_COMPLEX\_RPL6\_RPL30\_RPS13\_CHUK\_DDX3X\_NFKB2\_NFKBIB\_REL\_IKBKG\_NFKB1\_MAP3K8\_RELB\_GLG1\_NFKBIA\_RELA\_TNIP2\_\_GTF2I |
| 0.004500425 | 0.07048068 | REACTOME\_CD28\_CO\_STIMULATION |
| 0.07182297 | 0.19270521 | NETPATH\_IL\_2\_PATHWAY |
| 0.0033291676 | 0.055568002 | ST\_TUMOR\_NECROSIS\_FACTOR\_PATHWAY |
| 0.19046405 | 0.065832324 | NCI\_HIF2PATHWAY |
| 0.18954149 | 0.18433525 | ANATOMICAL\_STRUCTURE\_DEVELOPMENT |
| 0.09540232 | 0.082406156 | POSITIVE\_REGULATION\_OF\_SIGNAL\_TRANSDUCTION |
| 0.030544922 | 0.0031446011 | NEGATIVE\_REGULATION\_OF\_MAP\_KINASE\_ACTIVITY |
| 0.17585014 | 0.019340528 | CORUM\_NFKB1-NFKB2-REL-RELA-RELB\_COMPLEX |
| 0.19764018 | 0.030061496 | POSITIVE\_REGULATION\_OF\_CELLULAR\_PROCESS |
| 0.049802355 | 0.13080896 | POSITIVE\_REGULATION\_OF\_PHOSPHATE\_METABOLIC\_PROCESS |
| 0.14483164 | 0.18730994 | REGULATION\_OF\_CATALYTIC\_ACTIVITY |
| 0.11872337 | 0.034114335 | VIRAL\_REPRODUCTIVE\_PROCESS |
| 0.08485524 | 0.1729668 | POSITIVE\_REGULATION\_OF\_CELL\_DIFFERENTIATION |
| 0.030330766 | 0.0032938984 | INACTIVATION\_OF\_MAPK\_ACTIVITY |
| 0.14630988 | 0.15386285 | NCI\_EPHRINBREVPATHWAY |
| 0.07534794 | 0.03825301 | REGULATION\_OF\_KINASE\_ACTIVITY |
| 0.106404856 | 0.0648679 | NCI\_NFKAPPABATYPICALPATHWAY |
| 0.03677448 | 0.057645433 | REACTOME\_DOWNSTREAM\_EVENTS\_IN\_GPCR\_SIGNALING |
| 0.1286303 | 0.09919815 | BIOCARTA\_TALL1\_PATHWAY |
| 0.15138073 | 0.011160989 | NETPATH\_IL\_7\_PATHWAY |
| 0.026384508 | 0.001323389 | ADAPTIVE\_IMMUNE\_RESPONSE |
| 0.16804174 | 2.3349554E-4 | ANTI\_APOPTOSIS |
| 0.02137055 | 0.059259217 | KEGG\_SMALL\_CELL\_LUNG\_CANCER |
| 0.039465003 | 0.090868875 | POSITIVE\_REGULATION\_OF\_PHOSPHORYLATION |
| 0.19973694 | 0.01743104 | REACTOME\_AMINO\_ACID\_TRANSPORT\_ACROSS\_THE\_PLASMA\_MEMBRANE |
| 0.15891588 | 0.088299386 | NCI\_REGULATED\_PROTEOLYSIS\_OF\_P75NTR |
| 0.15445554 | 0.054322407 | BIOCARTA\_PPARA\_PATHWAY |
| 0.11095213 | 0.14472564 | REGULATION\_OF\_CYTOKINE\_PRODUCTION |
| 0.039691873 | 0.11765984 | MAPKKK\_CASCADE\_GO\_0000165 |
| 0.19023821 | 0.10234088 | ORGAN\_DEVELOPMENT |
| 0.058202147 | 0.07307491 | POSITIVE\_REGULATION\_OF\_CELLULAR\_METABOLIC\_PROCESS |
| 0.04541114 | 0.09532635 | INTRACELLULAR\_SIGNALING\_CASCADE |
| 0.042247143 | 0.12340284 | SIG\_CD40PATHWAYMAP |
| 0.11829043 | 0.033861402 | REGULATION\_OF\_CELL\_ADHESION |
| 0.1594092 | 0.06459408 | BIOCARTA\_RNA\_PATHWAY |
| 0.13933784 | 0.06699987 | ST\_FAS\_SIGNALING\_PATHWAY |
| 0.045901977 | 0.15677607 | CELL\_SURFACE\_RECEPTOR\_LINKED\_SIGNAL\_TRANSDUCTION\_GO\_0007166 |
| 0.13951814 | 0.18593393 | BIOCARTA\_DEATH\_PATHWAY |
| 0.038373187 | 0.16647865 | REACTOME\_DOWNSTREAM\_TCR\_SIGNALING |
| 0.08367486 | 0.063093565 | BIOCARTA\_STRESS\_PATHWAY |
| 0.19319388 | 0.14259836 | REACTOME\_SLC\_MEDIATED\_TRANSMEMBRANE\_TRANSPORT |
| 0.06895393 | 0.03988219 | KEGG\_GAP\_JUNCTION |
| 0.014696792 | 0.15870087 | PROTEIN\_KINASE\_ACTIVITY |
| 0.11586409 | 0.008753433 | REGULATION\_OF\_MULTICELLULAR\_ORGANISMAL\_PROCESS |
| 0.015543423 | 0.17623867 | REACTOME\_TOLL\_RECEPTOR\_CASCADES |
| 0.14205706 | 0.0 | KEGG\_HEMATOPOIETIC\_CELL\_LINEAGE |
| 0.15459792 | 0.039409634 | REACTOME\_P75NTR\_SIGNALS\_VIA\_NFKB |
| 0.039878834 | 0.1863261 | NCI\_CD28\_DEPENDENT\_PI3K\_AKT\_SIGNALING |
| 0.0014031419 | 0.06264309 | NCI\_IL12\_STAT4PATHWAY |
| 0.07208636 | 0.17238665 | ORGAN\_MORPHOGENESIS |
| 0.09992959 | 0.17945796 | BIOCARTA\_TOLL\_PATHWAY |
| 0.01780716 | 0.18703136 | REACTOME\_MAPK\_**KNOWN DRUG TARGETS**\_NUCLEAR\_EVENTS\_MEDIATED\_BY\_MAP\_KINASES |
| 0.040118776 | 0.1862735 | REGULATION\_OF\_MAP\_KINASE\_ACTIVITY |
| 0.021361278 | 0.094901785 | KEGG\_PATHWAYS\_IN\_CANCER |
| 6.440544E-4 | 0.066257045 | NETPATH\_WNT\_PATHWAY\_UP |
| 0.07025918 | 0.04753958 | BIOCARTA\_ARENRF2\_PATHWAY |
