# Supplementary material for: Computational approaches for discovery of common immunomodulators in fungal infections: towards broad-spectrum immunotherapeutic interventions
Source: BMC Microbiol. 2013 Oct 7;13:224. doi: 10.1186/1471-2180-13-224 (PMC3853472; doi:10.1186/1471-2180-13-224)
Supplement: Additional file 1 — Details of up- and down- regulated biclusters. [file 1471-2180-13-224-S1.zip › 2013-kidane-bmc/details-of-biclusters/upreg-biclust-26.html]

**BICLUSTER\_ID** : UPREG-26  
**PATHOGENS** /2/ : a. fumigatus,c. albicans  
**KNOWN DRUG TARGETS** /16/ : THBS1, CCL20, SERPINB2, GPR109B, PRNP, CCL5, PIM1, ADORA2A, PTGER4, NP, IL1B, PTGS2, ICAM1, IL8, PTGER2, TNF  

| Gene Set | Leading Edge Genes |
| --- | --- |
| NETPATH IL 1 PATHWAY UP | NR4A3, CCL20, SERPINB2, FOSL1, CXCL3, PRNP, NFKBIA, CXCL2, CCL4, PTX3, CXCL1, LIF, NP, CKS2, IL1B, PTGS2, ICAM1, IL8 |
| REACTOME CLASS A1 RHODOPSIN LIKE RECEPTORS | PTGER4, CCL20, CXCL1, C5AR1, EDN1, GPR109B, CXCL3, CCL5, IL8, PTGER2, CXCL2, CCL4, ADORA2A |
| NETPATH IL 3 PATHWAY UP | CSF1, USP36, SOCS3, TGFA, IL1B, OSM, PIM1, IL8, TNF, CCL4 |
| CYTOKINE ACTIVITY | CCL20, CXCL1, CSF1, CXCL3, IL1RN, CCL5, OSM, CXCL2, IL8, CSF2, TNF, CCL4 |
| CHEMOKINE ACTIVITY | CXCL3, CCL20, CXCL1, CCL5, IL8, CXCL2, CCL4 |
| NETPATH TNF ALPHA PATHWAY DOWN | THBS1, CCL20, CXCL3, NFKBIA, EGR1, CXCL2, IER3, REL, INSIG1, KLF6, CXCL1, PPP1R15A, KLF10, FOSB, MAPK6, EGR3, DDX3X, MAFF, DUSP2, PTGS2, TNFAIP3 |
| NCI NFAT TFPATHWAY | EGR3, FOSL1, EGR1, PTGS2, IL8, FOS, TNF |
| G PROTEIN COUPLED RECEPTOR BINDING | CXCL3, CCL20, CXCL1, CCL5, IL8, CXCL2, CCL4 |
| REACTOME PEPTIDE LIGAND BINDING RECEPTORS | CCL20, CXCL1, C5AR1, EDN1, CXCL3, CCL5, IL8, CXCL2, CCL4 |
| CHEMOKINE RECEPTOR BINDING | CXCL3, CCL20, CXCL1, CCL5, IL8, CXCL2, CCL4 |
| REACTOME CHEMOKINE RECEPTORS BIND CHEMOKINES | CXCL3, CCL20, CXCL1, CCL5, IL8, CXCL2, CCL4 |
| NETPATH IL 7 PATHWAY UP | CXCL1, TFRC, BCL2L1, TNFSF8, CXCL3, CCL5, IL8, CXCL2, CCL4 |
| NETPATH WNT PATHWAY DOWN | IL1B, CXCL1 |
| BIOCARTA STEM PATHWAY | CSF1, IL8, CSF2 |
| BIOCARTA FIBRINOLYSIS PATHWAY | SERPINB2 |
| REACTOME UNFOLDED PROTEIN RESPONSE | ERN1, DDIT3, DNAJB9, EIF2AK3, HERPUD1, EDEM1, ATF3, XBP1 |
| HORMONE ACTIVITY | EDN1 |
| NEGATIVE REGULATION OF CELLULAR BIOSYNTHETIC PROCESS |  |
| HORMONE SECRETION | LIF, OSM |
| GENERATION OF A SIGNAL INVOLVED IN CELL CELL SIGNALING |  |
| BIOCARTA PLATELETAPP PATHWAY |  |
| GROWTH FACTOR ACTIVITY | TGFA, IL1RN, CSF1, CSF2 |
| HEMATOPOIETIN INTERFERON CLASSD200 DOMAIN CYTOKINE RECEPTOR BINDING | OSM, CSF2 |
| REGULATION OF HORMONE SECRETION | LIF, OSM |
| NETPATH WNT PATHWAY UP |  |
| REACTOME STEROID HORMONES |  |

| Color legend | | | | | | | | | | | |
| --- | --- | --- | --- | --- | --- | --- | --- | --- | --- | --- | --- |
| q-value | 1 | 0.2 | 0.05 | 0.01 | 0.001 | 0.0001 |
| Color |  | |  |  |  | |

TABLE OF Q-VALUES

| candida albicans moddc135 | candida albicans neutrophils | aspergillus fumigatus dendritic | Gene Set |
| --- | --- | --- | --- |
| 0.0 | 0.19693953 | 0.0 | NETPATH\_IL\_1\_PATHWAY\_UP |
| 1.4066127E-4 | 0.17791544 | 0.003579989 | REACTOME\_CLASS\_A1\_RHODOPSIN\_LIKE\_RECEPTORS |
| 0.0 | 0.10933371 | 5.743034E-5 | NETPATH\_IL\_3\_PATHWAY\_UP |
| 0.0 | 7.5262925E-4 | 0.0 | CYTOKINE\_ACTIVITY |
| 0.0 | 0.091335885 | 3.546737E-5 | CHEMOKINE\_ACTIVITY |
| 2.3453117E-6 | 0.10874915 | 0.0036943294 | NETPATH\_TNF\_ALPHA\_PATHWAY\_DOWN |
| 5.8961764E-4 | 0.035003204 | 1.962291E-4 | NCI\_NFAT\_TFPATHWAY |
| 0.0 | 0.1173318 | 6.563468E-5 | G\_PROTEIN\_COUPLED\_RECEPTOR\_BINDING |
| 0.0 | 0.10786476 | 0.0 | REACTOME\_PEPTIDE\_LIGAND\_BINDING\_RECEPTORS |
| 0.0 | 0.08899127 | 2.8777304E-5 | CHEMOKINE\_RECEPTOR\_BINDING |
| 0.0 | 0.17955464 | 0.0 | REACTOME\_CHEMOKINE\_RECEPTORS\_BIND\_CHEMOKINES |
| 0.0 | 0.061718952 | 0.0 | NETPATH\_IL\_7\_PATHWAY\_UP |
| 0.002521776 | 0.053037744 | 0.15058221 | NETPATH\_WNT\_PATHWAY\_DOWN |
| 3.7597125E-5 | 0.18127525 | 0.016043266 | BIOCARTA\_STEM\_PATHWAY |
| 0.009271151 | 0.17570677 | 0.07972296 | BIOCARTA\_FIBRINOLYSIS\_PATHWAY |
| 0.013748358 | 0.13336742 | 0.025169797 | REACTOME\_UNFOLDED\_PROTEIN\_RESPONSE |
| 0.011306912 | 0.0028156147 | 7.06835E-5 | HORMONE\_ACTIVITY |
| 0.06449941 | 0.025412852 | 0.01092913 | NEGATIVE\_REGULATION\_OF\_CELLULAR\_BIOSYNTHETIC\_PROCESS |
| 0.013864454 | 0.064871006 | 0.0033732688 | HORMONE\_SECRETION |
| 0.095168166 | 7.8165246E-4 | 0.0021141812 | GENERATION\_OF\_A\_SIGNAL\_INVOLVED\_IN\_CELL\_CELL\_SIGNALING |
| 0.098546006 | 0.1780624 | 0.13522168 | BIOCARTA\_PLATELETAPP\_PATHWAY |
| 0.0 | 0.02203862 | 5.1121856E-4 | GROWTH\_FACTOR\_ACTIVITY |
| 8.233354E-5 | 0.037406106 | 1.621935E-5 | HEMATOPOIETIN\_INTERFERON\_CLASSD200\_DOMAIN\_CYTOKINE\_RECEPTOR\_BINDING |
| 0.020191431 | 0.10907822 | 0.009836641 | REGULATION\_OF\_HORMONE\_SECRETION |
| 0.066257045 | 0.18667124 | 0.16470632 | NETPATH\_WNT\_PATHWAY\_UP |
| 0.15807852 | 0.09484979 | 0.020148115 | REACTOME\_STEROID\_HORMONES |
