# Supplementary material for: Computational approaches for discovery of common immunomodulators in fungal infections: towards broad-spectrum immunotherapeutic interventions
Source: BMC Microbiol. 2013 Oct 7;13:224. doi: 10.1186/1471-2180-13-224 (PMC3853472; doi:10.1186/1471-2180-13-224)
Supplement: Additional file 1 — Details of up- and down- regulated biclusters. [file 1471-2180-13-224-S1.zip › 2013-kidane-bmc/details-of-biclusters/upreg-biclust-28.html]

**BICLUSTER\_ID** : UPREG-28  
**PATHOGENS** /2/ : c. albicans,a. fumigatus  
**KNOWN DRUG TARGETS** /7/ : FYN, CCL20, PTGS2, JUN, PIM1, IL8, PLAUR  

| Gene Set | Leading Edge Genes |
| --- | --- |
| NETPATH IL 1 PATHWAY UP | NR4A3, CCL20, LIF, JUN, CXCL1, DUSP3, NFKBIE, FOSL1, JMJD3, CXCL3, NFKBIA, PTGS2, ZFP36, IL8, CXCL2 |
| NETPATH TNF ALPHA PATHWAY DOWN | CCL20, JUN, CXCL3, NFKBIA, ZNF131, EGR1, FOSL2, CXCL2, BHLHB2, IER3, KLF6, REL, NR4A1, CXCL1, KLF10, PPP1R15A, JUNB, FOSB, MAPK6, VEGF, DDX3X, GTF2H1, MAFF, PTGS2, TNFAIP3, ZFP36 |
| NCI NFAT TFPATHWAY | FOSL1, EGR1, PTGS2, JUN, JUNB, IL8, FOS |
| NETPATH WNT PATHWAY UP | JUN, SIAH2, KLF10, MAPK6, FOS, IL8, CREM, DUSP1, PLAUR |
| NETPATH IL 3 PATHWAY UP | NFIL3, PIM1, IL8, FOS |
| REACTOME CLASS A1 RHODOPSIN LIKE RECEPTORS | CXCL3, CCL20, CXCL1, EDN1, CXCL2, IL8 |
| REACTOME PEPTIDE LIGAND BINDING RECEPTORS | CXCL3, CCL20, CXCL1, EDN1, CXCL2, IL8 |
| CYTOKINE ACTIVITY | CXCL3, CCL20, CXCL1, IL8, CXCL2 |
| NETPATH IL 7 PATHWAY UP | CXCL3, KLF2, CXCL1, JUN, IL8, CXCL2 |
| CHEMOKINE ACTIVITY | CXCL3, CCL20, CXCL1, CXCL2, IL8 |
| G PROTEIN COUPLED RECEPTOR BINDING | CXCL3, CCL20, CXCL1, CXCL2, IL8 |
| CHEMOKINE RECEPTOR BINDING | CXCL3, CCL20, CXCL1, CXCL2, IL8 |
| REACTOME CHEMOKINE RECEPTORS BIND CHEMOKINES | CXCL3, CCL20, CXCL1, CXCL2, IL8 |
| BIOCARTA STEM PATHWAY | IL8 |
| FEEDING BEHAVIOR | FYN |
| STRUCTURAL CONSTITUENT OF RIBOSOME | RPL27, RPL24, RPL38, RPS17, RPS16, RPS28, RPL18, RPL22, RPS8, RPL29, RPL13A, RPS20, RPS15A, RPS18, RPLP1, RPS10, RPS14, RPS5, RPS21, RPL13, RPS12, RPL30, RPS29, RPL19, RPL9, RPS13, RPL8, RPLP2, RPL31, RPS27, RPS24, RPS15 |
| NCI FORMATION OF A POOL OF FREE 40S SUBUNITS | RPL27, RPL24, RPL38, RPS17, RPS16, RPS28, RPL18, RPL22, RPL12, UBA52, RPS8, RPL13A, RPL29, RPS20, RPL36, RPL10A, RPS15A, RPS18, RPLP1, RPS10, RPS14, RPS5, RPS21, RPL13, RPS12, RPL30, RPS29, RPL19, RPL9, RPS13, RPL8, RPLP2, RPL35A, RPL31, RPS24, RPS27, RPS15 |
| REACTOME PEPTIDE CHAIN ELONGATION | RPL27, RPL24, RPL38, RPS17, RPS16, RPS28, RPL18, RPL22, RPL12, UBA52, RPS8, RPL13A, RPL29, RPS20, RPL36, RPL10A, RPS15A, RPS18, RPLP1, RPS10, RPS14, RPS5, RPS21, RPL13, RPS12, RPL30, RPS29, RPL19, RPL9, RPS13, RPL8, RPLP2, RPL35A, RPL31, RPS24, RPS27, RPS15 |
| NCI VIRAL MRNA TRANSLATION | RPL27, RPL24, RPL38, RPS17, RPS16, RPS28, RPL18, RPL22, RPL12, UBA52, RPS8, RPL13A, RPL29, RPS20, RPL36, RPL10A, RPS15A, RPS18, RPLP1, RPS10, RPS14, RPS5, RPS21, RPL13, RPS12, RPL30, RPS29, RPL19, RPL9, RPS13, RPL8, RPLP2, RPL35A, RPL31, RPS24, RPS27, RPS15 |
| REACTOME VIRAL MRNA TRANSLATION | RPL27, RPL24, RPL38, RPS17, RPS16, RPS28, RPL18, RPL22, RPL12, UBA52, RPS8, RPL13A, RPL29, RPS20, RPL36, RPL10A, RPS15A, RPS18, RPLP1, RPS10, RPS14, RPS5, RPS21, RPL13, RPS12, RPL30, RPS29, RPL19, RPL9, RPS13, RPL8, RPLP2, RPL35A, RPL31, RPS24, RPS27, RPS15 |
| DEVELOPMENTAL MATURATION | EREG |
| REACTOME REGULATION OF GENE EXPRESSION IN BETA CELLS | RPL27, RPL24, RPL38, RPS17, RPS16, RPS28, RPL18, RPL22, RPL12, UBA52, RPS8, RPL29, RPL13A, RPS20, RPL36, RPL10A, RPS15A, RPS18, RPLP1, RPS10, RPS14, RPS5, RPS21, RPL13, RPS12, RPL30, RPS29, RPL19, RPL9, RPS13, RPL8, RPLP2, RPL35A, RPL31, RPS24, RPS27, RPS15 |
| NCI GTP HYDROLYSIS AND JOINING OF THE 60S RIBOSOMAL SUBUNIT | RPL27, RPL24, RPL38, RPS17, RPS16, RPS28, RPL18, RPL22, RPL12, UBA52, RPS8, RPL13A, RPL29, RPS20, RPL36, RPL10A, RPS15A, RPS18, RPLP1, RPS10, RPS14, RPS5, RPS21, RPL13, RPS12, RPL30, RPS29, RPL19, RPL9, RPS13, RPL8, RPLP2, RPL35A, RPL31, RPS24, RPS27, RPS15 |
| NCI CAP DEPENDENT TRANSLATION INITIATION | RPL27, RPL24, RPL38, RPS17, RPS16, RPS28, RPL18, RPL22, RPL12, UBA52, RPS8, RPL13A, RPL29, RPS20, RPL36, RPL10A, RPS15A, RPS18, RPLP1, RPS10, RPS14, RPS5, RPS21, RPL13, RPS12, RPL30, RPS29, RPL19, RPL9, RPS13, RPL8, RPLP2, RPL35A, RPL31, RPS24, RPS27, RPS15 |
| NCI EUKARYOTIC TRANSLATION TERMINATION | RPL27, RPL24, RPL38, RPS17, RPS16, RPS28, RPL18, RPL22, RPL12, UBA52, RPS8, RPL13A, RPL29, RPS20, RPL36, RPL10A, RPS15A, RPS18, RPLP1, RPS10, RPS14, RPS5, RPS21, RPL13, RPS12, RPL30, RPS29, RPL19, RPL9, RPS13, RPL8, RPLP2, RPL35A, RPL31, RPS24, RPS27, RPS15 |
| NCI PEPTIDE CHAIN ELONGATION | RPL27, RPL24, RPL38, RPS17, RPS16, RPS28, RPL18, RPL22, RPL12, UBA52, RPS8, RPL13A, RPL29, RPS20, RPL36, RPL10A, RPS15A, RPS18, RPLP1, RPS10, RPS14, RPS5, RPS21, RPL13, RPS12, RPL30, RPS29, RPL19, RPL9, RPS13, RPL8, RPLP2, RPL35A, RPL31, RPS24, RPS27, RPS15 |
| KEGG RIBOSOME | RPL27, RPL24, RPL38, RPS17, RPS16, RPS28, RPL18, RPL22, RPL12, UBA52, RPS8, RPL29, RPS20, RPL36, RPL10A, RPS15A, RPS18, RPLP1, RPS10, RPS5, RPS21, RPL13, RPS12, RPL30, RPS29, RPL19, RPL8, RPLP2, RPL31, RPL35A, RPS27, RPS24, RPS15 |
| REACTOME GTP HYDROLYSIS AND JOINING OF THE 60S RIBOSOMAL SUBUNIT | RPL27, RPL24, RPL38, RPS17, RPS16, RPS28, RPL18, RPL22, RPL12, UBA52, RPS8, RPL13A, RPL29, RPS20, RPL36, RPL10A, RPS15A, RPS18, RPLP1, RPS10, RPS14, RPS5, RPS21, RPL13, RPS12, RPL30, RPS29, RPL19, RPL9, RPS13, RPL8, RPLP2, RPL35A, RPL31, RPS24, RPS27, RPS15 |
| REACTOME REGULATION OF BETA CELL DEVELOPMENT | RPL27, RPL24, RPL38, RPS17, RPS16, RPS28, RPL18, RPL22, RPL12, UBA52, RPS8, RPL29, RPL13A, RPS20, RPL36, RPL10A, RPS15A, RPS18, RPLP1, RPS10, RPS14, RPS5, RPS21, RPL13, RPS12, RPL30, RPS29, RPL19, RPL9, RPS13, RPL8, RPLP2, RPL35A, RPL31, RPS24, RPS27, RPS15 |
| REACTOME INFLUENZA VIRAL RNA TRANSCRIPTION AND REPLICATION | RPL27, RPL24, RPL38, RPS17, RPS16, RPS28, RPL18, RPL22, RPL12, UBA52, RPS8, RPL29, RPL13A, RPS20, RPL36, RPL10A, RPS15A, RPS18, RPLP1, RPS10, RPS14, RPS5, RPS21, RPL13, RPS12, RPL30, POLR2F, RPS29, RPS13, RPL19, RPL9, RPL8, RPLP2, RPL35A, RPL31, RPS24, RPS27, RPS15 |
| REACTOME FORMATION OF A POOL OF FREE 40S SUBUNITS | RPL27, RPL24, RPL38, RPS17, RPS16, RPS28, RPL18, RPL22, RPL12, UBA52, RPS8, RPL13A, RPL29, RPS20, RPL36, RPL10A, RPS15A, RPS18, RPLP1, RPS10, RPS14, RPS5, RPS21, RPL13, RPS12, RPL30, RPS29, RPL19, RPL9, RPS13, RPL8, RPLP2, RPL35A, RPL31, RPS24, RPS27, RPS15 |
| REACTOME TRANSLATION | RPL27, EEF1B2, RPL24, RPL38, RPS17, RPS16, RPS28, RPL18, RPL22, RPL12, UBA52, RPS8, RPL29, RPL13A, RPL10A, RPS20, RPL36, RPS15A, RPS18, RPLP1, RPS10, RPS14, RPS5, RPS21, RPL13, RPS12, RPL30, RPS29, RPL19, RPL9, RPS13, RPL8, RPLP2, RPL35A, RPL31, RPS24, RPS27, RPS15 |
| CORUM RIBOSOME CYTOPLASMIC | RPL27, RPL24, RPL38, RPS17, RPS16, RPS28, RPL18, RPL22, RPL12, RPS8, RPL13A, RPL29, RPS20, RPL36, RPL10A, RPS15A, RPS18, RPLP1, RPS10, RPS14, RPS5, RPS21, RPL13, RPS12, RPL30, RPS29, RPL19, RPL9, RPS13, RPL8, RPLP2, RPL35A, RPL31, RPS24, RPS27, RPS15 |
| NCI L13A MEDIATED TRANSLATIONAL SILENCING OF CERULOPLASMIN EXPRESSION | RPL27, RPL24, RPL38, RPS17, RPS16, RPS28, RPL18, RPL22, RPL12, UBA52, RPS8, RPL13A, RPL29, RPS20, RPL36, RPL10A, RPS15A, RPS18, RPLP1, RPS10, RPS14, RPS5, RPS21, RPL13, RPS12, RPL30, RPS29, RPL19, RPL9, RPS13, RPL8, RPLP2, RPL35A, RPL31, RPS24, RPS27, RPS15 |

| Color legend | | | | | | | | | | | |
| --- | --- | --- | --- | --- | --- | --- | --- | --- | --- | --- | --- |
| q-value | 1 | 0.2 | 0.05 | 0.01 | 0.001 | 0.0001 |
| Color |  | |  |  |  | |

TABLE OF Q-VALUES

| aspergillus fumigatus conidia a549 | candida albicans neutrophils | Gene Set |
| --- | --- | --- |
| 1.5328516E-5 | 0.19693953 | NETPATH\_IL\_1\_PATHWAY\_UP |
| 0.0 | 0.10874915 | NETPATH\_TNF\_ALPHA\_PATHWAY\_DOWN |
| 1.4434879E-4 | 0.035003204 | NCI\_NFAT\_TFPATHWAY |
| 6.440544E-4 | 0.18667124 | NETPATH\_WNT\_PATHWAY\_UP |
| 2.8740968E-5 | 0.10933371 | NETPATH\_IL\_3\_PATHWAY\_UP |
| 0.0015525026 | 0.17791544 | REACTOME\_CLASS\_A1\_RHODOPSIN\_LIKE\_RECEPTORS |
| 1.9160645E-5 | 0.10786476 | REACTOME\_PEPTIDE\_LIGAND\_BINDING\_RECEPTORS |
| 2.0902522E-5 | 7.5262925E-4 | CYTOKINE\_ACTIVITY |
| 3.284682E-5 | 0.061718952 | NETPATH\_IL\_7\_PATHWAY\_UP |
| 2.8400484E-5 | 0.091335885 | CHEMOKINE\_ACTIVITY |
| 1.8933655E-5 | 0.1173318 | G\_PROTEIN\_COUPLED\_RECEPTOR\_BINDING |
| 0.0 | 0.08899127 | CHEMOKINE\_RECEPTOR\_BINDING |
| 0.0 | 0.17955464 | REACTOME\_CHEMOKINE\_RECEPTORS\_BIND\_CHEMOKINES |
| 0.02162187 | 0.18127525 | BIOCARTA\_STEM\_PATHWAY |
| 0.028019777 | 0.034272384 | FEEDING\_BEHAVIOR |
| -0.0 | 9.2519214E-4 | STRUCTURAL\_CONSTITUENT\_OF\_RIBOSOME |
| -0.0 | 4.3731768E-4 | NCI\_FORMATION\_OF\_A\_POOL\_OF\_FREE\_40S\_SUBUNITS |
| -0.0 | 5.686901E-4 | REACTOME\_PEPTIDE\_CHAIN\_ELONGATION |
| -0.0 | 3.7892838E-4 | NCI\_VIRAL\_MRNA\_TRANSLATION |
| -0.0 | 5.190721E-4 | REACTOME\_VIRAL\_MRNA\_TRANSLATION |
| 0.072709754 | 0.07456012 | DEVELOPMENTAL\_MATURATION |
| -0.0 | 0.0031905666 | REACTOME\_REGULATION\_OF\_GENE\_EXPRESSION\_IN\_BETA\_CELLS |
| -0.0 | 4.0424606E-4 | NCI\_GTP\_HYDROLYSIS\_AND\_JOINING\_OF\_THE\_60S\_RIBOSOMAL\_SUBUNIT |
| -0.0 | 3.6774037E-4 | NCI\_CAP\_DEPENDENT\_TRANSLATION\_INITIATION |
| -0.0 | 4.7714828E-4 | NCI\_EUKARYOTIC\_TRANSLATION\_TERMINATION |
| -0.0 | 3.8171865E-4 | NCI\_PEPTIDE\_CHAIN\_ELONGATION |
| -0.0 | 4.142596E-4 | KEGG\_RIBOSOME |
| -0.0 | 4.919824E-4 | REACTOME\_GTP\_HYDROLYSIS\_AND\_JOINING\_OF\_THE\_60S\_RIBOSOMAL\_SUBUNIT |
| -0.0 | 0.017057288 | REACTOME\_REGULATION\_OF\_BETA\_CELL\_DEVELOPMENT |
| -0.0 | 0.049720928 | REACTOME\_INFLUENZA\_VIRAL\_RNA\_TRANSCRIPTION\_AND\_REPLICATION |
| -0.0 | 4.377411E-4 | REACTOME\_FORMATION\_OF\_A\_POOL\_OF\_FREE\_40S\_SUBUNITS |
| -0.0 | 6.10188E-4 | REACTOME\_TRANSLATION |
| -0.0 | 3.4322432E-4 | CORUM\_RIBOSOME\_CYTOPLASMIC |
| -0.0 | 3.8062505E-4 | NCI\_L13A\_MEDIATED\_TRANSLATIONAL\_SILENCING\_OF\_CERULOPLASMIN\_EXPRESSION |
