# Supplementary material for: Computational approaches for discovery of common immunomodulators in fungal infections: towards broad-spectrum immunotherapeutic interventions
Source: BMC Microbiol. 2013 Oct 7;13:224. doi: 10.1186/1471-2180-13-224 (PMC3853472; doi:10.1186/1471-2180-13-224)
Supplement: Additional file 1 — Details of up- and down- regulated biclusters. [file 1471-2180-13-224-S1.zip › 2013-kidane-bmc/details-of-biclusters/upreg-biclust-2.html]

**BICLUSTER\_ID** : UPREG-2  
**PATHOGENS** /2/ : a. fumigatus,c. albicans  
**KNOWN DRUG TARGETS** /111/ : CA2, PPARD, INDO, GPR109B, ISG20, ABL2, IFNG, CD86, TGM2, CXCL10, CFB, ABCA1, IL6R, PPARG, GSK3B, SERPINE1, F3, CXCR4, CKB, CCL20, CTPS, BCL2, CCL2, TREM1, SERPINB2, GLA, CD80, CASP7, SLC1A3, PRNP, FABP3, NNMT, CALCA, AMPD3, CCL5, GP1BA, CD55, SERPINA1, ANXA1, NP, LTA, CYP27B1, EGLN3, SNAP25, IL6, ANPEP, PTGS2, TLR2, TNF, MMP12, ALDH1A2, DDAH2, PLAT, SLC7A11, GFPT2, CTNNB1, ADORA2A, PTPN1, NFKB1, PTGER4, GPRC5A, PPIF, ABCC1, SLC22A4, DPP4, KCNE1, B4GALT1, FYN, MET, ACVR1, IL1B, CDK2, ICAM1, THBS1, SMOX, UAP1, PDE4B, ACSL1, CD40, LPL, TNFRSF1B, PTGIR, CCND1, IL1R2, SLC7A1, PIM1, CD59, ADORA2B, IL2RA, GZMB, CTLA4, COL6A1, NRP1, IL12B, MMP9, WARS, THBD, NR3C1, TOP1, TFPI, IL2RG, ADSS, KCNN4, F5, TAC1, PLOD2, MAPKAPK2, PTGER2, IL8, PLAUR, HSD11B1  

| Gene Set | Leading Edge Genes |
| --- | --- |
| NETPATH IL 2 PATHWAY UP | PFKFB3, PTPN22, DUSP6, IRF4, ETS1, PMAIP1, FOS, CFLAR, IFIT1, CSF1, LCP2, SERPINE1, UPP1, SOCS2, SLC39A8, GBP1, BCL2, CTPS, TCEB1, TNFRSF4, RELA, IL4R, IER3, DUSP5, LTA, SNAP25, IL6, ID2, SLC7A5, OSM, TNF, MX1, STAT5A, BTG3, IFI44, CREM, NFKB1, B4GALT5, IL18R1, DPP4, GADD45G, SRC, XBP1, RGS1, FYN, IL1B, ICAM1, IRF1, DUSP4, STAT4, PDE4B, BCL2L1, ACSL1, TNFRSF1B, CCND1, SOCS3, NR4A2, PIM1, IL2RA, SLAMF1, CSF2, GZMB, PPRC1, EZH2, CCL4, KLF6, LIF, TOP1, IL2RG, ETS2, MAPKAPK2, DAXX, IL8, PTGER2, CHSY1, PLAUR |
| NETPATH TGFBETA RECEPTOR PATHWAY UP | CDH4, PTPN22, INDO, HERPUD1, HBEGF, FARP1, ETS1, CLCF1, NFKBIA, EIF2C2, INHBA, IRAK2, KCNA3, RGS2, EDN1, CRADD, MAPK6, TNFRSF12A, WBP4, CYR61, RRAD, TNFAIP6, IFRD1, SERPINE1, F3, IL12A, HIVEP2, CKB, CCRN4L, IL1RAP, CTPS, RELA, FOSL1, NFATC1, AKAP12, CCL5, CXCL11, MMP1, ID4, IER3, INSIG1, DUSP5, KLF10, NP, SPRY2, TM4SF1, TGFA, IL6, ATF3, PTGS2, LILRB2, PMP22, OSM, RHOC, SLC7A5, CDKN2B, SATB1, SERPINB8, MSC, DDX21, ADAM19, TRIB3, MAP3K8, CHST11, PHLDA2, NRAS, GFPT2, SLC7A11, GTPBP2, EXT1, RELB, TNC, PDK3, ACOX3, SERPINB9, FYN, RYBP, ACVR1, CRY1, ICAM1, TFPI2, MARCKSL1, MYC, THBS1, RAI14, GADD45B, UAP1, ITGA6, BAMBI, SOX4, SMAD7, CCND1, MAPRE2, ARID5A, SLC7A1, PIM1, BMP1, MMP9, KIAA0247, ETS2, SKIL, ITGAV, PLOD2, TANK, MAPKAPK2, DAXX, PLAUR |
| NETPATH TNF ALPHA PATHWAY UP | RND1, IL7R, OPTN, IFIT5, HBEGF, ETS1, PMAIP1, NFKBIA, PDLIM4, INHBA, KLF7, CXCL10, CFLAR, ABCA1, CXCL1, CSF1, CYB5D1, PIM3, TAF9, CYR61, TNFAIP3, IL15RA, BID, PNRC1, NFKB2, CCL2, DPM1, CLIC4, CASP7, TRAF1, CXCL3, TBC1D4, CCL5, CXCL11, ALCAM, PTX3, IER3, ANXA1, DFNA5, PPP1R15A, KLF10, MITF, SLCO3A1, IL6, PTGS2, DUSP1, MX1, TRAF4, G0S2, TP53BP2, STAT5A, BTG3, TNFSF8, GFPT2, EGR1, RIPK2, CXCL2, RELB, SOD2, EMP3, NFKB1, TNIP1, RBBP8, SLC22A4, IL18R1, GBP2, BCL2A1, CKS2, PHLDA3, TNFAIP8, TNFAIP2, EFNA1, SERPINB9, WTAP, TNFRSF9, IL1B, CHST2, ICAM1, IRF1, NFKBIB, NR4A3, SMOX, STAT4, BCL2L1, LPL, TNFRSF1B, GK, SDC4, IL2RA, CSF2, IFIT2, PDGFB, COL6A1, BIRC3, IL12B, ADAM17, ETS2, F5, TNFAIP1, IFI27, MRPL39, IL8, PLAUR |
| NETPATH IL 1 PATHWAY UP | UCHL3, MMP12, HBEGF, DUSP6, NFKBIA, INHBA, CXCL2, SOD2, CFB, NFKB1, CXCL1, IL1A, TNFAIP6, CKS2, SERPINE1, IL1B, ICAM1, MYC, NR4A3, CCL20, CCL2, SERPINB2, RELA, PRNP, CXCL3, FOSL1, TNFRSF11B, PIM2, AMPD3, UST, MMP10, CSF2, MMP1, CCL4, PTX3, BIRC3, BMP1, LIF, WARS, NP, TFPI, CXCL5, IL6, PTGS2, IL8, HSD11B1 |
| APOPTOSIS GO | ESPL1, DDAH2, TP53BP2, BCL2L11, PHLDA2, CLCF1, TNFSF8, PMAIP1, NFKBIA, INHBA, TNFRSF18, ADORA2A, CFLAR, NFKB1, BTG1, IL1A, PDCD1, TNFSF18, BCL2A1, SEMA4D, CD38, TNFAIP8, SERPINB9, TNFSF9, IL1B, ACVR1, TNFRSF9, IL12A, SOCS2, BID, ERN1, GADD45B, SPHK1, BCL2, BCL2L1, CCL2, SERPINB2, RELA, CASP7, GADD45A, SOCS3, PLAGL1, PIM1, IL2RA, SAP30BP, GZMB, TNFSF14, IER3, IL12B, PPP1R15A, ANGPTL4, MALT1, LTA, IL6, DAXX, TLR2, TNF, MX1 |
| RESPONSE TO EXTERNAL STIMULUS | CCL22, EREG, PLAT, CCL24, INHBA, GNA12, CXCL2, FOS, RIPK2, IRAK2, CXCL10, ADORA2A, NFKB1, CCR7, CXCL1, C5AR1, IL1A, PPARG, TNFAIP6, CHST2, SERPINE1, GPR68, CCL20, IL1RAP, CCL1, CCL2, CD40, RELA, CXCR3, FOSL1, MGLL, CCL5, GP1BA, CXCL11, S100A9, CCL4, PTX3, ANXA1, THBD, TFPI, CXCL5, F5, CDKN2B, IL8, PLAUR |
| PROGRAMMED CELL DEATH | ESPL1, DDAH2, TP53BP2, BCL2L11, PHLDA2, CLCF1, TNFSF8, PMAIP1, NFKBIA, INHBA, TNFRSF18, ADORA2A, CFLAR, NFKB1, BTG1, IL1A, PDCD1, TNFSF18, BCL2A1, SEMA4D, CD38, TNFAIP8, SERPINB9, TNFSF9, IL1B, ACVR1, TNFRSF9, IL12A, SOCS2, BID, ERN1, GADD45B, SPHK1, BCL2, BCL2L1, CCL2, SERPINB2, RELA, CASP7, GADD45A, SOCS3, PLAGL1, PIM1, IL2RA, SAP30BP, GZMB, TNFSF14, IER3, IL12B, PPP1R15A, ANGPTL4, MALT1, LTA, IL6, DAXX, TLR2, TNF, MX1 |
| NETPATH IL 4 PATHWAY UP | CA2, CCL22, ALDH1A2, TP53BP2, BCL2L11, ELL2, ARID5B, CH25H, DUSP6, FJX1, FOXC1, EXT1, INHBA, AIM1, CD86, FOS, RIPK2, CREM, TNC, NFKB1, RGS2, SLC22A4, EBI2, IL10RA, F3, MET, MYC, UPP1, SLC39A8, BCL2, ETV3, CCL2, RELA, IL4R, MAP3K14, SOCS3, NNMT, IL1RN, HSPB8, S100A9, GZMB, PTX3, ANXA1, REL, RASGRP1, TFPI, EGLN3, DKK2, IL6, RSAD2, PTGER2, SLC11A2, SATB1 |
| KEGG CYTOKINE CYTOKINE RECEPTOR INTERACTION | CCL22, IL7R, CCL24, CRLF2, CLCF1, TNFSF8, IL23A, INHBA, CXCL2, TNFRSF18, CXCL10, CCR7, CXCL1, CSF1, IL18R1, IL1A, TNFRSF12A, TNFSF18, TNFSF9, IL10RA, MET, IL1B, ACVR1, TNFRSF9, IL15RA, IL12A, CCL20, CCL1, CCL2, CD40, TNFRSF1B, CXCR3, TNFRSF4, IL1R2, IL4R, CXCL3, TNFRSF11B, CCL5, IL2RA, CSF2, CXCL11, CCL4, TNFSF14, PDGFB, IL12B, LTA, CXCL5, IL6, IL8, OSM, KITLG, TNF |
| IMMUNE SYSTEM PROCESS | DYRK3, CCL22, CST7, EREG, IL7R, CCL24, ETS1, INHBA, CD86, EBI3, PTGER4, CSF1, DPP4, CD274, PDCD1, GBP2, SEMA4D, LCP2, RGS1, GEM, FYN, XBP1, IL12A, CD83, CCL20, BCL2, NCK2, CCL2, TREM1, CD276, TNFRSF4, IL1R2, IL4R, CCL5, IL2RA, CCL4, IL12B, MMP9, MALT1, TM7SF4, IL6, RSAD2, LILRB2, TNFAIP1, IL8 |
| REACTOME GPCR LIGAND BINDING | OPN3, CCL22, GPR109B, GAL, CXCL2, GNG4, CXCL10, ADORA2A, PTGER4, CCR7, CXCL1, EDN1, C5AR1, CXCR4, NMB, GPR68, CCL20, CCL2, CXCR3, PTGIR, CXCL3, CALCA, GPR132, CCL5, ADORA2B, CXCL11, CCL4, ANXA1, WNT10B, CXCL5, TAC1, IL8, PTGER2 |
| RESPONSE TO WOUNDING | CCL22, EREG, PLAT, CCL24, RIPK2, IRAK2, FOS, CXCL2, CXCL10, ADORA2A, NFKB1, CCR7, CXCL1, IL1A, TNFAIP6, SERPINE1, CHST2, GPR68, CCL20, IL1RAP, CD40, RELA, MGLL, CCL5, GP1BA, S100A9, CXCL11, PTX3, CCL4, ANXA1, THBD, TFPI, F5, IL8 |
| REGULATION OF APOPTOSIS | DDAH2, TP53BP2, BCL2L11, CLCF1, TNFSF8, PMAIP1, INHBA, TNFRSF18, CFLAR, NFKB1, BTG1, IL1A, GSK3B, TNFSF18, BCL2A1, TNFAIP8, SEMA4D, CD38, SERPINB9, TNFRSF9, ACVR1, IL12A, SOCS2, BID, ERN1, SPHK1, BCL2, BCL2L1, CCL2, SERPINB2, RELA, SOCS3, PLAGL1, PIM1, TNFSF14, SAP30BP, IER3, BIRC3, IL12B, ANGPTL4, MALT1, LTA, IL6, DAXX, TLR2, TNF, MX1 |
| REGULATION OF PROGRAMMED CELL DEATH | DDAH2, TP53BP2, BCL2L11, CLCF1, TNFSF8, PMAIP1, INHBA, TNFRSF18, CFLAR, NFKB1, BTG1, IL1A, GSK3B, TNFSF18, BCL2A1, TNFAIP8, SEMA4D, CD38, SERPINB9, TNFRSF9, ACVR1, IL12A, SOCS2, BID, ERN1, SPHK1, BCL2, BCL2L1, CCL2, SERPINB2, RELA, SOCS3, PLAGL1, PIM1, TNFSF14, SAP30BP, IER3, BIRC3, IL12B, ANGPTL4, MALT1, LTA, IL6, DAXX, TLR2, TNF, MX1 |
| IMMUNE RESPONSE | CCL22, CST7, EREG, IL7R, CCL24, ETS1, LAT, CD86, EBI3, IRF8, PTGER4, C5AR1, IFI6, CD274, DPP4, GBP2, PDCD1, AQP9, LCP2, FYN, GEM, RGS1, IL12A, CD83, CCL20, BCL2, CCL2, TREM1, TNFRSF4, IL1R2, IL4R, CCL5, IL2RA, CCL4, IL12B, MALT1, IL6, LILRB2, RSAD2, TNFAIP1 |
| NETPATH IL 5 PATHWAY UP | TPM4, HBEGF, DUSP6, GPR109B, EGR1, RELB, NFKB1, PPIF, MAPK6, IL1A, RAB21, EGR3, LCP2, XBP1, IL1B, ICAM1, UPP1, NFKBIB, BCL2, NFKB2, PDE4B, CCL2, RELA, GADD45A, PRNP, GK, CD55, IL2RA, PIM1, IER3, REL, DUSP5, PMP22, IL8 |
| CELL CELL SIGNALING | CCL22, NDP, PPARD, EREG, CCL24, HOMER1, TNFSF8, TXN, SCG5, CCL17, INHBA, CXCL10, ADORA2A, IL1F9, GJB2, KIF1B, TNFSF18, TNFAIP6, EFNA1, TNFSF9, IL1B, SSTR3, NMB, CCL20, CD9, PTGIR, SLC1A3, CALCA, CCL5, S100A9, CXCL11, CCL4, NRP1, LIF, KLF10, LTA, ADAM17, SPRY2, TFAP2C, CXCL5, SNAP25, LILRB2, PMP22, OSM |
| EXTRACELLULAR REGION | CCL22, NDP, EREG, HBEGF, FJX1, INHBA, CXCL2, EBI3, CXCL1, TFRC, EDN1, IL1A, TNFAIP2, GREM1, IL1B, FXYD6, IL12A, CCL20, CCL1, CCL2, LPL, NUCB2, GLA, CXCL3, DKK3, CALCA, IL1RN, TNFRSF11B, MMP10, PTX3, CCL4, SERPINA1, IL12B, MMP9, ANGPTL4, DKK2, IL6, TAC1, IL8, OSM |
| KEGG TOLL LIKE RECEPTOR SIGNALING PATHWAY | CD40, MAP3K8, SPP1, CD80, RELA, NFKBIA, MAPK13, CCL5, CD86, CXCL10, CCL4, NFKB1, IL12B, PIK3R5, IL6, IL1B, IL12A, IL8, IRF7, TICAM1, TLR2, TNF |
| NETPATH EGFR1 PATHWAY UP | CA2, DDX21, EMP1, EREG, HBEGF, DUSP6, PHLDA2, LAD1, EGR1, PDLIM4, KCTD11, TGM2, GPRC5A, GJB2, EHD1, TNFAIP3, MET, TFPI2, MYC, SNAI1, NR4A3, DUSP4, SPP1, SERPINB2, CCND1, GADD45A, IL4R, MGLL, DKK3, AKAP12, SDC4, MT1M, UBE2N, IER3, MMP1, ANXA1, LIF, PHLDA1, NP, CYP27B1, SPRY2, TGFA, CXCL5, ADSS, PTGS2, DUSP1, PLAUR |
| NEGATIVE REGULATION OF DEVELOPMENTAL PROCESS | DDAH2, EREG, CLCF1, INHBA, TNFRSF18, CFLAR, NFKB1, IL1A, GSK3B, TNFSF18, BCL2A1, TNFAIP8, SEMA4D, SERPINB9, ACVR1, SOCS2, BNIP3, SPHK1, AKT1S1, BCL2, BCL2L1, CCL2, SERPINB2, RELA, SOCS3, CALCA, PIM1, IER3, ANXA1, BIRC3, ANGPTL4, IL6, TNF |
| KEGG COMPLEMENT AND COAGULATION CASCADES | SERPINA1, C5AR1, PLAT, THBD, TFPI, F5, SERPINE1, F3, CD59, CD55, CFB, PLAUR |
| DEFENSE RESPONSE | CCL22, EREG, CCL24, INHBA, RIPK2, CXCL2, IRAK2, FOS, CXCL10, ADORA2A, NFKB1, CCR7, TNIP1, CXCL1, IL1A, TNFAIP6, CHST2, IL12A, BNIP3, CD83, GPR68, CCL20, MX2, IL1RAP, BCL2, CD40, RELA, FOSL1, MGLL, VEZF1, CCL5, S100A9, CCL4, PTX3, ANXA1, IL12B, LILRA3, KCNN4, RSAD2, LILRB2, IL8, MX1 |
| NETPATH TNF ALPHA PATHWAY DOWN | DDAH2, IFIT5, NFKBIA, EGR1, EXT1, CXCL2, NFKB1, CXCL1, ZHX2, FOSB, MAPK6, GSK3B, EGR3, TNFAIP2, MAFF, GEM, TNFAIP3, IRF1, SYNJ2, THBS1, CKB, CCL20, CD80, CCND1, CXCL3, MAP3K14, SDC4, IER3, REL, INSIG1, KLF6, PPP1R15A, KLF10, NR3C1, DDX3X, IL6, DUSP2, PTGS2 |
| REACTOME CLASS A1 RHODOPSIN LIKE RECEPTORS | GPR68, NMB, CCL22, OPN3, CCL20, CCL2, CXCR3, CXCL3, GPR109B, GAL, GPR132, CCL5, CXCL2, CXCL11, CXCL10, CCL4, ADORA2A, PTGER4, CCR7, CXCL1, EDN1, C5AR1, CXCL5, TAC1, IL8, PTGER2 |
| NEGATIVE REGULATION OF APOPTOSIS | SPHK1, DDAH2, BCL2, CCL2, BCL2L1, SERPINB2, RELA, CLCF1, SOCS3, PIM1, TNFRSF18, IER3, CFLAR, NFKB1, BIRC3, ANGPTL4, GSK3B, IL1A, TNFSF18, BCL2A1, TNFAIP8, SEMA4D, IL6, SERPINB9, ACVR1, SOCS2, TNF |
| NETPATH IL 4 PATHWAY DOWN | TNS1, CCL20, GADD45B, IL7R, CCL2, CD9, LPL, SPP1, NFKBIA, CXCL2, IL2RA, PDGFB, GBP2, GEM, IL6, PTGS2, CHST2, ICAM1, NINJ1, IRF1, IL8, TLR2, MX1, TNF |
| RECEPTOR BINDING | CCL22, NDP, EREG, CCL24, HBEGF, SORBS1, CLCF1, REEP1, INHBA, CXCL2, CXCL10, CXCL1, CSF1, EDN1, SEMA4D, EFNA1, OASL, IL12A, SOCS2, BID, NMB, JMJD1C, CCL20, CCL1, CCL2, GLA, SQSTM1, CXCL3, IL1RN, CALCA, TNFRSF11B, CCL5, CSF2, SPRED2, ALCAM, CXCL11, CCL4, DLL1, ANXA1, IL12B, IL1F5, TGFA, CXCL5, INHBC, DAXX, IL8, OSM, PLXNC1, TNF |
| NEGATIVE REGULATION OF PROGRAMMED CELL DEATH | SPHK1, DDAH2, BCL2, CCL2, BCL2L1, SERPINB2, RELA, CLCF1, SOCS3, PIM1, TNFRSF18, IER3, CFLAR, NFKB1, BIRC3, ANGPTL4, GSK3B, IL1A, TNFSF18, BCL2A1, TNFAIP8, SEMA4D, IL6, SERPINB9, ACVR1, SOCS2, TNF |
| KEGG HEMATOPOIETIC CELL LINEAGE | IL7R, CD9, ITGA6, IL4R, IL1R2, GP1BA, IL2RA, CD55, CD59, CSF2, CSF1, TFRC, IL6R, IL1A, CD38, IL6, ANPEP, IL1B, CD8A, KITLG, TNF |
| EXTRACELLULAR REGION PART | CCL22, NDP, EREG, HBEGF, FJX1, INHBA, CXCL2, EBI3, CXCL1, EDN1, IL1A, TNFAIP2, GREM1, IL1B, IL12A, FXYD6, CCL20, CCL1, CCL2, NUCB2, CXCL3, IL1RN, MMP10, CCL4, SERPINA1, IL12B, MMP9, DKK2, IL6, TAC1, OSM, IL8 |
| REGULATION OF CELL PROLIFERATION | EREG, BTG3, ETS1, CD86, EBI3, CXCL10, BTG2, CSF1, CXCL1, EDN1, BTG1, IL1A, TNFRSF9, IL1B, CDK2, MYC, SPHK1, NCK2, CD276, FABP3, FOSL1, MATK, SLAMF1, NRP1, ARHGEF2, IL12B, KLF10, TGFA, CXCL5, IL6, IL8, OSM, CDKN2B |
| EXTRACELLULAR SPACE | CCL22, NDP, EREG, HBEGF, FJX1, INHBA, CXCL2, EBI3, CXCL1, EDN1, IL1A, TNFAIP2, GREM1, IL1B, IL12A, FXYD6, CCL20, CCL1, CCL2, NUCB2, CXCL3, DKK3, IL1RN, MMP10, CCL4, SERPINA1, IL12B, MMP9, DKK2, IL6, TAC1, OSM, IL8 |
| NETPATH KIT RECEPTOR PATHWAY UP | CCL22, SNAI1, DUSP4, BCL2, BCL2L1, CCL2, CCND1, CXCR3, RELA, EGR1, CCL5, FOS, PIM1, CSF2, IER3, MMP9, IL2RG, IL6, KITLG, TNF |
| NCI AMB2 NEUTROPHILS PATHWAY | NFKB1, MMP9, LAMB3, PLAT, CYR61, SRC, FYN, IL6, ICAM1, PLAUR, TNF |
| KEGG VIRAL MYOCARDITIS | CD40, CD80, CCND1, FYN, ICAM1, ABL2, CD55, CD86, BID |
| INFLAMMATORY RESPONSE | GPR68, CCL22, CCL20, IL1RAP, CD40, CCL24, RELA, MGLL, CCL5, RIPK2, IRAK2, FOS, CXCL2, S100A9, CXCL10, CCL4, PTX3, ADORA2A, NFKB1, ANXA1, CCR7, CXCL1, IL1A, TNFAIP6, CHST2, IL8 |
| WOUND HEALING | EREG, PLAT, THBD, TFPI, F5, SERPINE1, GNA12, GP1BA, ADORA2A |
| CYTOKINE ACTIVITY | CCL22, CCL20, CCL2, CCL1, CCL24, CXCL3, IL1RN, TNFRSF11B, CCL5, INHBA, CXCL2, SPRED2, CXCL11, CSF2, CXCL10, CCL4, IL12B, CSF1, CXCL1, IL1F5, CXCL5, IL12A, OSM, IL8, TNF |
| KEGG NOD LIKE RECEPTOR SIGNALING PATHWAY | NFKB1, NFKBIB, CXCL1, CCL2, NFKBIA, IL6, TNFAIP3, IL1B, MAPK13, CCL5, IL8, RIPK2, CXCL2, TNF |
| NCI NFAT TFPATHWAY | CTLA4, PTPN1, IRF4, EGR3, FOSL1, EGR1, PTGS2, NFATC1, CBLB, IL8, IL2RA, IFNG, FOS, CSF2, TNF |
| NCI IL23PATHWAY | NFKB1, IL12B, CXCL1, STAT4, CCL2, IL18R1, STAT5A, RELA, SOCS3, NFKBIA, IL6, IL1B, STAT3, IL23A, IFNG, TNF |
| REACTOME G ALPHA I SIGNALLING EVENTS | SSTR3, OPN3, CCL20, CXCR3, CXCL3, GPR109B, GAL, CXCL16, CCL5, CXCL2, CXCL11, GNG4, GNAI1, CXCL10, ANXA1, CCR7, CXCL1, C5AR1, CXCL5, IL8, CXCR4 |
| NCI IL12 2PATHWAY | GADD45B, NFKB2, STAT4, STAT5A, RELA, FOS, IFNG, RIPK2, RELB, IL2RA, CCL4, GZMB, NFKB1, IL12B, IL18R1, GADD45G, IL2RG, IL1B, STAT3, IL12A, CD8A |
| COAGULATION | F5, SERPINE1, GNA12, PLAT, GP1BA, THBD, TFPI, ADORA2A |
| KEGG TYPE I DIABETES MELLITUS | IL12B, CD80, IL1A, LTA, IL1B, IL12A, CD86, TNF, GZMB |
| ANTI APOPTOSIS | DDAH2, SPHK1, BCL2, BCL2L1, CCL2, SERPINB2, RELA, SOCS3, TNFRSF18, CFLAR, IER3, NFKB1, ANXA1, BIRC3, IL1A, TNFSF18, BCL2A1, SEMA4D, TNFAIP8, SERPINB9, SOCS2, TNF |
| BEHAVIOR | CCL22, CCL20, CCL1, CCL2, CCL24, CXCR3, FOSL1, CCL5, CXCL2, CXCL10, CXCL11, CCR7, CXCL1, FOSB, C5AR1, CXCL5, FYN, IL8, PLAUR |
| NETPATH IL 3 PATHWAY UP | BCL2, BCL2L1, CCL2, SPP1, SOCS3, MATK, PIM1, CD86, CCL4, CSF1, USP36, TGFA, IL1B, IL8, OSM, SOCS2, TNF |
| KEGG ALLOGRAFT REJECTION | IL12B, CD40, CD80, IL12A, IFNG, CD86, TNF, GZMB |
| BLOOD COAGULATION | F5, SERPINE1, GNA12, PLAT, GP1BA, THBD, TFPI, ADORA2A |
| KEGG GRAFT VERSUS HOST DISEASE | IL6, IL1B, CD86, CD80, IL1A, TNF, GZMB |
| REACTOME PEPTIDE LIGAND BINDING RECEPTORS | CCL22, NMB, CCL20, CCL2, CXCR3, CXCL3, GAL, CCL5, CXCL2, CXCL10, CXCL11, CCL4, CCR7, CXCL1, EDN1, C5AR1, CXCL5, TAC1, IL8 |
| NETPATH IL 7 PATHWAY UP | CCL22, BCL2, BCL2L1, STAT5A, CD80, TNFSF8, CXCL3, CCL5, CD86, CXCL2, IL2RA, CCL4, CXCL1, TFRC, CXCL5, IL8 |
| KEGG T CELL RECEPTOR SIGNALING PATHWAY | NFKBIB, NFAT5, NCK2, MAP3K8, NRAS, RELA, MAP3K14, NFKBIA, CBLB, NFATC1, PPP3CC, MAPK13, FOS, IFNG, CSF2, NFKB1, CTLA4, MALT1, GSK3B, RASGRP1, PDCD1, LCP2, PIK3R5, FYN, CD8A, TNF |
| RESPONSE TO BIOTIC STIMULUS | CCL22, ABCE1, ERN1, BCL2, HERPUD1, HSPA2, FOSL1, IFI44, CCL5, ISG20, CCL4, IL12B, DNAJB5, EIF2AK3, FGR, GSK3B, RSAD2, IL12A, IRF7, TNF |
| KEGG JAK STAT SIGNALING PATHWAY | STAT4, IL7R, BCL2L1, STAT5A, CCND1, CLCF1, CRLF2, IL4R, SOCS3, CBLB, IL23A, PIM1, IL2RA, CSF2, SPRED2, IL12B, LIF, SPRY2, IL2RG, PIK3R5, IL6, IL10RA, IL15RA, IL12A, OSM, SPRY1, SOCS2, MYC |
| KEGG LEISHMANIA INFECTION | NFKB1, NFKBIB, IL12B, IL1A, RELA, NFKBIA, IL1B, PTGS2, IL12A, MAPK13, MARCKSL1, FOS, TLR2, TNF |
| LOCOMOTORY BEHAVIOR | CCL22, CCL20, CCL1, CCL2, CCL24, CXCR3, FOSL1, CCL5, CXCL2, CXCL10, CXCL11, CCR7, CXCL1, C5AR1, CXCL5, IL8, PLAUR |
| POSITIVE REGULATION OF CELL PROLIFERATION | SPHK1, EREG, NCK2, CD276, FOSL1, MATK, CD86, SLAMF1, CXCL10, EBI3, IL12B, NRP1, LIF, CSF1, EDN1, TGFA, CXCL5, IL6, CDK2, MYC |
| CHEMOKINE ACTIVITY | CCL22, CCL20, CXCL1, CCL1, CCL2, CCL24, CXCL3, CXCL5, CCL5, IL8, CXCL2, CXCL10, CXCL11, CCL4 |
| REACTOME CHEMOKINE RECEPTORS BIND CHEMOKINES | CCL22, CCR7, CCL20, CXCL1, CCL2, CXCR3, CXCL3, CXCL5, CCL5, IL8, CXCL2, CXCL11, CXCL10, CCL4 |
| KEGG CELL ADHESION MOLECULES CAMS | CDH4, ITGA6, CD40, CD80, CD276, SDC4, CD86, ALCAM, CLDN1, CLDN14, CD58, CTLA4, CD274, PDCD1, PVRL2, ITGAV, ICAM1, CD8A, ITGA9 |
| NCI CD8TCRDOWNSTREAMPATHWAY | STAT4, NRAS, TNFRSF4, IL2RG, FOSL1, EGR1, TNFRSF9, NFATC1, CD8A, IFNG, FOS, TNFRSF18, IL2RA, TNF, GZMB |
| REACTOME FORMATION OF FIBRIN CLOT CLOTTING CASCADE | F5, F3, GP1BA, THBD, TFPI |
| KEGG MAPK SIGNALING PATHWAY | DDIT3, MAP3K8, DUSP6, NRAS, MAPK13, RELB, FOS, RPS6KA3, NFKB1, MAP3K4, IL1A, GADD45G, IL1B, MAP4K4, DUSP4, GADD45B, NFKB2, DUSP10, RELA, GADD45A, DUSP14, IL1R2, MAP3K14, PPP3CC, DUSP8, PDGFB, STK4, DUSP5, RASGRP1, DUSP2, MAPKAPK2, DAXX, DUSP1, TNF |
| NCI LYSOPHOSPHOLIPID PATHWAY | NFKB1, MMP9, HBEGF, GSK3B, GNA13, TIAM1, RELA, SRC, NFKBIA, IL6, GNA12, IL8, FOS, GNAI1 |
| KEGG PRION DISEASES | PRNP, IL6, FYN, EGR1, IL1B, CCL5, IL1A |
| BIOCARTA CYTOKINE PATHWAY | IL6, IL12B, IL12A, IL8, IL1A, LTA, TNF |
| CHEMOKINE RECEPTOR BINDING | CCL22, CCL20, CXCL1, CCL1, CCL2, CCL24, CXCL3, CXCL5, CCL5, IL8, CXCL2, CXCL10, CXCL11, CCL4 |
| KEGG AUTOIMMUNE THYROID DISEASE | CTLA4, CD86, CD40, CD80, GZMB |
| KEGG RIG I LIKE RECEPTOR SIGNALING PATHWAY | NFKB1, NFKBIB, IL12B, ISG15, IFIH1, RELA, DDX3X, TRIM25, NFKBIA, IL12A, TANK, MAPK13, IL8, IRF7, CXCL10, TNF |
| NETPATH IL 6 PATHWAY UP | GADD45B, PPARD, HBEGF, MAP3K8, SLC39A14, SPP1, SOCS3, RHOBTB3, PIM2, TNFRSF11B, FOS, PIM1, ABCC1, CXCL1, MMP9, MAFF, STAT3, ID2, IRF1, SOCS2, MX1, TNF |
| NCI SYNDECAN 4 PATHWAY | THBS1, MMP9, SDC4, CCL5, CXCR4, TFPI, TNC |
| BIOCARTA INFLAM PATHWAY | IL12B, CSF1, IL1A, LTA, IL6, IL12A, IL8, CSF2, TNF |
| NCI IL27PATHWAY | IL12B, STAT4, STAT5A, IL6, IL1B, IL12A, STAT3, IFNG, EBI3, TNF |
| G PROTEIN COUPLED RECEPTOR BINDING | CCL22, CCL20, CXCL1, CCL1, CCL2, CCL24, CXCL3, CXCL5, CCL5, IL8, CXCL2, CXCL10, CXCL11, CCL4 |
| KEGG CYTOSOLIC DNA SENSING PATHWAY | NFKB1, NFKBIB, RELA, NFKBIA, IL6, IL1B, CCL5, IRF7, CXCL10, DDX58, CCL4 |
| BIOCARTA IL1R PATHWAY | NFKB1, IL1A, IRAK3, RELA, MAP3K14, NFKBIA, IL1RN, IL6, IL1B, IRAK2, TNF |
| NCI DISSOLUTION OF FIBRIN CLOT | SERPINE1, PLAT, SERPINB2, PLAUR |
| BIOCARTA LAIR PATHWAY | IL6, ICAM1, IL8, IL1A, TNF |
| KEGG INTESTINAL IMMUNE NETWORK FOR IGA PRODUCTION | MAP3K14, ICOSLG, IL6, IL15RA, CD86, CD40, CD80, AICDA |
| RESPONSE TO OTHER ORGANISM | CCL22, BCL2, FOSL1, RSAD2, IFI44, ISG20, CCL5, IL12A, IRF7, CCL4, TNF |
| NCI ATF2 PATHWAY | DUSP5, BCL2, DUSP10, DDIT3, CCND1, GADD45A, SOCS3, CSRP2, IL6, ATF3, IL23A, DUSP8, FOS, IFNG, DUSP1 |
| HUMORAL IMMUNE RESPONSE | CD83, IL6, BCL2, CCL2, TREM1, EBI3, PDCD1 |
| RESPONSE TO VIRUS | CCL22, BCL2, FOSL1, RSAD2, IFI44, ISG20, CCL5, IRF7, TNF, CCL4 |
| BIOCARTA TH1TH2 PATHWAY | IL4R, IL12B, IL12A, CD40, CD86, IL18R1, IL2RA |
| POSITIVE REGULATION OF MULTICELLULAR ORGANISMAL PROCESS | IL12B, SPHK1, EREG, NCK2, MALT1, CD276, CALCA, FYN, IL12A, IFNG, EBI3 |
| KEGG EPITHELIAL CELL SIGNALING IN HELICOBACTER PYLORI INFECTION | NFKB1, CXCL1, HBEGF, ATP6V1D, RELA, MAP3K14, SRC, NFKBIA, MET, MAPK13, CCL5, IL8 |
| POSITIVE REGULATION OF CYTOKINE PRODUCTION | CALCA, IL12B, EREG, IL12A, IFNG, MALT1 |
| NETPATH IL 9 PATHWAY UP | SOCS3, MRAS, CCL2, PIM1, MYC, SOCS2, GZMB |
| NETPATH IL 1 PATHWAY | NFKB1, NFKBIB, IL1RAP, PELI1, MAP3K7IP2, IL1A, IRAK3, RELA, IL1R2, MAP3K14, NFKBIA, IL1RN, IL1B, IRAK2 |
| BIOCARTA FREE PATHWAY | NFKB1, IL8, RELA, TNF |
| BIOCARTA FIBRINOLYSIS PATHWAY | SERPINE1, PLAT, SERPINB2 |
| SA MMP CYTOKINE CONNECTION | TNFSF8, CSF1, IL1B, TNFRSF1B, TNF |
| KEGG ECM RECEPTOR INTERACTION | THBS1, COL6A1, LAMB3, ITGA6, SPP1, IBSP, ITGAV, SDC4, GP1BA, ITGA9, TNC |
| REACTOME INTEGRIN CELL SURFACE INTERACTIONS | PTPN1, THBS1, ITGA6, SPP1, IBSP, SRC, ITGAV, ICAM1, ITGA9, TNC |
| BIOCARTA DC PATHWAY | IL12B, IL12A, CD40, CSF2, TLR2 |
| BIOCARTA TNFR2 PATHWAY | NFKB1, LTA, TNFRSF1B, RELA, TRAF1, MAP3K14, NFKBIA, TNFAIP3, TANK, DUSP1 |
| NCI S1P S1P1 PATHWAY | ITGAV, SPHK1, ABCC1, PTGS2 |
| BIOCARTA STEM PATHWAY | IL6, CSF1, CD8A, IL8, CSF2 |
| INTERLEUKIN BINDING | IL2RG, IL4R, IL10RA, IL15RA, IL7R, IL12A, IL2RA, EBI3 |
| KEGG HYPERTROPHIC CARDIOMYOPATHY HCM | EMD, TPM4, ITGA6, ITGB7, ATP2A2, CACNG6, IL6, ITGAV, PRKAG2, ITGA9, TNF |
| NCI CHEMOKINE RECEPTORS BIND CHEMOKINES | CCL20, CCR7, CCL5 |
| NETPATH HEDGEHOG PATHWAY UP | PMP22, THBD, CCND1 |
| REACTOME PLATELET ADHESION TO EXPOSED COLLAGEN | FYN, GP1BA |
| BIOCARTA IL17 PATHWAY | CD58, IL6, CD8A, IL8, KITLG |
| NCI TCRCALCIUMPATHWAY | FOSL1, NFATC1, PTGS2, IL2RA, CSF2 |
| BIOCARTA CDMAC PATHWAY | NFKB1, NFKBIA, FOS, MYC, TNF, RELA |
| REACTOME AMINO ACID TRANSPORT ACROSS THE PLASMA MEMBRANE | SLC7A11, SLC16A10, SLC7A1, SLC7A5 |
| CYTOKINE METABOLIC PROCESS | IL6, IL12B, EREG, INHBA, EBI3, IRF4, CD276 |
| REACTOME TRAF6 MEDIATED INDUCTION OF THE ANTIVIRAL CYTOKINE IFN ALPHA BETA CASCADE | NFKB1, DUSP4, NFKBIB, NFKB2, DUSP6, NFKBIA, MAPKAPK2, FOS, RPS6KA3, TICAM1 |
| POSITIVE REGULATION OF IMMUNE RESPONSE | FYN, IL12B, EREG, IL12A, MALT1 |
| NCI CD40 PATHWAY | NFKB1, BIRC3, BCL2L1, CD40, STAT5A, BIRC2, RELA, TRAF1, MAP3K14, NFKBIA, TNFAIP3, CBLB, MYC |
| REGULATION OF IMMUNE RESPONSE | FYN, IL12B, EREG, IL12A, MALT1 |
| BIOCARTA ASBCELL PATHWAY | CD40, CD80 |
| HEMATOPOIETIN INTERFERON CLASSD200 DOMAIN CYTOKINE RECEPTOR BINDING | IL12B, IFNG, OSM, CSF2 |
| NCI NFKAPPABCANONICALPATHWAY | NFKB1, NFKBIA, TNFAIP3, RIPK2, CYLD, BIRC2, MALT1, TNF |
| DELAYED RECTIFIER POTASSIUM CHANNEL ACTIVITY | KCNA3, KCNE1 |
| NEGATIVE REGULATION OF CYTOKINE BIOSYNTHETIC PROCESS | IL6, INHBA |
| INTERFERON GAMMA PRODUCTION | IL12B, IL12A, INHBA, EBI3, CD276 |
| BIOCARTA NKT PATHWAY | CCR7, IL12B, IL18R1, CXCR3, IL4R, IL12A, CSF2, CCL4 |
| GROWTH FACTOR ACTIVITY | IL12B, NDP, CSF1, IL1F5, CLCF1, TGFA, IL1RN, INHBA, CSF2 |
| APICOLATERAL PLASMA MEMBRANE | B4GALT1, CLDN12, CLDN14, CLDN1, SORBS1, VAPA, TJP1 |
| NCI APOPTOTIC CLEAVAGE OF CELL ADHESION PROTEINS | TJP2, CTNNB1, TJP1 |
| BIOCARTA ERYTH PATHWAY | IL6, CSF2, IL1A, KITLG |
| REACTOME HUMAN TAK1 ACTIVATES NFKB BY PHOSPHORYLATION AND ACTIVATION OF IKKS COMPLEX | NFKB1, NFKBIA, NFKBIB, NFKB2, TICAM1, RELA |
| BIOCARTA CARDIACEGF PATHWAY | NFKB1, EDN1, FOS, MYC, RELA |
| POSITIVE REGULATION OF PEPTIDYL TYROSINE PHOSPHORYLATION | IL12A, CD80, CLCF1 |
| CORUM NFKB1-NFKB2-REL-RELA-RELB COMPLEX | NFKB1, REL, NFKB2, RELB, RELA |
| HORMONE SECRETION | SCG5, SNAP25, LIF, INHBA, OSM |
| REACTOME APOPTOTIC CLEAVAGE OF CELL ADHESION PROTEINS | TJP2, CTNNB1, TJP1 |
| CORUM CHUK-NFKB2-REL-IKBKG-SPAG9-NFKB1-NFKBIE-COPB2-TNIP1-NFKBIA-RELA-TNIP2 COMPLEX | NFKB1, NFKBIA, SPAG9, REL, TNIP1, NFKB2, TNIP2, RELA |
| INTERFERON GAMMA BIOSYNTHETIC PROCESS | IL12B, INHBA, EBI3, CD276 |
| NCI DOWNSTREAM TCR SIGNALING | NFKB1, NFKBIA, RIPK2, MALT1, MAP3K7IP2, RELA |
| NETPATH WNT PATHWAY DOWN | IL1B, CXCL1, SPP1, IBSP, TNFRSF4 |
| REGULATION OF INTERFERON GAMMA BIOSYNTHETIC PROCESS | IL12B, INHBA, EBI3, CD276 |
| REGULATION OF HORMONE SECRETION | SCG5, SNAP25, LIF, INHBA, OSM |
| KEGG GLYCOSAMINOGLYCAN BIOSYNTHESIS KERATAN SULFATE | B3GNT2, B4GALT1, CHST2 |
| HEMOSTASIS |  |
| NCI ERBB NETWORK PATHWAY | TGFA, EREG, HBEGF |
| KEGG GLYCOSPHINGOLIPID BIOSYNTHESIS LACTO AND NEOLACTO SERIES |  |
| NCI HIF1 TFPATHWAY |  |
| SKELETAL DEVELOPMENT |  |
| NCI INTEGRIN CELL SURFACE INTERACTIONS |  |
| POSITIVE REGULATION OF RESPONSE TO STIMULUS |  |
| GLYCOLIPID METABOLIC PROCESS |  |
| INTERCALATED DISC |  |
| CORUM TNF-ALPHA/NF-KAPPA B SIGNALING COMPLEX CHUK KPNA3 NFKB2 NFKBIB REL IKBKG NFKB1 NFKBIE RELB NFKBIA RELA TNIP2 |  |
| CALCIUM MEDIATED SIGNALING |  |
| REACTOME PLATELET DEGRANULATION |  |
| NEGATIVE REGULATION OF CELLULAR BIOSYNTHETIC PROCESS |  |
| CORUM TNF-ALPHA/NF-KAPPA B SIGNALING COMPLEX RPL6 RPL30 RPS13 CHUK DDX3X NFKB2 NFKBIB REL IKBKG NFKB1 MAP3K8 RELB GLG1 NFKBIA RELA TNIP2 GTF2I |  |
| ST TUMOR NECROSIS FACTOR PATHWAY |  |
| NCI HIF2PATHWAY |  |
| INACTIVATION OF MAPK ACTIVITY | DUSP2, DUSP8, DUSP6, SPRED2 |
| BIOCARTA PLATELETAPP PATHWAY |  |
| ANTIGEN BINDING | LILRB4, IL7R, LILRA3, SLC7A5, SLAMF1 |
| APICAL JUNCTION COMPLEX |  |
| REACTOME UNFOLDED PROTEIN RESPONSE | ERN1, DDIT3, DNAJB9, EIF2AK3, HERPUD1, EDEM1, ATF3, XBP1 |
| BILE ACID METABOLIC PROCESS | NPC1, ACOX3, HSD3B7 |
| KEGG ADIPOCYTOKINE SIGNALING PATHWAY |  |
| NEGATIVE REGULATION OF CELL PROLIFERATION |  |
| REGULATION OF BODY FLUID LEVELS |  |
| REACTOME STEROID HORMONES |  |
| HORMONE ACTIVITY | NMB, EDN1, INHBA |
| NCI TCRRASPATHWAY |  |
| GENERATION OF A SIGNAL INVOLVED IN CELL CELL SIGNALING |  |
| BIOCARTA DEATH PATHWAY |  |
| ACTIVATION OF IMMUNE RESPONSE |  |
| NCI IL12 STAT4PATHWAY |  |
| NETPATH WNT PATHWAY UP |  |
| REACTOME G ALPHA S SIGNALLING EVENTS |  |
| REGULATION OF MITOTIC CELL CYCLE |  |

| Color legend | | | | | | | | | | | |
| --- | --- | --- | --- | --- | --- | --- | --- | --- | --- | --- | --- |
| q-value | 1 | 0.2 | 0.05 | 0.01 | 0.001 | 0.0001 |
| Color |  | |  |  |  | |

TABLE OF Q-VALUES

| candida albicans moddc135 | aspergillus fumigatus dendritic | Gene Set |
| --- | --- | --- |
| 0.0 | 0.002894239 | NETPATH\_IL\_2\_PATHWAY\_UP |
| 3.2457785E-4 | 0.104725674 | NETPATH\_TGFBETA\_RECEPTOR\_PATHWAY\_UP |
| 2.4793292E-6 | 0.03056191 | NETPATH\_TNF\_ALPHA\_PATHWAY\_UP |
| 0.0 | 0.0 | NETPATH\_IL\_1\_PATHWAY\_UP |
| 1.3461978E-4 | 0.13303934 | APOPTOSIS\_GO |
| 2.43421E-4 | 0.024326107 | RESPONSE\_TO\_EXTERNAL\_STIMULUS |
| 1.4178407E-4 | 0.110362284 | PROGRAMMED\_CELL\_DEATH |
| 2.7103227E-4 | 0.0022578288 | NETPATH\_IL\_4\_PATHWAY\_UP |
| 0.0 | 0.0 | KEGG\_CYTOKINE\_CYTOKINE\_RECEPTOR\_INTERACTION |
| 2.410459E-6 | 0.17588222 | IMMUNE\_SYSTEM\_PROCESS |
| 0.0016598669 | 0.023223832 | REACTOME\_GPCR\_LIGAND\_BINDING |
| 2.1165008E-6 | 0.0047411146 | RESPONSE\_TO\_WOUNDING |
| 5.117286E-4 | 0.13189097 | REGULATION\_OF\_APOPTOSIS |
| 4.9259793E-4 | 0.13312297 | REGULATION\_OF\_PROGRAMMED\_CELL\_DEATH |
| 0.0 | 0.030914374 | IMMUNE\_RESPONSE |
| 2.7117665E-6 | 0.0033619835 | NETPATH\_IL\_5\_PATHWAY\_UP |
| 4.9217895E-4 | 0.009947655 | CELL\_CELL\_SIGNALING |
| 1.07919106E-4 | 0.001696513 | EXTRACELLULAR\_REGION |
| 2.892551E-6 | 0.091590926 | KEGG\_TOLL\_LIKE\_RECEPTOR\_SIGNALING\_PATHWAY |
| 0.0 | 3.9014107E-5 | NETPATH\_EGFR1\_PATHWAY\_UP |
| 2.2477773E-4 | 0.038165387 | NEGATIVE\_REGULATION\_OF\_DEVELOPMENTAL\_PROCESS |
| 0.0020074716 | 0.03170033 | KEGG\_COMPLEMENT\_AND\_COAGULATION\_CASCADES |
| 2.1694132E-6 | 0.106237434 | DEFENSE\_RESPONSE |
| 2.3453117E-6 | 0.0036943294 | NETPATH\_TNF\_ALPHA\_PATHWAY\_DOWN |
| 1.4066127E-4 | 0.003579989 | REACTOME\_CLASS\_A1\_RHODOPSIN\_LIKE\_RECEPTORS |
| 5.556078E-6 | 0.023920687 | NEGATIVE\_REGULATION\_OF\_APOPTOSIS |
| 0.0 | 0.0027640385 | NETPATH\_IL\_4\_PATHWAY\_DOWN |
| 0.0 | 0.009376848 | RECEPTOR\_BINDING |
| 3.9515476E-6 | 0.017299755 | NEGATIVE\_REGULATION\_OF\_PROGRAMMED\_CELL\_DEATH |
| 0.0 | 0.001619189 | KEGG\_HEMATOPOIETIC\_CELL\_LINEAGE |
| 4.004787E-5 | 6.125904E-5 | EXTRACELLULAR\_REGION\_PART |
| 0.00510492 | 0.11457803 | REGULATION\_OF\_CELL\_PROLIFERATION |
| 0.0 | 0.0 | EXTRACELLULAR\_SPACE |
| 3.7335965E-5 | 0.013592186 | NETPATH\_KIT\_RECEPTOR\_PATHWAY\_UP |
| 2.0661078E-6 | 0.0036572856 | NCI\_AMB2\_NEUTROPHILS\_PATHWAY |
| 7.433137E-4 | 0.16848037 | KEGG\_VIRAL\_MYOCARDITIS |
| 4.0434443E-6 | 0.015176189 | INFLAMMATORY\_RESPONSE |
| 0.010256637 | 0.024691083 | WOUND\_HEALING |
| 0.0 | 0.0 | CYTOKINE\_ACTIVITY |
| 2.5522509E-6 | 0.021367086 | KEGG\_NOD\_LIKE\_RECEPTOR\_SIGNALING\_PATHWAY |
| 5.8961764E-4 | 1.962291E-4 | NCI\_NFAT\_TFPATHWAY |
| 0.0 | 0.003802986 | NCI\_IL23PATHWAY |
| 0.009618234 | 0.13351512 | REACTOME\_G\_ALPHA\_I\_SIGNALLING\_EVENTS |
| 0.0 | 0.0038984409 | NCI\_IL12\_2PATHWAY |
| 0.002985779 | 0.022957014 | COAGULATION |
| 0.0 | 0.018020527 | KEGG\_TYPE\_I\_DIABETES\_MELLITUS |
| 2.3349554E-4 | 0.024385693 | ANTI\_APOPTOSIS |
| 2.2250392E-6 | 0.023901414 | BEHAVIOR |
| 0.0 | 5.743034E-5 | NETPATH\_IL\_3\_PATHWAY\_UP |
| 0.0 | 0.035359737 | KEGG\_ALLOGRAFT\_REJECTION |
| 0.0028893442 | 0.024227347 | BLOOD\_COAGULATION |
| 0.0 | 0.036907617 | KEGG\_GRAFT\_VERSUS\_HOST\_DISEASE |
| 0.0 | 0.0 | REACTOME\_PEPTIDE\_LIGAND\_BINDING\_RECEPTORS |
| 0.0 | 0.0 | NETPATH\_IL\_7\_PATHWAY\_UP |
| 0.002180355 | 0.06693496 | KEGG\_T\_CELL\_RECEPTOR\_SIGNALING\_PATHWAY |
| 3.6631514E-5 | 0.1161462 | RESPONSE\_TO\_BIOTIC\_STIMULUS |
| 2.9922942E-6 | 0.04655676 | KEGG\_JAK\_STAT\_SIGNALING\_PATHWAY |
| 3.0991619E-6 | 0.16195871 | KEGG\_LEISHMANIA\_INFECTION |
| 3.2139455E-6 | 0.04213685 | LOCOMOTORY\_BEHAVIOR |
| 4.1946248E-4 | 0.13047208 | POSITIVE\_REGULATION\_OF\_CELL\_PROLIFERATION |
| 0.0 | 3.546737E-5 | CHEMOKINE\_ACTIVITY |
| 0.0 | 0.0 | REACTOME\_CHEMOKINE\_RECEPTORS\_BIND\_CHEMOKINES |
| 0.0032991811 | 0.049544487 | KEGG\_CELL\_ADHESION\_MOLECULES\_CAMS |
| 2.160868E-5 | 5.0409563E-4 | NCI\_CD8TCRDOWNSTREAMPATHWAY |
| 0.013854358 | 0.07934187 | REACTOME\_FORMATION\_OF\_FIBRIN\_CLOT\_CLOTTING\_CASCADE |
| 0.004631088 | 0.18435535 | KEGG\_MAPK\_SIGNALING\_PATHWAY |
| 0.0074755205 | 0.08021661 | NCI\_LYSOPHOSPHOLIPID\_PATHWAY |
| 3.6304154E-5 | 0.13481797 | KEGG\_PRION\_DISEASES |
| 0.0 | 0.0012329832 | BIOCARTA\_CYTOKINE\_PATHWAY |
| 0.0 | 2.8777304E-5 | CHEMOKINE\_RECEPTOR\_BINDING |
| 6.802776E-4 | 0.09974431 | KEGG\_AUTOIMMUNE\_THYROID\_DISEASE |
| 1.6542599E-5 | 0.030804053 | KEGG\_RIG\_I\_LIKE\_RECEPTOR\_SIGNALING\_PATHWAY |
| 0.0 | 5.405209E-5 | NETPATH\_IL\_6\_PATHWAY\_UP |
| 0.006149904 | 0.04704689 | NCI\_SYNDECAN\_4\_PATHWAY |
| 0.0 | 5.4974487E-5 | BIOCARTA\_INFLAM\_PATHWAY |
| 9.398161E-5 | 0.07672392 | NCI\_IL27PATHWAY |
| 0.0 | 6.563468E-5 | G\_PROTEIN\_COUPLED\_RECEPTOR\_BINDING |
| 1.3989894E-4 | 0.15817112 | KEGG\_CYTOSOLIC\_DNA\_SENSING\_PATHWAY |
| 2.7992428E-6 | 0.13609284 | BIOCARTA\_IL1R\_PATHWAY |
| 0.034454864 | 0.07889986 | NCI\_DISSOLUTION\_OF\_FIBRIN\_CLOT |
| 0.0023013013 | 0.14520223 | BIOCARTA\_LAIR\_PATHWAY |
| 1.4610786E-4 | 0.08491679 | KEGG\_INTESTINAL\_IMMUNE\_NETWORK\_FOR\_IGA\_PRODUCTION |
| 6.656398E-5 | 0.16764152 | RESPONSE\_TO\_OTHER\_ORGANISM |
| 5.803015E-6 | 0.031688064 | NCI\_ATF2\_PATHWAY |
| 4.1889373E-4 | 0.13597828 | HUMORAL\_IMMUNE\_RESPONSE |
| 2.2835927E-6 | 0.08502608 | RESPONSE\_TO\_VIRUS |
| 1.6570099E-4 | 0.08045883 | BIOCARTA\_TH1TH2\_PATHWAY |
| 0.0138140945 | 0.14453259 | POSITIVE\_REGULATION\_OF\_MULTICELLULAR\_ORGANISMAL\_PROCESS |
| 0.002267632 | 0.09923221 | KEGG\_EPITHELIAL\_CELL\_SIGNALING\_IN\_HELICOBACTER\_PYLORI\_INFECTION |
| 0.024859793 | 0.1820822 | POSITIVE\_REGULATION\_OF\_CYTOKINE\_PRODUCTION |
| 0.002537575 | 0.0878706 | NETPATH\_IL\_9\_PATHWAY\_UP |
| 5.2014195E-5 | 0.021979617 | NETPATH\_IL\_1\_PATHWAY |
| 0.005572529 | 0.11735649 | BIOCARTA\_FREE\_PATHWAY |
| 0.009271151 | 0.07972296 | BIOCARTA\_FIBRINOLYSIS\_PATHWAY |
| 0.0035063634 | 0.16531254 | SA\_MMP\_CYTOKINE\_CONNECTION |
| 0.009347258 | 0.0019129739 | KEGG\_ECM\_RECEPTOR\_INTERACTION |
| 0.025966307 | 0.122265436 | REACTOME\_INTEGRIN\_CELL\_SURFACE\_INTERACTIONS |
| 0.002013036 | 0.042549185 | BIOCARTA\_DC\_PATHWAY |
| 9.2645256E-5 | 0.09709073 | BIOCARTA\_TNFR2\_PATHWAY |
| 0.03350293 | 0.19104198 | NCI\_S1P\_S1P1\_PATHWAY |
| 3.7597125E-5 | 0.016043266 | BIOCARTA\_STEM\_PATHWAY |
| 5.676862E-6 | 0.13711655 | INTERLEUKIN\_BINDING |
| 0.014559986 | 0.13966201 | KEGG\_HYPERTROPHIC\_CARDIOMYOPATHY\_HCM |
| 3.2052063E-4 | 0.14347613 | NCI\_CHEMOKINE\_RECEPTORS\_BIND\_CHEMOKINES |
| 0.044520505 | 0.07953078 | NETPATH\_HEDGEHOG\_PATHWAY\_UP |
| 0.010782168 | 0.080586836 | REACTOME\_PLATELET\_ADHESION\_TO\_EXPOSED\_COLLAGEN |
| 1.6053132E-4 | 0.035758696 | BIOCARTA\_IL17\_PATHWAY |
| 0.0074842023 | 0.17548726 | NCI\_TCRCALCIUMPATHWAY |
| 0.0035081438 | 0.046208695 | BIOCARTA\_CDMAC\_PATHWAY |
| 0.01743104 | 0.14575876 | REACTOME\_AMINO\_ACID\_TRANSPORT\_ACROSS\_THE\_PLASMA\_MEMBRANE |
| 0.020787546 | 0.13548742 | CYTOKINE\_METABOLIC\_PROCESS |
| 0.011636542 | 0.18952166 | REACTOME\_TRAF6\_MEDIATED\_INDUCTION\_OF\_THE\_ANTIVIRAL\_CYTOKINE\_IFN\_ALPHA\_BETA\_CASCADE |
| 0.0127873905 | 0.13899016 | POSITIVE\_REGULATION\_OF\_IMMUNE\_RESPONSE |
| 1.14572584E-4 | 0.030180147 | NCI\_CD40\_PATHWAY |
| 0.022878889 | 0.13338058 | REGULATION\_OF\_IMMUNE\_RESPONSE |
| 0.0067488668 | 0.16922034 | BIOCARTA\_ASBCELL\_PATHWAY |
| 8.233354E-5 | 1.621935E-5 | HEMATOPOIETIN\_INTERFERON\_CLASSD200\_DOMAIN\_CYTOKINE\_RECEPTOR\_BINDING |
| 0.04540201 | 0.09227219 | NCI\_NFKAPPABCANONICALPATHWAY |
| 0.039900873 | 0.19854371 | DELAYED\_RECTIFIER\_POTASSIUM\_CHANNEL\_ACTIVITY |
| 0.050513387 | 0.18283321 | NEGATIVE\_REGULATION\_OF\_CYTOKINE\_BIOSYNTHETIC\_PROCESS |
| 0.0012447159 | 0.05717223 | INTERFERON\_GAMMA\_PRODUCTION |
| 2.6295918E-6 | 0.046159845 | BIOCARTA\_NKT\_PATHWAY |
| 0.0 | 5.1121856E-4 | GROWTH\_FACTOR\_ACTIVITY |
| 0.051811732 | 0.0835772 | APICOLATERAL\_PLASMA\_MEMBRANE |
| 0.006041763 | 0.15994835 | NCI\_APOPTOTIC\_CLEAVAGE\_OF\_CELL\_ADHESION\_\_PROTEINS |
| 3.5301655E-5 | 0.012676984 | BIOCARTA\_ERYTH\_PATHWAY |
| 0.008763204 | 0.13552004 | REACTOME\_HUMAN\_TAK1\_ACTIVATES\_NFKB\_BY\_PHOSPHORYLATION\_AND\_ACTIVATION\_OF\_IKKS\_COMPLEX |
| 0.0036709863 | 0.020797646 | BIOCARTA\_CARDIACEGF\_PATHWAY |
| 0.011540983 | 0.13259868 | POSITIVE\_REGULATION\_OF\_PEPTIDYL\_TYROSINE\_PHOSPHORYLATION |
| 0.019340528 | 0.18329455 | CORUM\_NFKB1-NFKB2-REL-RELA-RELB\_COMPLEX |
| 0.013864454 | 0.0033732688 | HORMONE\_SECRETION |
| 0.008676701 | 0.15889207 | REACTOME\_APOPTOTIC\_CLEAVAGE\_OF\_CELL\_ADHESION\_PROTEINS |
| 3.231262E-4 | 0.030155556 | CORUM\_CHUK-NFKB2-REL-IKBKG-SPAG9-NFKB1-NFKBIE-COPB2-TNIP1-NFKBIA-RELA-TNIP2\_COMPLEX |
| 0.0016041094 | 0.08494553 | INTERFERON\_GAMMA\_BIOSYNTHETIC\_PROCESS |
| 0.0038455338 | 0.16680549 | NCI\_DOWNSTREAM\_TCR\_SIGNALING |
| 0.002521776 | 0.15058221 | NETPATH\_WNT\_PATHWAY\_DOWN |
| 0.0028675264 | 0.116786905 | REGULATION\_OF\_INTERFERON\_GAMMA\_BIOSYNTHETIC\_PROCESS |
| 0.020191431 | 0.009836641 | REGULATION\_OF\_HORMONE\_SECRETION |
| 0.02637748 | 0.0879128 | KEGG\_GLYCOSAMINOGLYCAN\_BIOSYNTHESIS\_KERATAN\_SULFATE |
| 0.19500217 | 0.14158979 | HEMOSTASIS |
| 0.035804305 | 0.026547316 | NCI\_ERBB\_NETWORK\_PATHWAY |
| 0.098667026 | 0.081610605 | KEGG\_GLYCOSPHINGOLIPID\_BIOSYNTHESIS\_LACTO\_AND\_NEOLACTO\_SERIES |
| 0.13496065 | 0.010121106 | NCI\_HIF1\_TFPATHWAY |
| 0.118211694 | 0.13599485 | SKELETAL\_DEVELOPMENT |
| 0.059418194 | 0.09914407 | NCI\_INTEGRIN\_CELL\_SURFACE\_INTERACTIONS |
| 0.06268975 | 0.12023649 | POSITIVE\_REGULATION\_OF\_RESPONSE\_TO\_STIMULUS |
| 0.088080995 | 0.19187756 | GLYCOLIPID\_METABOLIC\_PROCESS |
| 0.059923522 | 0.07949442 | INTERCALATED\_DISC |
| 2.466239E-4 | 0.03884668 | CORUM\_TNF-ALPHA/NF-KAPPA\_B\_SIGNALING\_COMPLEX\_CHUK\_KPNA3\_NFKB2\_NFKBIB\_REL\_IKBKG\_\_NFKB1\_NFKBIE\_RELB\_\_NFKBIA\_RELA\_TNIP2 |
| 0.13157527 | 0.12361986 | CALCIUM\_MEDIATED\_SIGNALING |
| 0.18123625 | 0.119487554 | REACTOME\_PLATELET\_DEGRANULATION |
| 0.06449941 | 0.01092913 | NEGATIVE\_REGULATION\_OF\_CELLULAR\_BIOSYNTHETIC\_PROCESS |
| 2.3260359E-4 | 0.03866104 | CORUM\_TNF-ALPHA/NF-KAPPA\_B\_SIGNALING\_COMPLEX\_RPL6\_RPL30\_RPS13\_CHUK\_DDX3X\_NFKB2\_NFKBIB\_REL\_IKBKG\_NFKB1\_MAP3K8\_RELB\_GLG1\_NFKBIA\_RELA\_TNIP2\_\_GTF2I |
| 0.055568002 | 0.13624333 | ST\_TUMOR\_NECROSIS\_FACTOR\_PATHWAY |
| 0.065832324 | 0.04927809 | NCI\_HIF2PATHWAY |
| 0.0032938984 | 0.1325342 | INACTIVATION\_OF\_MAPK\_ACTIVITY |
| 0.098546006 | 0.13522168 | BIOCARTA\_PLATELETAPP\_PATHWAY |
| 0.015908092 | 0.11939131 | ANTIGEN\_BINDING |
| 0.05288436 | 0.080938905 | APICAL\_JUNCTION\_COMPLEX |
| 0.013748358 | 0.025169797 | REACTOME\_UNFOLDED\_PROTEIN\_RESPONSE |
| 0.01133793 | 0.11784687 | BILE\_ACID\_METABOLIC\_PROCESS |
| 0.12818907 | 0.11545533 | KEGG\_ADIPOCYTOKINE\_SIGNALING\_PATHWAY |
| 0.06107344 | 0.17295587 | NEGATIVE\_REGULATION\_OF\_CELL\_PROLIFERATION |
| 0.19393124 | 0.117594175 | REGULATION\_OF\_BODY\_FLUID\_LEVELS |
| 0.15807852 | 0.020148115 | REACTOME\_STEROID\_HORMONES |
| 0.011306912 | 7.06835E-5 | HORMONE\_ACTIVITY |
| 0.100472346 | 0.16336772 | NCI\_TCRRASPATHWAY |
| 0.095168166 | 0.0021141812 | GENERATION\_OF\_A\_SIGNAL\_INVOLVED\_IN\_CELL\_CELL\_SIGNALING |
| 0.18593393 | 0.13267855 | BIOCARTA\_DEATH\_PATHWAY |
| 0.16686626 | 0.18264878 | ACTIVATION\_OF\_IMMUNE\_RESPONSE |
| 0.06264309 | 0.031240948 | NCI\_IL12\_STAT4PATHWAY |
| 0.066257045 | 0.16470632 | NETPATH\_WNT\_PATHWAY\_UP |
| 0.095360726 | 0.1470429 | REACTOME\_G\_ALPHA\_S\_SIGNALLING\_EVENTS |
| 0.08269128 | 0.12842031 | REGULATION\_OF\_MITOTIC\_CELL\_CYCLE |
