# Supplementary material for: Computational approaches for discovery of common immunomodulators in fungal infections: towards broad-spectrum immunotherapeutic interventions
Source: BMC Microbiol. 2013 Oct 7;13:224. doi: 10.1186/1471-2180-13-224 (PMC3853472; doi:10.1186/1471-2180-13-224)
Supplement: Additional file 1 — Details of up- and down- regulated biclusters. [file 1471-2180-13-224-S1.zip › 2013-kidane-bmc/details-of-biclusters/upreg-biclust-30.html]

**BICLUSTER\_ID** : UPREG-30  
**PATHOGENS** /2/ : a. alternata,a. fumigatus  
**KNOWN DRUG TARGETS** /17/ : PLAT, CCL2, CCND1, CASP7, CAPN1, PLAU, ADCY7, TGM2, FGF2, GPRC5A, PIK3R1, NP, CALM1, MET, SERPINE1, IL8, PLAUR  

| Gene Set | Leading Edge Genes |
| --- | --- |
| NETPATH EGFR1 PATHWAY UP | EMP1, EREG, ITGA3, HPCAL1, CCND1, PLAU, TCOF1, SOX9, AKAP12, SDC4, COTL1, TGM2, IER3, GPRC5A, NP, TGFA, TNFAIP3, MET, TFPI2, PLAUR |
| LOCOMOTORY BEHAVIOR | FOSL1, PLAU, CXCL1, CCL2, IL8, PLAUR, FGF2 |
| NCI DISSOLUTION OF FIBRIN CLOT | PLAU, SERPINE1, PLAT, PLAUR |
| KEGG CHEMOKINE SIGNALING PATHWAY | CXCL1, PIK3R1, CCL2, RELA, CXCL5, NFKBIA, ADCY7, IL8, GRK5, CXCL2, PXN |
| KEGG CYTOKINE CYTOKINE RECEPTOR INTERACTION | CCL2, VEGFC, OSMR, IL22RA1, BMPR2, CXCL2, IL17RA, PDGFA, CXCL1, VEGFB, LIFR, TNFRSF11A, CXCL5, FAS, MET, IL8 |
| KEGG APOPTOSIS | BIRC3, PIK3R3, IRAK1, PIK3R1, TRAF2, BIRC2, CAPN1, CASP7, RELA, MAP3K14, NFKBIA, FAS, ENDOD1, BID, CFLAR, MYD88 |
| NCI NFAT TFPATHWAY | FOSL1, CALM1, JUNB, IL8 |
| CHEMOKINE ACTIVITY | CXCL1, CCL2, IL8 |
| VIRAL GENOME REPLICATION | TNIP1, CCL2, IL8 |
| KEGG NOD LIKE RECEPTOR SIGNALING PATHWAY | BIRC3, CXCL1, CCL2, BIRC2, RELA, NFKBIA, TNFAIP3, IL8, CXCL2 |
| CHEMOKINE RECEPTOR BINDING | CXCL1, CCL2, IL8 |
| REACTOME CHEMOKINE RECEPTORS BIND CHEMOKINES | CXCL1, CCL2, IL8 |
| KEGG RIG I LIKE RECEPTOR SIGNALING PATHWAY | ISG15, TRAF3, TRAF2, DDX3Y, IKBKE, RELA, DDX3X, TRIM25, NFKBIA, IL8, DDX58 |
| BIOCARTA STEM PATHWAY | IL8 |
| LEUKOCYTE CHEMOTAXIS | IL8 |
| REGULATION OF RESPONSE TO STIMULUS | EREG, TRAF2, IL8 |
| NETPATH IL 7 PATHWAY UP | CXCL5, CXCL1, MCL1, TRAF3, IL8, CXCL2 |
| BIOCARTA INFLAM PATHWAY | PDGFA, IL8 |
| CELL MIGRATION | VEGFC, PPAP2B, SPHK1, IL8 |
| POSITIVE REGULATION OF CELL PROLIFERATION | SPHK1, PDGFA, EREG, VEGFB, CD47, CAPN1, RHOG, TGFA, FOSL1, TBX3, TBRG4 |
| BIOCARTA CYTOKINE PATHWAY | IL8 |
| BIOCARTA LYM PATHWAY | IL8 |
| POSITIVE REGULATION OF RESPONSE TO STIMULUS | EREG, TRAF2, IL8 |
| POSITIVE REGULATION OF DEFENSE RESPONSE | EREG |
| BIOCARTA NFKB PATHWAY | MAP3K14, NFKBIA, IRAK1, TNFAIP3, MYD88, RELA |
| REGULATION OF PEPTIDYL TYROSINE PHOSPHORYLATION |  |
| NCI TNFPATHWAY | SQSTM1, BIRC3, TNFAIP3, MAP4K4, TRAF2, BIRC2, RELA |
| NCI CD40 PATHWAY | MAP3K14, NFKBIA, BIRC3, TNFAIP3, TRAF2, TRAF3, BIRC2, RELA |
| CELLULAR DEFENSE RESPONSE | FOSL1, MICB |
| KEGG CYTOSOLIC DNA SENSING PATHWAY | POLR1C, NFKBIA, IRF3, PYCARD, IKBKE, DDX58, RELA |
| POSITIVE REGULATION OF PEPTIDYL TYROSINE PHOSPHORYLATION |  |
| ST TUMOR NECROSIS FACTOR PATHWAY | NFKBIA, BIRC3, TNFAIP3, NFKBIE, TRAF2, BIRC2, CFLAR |
| REGULATION OF JAK STAT CASCADE | HGS |

| Color legend | | | | | | | | | | | |
| --- | --- | --- | --- | --- | --- | --- | --- | --- | --- | --- | --- |
| q-value | 1 | 0.2 | 0.05 | 0.01 | 0.001 | 0.0001 |
| Color |  | |  |  |  | |

TABLE OF Q-VALUES

| alternaria alternata beas2b | aspergillus fumigatus cluture filtrates a549 | Gene Set |
| --- | --- | --- |
| 0.0482491 | 8.3157216E-4 | NETPATH\_EGFR1\_PATHWAY\_UP |
| 0.0 | 0.064572826 | LOCOMOTORY\_BEHAVIOR |
| 0.059493493 | 0.11790499 | NCI\_DISSOLUTION\_OF\_FIBRIN\_CLOT |
| 0.029972985 | 0.10088094 | KEGG\_CHEMOKINE\_SIGNALING\_PATHWAY |
| 4.8502272E-5 | 0.050987493 | KEGG\_CYTOKINE\_CYTOKINE\_RECEPTOR\_INTERACTION |
| 0.14210635 | 0.189176 | KEGG\_APOPTOSIS |
| 0.0028159413 | 0.14430721 | NCI\_NFAT\_TFPATHWAY |
| 0.0 | 0.13061193 | CHEMOKINE\_ACTIVITY |
| 0.12211427 | 0.097631186 | VIRAL\_GENOME\_REPLICATION |
| 0.0015941259 | 0.04048064 | KEGG\_NOD\_LIKE\_RECEPTOR\_SIGNALING\_PATHWAY |
| 0.0 | 0.12956315 | CHEMOKINE\_RECEPTOR\_BINDING |
| 0.0 | 0.0 | REACTOME\_CHEMOKINE\_RECEPTORS\_BIND\_CHEMOKINES |
| 0.0 | 0.13297741 | KEGG\_RIG\_I\_LIKE\_RECEPTOR\_SIGNALING\_PATHWAY |
| 0.048412904 | 0.014165326 | BIOCARTA\_STEM\_PATHWAY |
| 0.036746003 | 0.10609597 | LEUKOCYTE\_CHEMOTAXIS |
| 0.039942924 | 0.14550571 | REGULATION\_OF\_RESPONSE\_TO\_STIMULUS |
| 0.08906518 | 0.0386872 | NETPATH\_IL\_7\_PATHWAY\_UP |
| 0.024269812 | 0.0013572491 | BIOCARTA\_INFLAM\_PATHWAY |
| 0.0551639 | 0.1551768 | CELL\_MIGRATION |
| 0.02215129 | 0.13124275 | POSITIVE\_REGULATION\_OF\_CELL\_PROLIFERATION |
| 0.06687076 | 5.6428078E-5 | BIOCARTA\_CYTOKINE\_PATHWAY |
| 0.02800103 | 0.1187666 | BIOCARTA\_LYM\_PATHWAY |
| 0.06788228 | 0.08907495 | POSITIVE\_REGULATION\_OF\_RESPONSE\_TO\_STIMULUS |
| 0.032808315 | 0.08528607 | POSITIVE\_REGULATION\_OF\_DEFENSE\_RESPONSE |
| 0.10728212 | 0.09701282 | BIOCARTA\_NFKB\_PATHWAY |
| 0.06715995 | 0.04849489 | REGULATION\_OF\_PEPTIDYL\_TYROSINE\_PHOSPHORYLATION |
| 0.15428899 | 0.1284036 | NCI\_TNFPATHWAY |
| 0.039924555 | 0.08014807 | NCI\_CD40\_PATHWAY |
| 0.15413347 | 0.08863518 | CELLULAR\_DEFENSE\_RESPONSE |
| 4.208835E-4 | 0.13053004 | KEGG\_CYTOSOLIC\_DNA\_SENSING\_PATHWAY |
| 0.15405402 | 0.13979301 | POSITIVE\_REGULATION\_OF\_PEPTIDYL\_TYROSINE\_PHOSPHORYLATION |
| 0.11110564 | 0.0015568112 | ST\_TUMOR\_NECROSIS\_FACTOR\_PATHWAY |
| 0.09628014 | 0.15667865 | REGULATION\_OF\_JAK\_STAT\_CASCADE |
