# Supplementary material for: Computational approaches for discovery of common immunomodulators in fungal infections: towards broad-spectrum immunotherapeutic interventions
Source: BMC Microbiol. 2013 Oct 7;13:224. doi: 10.1186/1471-2180-13-224 (PMC3853472; doi:10.1186/1471-2180-13-224)
Supplement: Additional file 1 — Details of up- and down- regulated biclusters. [file 1471-2180-13-224-S1.zip › 2013-kidane-bmc/details-of-biclusters/upreg-biclust-10.html]

**BICLUSTER\_ID** : UPREG-10  
**PATHOGENS** /1/ : a. fumigatus  
**KNOWN DRUG TARGETS** /39/ : ATP2C1, PPP2CB, AURKA, PTK2, TGFB1, EGLN1, MAP2K1, EGFR, NFKB1, GPRC5A, IGF1R, PPARG, GSK3B, PDPK1, SERPINE1, MET, ACVR1, MAPK1, EPHA2, JUN, CCL2, CASP3, CASP7, PLAU, ADCY7, FGF2, LAMC1, CSK, PARP1, PPP3R1, HDAC2, RPS6KA5, PTGS2, PPAT, CREB1, MAPKAPK2, IL8, GRB2, PLAUR  

| Gene Set | Leading Edge Genes |
| --- | --- |
| KEGG PATHWAYS IN CANCER | TRAF4, ITGA3, NRAS, NFKBIA, E2F3, PTK2, DVL1, TGFB1, ITGA2, EGLN1, MAP2K1, EGFR, PDGFA, MSH2, IGF1R, TRAF3, PPARG, GSK3B, MET, TRAF6, MAPK1, CCNE1, RALA, JUN, ITGA6, BIRC2, DVL3, SOS1, CASP3, TGFB2, RELA, E2F1, VEGFC, PIK3CA, GLI2, BRCA2, PIAS2, COL4A2, CUL2, RALGDS, NKX3-1, FGF2, ARNT2, LAMC1, BIRC3, CCNE2, TGFA, SMAD3, MITF, DAPK3, HDAC2, PTGS2, IL8, SMAD4, GRB2 |
| KEGG FOCAL ADHESION | ARHGAP5, DIAPH1, ACTN4, ITGA3, VASP, PAK2, PTK2, ITGA2, EGFR, ITGA5, MAP2K1, FLNA, PDGFA, IGF1R, CAV2, GSK3B, PDPK1, MET, ZYX, MAPK1, CAV1, JUN, ITGA6, SOS1, BIRC2, VEGFC, CAPN2, PIK3CA, COL4A2, CRK, LAMC1, CCND3, BIRC3, PXN, GRB2 |
| BIOCARTA MAPK PATHWAY | MAPK7, JUN, MAP3K12, RELA, TGFB2, MAP4K2, MEF2A, PAK2, MAP3K14, NFKBIA, MAP4K3, MAP3K2, TGFB1, MAP2K1, NFKB1, MAP2K4, MAP3K7, RPS6KA5, MAPKAPK2, DAXX, CREB1, MAP4K4, MAPK1, GRB2 |
| KEGG MAPK SIGNALING PATHWAY | MAPK7, BDNF, DUSP6, PAK2, RASA2, RRAS2, RELB, EGFR, MAP2K1, PDGFA, MAP3K7, TRAF6, DUSP4, DUSP10, JUN, SOS1, TGFB2, RELA, CASP3, DUSP14, MAP3K14, PPP3CC, MAP3K2, FGF2, DUSP5, DUSP3, PPP3CB, PPP3R1, RPS6KA5, CRKL, DAXX, MAPKAPK2, GRB2, DUSP1 |
| KEGG CHEMOKINE SIGNALING PATHWAY | CCL2, SOS1, RELA, CXCL3, NFKBIA, PIK3CA, PTK2, ADCY7, CXCL2, MAP2K1, NFKB1, CXCL1, CSK, GSK3B, CXCL5, CRKL, IL8, PXN, GRB2 |
| NCI LYSOPHOSPHOLIPID PATHWAY | NFKB1, JUN, HBEGF, GSK3B, CASP3, RELA, NFKBIA, PTK2, ADCY7, IL8, PXN, EGFR |
| REACTOME TOLL LIKE RECEPTOR 3 CASCADE | PPP2R5D, DUSP4, MAPK7, JUN, NFKB2, PPP2CB, DUSP6, MEF2A, RELA, NFKBIA, MAP2K1, NFKB1, DUSP3, MAP2K4, MAP3K7, RPS6KA5, PPP2R1B, MAPKAPK2, CREB1, MAPK1, TICAM1, TRAF6 |
| KEGG EPITHELIAL CELL SIGNALING IN HELICOBACTER PYLORI INFECTION | NFKB1, CXCL1, JUN, CSK, HBEGF, CASP3, RELA, TJP1, ATP6V0A2, MAP3K14, NFKBIA, ADAM10, MET, IL8, EGFR |
| NETPATH TNF ALPHA PATHWAY | TXLNA, TRAF4, NFKBIA, PTK2, BTRC, RELB, CFLAR, NFKB1, FLNA, TNIP1, TRAF3, MAP3K7IP2, CSNK2A2, GSK3B, SMARCA4, TNFAIP3, RASAL2, TRAF6, CAV1, KTN1, NFKB2, NFKBIE, BIRC2, RELA, CASP3, CASP7, MAP3K14, MARK2, MAP3K2, REL, BIRC3, PSMD12, DPF2, PPP6C, NSMAF, KPNA3, DDX3X, HDAC2, RPS6KA5 |
| KEGG CYTOKINE CYTOKINE RECEPTOR INTERACTION | TNFRSF10B, CCL2, TGFB2, CLCF1, CXCL3, VEGFC, OSMR, TGFB1, CXCL2, IL10RB, EGFR, TNFRSF21, PDGFA, CXCL1, TNFRSF12A, CXCL5, TNFSF9, MET, ACVR1, IL8 |
| KEGG CHRONIC MYELOID LEUKEMIA | SOS1, RELA, TGFB2, E2F1, NFKBIA, E2F3, PIK3CA, CBLB, TGFB1, MAP2K1, NFKB1, SMAD3, HDAC2, CRKL, SMAD4, MAPK1, GRB2 |
| REACTOME MAP KINASES ACTIVATION IN TLR CASCADE | DUSP4, MAPK7, JUN, PPP2CB, DUSP6, MEF2A, MAP2K1, DUSP3, MAP2K4, MAP3K7, RPS6KA5, PPP2R1B, MAPKAPK2, TRAF6, TICAM1, MAPK1 |
| NETPATH EGFR1 PATHWAY UP | EMP1, EREG, ITGA3, DUSP6, PHLDA2, SLC20A1, GPRC5A, TAGLN, EHD1, TNFAIP3, MET, TFPI2, SFN, BUB1B, CAV1, DUSP4, PPP1R10, TGFB2, PLAU, PTHLH, AKAP12, SDC4, IER3, ROR1, NAV3, PHLDA1, PLK2, SPRY2, PPAT, PTGS2, DUSP1, PLAUR |
| NCI MET PATHWAY | JUN, SOS1, PDPK1, PIK3CA, PTK2, MET, PXN, GRB2, MAP2K1 |
| BIOCARTA MET PATHWAY | JUN, SOS1, PIK3CA, CRKL, MET, PTK2, PXN, GRB2, MAP2K1 |
| REACTOME TRAF6 MEDIATED INDUCTION OF THE ANTIVIRAL CYTOKINE IFN ALPHA BETA CASCADE | NFKB1, DUSP4, MAPK7, JUN, PPP2CB, DUSP3, DUSP6, MAP3K7, MEF2A, RELA, NFKBIA, RPS6KA5, TRAF6, TICAM1, MAP2K1 |
| LOCOMOTORY BEHAVIOR | CXCL1, CCL2, TGFB2, PLAU, FOSL1, IL8, PLAUR, FGF2 |
| KEGG T CELL RECEPTOR SIGNALING PATHWAY | JUN, NFKBIE, SOS1, PAK2, RELA, MAP3K14, NFKBIA, PIK3CA, CBLB, PPP3CC, MAP2K1, PPP3CB, GSK3B, MALT1, MAP3K7, PPP3R1, GRB2 |
| BIOCARTA FAS PATHWAY | LMNB1, JUN, PARP1, MAP2K4, LMNA, MAP3K7, CASP3, CASP7, PAK2, FAS, DAXX, LMNB2, CFLAR |
| KEGG B CELL RECEPTOR SIGNALING PATHWAY | JUN, NFKBIE, PPP3CB, MALT1, GSK3B, SOS1, RELA, NFKBIA, PPP3R1, PIK3CA, PPP3CC, MAP2K1 |
| NCI NFAT TFPATHWAY | FOSL1, GATA3, PTGS2, JUN, JUNB, IL8, PPARG |
| BIOCARTA HIVNEF PATHWAY | LMNB1, BIRC2, LMNA, CASP3, CASP7, PAK2, RELA, MAP3K14, NFKBIA, PTK2, CFLAR, BIRC3, PARP1, PSEN2, FAS, PSEN1, DAXX, LMNB2 |
| BIOCARTA AGR PATHWAY | JUN, PAK2, GIT2, PTK2, DVL1, PXN, MAPK1, EGFR |
| ST B CELL ANTIGEN RECEPTOR | NFKBIA, PIK3CA, CSK, NFKBIE, SOS1, MAP2K1, GRB2 |
| KEGG SMALL CELL LUNG CANCER | CCNE1, TRAF4, ITGA6, ITGA3, BIRC2, RELA, NFKBIA, E2F3, PIK3CA, PTK2, ITGA2, LAMC1, BIRC3, TRAF3, CCNE2, PTGS2, TRAF6 |
| NCI DISSOLUTION OF FIBRIN CLOT | PLAU, SERPINE1, PLAUR |
| BIOCARTA FCER1 PATHWAY | JUN, PPP3CB, SOS1, PAK2, PIK3CA, PPP3CC, GRB2, MAP2K1 |
| BIOCARTA NGF PATHWAY | PIK3CA, JUN, SOS1, GRB2, MAP2K1 |
| ST T CELL SIGNAL TRANSDUCTION | NFKBIA, CSK, NFKBIE, MAPK1, SOS1, GRB2, PAK2 |
| BIOCARTA TCR PATHWAY | JUN, PPP3CB, SOS1, RELA, NFKBIA, PIK3CA, PPP3CC, MAP2K1, GRB2 |
| BIOCARTA TNFR1 PATHWAY | JUN, PARP1, MAP2K4, LMNA, CASP3, MAP3K7, PAK2, LMNB2 |
| BIOCARTA GLEEVEC PATHWAY | PIK3CA, CRKL, JUN, SOS1, MAP2K1, GRB2 |
| NCI PDGFRAPATHWAY | CAV1, PIK3CA, PDGFA, CRKL, JUN, SHB, SOS1, GRB2 |
| VIRAL INFECTIOUS CYCLE | TNIP1, CCL2, IL8 |
| BIOCARTA CDMAC PATHWAY | NFKBIA, JUN, MAP2K1, RELA |
| NETPATH IL 7 PATHWAY UP | CXCL1, JUN, MCL1, TRAF3, CXCL3, CXCL5, CXCL2, IL8 |
| CHEMOKINE ACTIVITY | CXCL3, CXCL1, CCL2, IL8 |
| VIRAL GENOME REPLICATION | TNIP1, CCL2, IL8 |
| KEGG NOD LIKE RECEPTOR SIGNALING PATHWAY | BIRC3, CXCL1, CCL2, BIRC2, RELA, NFKBIA, TNFAIP3, CXCL2, IL8, TRAF6 |
| CHEMOKINE RECEPTOR BINDING | CXCL3, CXCL1, CCL2, IL8 |
| REACTOME CHEMOKINE RECEPTORS BIND CHEMOKINES | CXCL3, CXCL1, CCL2, IL8 |
| KEGG RIG I LIKE RECEPTOR SIGNALING PATHWAY | NFKB1, TRAF3, DDX3Y, MAP3K7, RELA, DDX3X, NFKBIA, IL8, TRAF6, DDX58 |
| BIOCARTA INFLAM PATHWAY | PDGFA, TGFB1, IL8, TGFB2 |
| POSITIVE REGULATION OF CELL PROLIFERATION | LAMC1, PDGFA, EREG, CDC7, TGFB2, TBX3, TGFA, PTHLH, FOSL1, TBRG4, HOXC10, EGFR |
| NCI AVB3 OPN PATHWAY | PLAU, MAP3K14, NFKBIA, PIK3CA, JUN, PIP5K1A, RELA |
| ST TUMOR NECROSIS FACTOR PATHWAY | BIRC3, JUN, NFKBIE, BIRC2, CASP3, MAP3K7, NFKBIA, TNFAIP3, CFLAR |
| POSITIVE REGULATION OF PHOSPHATE METABOLIC PROCESS | CCND3, EREG, TGFB1, CLCF1, EGFR |
| ECTODERM DEVELOPMENT | PTHLH, ATP2C1, EMP1, FST, SMURF1, TGFB2 |
| BIOCARTA STEM PATHWAY | IL8 |
| NCI PLK1 PATHWAY | FBXO5, BUB1B, ECT2, KIF2A, CDC20, BUB1, CDC25C, PLK1, AURKA, PRC1, CENPE, BTRC, GORASP1 |
| NCI TNFPATHWAY | NFKB1, CAV1, BIRC3, MAP3K7IP2, BIRC2, MAP3K7, MAP4K2, PRKCI, RELA, NSMAF, SQSTM1, MAP4K3, TNFAIP3, MAP4K4 |
| ADAPTIVE IMMUNE RESPONSE | SOCS5, TGFB1, MALT1, TRAF6, MAP3K7 |
| POSITIVE REGULATION OF PHOSPHORYLATION | CCND3, EREG, CLCF1, EGFR |
| CORUM CHUK-NFKB2-REL-IKBKG-SPAG9-NFKB1-NFKBIE-COPB2-TNIP1-NFKBIA-RELA-TNIP2 COMPLEX | NFKB1, NFKBIA, SPAG9, REL, TNIP1, NFKBIE, RELA |
| BIOCARTA CYTOKINE PATHWAY | IL8 |
| POSITIVE REGULATION OF MULTICELLULAR ORGANISMAL PROCESS | SOCS5, FST, EREG, TGFB1, TRAF6, MALT1, TGFB2 |
| POSITIVE REGULATION OF RESPONSE TO STIMULUS | EREG, IL8, MALT1, TRAF6, MAP3K7, TGFB2 |
| ADAPTIVE IMMUNE RESPONSE GO 0002460 | SOCS5, TGFB1, MALT1, TRAF6, MAP3K7 |
| PEPTIDYL TYROSINE PHOSPHORYLATION | NF2, LYN, EGFR, CLCF1 |
| BIOCARTA ERYTH PATHWAY | TGFB1, TGFB2 |
| REGULATION OF IMMUNE SYSTEM PROCESS | SOCS5, EREG, TGFB1, TRAF6, MALT1, TGFB2 |
| REGULATION OF PEPTIDYL TYROSINE PHOSPHORYLATION | CLCF1, EGFR |
| NCI CD40 PATHWAY | BIRC3, JUN, TRAF3, BIRC2, RELA, MAP3K14, NFKBIA, TNFAIP3, TRAF6 |
| POSITIVE REGULATION OF IMMUNE SYSTEM PROCESS | SOCS5, EREG, TGFB1, TRAF6, MALT1, MAP3K7, TGFB2 |
| REGULATION OF RESPONSE TO STIMULUS | EREG, IL8, MALT1, TRAF6, MAP3K7, TGFB2 |
| BIOCARTA IL17 PATHWAY | IL8 |
| PEPTIDYL TYROSINE MODIFICATION | NF2, TPST2, LYN, EGFR, CLCF1 |
| NCI EPHRINA EPHAPATHWAY | EPHA2 |
| VIRAL REPRODUCTIVE PROCESS |  |
| BIOCARTA NFKB PATHWAY | MAP3K14, NFKBIA, IRAK1, TNFAIP3, TRAF6, MAP3K7, RELA |
| NETPATH IL 1 PATHWAY |  |
| KEGG HYPERTROPHIC CARDIOMYOPATHY HCM |  |
| NCI CXCR4 PATHWAY |  |
| TISSUE MORPHOGENESIS | TBX3, TGFB2 |
| BIOCARTA LYM PATHWAY |  |
| REGULATION OF CYTOKINE PRODUCTION | SMAD3, EREG, SMAD4, MALT1, TRAF6, MAP3K7, TGFB2 |
| BIOCARTA CD40 PATHWAY | MAP3K14, NFKBIA, TNFAIP3, TRAF3, TRAF6, DUSP1, RELA |
| NETPATH ANDROGEN RECEPTOR PATHWAY |  |
| POSITIVE REGULATION OF IMMUNE RESPONSE | EREG, MALT1, TRAF6, MAP3K7, TGFB2 |
| EPIDERMIS DEVELOPMENT | EMP1, FST, TGFB2 |
| NEGATIVE REGULATION OF IMMUNE SYSTEM PROCESS |  |
| SIG CD40PATHWAYMAP | NFKBIA, PIK3CA, NFKBIE, TRAF3, TRAF6, DUSP1 |
| CORUM TNF-ALPHA/NF-KAPPA B SIGNALING COMPLEX CHUK KPNA3 NFKB2 NFKBIB REL IKBKG NFKB1 NFKBIE RELB NFKBIA RELA TNIP2 |  |
| LEUKOCYTE CHEMOTAXIS |  |
| REGULATION OF IMMUNE RESPONSE | EREG, MALT1, TRAF6, MAP3K7, TGFB2 |
| KEGG HEMATOPOIETIC CELL LINEAGE |  |
| CORUM TNF-ALPHA/NF-KAPPA B SIGNALING COMPLEX RPL6 RPL30 RPS13 CHUK DDX3X NFKB2 NFKBIB REL IKBKG NFKB1 MAP3K8 RELB GLG1 NFKBIA RELA TNIP2 GTF2I |  |
| BIOCARTA TNFR2 PATHWAY | MAP3K14, NFKBIA, TNFAIP3, TRAF3, DUSP1, RELA |
| POSITIVE REGULATION OF JNK ACTIVITY |  |
| PRODUCTION OF MOLECULAR MEDIATOR OF IMMUNE RESPONSE |  |
| NEGATIVE REGULATION OF MULTICELLULAR ORGANISMAL PROCESS |  |

| Color legend | | | | | | | | | | | |
| --- | --- | --- | --- | --- | --- | --- | --- | --- | --- | --- | --- |
| q-value | 1 | 0.2 | 0.05 | 0.01 | 0.001 | 0.0001 |
| Color |  | |  |  |  | |

TABLE OF Q-VALUES

| aspergillus fumigatus conidia a549 | aspergillus fumigatus cluture filtrates a549 | Gene Set |
| --- | --- | --- |
| 0.021361278 | 0.088504635 | KEGG\_PATHWAYS\_IN\_CANCER |
| 0.027411686 | 0.010211144 | KEGG\_FOCAL\_ADHESION |
| 0.022544334 | 0.091613196 | BIOCARTA\_MAPK\_PATHWAY |
| 0.003983041 | 0.120740704 | KEGG\_MAPK\_SIGNALING\_PATHWAY |
| 0.028497338 | 0.10088094 | KEGG\_CHEMOKINE\_SIGNALING\_PATHWAY |
| 0.009436423 | 0.084783584 | NCI\_LYSOPHOSPHOLIPID\_PATHWAY |
| 0.008573098 | 0.08971636 | REACTOME\_TOLL\_LIKE\_RECEPTOR\_3\_CASCADE |
| 0.044559825 | 0.08678882 | KEGG\_EPITHELIAL\_CELL\_SIGNALING\_IN\_HELICOBACTER\_PYLORI\_INFECTION |
| 0.109485276 | 0.08667545 | NETPATH\_TNF\_ALPHA\_PATHWAY |
| 2.5672338E-5 | 0.050987493 | KEGG\_CYTOKINE\_CYTOKINE\_RECEPTOR\_INTERACTION |
| 0.08917099 | 0.17137855 | KEGG\_CHRONIC\_MYELOID\_LEUKEMIA |
| 0.012436739 | 0.1536006 | REACTOME\_MAP\_KINASES\_ACTIVATION\_IN\_TLR\_CASCADE |
| 2.5547526E-5 | 8.3157216E-4 | NETPATH\_EGFR1\_PATHWAY\_UP |
| 0.085733145 | 0.13343371 | NCI\_MET\_PATHWAY |
| 0.05619789 | 0.11091478 | BIOCARTA\_MET\_PATHWAY |
| 0.008140157 | 0.10125844 | REACTOME\_TRAF6\_MEDIATED\_INDUCTION\_OF\_THE\_ANTIVIRAL\_CYTOKINE\_IFN\_ALPHA\_BETA\_CASCADE |
| 2.2720385E-5 | 0.064572826 | LOCOMOTORY\_BEHAVIOR |
| 0.0076145586 | 0.13845338 | KEGG\_T\_CELL\_RECEPTOR\_SIGNALING\_PATHWAY |
| 0.04840773 | 0.059661794 | BIOCARTA\_FAS\_PATHWAY |
| 0.049602754 | 0.13446142 | KEGG\_B\_CELL\_RECEPTOR\_SIGNALING\_PATHWAY |
| 1.4434879E-4 | 0.14430721 | NCI\_NFAT\_TFPATHWAY |
| 0.029508216 | 0.08937066 | BIOCARTA\_HIVNEF\_PATHWAY |
| 0.10652275 | 0.09843292 | BIOCARTA\_AGR\_PATHWAY |
| 0.044324104 | 0.074949 | ST\_B\_CELL\_ANTIGEN\_RECEPTOR |
| 0.02137055 | 0.13113259 | KEGG\_SMALL\_CELL\_LUNG\_CANCER |
| 0.01466968 | 0.11790499 | NCI\_DISSOLUTION\_OF\_FIBRIN\_CLOT |
| 0.093007945 | 0.04818694 | BIOCARTA\_FCER1\_PATHWAY |
| 0.09468745 | 0.131192 | BIOCARTA\_NGF\_PATHWAY |
| 0.01957019 | 0.11773539 | ST\_T\_CELL\_SIGNAL\_TRANSDUCTION |
| 0.019508844 | 0.153501 | BIOCARTA\_TCR\_PATHWAY |
| 0.11049662 | 0.048329744 | BIOCARTA\_TNFR1\_PATHWAY |
| 0.0937287 | 0.14519274 | BIOCARTA\_GLEEVEC\_PATHWAY |
| 0.002269441 | 0.13217598 | NCI\_PDGFRAPATHWAY |
| 0.100959316 | 0.08900039 | VIRAL\_INFECTIOUS\_CYCLE |
| 0.050761063 | 0.19136539 | BIOCARTA\_CDMAC\_PATHWAY |
| 3.284682E-5 | 0.0386872 | NETPATH\_IL\_7\_PATHWAY\_UP |
| 2.8400484E-5 | 0.13061193 | CHEMOKINE\_ACTIVITY |
| 0.06791781 | 0.097631186 | VIRAL\_GENOME\_REPLICATION |
| 7.929928E-5 | 0.04048064 | KEGG\_NOD\_LIKE\_RECEPTOR\_SIGNALING\_PATHWAY |
| 0.0 | 0.12956315 | CHEMOKINE\_RECEPTOR\_BINDING |
| 0.0 | 0.0 | REACTOME\_CHEMOKINE\_RECEPTORS\_BIND\_CHEMOKINES |
| 0.08271712 | 0.13297741 | KEGG\_RIG\_I\_LIKE\_RECEPTOR\_SIGNALING\_PATHWAY |
| 0.01884623 | 0.0013572491 | BIOCARTA\_INFLAM\_PATHWAY |
| 0.08752097 | 0.13124275 | POSITIVE\_REGULATION\_OF\_CELL\_PROLIFERATION |
| 0.058076452 | 0.14755891 | NCI\_AVB3\_OPN\_PATHWAY |
| 0.0033291676 | 0.0015568112 | ST\_TUMOR\_NECROSIS\_FACTOR\_PATHWAY |
| 0.049802355 | 0.09764229 | POSITIVE\_REGULATION\_OF\_PHOSPHATE\_METABOLIC\_PROCESS |
| 0.025122251 | 0.12919737 | ECTODERM\_DEVELOPMENT |
| 0.02162187 | 0.014165326 | BIOCARTA\_STEM\_PATHWAY |
| 0.09106791 | 0.13886009 | NCI\_PLK1\_PATHWAY |
| 0.112507604 | 0.1284036 | NCI\_TNFPATHWAY |
| 0.026384508 | 0.05319312 | ADAPTIVE\_IMMUNE\_RESPONSE |
| 0.039465003 | 0.12935251 | POSITIVE\_REGULATION\_OF\_PHOSPHORYLATION |
| 0.02733804 | 0.17240939 | CORUM\_CHUK-NFKB2-REL-IKBKG-SPAG9-NFKB1-NFKBIE-COPB2-TNIP1-NFKBIA-RELA-TNIP2\_COMPLEX |
| 0.007446698 | 5.6428078E-5 | BIOCARTA\_CYTOKINE\_PATHWAY |
| 0.037143324 | 0.08996527 | POSITIVE\_REGULATION\_OF\_MULTICELLULAR\_ORGANISMAL\_PROCESS |
| 0.01471888 | 0.08907495 | POSITIVE\_REGULATION\_OF\_RESPONSE\_TO\_STIMULUS |
| 0.038137298 | 0.0014208762 | ADAPTIVE\_IMMUNE\_RESPONSE\_GO\_0002460 |
| 0.0517779 | 0.13131265 | PEPTIDYL\_TYROSINE\_PHOSPHORYLATION |
| 0.09741977 | 0.14564127 | BIOCARTA\_ERYTH\_PATHWAY |
| 0.09243208 | 0.12798534 | REGULATION\_OF\_IMMUNE\_SYSTEM\_PROCESS |
| 0.08275426 | 0.04849489 | REGULATION\_OF\_PEPTIDYL\_TYROSINE\_PHOSPHORYLATION |
| 3.4622008E-5 | 0.08014807 | NCI\_CD40\_PATHWAY |
| 0.039703723 | 0.13785362 | POSITIVE\_REGULATION\_OF\_IMMUNE\_SYSTEM\_PROCESS |
| 0.11097098 | 0.14550571 | REGULATION\_OF\_RESPONSE\_TO\_STIMULUS |
| 0.045313723 | 0.001628699 | BIOCARTA\_IL17\_PATHWAY |
| 0.04039933 | 0.07342324 | PEPTIDYL\_TYROSINE\_MODIFICATION |
| 0.091415726 | 0.13178568 | NCI\_EPHRINA\_EPHAPATHWAY |
| 0.11872337 | 0.08926697 | VIRAL\_REPRODUCTIVE\_PROCESS |
| 0.07733991 | 0.09701282 | BIOCARTA\_NFKB\_PATHWAY |
| 0.17969474 | 0.13162808 | NETPATH\_IL\_1\_PATHWAY |
| 0.1734655 | 0.10251222 | KEGG\_HYPERTROPHIC\_CARDIOMYOPATHY\_HCM |
| 0.19418271 | 0.16484222 | NCI\_CXCR4\_PATHWAY |
| 0.06765847 | 0.14050001 | TISSUE\_MORPHOGENESIS |
| 0.12109292 | 0.1187666 | BIOCARTA\_LYM\_PATHWAY |
| 0.11095213 | 0.1531107 | REGULATION\_OF\_CYTOKINE\_PRODUCTION |
| 0.019903738 | 0.13739029 | BIOCARTA\_CD40\_PATHWAY |
| 0.15857422 | 0.14368615 | NETPATH\_ANDROGEN\_RECEPTOR\_PATHWAY |
| 0.030143488 | 0.12950608 | POSITIVE\_REGULATION\_OF\_IMMUNE\_RESPONSE |
| 0.042364307 | 0.12961179 | EPIDERMIS\_DEVELOPMENT |
| 0.18911812 | 0.07195826 | NEGATIVE\_REGULATION\_OF\_IMMUNE\_SYSTEM\_PROCESS |
| 0.042247143 | 0.15359046 | SIG\_CD40PATHWAYMAP |
| 0.03207342 | 0.15513363 | CORUM\_TNF-ALPHA/NF-KAPPA\_B\_SIGNALING\_COMPLEX\_CHUK\_KPNA3\_NFKB2\_NFKBIB\_REL\_IKBKG\_\_NFKB1\_NFKBIE\_RELB\_\_NFKBIA\_RELA\_TNIP2 |
| 0.16236398 | 0.10609597 | LEUKOCYTE\_CHEMOTAXIS |
| 0.013679031 | 0.09040961 | REGULATION\_OF\_IMMUNE\_RESPONSE |
| 0.14205706 | 0.17998308 | KEGG\_HEMATOPOIETIC\_CELL\_LINEAGE |
| 0.07160611 | 0.13089982 | CORUM\_TNF-ALPHA/NF-KAPPA\_B\_SIGNALING\_COMPLEX\_RPL6\_RPL30\_RPS13\_CHUK\_DDX3X\_NFKB2\_NFKBIB\_REL\_IKBKG\_NFKB1\_MAP3K8\_RELB\_GLG1\_NFKBIA\_RELA\_TNIP2\_\_GTF2I |
| 0.011185336 | 0.092164285 | BIOCARTA\_TNFR2\_PATHWAY |
| 0.18629128 | 0.1406128 | POSITIVE\_REGULATION\_OF\_JNK\_ACTIVITY |
| 0.18744552 | 0.08596912 | PRODUCTION\_OF\_MOLECULAR\_MEDIATOR\_OF\_IMMUNE\_RESPONSE |
| 0.17614537 | 0.048869196 | NEGATIVE\_REGULATION\_OF\_MULTICELLULAR\_ORGANISMAL\_PROCESS |
