# Supplementary material for: Computational approaches for discovery of common immunomodulators in fungal infections: towards broad-spectrum immunotherapeutic interventions
Source: BMC Microbiol. 2013 Oct 7;13:224. doi: 10.1186/1471-2180-13-224 (PMC3853472; doi:10.1186/1471-2180-13-224)
Supplement: Additional file 1 — Details of up- and down- regulated biclusters. [file 1471-2180-13-224-S1.zip › 2013-kidane-bmc/details-of-biclusters/upreg-biclust-12.html]

**BICLUSTER\_ID** : UPREG-12  
**PATHOGENS** /2/ : a. alternata,a. fumigatus  
**KNOWN DRUG TARGETS** /17/ : PDE4D, CCL2, PLAU, ASNS, PIM1, CD55, GPRC5A, GARS, PPIF, TFPI, TOP1, IL6, MET, SERPINE1, ICAM1, IL8, PLAUR  

| Gene Set | Leading Edge Genes |
| --- | --- |
| NETPATH IL 2 PATHWAY UP | DUSP4, ZNF267, PFKFB3, IL1RAP, RELA, SLC2A3, PMAIP1, ASNS, PIM1, FOS, CREM, BHLHB2, IER3, CFLAR, IFIT1, KLF6, DUSP5, LIF, GARS, TOP1, ETS2, VEGF, NFIL3, SERPINE1, ICAM1, IL8, IRF1, SLC7A5, CHSY1, PLAUR |
| NETPATH IL 5 PATHWAY UP | BIRC3, DUSP5, PPIF, CCL2, NFKBIE, RELA, NFIL3, EGR1, ICAM1, IL8, CD55, PIM1, RELB, IER3, IER2 |
| RESPONSE TO EXTERNAL STIMULUS | IL1RAP, CXCL1, EREG, CCL2, CEBPB, TFPI, RELA, PLAU, FOSL1, CXCL5, SERPINE1, IL8, RIPK2, FOS, CXCL2, PLAUR |
| KEGG CYTOKINE CYTOKINE RECEPTOR INTERACTION | PDGFB, LIF, IL1RAP, CXCL1, CCL2, IL15, CXCL5, IL6, MET, CXCL2, IL8 |
| LOCOMOTORY BEHAVIOR | FOSL1, PLAU, CXCL5, CXCL1, CCL2, IL8, CXCL2, PLAUR |
| NETPATH EGFR1 PATHWAY UP | EMP1, EREG, ITGA3, PLAU, EGR1, AKAP12, SDC4, IER3, GPRC5A, LIF, VEGF, CXCL5, MET, TNFAIP3, TFPI2, MYC, PLAUR |
| BEHAVIOR | CXCL1, CCL2, FOSB, PLAU, FOSL1, CXCL5, CXCL2, IL8, PLAUR |
| NETPATH IL 1 PATHWAY UP | PDE4D, NFKBIE, CCL2, RELA, FOSL1, NFKBIA, CXCL2, SOD2, BIRC3, CXCL1, LIF, CXCL5, SERPINE1, ZFP36, IL8, MYC |
| NETPATH IL 3 PATHWAY UP | NFIL3, MCL1, CCL2, PIM1, IL8, FOS |
| IMMUNE SYSTEM PROCESS | NFIL3, SEMA4D, IL6, EREG, CCL2, CEBPB, IL8, IL15, BNIP3 |
| RESPONSE TO WOUNDING | CXCL1, EREG, IL1RAP, CEBPB, TFPI, RELA, SERPINE1, IL8, RIPK2, FOS, CXCL2 |
| REACTOME CLASS A1 RHODOPSIN LIKE RECEPTORS | CXCL5, CXCL1, CCL2, CXCL2, IL8 |
| REACTOME PEPTIDE LIGAND BINDING RECEPTORS | CXCL5, CXCL1, CCL2, CXCL2, IL8 |
| CYTOKINE ACTIVITY | CXCL5, CXCL1, CCL2, IL8, CXCL2 |
| NETPATH IL 4 PATHWAY DOWN | NFKBIA, CCL2, CXCL2, IL8, IRF1, DDIT4 |
| CHEMOKINE ACTIVITY | CXCL5, CXCL1, CCL2, CXCL2, IL8 |
| RECEPTOR BINDING | CXCL1, EREG, CCL2, HMGA1, TGFA, EFNA1, CXCL5, IL8, CXCL2 |
| NCI DISSOLUTION OF FIBRIN CLOT | SERPINE1, PLAUR |
| KEGG JAK STAT SIGNALING PATHWAY | IL6, LIF, PIM1, MYC, SPRY1, IL15 |
| NETPATH KIT RECEPTOR PATHWAY UP | DUSP4, MCL1, JUNB, CCL2, RELA, VEGF, EGR1, PIM1, FOS, IER3 |
| G PROTEIN COUPLED RECEPTOR BINDING | CXCL5, CXCL1, CCL2, CXCL2, IL8 |
| KEGG NOD LIKE RECEPTOR SIGNALING PATHWAY | BIRC3, CXCL1, CCL2, BIRC2, RELA, NFKBIA, TNFAIP3, RIPK2, CXCL2, IL8 |
| REACTOME GPCR LIGAND BINDING | CXCL5, CXCL1, CCL2, IL8, CXCL2 |
| EXTRACELLULAR SPACE | TNFAIP2, CXCL1, EREG, CCL2, CXCL2, IL8 |
| CHEMOKINE RECEPTOR BINDING | CXCL5, CXCL1, CCL2, CXCL2, IL8 |
| REACTOME CHEMOKINE RECEPTORS BIND CHEMOKINES | CXCL5, CXCL1, CCL2, CXCL2, IL8 |
| KEGG RIG I LIKE RECEPTOR SIGNALING PATHWAY | NFKBIA, DDX3X, IL8, RELA |
| NCI NFAT TFPATHWAY | FOSL1, EGR1, JUNB, IL8, FOS |
| BIOCARTA STEM PATHWAY | IL8 |
| NETPATH IL 6 PATHWAY UP | CXCL1, MCL1, JUNB, CEBPB, SLC2A3, MAFF, ZFP36, IRF1, PIM1, BHLHB2 |
| IMMUNE RESPONSE | NFIL3, EREG, CEBPB, CCL2, BNIP3, IL15 |
| CALCIUM MEDIATED SIGNALING | IL8 |
| NCI IL23PATHWAY | NFKBIA, CXCL1, CCL2, RELA |
| BIOCARTA INFLAM PATHWAY | IL8, IL15 |
| NETPATH IL 7 PATHWAY UP | CXCL5, CXCL1, MCL1, IL8, CXCL2 |
| POSITIVE REGULATION OF CELL PROLIFERATION | TGFA, FOSL1, CXCL5, IL6, LIF, EREG, MYC, IL15 |
| REACTOME G ALPHA I SIGNALLING EVENTS | CXCL5, CXCL1, IL8, CXCL2 |
| BIOCARTA CYTOKINE PATHWAY | IL8, IL15 |
| DEFENSE RESPONSE | TNIP1, CXCL1, EREG, IL1RAP, CEBPB, RELA, FOSL1, VEZF1, IL8, RIPK2, FOS, CXCL2, BNIP3 |
| INFLAMMATORY RESPONSE | CXCL1, CEBPB, IL8, CXCL2, RIPK2, RELA |
| KEGG TOLL LIKE RECEPTOR SIGNALING PATHWAY | NFKBIA, IL8, FOS, MAP3K8, TICAM1, RELA |
| POSITIVE REGULATION OF RESPONSE TO STIMULUS | EREG, IL8 |
| NETPATH TNF ALPHA PATHWAY DOWN | IFIT5, MAP3K14, NFKBIA, EGR1, SDC4, CXCL2, BHLHB2, IER3, KLF6, CXCL1, NR4A1, KLF10, PPP1R15A, JUNB, FOSB, VEGF, DDX3X, TNFAIP2, MAFF, TNFAIP3, ZFP36, IRF1 |
| NCI CHEMOKINE RECEPTORS BIND CHEMOKINES |  |
| KEGG LEISHMANIA INFECTION | NFKBIA, FOS, RELA |
| CELL CELL SIGNALING |  |
| DEVELOPMENTAL GROWTH |  |
| NETPATH HEDGEHOG PATHWAY UP | VEGF, NR4A1 |
| NCI CD40 PATHWAY | MAP3K14, NFKBIA, BIRC3, TNFAIP3, MYC, BIRC2, RELA |
| BIOCARTA DEATH PATHWAY |  |
| EXTRACELLULAR REGION PART |  |
| BIOCARTA LAIR PATHWAY |  |
| ST TUMOR NECROSIS FACTOR PATHWAY | NFKBIA, BIRC3, TNFAIP3, NFKBIE, BIRC2, CFLAR |
| NCI IL12 2PATHWAY | RELB, FOS, RIPK2, RELA |

| Color legend | | | | | | | | | | | |
| --- | --- | --- | --- | --- | --- | --- | --- | --- | --- | --- | --- |
| q-value | 1 | 0.2 | 0.05 | 0.01 | 0.001 | 0.0001 |
| Color |  | |  |  |  | |

TABLE OF Q-VALUES

| aspergillus fumigatus conidia a549 | alternaria alternata beas2b | aspergillus fumigatus dendritic | Gene Set |
| --- | --- | --- | --- |
| 2.9144692E-4 | 0.0 | 0.002894239 | NETPATH\_IL\_2\_PATHWAY\_UP |
| 1.0097658E-4 | 0.0012581189 | 0.0033619835 | NETPATH\_IL\_5\_PATHWAY\_UP |
| 6.960372E-4 | 1.715488E-5 | 0.024326107 | RESPONSE\_TO\_EXTERNAL\_STIMULUS |
| 2.5672338E-5 | 4.8502272E-5 | 0.0 | KEGG\_CYTOKINE\_CYTOKINE\_RECEPTOR\_INTERACTION |
| 2.2720385E-5 | 0.0 | 0.04213685 | LOCOMOTORY\_BEHAVIOR |
| 2.5547526E-5 | 0.0482491 | 3.9014107E-5 | NETPATH\_EGFR1\_PATHWAY\_UP |
| 1.642341E-5 | 1.2214923E-5 | 0.023901414 | BEHAVIOR |
| 1.5328516E-5 | 0.0014360275 | 0.0 | NETPATH\_IL\_1\_PATHWAY\_UP |
| 2.8740968E-5 | 0.0027465345 | 5.743034E-5 | NETPATH\_IL\_3\_PATHWAY\_UP |
| 0.09567344 | 4.841327E-5 | 0.17588222 | IMMUNE\_SYSTEM\_PROCESS |
| 0.0013995847 | 6.9193807E-6 | 0.0047411146 | RESPONSE\_TO\_WOUNDING |
| 0.0015525026 | 7.181068E-4 | 0.003579989 | REACTOME\_CLASS\_A1\_RHODOPSIN\_LIKE\_RECEPTORS |
| 1.9160645E-5 | 0.0 | 0.0 | REACTOME\_PEPTIDE\_LIGAND\_BINDING\_RECEPTORS |
| 2.0902522E-5 | 0.0 | 0.0 | CYTOKINE\_ACTIVITY |
| 8.290948E-4 | 0.0 | 0.0027640385 | NETPATH\_IL\_4\_PATHWAY\_DOWN |
| 2.8400484E-5 | 0.0 | 3.546737E-5 | CHEMOKINE\_ACTIVITY |
| 0.096030585 | 0.00988802 | 0.009376848 | RECEPTOR\_BINDING |
| 0.01466968 | 0.059493493 | 0.07889986 | NCI\_DISSOLUTION\_OF\_FIBRIN\_CLOT |
| 1.768675E-5 | 0.13215114 | 0.04655676 | KEGG\_JAK\_STAT\_SIGNALING\_PATHWAY |
| 0.003725802 | 0.026568137 | 0.013592186 | NETPATH\_KIT\_RECEPTOR\_PATHWAY\_UP |
| 1.8933655E-5 | 9.6749085E-5 | 6.563468E-5 | G\_PROTEIN\_COUPLED\_RECEPTOR\_BINDING |
| 7.929928E-5 | 0.0015941259 | 0.021367086 | KEGG\_NOD\_LIKE\_RECEPTOR\_SIGNALING\_PATHWAY |
| 0.06493268 | 0.023852112 | 0.023223832 | REACTOME\_GPCR\_LIGAND\_BINDING |
| 0.07053614 | 0.0027369289 | 0.0 | EXTRACELLULAR\_SPACE |
| 0.0 | 0.0 | 2.8777304E-5 | CHEMOKINE\_RECEPTOR\_BINDING |
| 0.0 | 0.0 | 0.0 | REACTOME\_CHEMOKINE\_RECEPTORS\_BIND\_CHEMOKINES |
| 0.08271712 | 0.0 | 0.030804053 | KEGG\_RIG\_I\_LIKE\_RECEPTOR\_SIGNALING\_PATHWAY |
| 1.4434879E-4 | 0.0028159413 | 1.962291E-4 | NCI\_NFAT\_TFPATHWAY |
| 0.02162187 | 0.048412904 | 0.016043266 | BIOCARTA\_STEM\_PATHWAY |
| 3.6444217E-5 | 0.004494796 | 5.405209E-5 | NETPATH\_IL\_6\_PATHWAY\_UP |
| 0.039666753 | 4.6192636E-5 | 0.030914374 | IMMUNE\_RESPONSE |
| 0.020051014 | 0.056881335 | 0.12361986 | CALCIUM\_MEDIATED\_SIGNALING |
| 1.4370484E-5 | 6.501296E-4 | 0.003802986 | NCI\_IL23PATHWAY |
| 0.01884623 | 0.024269812 | 5.4974487E-5 | BIOCARTA\_INFLAM\_PATHWAY |
| 3.284682E-5 | 0.08906518 | 0.0 | NETPATH\_IL\_7\_PATHWAY\_UP |
| 0.08752097 | 0.02215129 | 0.13047208 | POSITIVE\_REGULATION\_OF\_CELL\_PROLIFERATION |
| 0.017489815 | 4.58652E-5 | 0.13351512 | REACTOME\_G\_ALPHA\_I\_SIGNALLING\_EVENTS |
| 0.007446698 | 0.06687076 | 0.0012329832 | BIOCARTA\_CYTOKINE\_PATHWAY |
| 0.005702261 | 0.0 | 0.106237434 | DEFENSE\_RESPONSE |
| 6.0851693E-5 | 0.0 | 0.015176189 | INFLAMMATORY\_RESPONSE |
| 0.017372232 | 2.0601391E-4 | 0.091590926 | KEGG\_TOLL\_LIKE\_RECEPTOR\_SIGNALING\_PATHWAY |
| 0.01471888 | 0.06788228 | 0.12023649 | POSITIVE\_REGULATION\_OF\_RESPONSE\_TO\_STIMULUS |
| 0.0 | 0.09455213 | 0.0036943294 | NETPATH\_TNF\_ALPHA\_PATHWAY\_DOWN |
| 0.009898863 | 0.09035489 | 0.14347613 | NCI\_CHEMOKINE\_RECEPTORS\_BIND\_CHEMOKINES |
| 0.044578478 | 0.031793345 | 0.16195871 | KEGG\_LEISHMANIA\_INFECTION |
| 0.12902527 | 0.068371326 | 0.009947655 | CELL\_CELL\_SIGNALING |
| 0.19798702 | 0.17261824 | 0.18452302 | DEVELOPMENTAL\_GROWTH |
| 0.006852322 | 0.1578012 | 0.07953078 | NETPATH\_HEDGEHOG\_PATHWAY\_UP |
| 3.4622008E-5 | 0.039924555 | 0.030180147 | NCI\_CD40\_PATHWAY |
| 0.13951814 | 0.036749456 | 0.13267855 | BIOCARTA\_DEATH\_PATHWAY |
| 0.13260934 | 0.18083072 | 6.125904E-5 | EXTRACELLULAR\_REGION\_PART |
| 0.118695535 | 0.022626787 | 0.14520223 | BIOCARTA\_LAIR\_PATHWAY |
| 0.0033291676 | 0.11110564 | 0.13624333 | ST\_TUMOR\_NECROSIS\_FACTOR\_PATHWAY |
| 0.0054974956 | 0.0064716395 | 0.0038984409 | NCI\_IL12\_2PATHWAY |
