# Supplementary material for: Computational approaches for discovery of common immunomodulators in fungal infections: towards broad-spectrum immunotherapeutic interventions
Source: BMC Microbiol. 2013 Oct 7;13:224. doi: 10.1186/1471-2180-13-224 (PMC3853472; doi:10.1186/1471-2180-13-224)
Supplement: Additional file 1 — Details of up- and down- regulated biclusters. [file 1471-2180-13-224-S1.zip › 2013-kidane-bmc/details-of-biclusters/upreg-biclust-14.html]

**BICLUSTER\_ID** : UPREG-14  
**PATHOGENS** /2/ : a. alternata,s. chartarum  
**KNOWN DRUG TARGETS** /34/ : MMP2, SLC7A11, TGFB1, CXCL10, CFB, HDAC9, PPIF, JAK2, IL1B, F3, CASP1, CCL2, CD40, PLAU, ADCY7, CCL5, ADORA2B, CD55, PIM1, C1S, ADA, SLC16A3, IFNGR1, CSK, NP, TAP1, CTSS, CTSB, KCNN4, IL6, PSAP, PTGER2, ABP1, PLAUR  

| Gene Set | Leading Edge Genes |
| --- | --- |
| KEGG CYTOKINE CYTOKINE RECEPTOR INTERACTION | IL1RAP, CCL2, CD40, OSMR, CXCL16, CCL5, CXCL2, IL17RA, CXCL11, CXCL10, CXCL1, CCL28, IFNGR1, TNFRSF11A, CXCL5, FAS, IL6, TNFRSF14, IL1B |
| RESPONSE TO EXTERNAL STIMULUS | HDAC9, ELF3, CXCL1, CCL2, CD40, MDK, PLAU, CXCL5, C3AR1, CCL5, FOS, CXCL2, CXCL11, CXCL10, PLAUR |
| REACTOME SIGNALING IN IMMUNE SYSTEM | CD200, CD40, MAP3K8, IKBKE, TLR4, SLC7A11, NFKBIA, FOS, PDCD1LG2, CFB, IFITM1, C1S, ICOSLG, SLC16A3, CSK, CD274, TBK1, TNFRSF14, CD14, TICAM1 |
| NETPATH IL 5 PATHWAY UP | BIRC3, PPIF, TRAF3, CCL2, MAX, NFKBIE, TLR4, NFIL3, EGR1, IL1B, RELB, PIM1, CD55, ABP1, CASP4, MYD88 |
| NETPATH IL 2 PATHWAY UP | ADAM19, SLC2A3, TLR4, TRAFD1, PLAU, PMAIP1, PRKCD, IFI44, CTSH, PIM1, FOS, CREM, BHLHB2, B4GALT5, KLF6, IRF5, NFIL3, TNFRSF11A, KCNAB1, FAS, IL1B, CASP1, PBEF1, PTGER2, MYC, PLAUR, MX1 |
| IMMUNE SYSTEM PROCESS | ICOSLG, HDAC9, IL27RA, CCL2, TRAF2, CD274, TLR4, NFIL3, CTSS, SEMA4D, IL6, HCLS1, CCL5, LAT2, MAFB |
| REACTOME CLASS A1 RHODOPSIN LIKE RECEPTORS | CXCL1, CCL2, CXCL5, CXCL16, C3AR1, CCL5, PTGER2, CXCL2, ADORA2B, CXCL10 |
| KEGG COMPLEMENT AND COAGULATION CASCADES | PLAU, C1S, F3, CFB, PLAUR |
| BEHAVIOR | PLAU, CXCL5, CXCL1, CCL5, CCL2, CXCL2, CXCL10, PLAUR |
| DEFENSE RESPONSE | TCIRG1, HDAC9, ELF3, TNIP1, CXCL1, LGALS3BP, KCNN4, C3AR1, CCL5, ADORA2B, FOS, CXCL2, CXCL10, MX1 |
| LOCOMOTORY BEHAVIOR | PLAU, CXCL5, CXCL1, CCL5, CCL2, CXCL2, CXCL11, CXCL10, PLAUR |
| KEGG CHEMOKINE SIGNALING PATHWAY | JAK2, CXCL1, CCL2, CXCL5, NFKBIA, PRKCD, STAT2, ADCY7, CXCL16, RAC2, CCL5, CXCL2, CXCL11, CXCL10 |
| REACTOME GPCR LIGAND BINDING | CXCL1, CCL2, CXCL5, CXCL16, CCL5, CXCL2, ADORA2B, PTGER2, CXCL10 |
| KEGG JAK STAT SIGNALING PATHWAY | OSMR, IL6, JAK2, IFNGR1, STAT2, PIM1, MYC |
| KEGG NOD LIKE RECEPTOR SIGNALING PATHWAY | BIRC3, CXCL1, CCL2, BIRC2, NFKBIA, TNFAIP3, IL1B, CASP1, CCL5, CXCL2 |
| RESPONSE TO WOUNDING | HDAC9, ELF3, CXCL1, CD40, MDK, C3AR1, CCL5, FOS, CXCL2, CXCL11, CXCL10 |
| EXTRACELLULAR SPACE | CXCL1, TGFBI, CCL2, MMP2, TNFAIP2, LGALS3BP, IL1B, PSAP, CXCL2 |
| KEGG TOLL LIKE RECEPTOR SIGNALING PATHWAY | CD40, MAP3K8, IKBKE, TLR4, CTSK, NFKBIA, CCL5, FOS, CXCL11, CXCL10, IRF5, TRAF3, TBK1, IL1B, CD14, TICAM1 |
| NETPATH IL 1 PATHWAY UP | MMP14, NFKBIE, CCL2, NFKBIA, PRKCD, PTGES, MT2A, CXCL2, SOD2, CFB, BMP1, BIRC3, CXCL1, NP, ST3GAL4, CXCL5, IL1B, PBEF1, ZFP36, MYC |
| KEGG PRIMARY IMMUNODEFICIENCY | ADA, TAP2, CD40, TAP1 |
| REACTOME PEPTIDE LIGAND BINDING RECEPTORS | CXCL5, CXCL1, C3AR1, CXCL16, CCL5, CCL2, CXCL2, CXCL10 |
| CYTOKINE ACTIVITY | GDF15, CXCL1, CCL2, CXCL5, CXCL16, CCL5, CXCL2, CXCL10, CXCL11 |
| CHEMOKINE ACTIVITY | CXCL1, CCL2, CXCL5, CXCL16, CCL5, CXCL2, CXCL10, CXCL11 |
| CHEMOKINE RECEPTOR BINDING | CXCL1, CCL2, CXCL5, CXCL16, CCL5, CXCL2, CXCL10, CXCL11 |
| KEGG INTESTINAL IMMUNE NETWORK FOR IGA PRODUCTION | ICOSLG, IL6, CCL28, TGFB1, CD40, ITGB7 |
| REACTOME CHEMOKINE RECEPTORS BIND CHEMOKINES | CXCL5, CXCL1, CCL5, CCL2, CXCL2, CXCL10 |
| NETPATH IL 3 PATHWAY UP | NFIL3, IL1B, CCL2, PIM1, FOS, MYC |
| IMMUNE RESPONSE | NFIL3, CTSS, CCL5, CCL2, TRAF2, CD274 |
| REACTOME G ALPHA I SIGNALLING EVENTS | CXCL5, CXCL1, C3AR1, ADCY7, CXCL16, CCL5, CXCL2, CXCL10 |
| G PROTEIN COUPLED RECEPTOR BINDING | CXCL1, CCL2, CXCL5, CXCL16, CCL5, CXCL2, CXCL10, CXCL11 |
| INFLAMMATORY RESPONSE | HDAC9, ELF3, CXCL1, CCL5, CXCL2, CXCL11, CXCL10 |
| KEGG ANTIGEN PROCESSING AND PRESENTATION | CTSB, CTSS, PSME2, TAP2, LGMN, IFI30, TAP1, PSME1 |
| KEGG GRAFT VERSUS HOST DISEASE | FAS, IL6, IL1B |
| REACTOME COMPLEMENT CASCADE | C1S, CFB |
| CATION HOMEOSTASIS | C3AR1, HFE, CCL5, GNA15, MT2A, CCL2, MYC |
| REACTOME INNATE IMMUNITY SIGNALING | C1S, IKBKE, TLR4, NFKBIA, CD14, FOS, TICAM1, CFB |
| CHEMICAL HOMEOSTASIS | HFE, C3AR1, CCL5, GNA15, MT2A, CCL2, MYC |
| CELLULAR CATION HOMEOSTASIS | C3AR1, HFE, CCL5, GNA15, MT2A, CCL2, MYC |
| KEGG LEISHMANIA INFECTION | NFKBIA, IL1B, JAK2, FOS, TLR4 |
| BIOCARTA COMP PATHWAY | C1S, CFB |
| CELLULAR DEFENSE RESPONSE | TCIRG1, LGALS3BP, CCL5, ADORA2B |
| ION HOMEOSTASIS | HFE, C3AR1, CCL5, GNA15, MT2A, CCL2, MYC |
| NCI IL12 2PATHWAY | IL1B, JAK2, RELB, FOS |
| CELLULAR HOMEOSTASIS | HFE, C3AR1, CCL5, GNA15, MT2A, CCL2, MYC |
| REACTOME INITIAL TRIGGERING OF COMPLEMENT | C1S, CFB |
| HUMORAL IMMUNE RESPONSE | CCL2 |
| BIOCARTA NO2IL12 PATHWAY | JAK2 |
| NETPATH IL 7 PATHWAY UP | CXCL5, CXCL1, LITAF, CCL5, TRAF3, CXCL2, MYC |
| RESPONSE TO VIRUS | IFI44, CCL5, BCL3 |
| BIOCARTA CLASSIC PATHWAY | C1S |
| NCI IMMUNOREGULATORY INTERACTIONS BETWEEN A LYMPHOID AND A NON LYMPHOID CELL | CD200, IFITM1, CD40 |
| BIOCARTA IL22BP PATHWAY | JAK2, STAT2 |
| NETPATH IL 6 PATHWAY UP | CXCL1, CEBPD, JUNB, BCL3, SLC2A3, MAFF, ZFP36, PIM1, BHLHB2, MX1, SBNO2 |
| RESPONSE TO OTHER ORGANISM | IFI44, CCL5, BCL3 |
| NCI CD40 PATHWAY | BIRC3, TRAF2, TRAF3, CD40, BIRC2, NFKBIA, TNFAIP3, MYC |
| MULTI ORGANISM PROCESS | MAFF, TNIP1, IFI44, CCL5, BCL3 |
| RESPONSE TO BIOTIC STIMULUS | DNAJA1, IFI44, CCL5, BCL3 |
| REACTOME IMMUNOREGULATORY INTERACTIONS BETWEEN A LYMPHOID AND A NON LYMPHOID CELL | CD200, IFITM1, CD40 |
| BIOCARTA CASPASE PATHWAY | BIRC3, CASP1, BIRC2, CASP4 |
| REACTOME CLASSICAL ANTIBODY MEDIATED COMPLEMENT ACTIVATION | C1S |
| NICOTINIC ACETYLCHOLINE ACTIVATED CATION SELECTIVE CHANNEL ACTIVITY |  |
| NICOTINIC ACETYLCHOLINE GATED RECEPTOR CHANNEL COMPLEX |  |
| CYTOKINE PRODUCTION | TRAF2, BCL3, TLR4 |
| RESPONSE TO BACTERIUM |  |
| BIOCARTA LAIR PATHWAY |  |
| POSITIVE REGULATION OF CYTOKINE BIOSYNTHETIC PROCESS | TLR4 |
| CYTOKINE METABOLIC PROCESS | BCL3, TLR4 |
| NCI NFAT TFPATHWAY | EGR1, JUNB, FOS |
| REGULATION OF INTERFERON GAMMA BIOSYNTHETIC PROCESS |  |
| NCI CHEMOKINE RECEPTORS BIND CHEMOKINES | CXCL16 |
| POSITIVE REGULATION OF TRANSLATION | BCL3, TLR4 |
| REGULATION OF CYTOKINE BIOSYNTHETIC PROCESS | BCL3, TLR4 |
| REACTOME TOLL LIKE RECEPTOR 4 CASCADE | CD14, TICAM1, TBK1, TLR4 |
| CYTOKINE BIOSYNTHETIC PROCESS | BCL3, TLR4 |

| Color legend | | | | | | | | | | | |
| --- | --- | --- | --- | --- | --- | --- | --- | --- | --- | --- | --- |
| q-value | 1 | 0.2 | 0.05 | 0.01 | 0.001 | 0.0001 |
| Color |  | |  |  |  | |

TABLE OF Q-VALUES

| alternaria alternata beas2b | stachybotrys chartarum lung | Gene Set |
| --- | --- | --- |
| 4.8502272E-5 | 8.667609E-4 | KEGG\_CYTOKINE\_CYTOKINE\_RECEPTOR\_INTERACTION |
| 1.715488E-5 | 0.0030316834 | RESPONSE\_TO\_EXTERNAL\_STIMULUS |
| 0.02860721 | 2.8009398E-4 | REACTOME\_SIGNALING\_IN\_IMMUNE\_SYSTEM |
| 0.0012581189 | 0.012120066 | NETPATH\_IL\_5\_PATHWAY\_UP |
| 0.0 | 0.098460175 | NETPATH\_IL\_2\_PATHWAY\_UP |
| 4.841327E-5 | 1.1035433E-4 | IMMUNE\_SYSTEM\_PROCESS |
| 7.181068E-4 | 2.021267E-4 | REACTOME\_CLASS\_A1\_RHODOPSIN\_LIKE\_RECEPTORS |
| 0.0 | 5.516338E-5 | KEGG\_COMPLEMENT\_AND\_COAGULATION\_CASCADES |
| 1.2214923E-5 | 0.004897092 | BEHAVIOR |
| 0.0 | 4.4228967E-5 | DEFENSE\_RESPONSE |
| 0.0 | 8.301471E-4 | LOCOMOTORY\_BEHAVIOR |
| 0.029972985 | 0.003478014 | KEGG\_CHEMOKINE\_SIGNALING\_PATHWAY |
| 0.023852112 | 0.068581946 | REACTOME\_GPCR\_LIGAND\_BINDING |
| 0.13215114 | 0.099357516 | KEGG\_JAK\_STAT\_SIGNALING\_PATHWAY |
| 0.0015941259 | 0.06922398 | KEGG\_NOD\_LIKE\_RECEPTOR\_SIGNALING\_PATHWAY |
| 6.9193807E-6 | 0.003132014 | RESPONSE\_TO\_WOUNDING |
| 0.0027369289 | 0.17841046 | EXTRACELLULAR\_SPACE |
| 2.0601391E-4 | 0.1078629 | KEGG\_TOLL\_LIKE\_RECEPTOR\_SIGNALING\_PATHWAY |
| 0.0014360275 | 0.0027125722 | NETPATH\_IL\_1\_PATHWAY\_UP |
| 0.110834025 | 4.1575127E-4 | KEGG\_PRIMARY\_IMMUNODEFICIENCY |
| 0.0 | 8.2969455E-5 | REACTOME\_PEPTIDE\_LIGAND\_BINDING\_RECEPTORS |
| 0.0 | 0.012438191 | CYTOKINE\_ACTIVITY |
| 0.0 | 7.375062E-5 | CHEMOKINE\_ACTIVITY |
| 0.0 | 7.93557E-5 | CHEMOKINE\_RECEPTOR\_BINDING |
| 0.0061623864 | 0.0012814513 | KEGG\_INTESTINAL\_IMMUNE\_NETWORK\_FOR\_IGA\_PRODUCTION |
| 0.0 | 5.5177166E-5 | REACTOME\_CHEMOKINE\_RECEPTORS\_BIND\_CHEMOKINES |
| 0.0027465345 | 3.9366656E-4 | NETPATH\_IL\_3\_PATHWAY\_UP |
| 4.6192636E-5 | 3.6784775E-5 | IMMUNE\_RESPONSE |
| 4.58652E-5 | 0.0064382344 | REACTOME\_G\_ALPHA\_I\_SIGNALLING\_EVENTS |
| 9.6749085E-5 | 1.8657849E-4 | G\_PROTEIN\_COUPLED\_RECEPTOR\_BINDING |
| 0.0 | 3.9669833E-4 | INFLAMMATORY\_RESPONSE |
| 0.023742095 | 0.021733731 | KEGG\_ANTIGEN\_PROCESSING\_AND\_PRESENTATION |
| 0.020532541 | 0.16922897 | KEGG\_GRAFT\_VERSUS\_HOST\_DISEASE |
| 3.9136506E-4 | 0.004678987 | REACTOME\_COMPLEMENT\_CASCADE |
| 0.17835741 | 3.4417826E-4 | CATION\_HOMEOSTASIS |
| 0.012348721 | 0.047102768 | REACTOME\_INNATE\_IMMUNITY\_SIGNALING |
| 0.08715195 | 3.4941523E-4 | CHEMICAL\_HOMEOSTASIS |
| 0.18564403 | 3.452314E-4 | CELLULAR\_CATION\_HOMEOSTASIS |
| 0.031793345 | 0.013637285 | KEGG\_LEISHMANIA\_INFECTION |
| 0.007055827 | 0.0036915434 | BIOCARTA\_COMP\_PATHWAY |
| 0.15413347 | 0.0013074436 | CELLULAR\_DEFENSE\_RESPONSE |
| 0.057703048 | 2.7618292E-4 | ION\_HOMEOSTASIS |
| 0.0064716395 | 0.10351314 | NCI\_IL12\_2PATHWAY |
| 0.13101315 | 6.074107E-4 | CELLULAR\_HOMEOSTASIS |
| 0.0073246225 | 0.019787867 | REACTOME\_INITIAL\_TRIGGERING\_OF\_COMPLEMENT |
| 0.033842232 | 3.5422304E-4 | HUMORAL\_IMMUNE\_RESPONSE |
| 0.015852578 | 0.029798996 | BIOCARTA\_NO2IL12\_PATHWAY |
| 0.08906518 | 0.038418543 | NETPATH\_IL\_7\_PATHWAY\_UP |
| 7.040857E-5 | 0.012842769 | RESPONSE\_TO\_VIRUS |
| 0.01313057 | 0.012646181 | BIOCARTA\_CLASSIC\_PATHWAY |
| 0.017179936 | 0.003889722 | NCI\_IMMUNOREGULATORY\_INTERACTIONS\_BETWEEN\_A\_LYMPHOID\_AND\_A\_NON\_LYMPHOID\_CELL |
| 0.024500562 | 0.1804909 | BIOCARTA\_IL22BP\_PATHWAY |
| 0.004494796 | 0.010593823 | NETPATH\_IL\_6\_PATHWAY\_UP |
| 0.0 | 0.0020648958 | RESPONSE\_TO\_OTHER\_ORGANISM |
| 0.039924555 | 0.10266698 | NCI\_CD40\_PATHWAY |
| 6.4580886E-6 | 0.1695661 | MULTI\_ORGANISM\_PROCESS |
| 0.005413289 | 0.01000818 | RESPONSE\_TO\_BIOTIC\_STIMULUS |
| 0.0027055622 | 0.0013304139 | REACTOME\_IMMUNOREGULATORY\_INTERACTIONS\_BETWEEN\_A\_LYMPHOID\_AND\_A\_NON\_LYMPHOID\_CELL |
| 0.17900206 | 0.09126057 | BIOCARTA\_CASPASE\_PATHWAY |
| 0.048433393 | 0.15900218 | REACTOME\_CLASSICAL\_ANTIBODY\_MEDIATED\_COMPLEMENT\_ACTIVATION |
| 0.0329081 | 0.050021812 | NICOTINIC\_ACETYLCHOLINE\_ACTIVATED\_CATION\_SELECTIVE\_CHANNEL\_ACTIVITY |
| 0.030765496 | 0.0548055 | NICOTINIC\_ACETYLCHOLINE\_GATED\_RECEPTOR\_CHANNEL\_COMPLEX |
| 0.09149431 | 0.004944692 | CYTOKINE\_PRODUCTION |
| 0.020273667 | 0.09779012 | RESPONSE\_TO\_BACTERIUM |
| 0.022626787 | 0.03715054 | BIOCARTA\_LAIR\_PATHWAY |
| 0.0104400525 | 0.0047633494 | POSITIVE\_REGULATION\_OF\_CYTOKINE\_BIOSYNTHETIC\_PROCESS |
| 0.0048122746 | 0.0035099161 | CYTOKINE\_METABOLIC\_PROCESS |
| 0.0028159413 | 0.06361532 | NCI\_NFAT\_TFPATHWAY |
| 0.06865664 | 0.1687969 | REGULATION\_OF\_INTERFERON\_GAMMA\_BIOSYNTHETIC\_PROCESS |
| 0.09035489 | 0.037954282 | NCI\_CHEMOKINE\_RECEPTORS\_BIND\_CHEMOKINES |
| 0.02836 | 5.324376E-4 | POSITIVE\_REGULATION\_OF\_TRANSLATION |
| 0.0027807166 | 0.001354138 | REGULATION\_OF\_CYTOKINE\_BIOSYNTHETIC\_PROCESS |
| 0.18977349 | 0.10614081 | REACTOME\_TOLL\_LIKE\_RECEPTOR\_4\_CASCADE |
| 0.010602886 | 0.0037536372 | CYTOKINE\_BIOSYNTHETIC\_PROCESS |
