# Supplementary material for: Computational approaches for discovery of common immunomodulators in fungal infections: towards broad-spectrum immunotherapeutic interventions
Source: BMC Microbiol. 2013 Oct 7;13:224. doi: 10.1186/1471-2180-13-224 (PMC3853472; doi:10.1186/1471-2180-13-224)
Supplement: Additional file 1 — Details of up- and down- regulated biclusters. [file 1471-2180-13-224-S1.zip › 2013-kidane-bmc/details-of-biclusters/upreg-biclust-16.html]

**BICLUSTER\_ID** : UPREG-16  
**PATHOGENS** /2/ : a. fumigatus,c. albicans  
**KNOWN DRUG TARGETS** /22/ : PLAT, CCL2, CCND1, CD55, CD59, TGM2, PTPN1, NFKB1, COL6A1, GPRC5A, IL6R, NP, GSK3B, ANPEP, SERPINE1, PTGS2, ACVR1, MET, CDK2, MAPKAPK2, IL8, PLAUR  

| Gene Set | Leading Edge Genes |
| --- | --- |
| NETPATH EGFR1 PATHWAY UP | EMP1, DUSP4, EREG, HBEGF, DUSP6, PHLDA2, CCND1, IL4R, DKK3, AKAP12, SDC4, UBE2N, TGM2, IER3, GPRC5A, PHLDA1, NP, SPRY2, TGFA, EHD1, PTGS2, TNFAIP3, MET, TFPI2, DUSP1, PLAUR |
| KEGG CYTOKINE CYTOKINE RECEPTOR INTERACTION | CXCL1, CCL2, IL18R1, TNFRSF12A, CRLF2, CLCF1, IL4R, CXCL3, CXCL5, TNFSF9, MET, ACVR1, TNFRSF9, IL8, CXCL2 |
| KEGG HEMATOPOIETIC CELL LINEAGE | IL4R, CD38, ANPEP, IL6R, CD9, ITGA6, CD59, CD55 |
| KEGG EPITHELIAL CELL SIGNALING IN HELICOBACTER PYLORI INFECTION | NFKB1, CXCL1, HBEGF, RELA, MAP3K14, SRC, NFKBIA, MET, IL8 |
| NCI NFAT TFPATHWAY | PTPN1, FOSL1, PTGS2, IL8 |
| LOCOMOTORY BEHAVIOR | FOSL1, CXCL1, CCL2, IL8, PLAUR |
| NCI DISSOLUTION OF FIBRIN CLOT | SERPINE1, PLAT, PLAUR |
| NCI LYSOPHOSPHOLIPID PATHWAY | NFKB1, HBEGF, GSK3B, GNA13, RELA, SRC, NFKBIA, GNA12, IL8 |
| KEGG RIG I LIKE RECEPTOR SIGNALING PATHWAY | NFKB1, DDX3X, TRIM25, NFKBIA, NFKBIB, ISG15, IL8, RELA |
| CHEMOKINE ACTIVITY | CXCL3, CXCL1, CCL2, IL8 |
| KEGG NOD LIKE RECEPTOR SIGNALING PATHWAY | NFKBIA, NFKBIB, TNFAIP3, CXCL1, CCL2, IL8, CXCL2 |
| CHEMOKINE RECEPTOR BINDING | CXCL3, CXCL1, CCL2, IL8 |
| REACTOME TRAF6 MEDIATED INDUCTION OF THE ANTIVIRAL CYTOKINE IFN ALPHA BETA CASCADE | NFKB1, DUSP4, NFKBIB, NFKB2, DUSP6, NFKBIA, MAPKAPK2, TICAM1 |
| REACTOME CHEMOKINE RECEPTORS BIND CHEMOKINES | CXCL3, CXCL1, CCL2, IL8 |
| BIOCARTA STEM PATHWAY | IL8 |
| NETPATH IL 1 PATHWAY | NFKB1, MAP3K14, NFKBIA, NFKBIB, MAP3K7IP2, RELA |
| KEGG ECM RECEPTOR INTERACTION | COL6A1, ITGAV, LAMB3, SDC4, ITGA6, TNC |
| KEGG MAPK SIGNALING PATHWAY | DUSP4, DUSP5, DUSP10, NFKB2, DUSP6, RELA, NRAS, MAP3K14, DUSP14, PPP3CC, DAXX, MAPKAPK2, MAP4K4, RELB, DUSP1 |
| BIOCARTA INFLAM PATHWAY | IL8 |
| NETPATH IL 7 PATHWAY UP | CXCL3, CXCL5, CXCL1, IL8, CXCL2 |
| KEGG T CELL RECEPTOR SIGNALING PATHWAY | NFKBIB, NCK2, MALT1, GSK3B, NRAS, RELA, MAP3K14, NFKBIA, PPP3CC, CBLB |
| BIOCARTA IL17 PATHWAY | CD58, IL8 |
| POSITIVE REGULATION OF CELL PROLIFERATION | TGFA, FOSL1, SPHK1, EREG, CDK2 |
| CORUM CHUK-NFKB2-REL-IKBKG-SPAG9-NFKB1-NFKBIE-COPB2-TNIP1-NFKBIA-RELA-TNIP2 COMPLEX | NFKB1, NFKBIA, SPAG9, REL, TNIP1, NFKB2, TNIP2, RELA |
| KEGG CYTOSOLIC DNA SENSING PATHWAY | NFKB1, NFKBIA, NFKBIB, DDX58, RELA |
| BIOCARTA CYTOKINE PATHWAY | IL8 |
| POSITIVE REGULATION OF CYTOKINE PRODUCTION | EREG, MALT1 |
| BIOCARTA ERYTH PATHWAY |  |
| POSITIVE REGULATION OF IMMUNE RESPONSE | EREG, MALT1 |
| CORUM TNF-ALPHA/NF-KAPPA B SIGNALING COMPLEX CHUK KPNA3 NFKB2 NFKBIB REL IKBKG NFKB1 NFKBIE RELB NFKBIA RELA TNIP2 |  |
| NCI CD40 PATHWAY | MAP3K14, NFKBIA, BIRC3, TNFAIP3, BIRC2, RELA |
| KEGG HYPERTROPHIC CARDIOMYOPATHY HCM | ITGAV, ITGA6, PRKAG2 |
| REGULATION OF IMMUNE RESPONSE | EREG, MALT1 |
| BIOCARTA CDMAC PATHWAY | NFKBIA, RELA |
| BIOCARTA ASBCELL PATHWAY |  |
| CORUM TNF-ALPHA/NF-KAPPA B SIGNALING COMPLEX RPL6 RPL30 RPS13 CHUK DDX3X NFKB2 NFKBIB REL IKBKG NFKB1 MAP3K8 RELB GLG1 NFKBIA RELA TNIP2 GTF2I |  |
| BIOCARTA TNFR2 PATHWAY | MAP3K14, NFKBIA, TNFAIP3, DUSP1, RELA |
| ST TUMOR NECROSIS FACTOR PATHWAY |  |
| POSITIVE REGULATION OF PEPTIDYL TYROSINE PHOSPHORYLATION | CLCF1 |
| POSITIVE REGULATION OF MULTICELLULAR ORGANISMAL PROCESS | SPHK1, EREG, MALT1 |
| BIOCARTA DC PATHWAY |  |
| POSITIVE REGULATION OF RESPONSE TO STIMULUS |  |

| Color legend | | | | | | | | | | | |
| --- | --- | --- | --- | --- | --- | --- | --- | --- | --- | --- | --- |
| q-value | 1 | 0.2 | 0.05 | 0.01 | 0.001 | 0.0001 |
| Color |  | |  |  |  | |

TABLE OF Q-VALUES

| candida albicans moddc135 | aspergillus fumigatus cluture filtrates a549 | aspergillus fumigatus dendritic | Gene Set |
| --- | --- | --- | --- |
| 0.0 | 8.3157216E-4 | 3.9014107E-5 | NETPATH\_EGFR1\_PATHWAY\_UP |
| 0.0 | 0.050987493 | 0.0 | KEGG\_CYTOKINE\_CYTOKINE\_RECEPTOR\_INTERACTION |
| 0.0 | 0.17998308 | 0.001619189 | KEGG\_HEMATOPOIETIC\_CELL\_LINEAGE |
| 0.002267632 | 0.08678882 | 0.09923221 | KEGG\_EPITHELIAL\_CELL\_SIGNALING\_IN\_HELICOBACTER\_PYLORI\_INFECTION |
| 5.8961764E-4 | 0.14430721 | 1.962291E-4 | NCI\_NFAT\_TFPATHWAY |
| 3.2139455E-6 | 0.064572826 | 0.04213685 | LOCOMOTORY\_BEHAVIOR |
| 0.034454864 | 0.11790499 | 0.07889986 | NCI\_DISSOLUTION\_OF\_FIBRIN\_CLOT |
| 0.0074755205 | 0.084783584 | 0.08021661 | NCI\_LYSOPHOSPHOLIPID\_PATHWAY |
| 1.6542599E-5 | 0.13297741 | 0.030804053 | KEGG\_RIG\_I\_LIKE\_RECEPTOR\_SIGNALING\_PATHWAY |
| 0.0 | 0.13061193 | 3.546737E-5 | CHEMOKINE\_ACTIVITY |
| 2.5522509E-6 | 0.04048064 | 0.021367086 | KEGG\_NOD\_LIKE\_RECEPTOR\_SIGNALING\_PATHWAY |
| 0.0 | 0.12956315 | 2.8777304E-5 | CHEMOKINE\_RECEPTOR\_BINDING |
| 0.011636542 | 0.10125844 | 0.18952166 | REACTOME\_TRAF6\_MEDIATED\_INDUCTION\_OF\_THE\_ANTIVIRAL\_CYTOKINE\_IFN\_ALPHA\_BETA\_CASCADE |
| 0.0 | 0.0 | 0.0 | REACTOME\_CHEMOKINE\_RECEPTORS\_BIND\_CHEMOKINES |
| 3.7597125E-5 | 0.014165326 | 0.016043266 | BIOCARTA\_STEM\_PATHWAY |
| 5.2014195E-5 | 0.13162808 | 0.021979617 | NETPATH\_IL\_1\_PATHWAY |
| 0.009347258 | 0.132169 | 0.0019129739 | KEGG\_ECM\_RECEPTOR\_INTERACTION |
| 0.004631088 | 0.120740704 | 0.18435535 | KEGG\_MAPK\_SIGNALING\_PATHWAY |
| 0.0 | 0.0013572491 | 5.4974487E-5 | BIOCARTA\_INFLAM\_PATHWAY |
| 0.0 | 0.0386872 | 0.0 | NETPATH\_IL\_7\_PATHWAY\_UP |
| 0.002180355 | 0.13845338 | 0.06693496 | KEGG\_T\_CELL\_RECEPTOR\_SIGNALING\_PATHWAY |
| 1.6053132E-4 | 0.001628699 | 0.035758696 | BIOCARTA\_IL17\_PATHWAY |
| 4.1946248E-4 | 0.13124275 | 0.13047208 | POSITIVE\_REGULATION\_OF\_CELL\_PROLIFERATION |
| 3.231262E-4 | 0.17240939 | 0.030155556 | CORUM\_CHUK-NFKB2-REL-IKBKG-SPAG9-NFKB1-NFKBIE-COPB2-TNIP1-NFKBIA-RELA-TNIP2\_COMPLEX |
| 1.3989894E-4 | 0.13053004 | 0.15817112 | KEGG\_CYTOSOLIC\_DNA\_SENSING\_PATHWAY |
| 0.0 | 5.6428078E-5 | 0.0012329832 | BIOCARTA\_CYTOKINE\_PATHWAY |
| 0.024859793 | 0.11768008 | 0.1820822 | POSITIVE\_REGULATION\_OF\_CYTOKINE\_PRODUCTION |
| 3.5301655E-5 | 0.14564127 | 0.012676984 | BIOCARTA\_ERYTH\_PATHWAY |
| 0.0127873905 | 0.12950608 | 0.13899016 | POSITIVE\_REGULATION\_OF\_IMMUNE\_RESPONSE |
| 2.466239E-4 | 0.15513363 | 0.03884668 | CORUM\_TNF-ALPHA/NF-KAPPA\_B\_SIGNALING\_COMPLEX\_CHUK\_KPNA3\_NFKB2\_NFKBIB\_REL\_IKBKG\_\_NFKB1\_NFKBIE\_RELB\_\_NFKBIA\_RELA\_TNIP2 |
| 1.14572584E-4 | 0.08014807 | 0.030180147 | NCI\_CD40\_PATHWAY |
| 0.014559986 | 0.10251222 | 0.13966201 | KEGG\_HYPERTROPHIC\_CARDIOMYOPATHY\_HCM |
| 0.022878889 | 0.09040961 | 0.13338058 | REGULATION\_OF\_IMMUNE\_RESPONSE |
| 0.0035081438 | 0.19136539 | 0.046208695 | BIOCARTA\_CDMAC\_PATHWAY |
| 0.0067488668 | 0.1806629 | 0.16922034 | BIOCARTA\_ASBCELL\_PATHWAY |
| 2.3260359E-4 | 0.13089982 | 0.03866104 | CORUM\_TNF-ALPHA/NF-KAPPA\_B\_SIGNALING\_COMPLEX\_RPL6\_RPL30\_RPS13\_CHUK\_DDX3X\_NFKB2\_NFKBIB\_REL\_IKBKG\_NFKB1\_MAP3K8\_RELB\_GLG1\_NFKBIA\_RELA\_TNIP2\_\_GTF2I |
| 9.2645256E-5 | 0.092164285 | 0.09709073 | BIOCARTA\_TNFR2\_PATHWAY |
| 0.055568002 | 0.0015568112 | 0.13624333 | ST\_TUMOR\_NECROSIS\_FACTOR\_PATHWAY |
| 0.011540983 | 0.13979301 | 0.13259868 | POSITIVE\_REGULATION\_OF\_PEPTIDYL\_TYROSINE\_PHOSPHORYLATION |
| 0.0138140945 | 0.08996527 | 0.14453259 | POSITIVE\_REGULATION\_OF\_MULTICELLULAR\_ORGANISMAL\_PROCESS |
| 0.002013036 | 0.07580912 | 0.042549185 | BIOCARTA\_DC\_PATHWAY |
| 0.06268975 | 0.08907495 | 0.12023649 | POSITIVE\_REGULATION\_OF\_RESPONSE\_TO\_STIMULUS |
