# Supplementary material for: Computational approaches for discovery of common immunomodulators in fungal infections: towards broad-spectrum immunotherapeutic interventions
Source: BMC Microbiol. 2013 Oct 7;13:224. doi: 10.1186/1471-2180-13-224 (PMC3853472; doi:10.1186/1471-2180-13-224)
Supplement: Additional file 1 — Details of up- and down- regulated biclusters. [file 1471-2180-13-224-S1.zip › 2013-kidane-bmc/details-of-biclusters/upreg-biclust-18.html]

**BICLUSTER\_ID** : UPREG-18  
**PATHOGENS** /4/ : p. jirovecii,a. alternata,a. fumigatus,c. albicans  
**KNOWN DRUG TARGETS** /11/ : CCL2, CD40, TFPI, IL6, SERPINE1, F3, IL1B, CCL5, PTGER2, CXCL10, PLAUR  

| Gene Set | Leading Edge Genes |
| --- | --- |
| RESPONSE TO EXTERNAL STIMULUS | IL1RAP, CXCL1, CCL2, CD40, TFPI, SERPINE1, CCL5, CXCL2, CXCL10, PLAUR |
| KEGG CYTOKINE CYTOKINE RECEPTOR INTERACTION | CSF1, CXCL1, CCL2, CD40, IL6, IL1B, CCL5, CXCL2, CXCL10 |
| RESPONSE TO WOUNDING | IL1RAP, CXCL1, CD40, TFPI, SERPINE1, CCL5, CXCL2, CXCL10 |
| KEGG CYTOSOLIC DNA SENSING PATHWAY | NFKBIA, IL6, IL1B, CCL5, CXCL10, IRF7 |
| BEHAVIOR | CXCL1, CCL5, CCL2, CXCL2, CXCL10, PLAUR |
| REACTOME CLASS A1 RHODOPSIN LIKE RECEPTORS | CXCL1, CCL5, CCL2, PTGER2, CXCL2, CXCL10 |
| LOCOMOTORY BEHAVIOR | CXCL1, CCL5, CCL2, CXCL2, CXCL10, PLAUR |
| REACTOME GPCR LIGAND BINDING | CXCL1, CCL5, CCL2, PTGER2, CXCL2, CXCL10 |
| CHEMOKINE ACTIVITY | CXCL1, CCL5, CCL2, CXCL2, CXCL10 |
| KEGG COMPLEMENT AND COAGULATION CASCADES | F3, SERPINE1, PLAUR |
| G PROTEIN COUPLED RECEPTOR BINDING | CXCL1, CCL5, CCL2, CXCL2, CXCL10 |
| DEFENSE RESPONSE | MX2, IL1RAP, CXCL1, CD40, CCL5, CXCL2, CXCL10, MX1 |
| REACTOME PEPTIDE LIGAND BINDING RECEPTORS | CXCL1, CCL5, CCL2, CXCL2, CXCL10 |
| CHEMOKINE RECEPTOR BINDING | CXCL1, CCL5, CCL2, CXCL2, CXCL10 |
| CYTOKINE ACTIVITY | CXCL1, CSF1, CCL5, CCL2, CXCL2, CXCL10 |
| REACTOME CHEMOKINE RECEPTORS BIND CHEMOKINES | CXCL1, CCL5, CCL2, CXCL2, CXCL10 |
| REACTOME G ALPHA I SIGNALLING EVENTS | CXCL1, CCL5, CXCL2, CXCL10 |
| KEGG GRAFT VERSUS HOST DISEASE | IL6, IL1B |
| INFLAMMATORY RESPONSE | CXCL1, CCL5, CXCL2, CXCL10 |
| EXTRACELLULAR SPACE | IL1B, CXCL1, CCL2, CXCL2 |
| EXTRACELLULAR REGION PART | IL1B, CXCL1, CCL2, CXCL2 |
| KEGG INTESTINAL IMMUNE NETWORK FOR IGA PRODUCTION | IL6, CD40 |
| NCI IL23PATHWAY | NFKBIA, IL1B, CXCL1, CCL2 |
| NETPATH WNT PATHWAY DOWN | IL1B, CXCL1 |
| HUMORAL IMMUNE RESPONSE | CCL2 |
| KEGG ALLOGRAFT REJECTION | CD40 |
| BIOCARTA FIBRINOLYSIS PATHWAY | SERPINE1 |
| KEGG TYPE I DIABETES MELLITUS | IL1B |
| KEGG AUTOIMMUNE THYROID DISEASE | CD40 |
| BIOCARTA INFLAM PATHWAY | CSF1 |

| Color legend | | | | | | | | | | | |
| --- | --- | --- | --- | --- | --- | --- | --- | --- | --- | --- | --- |
| q-value | 1 | 0.2 | 0.05 | 0.01 | 0.001 | 0.0001 |
| Color |  | |  |  |  | |

TABLE OF Q-VALUES

| candida albicans moddc135 | alternaria alternata beas2b | pneumocystis carinnii macrophage | aspergillus fumigatus dendritic | Gene Set |
| --- | --- | --- | --- | --- |
| 2.43421E-4 | 1.715488E-5 | 0.14160725 | 0.024326107 | RESPONSE\_TO\_EXTERNAL\_STIMULUS |
| 0.0 | 4.8502272E-5 | 0.016364018 | 0.0 | KEGG\_CYTOKINE\_CYTOKINE\_RECEPTOR\_INTERACTION |
| 2.1165008E-6 | 6.9193807E-6 | 0.04277774 | 0.0047411146 | RESPONSE\_TO\_WOUNDING |
| 1.3989894E-4 | 4.208835E-4 | 0.16132888 | 0.15817112 | KEGG\_CYTOSOLIC\_DNA\_SENSING\_PATHWAY |
| 2.2250392E-6 | 1.2214923E-5 | 0.15872823 | 0.023901414 | BEHAVIOR |
| 1.4066127E-4 | 7.181068E-4 | 1.738751E-4 | 0.003579989 | REACTOME\_CLASS\_A1\_RHODOPSIN\_LIKE\_RECEPTORS |
| 3.2139455E-6 | 0.0 | 0.108142614 | 0.04213685 | LOCOMOTORY\_BEHAVIOR |
| 0.0016598669 | 0.023852112 | 0.014167708 | 0.023223832 | REACTOME\_GPCR\_LIGAND\_BINDING |
| 0.0 | 0.0 | 0.041768454 | 3.546737E-5 | CHEMOKINE\_ACTIVITY |
| 0.0020074716 | 0.0 | 0.0145290345 | 0.03170033 | KEGG\_COMPLEMENT\_AND\_COAGULATION\_CASCADES |
| 0.0 | 9.6749085E-5 | 0.017283333 | 6.563468E-5 | G\_PROTEIN\_COUPLED\_RECEPTOR\_BINDING |
| 2.1694132E-6 | 0.0 | 0.04436318 | 0.106237434 | DEFENSE\_RESPONSE |
| 0.0 | 0.0 | 2.594948E-4 | 0.0 | REACTOME\_PEPTIDE\_LIGAND\_BINDING\_RECEPTORS |
| 0.0 | 0.0 | 0.04107923 | 2.8777304E-5 | CHEMOKINE\_RECEPTOR\_BINDING |
| 0.0 | 0.0 | 1.1591674E-4 | 0.0 | CYTOKINE\_ACTIVITY |
| 0.0 | 0.0 | 0.016418632 | 0.0 | REACTOME\_CHEMOKINE\_RECEPTORS\_BIND\_CHEMOKINES |
| 0.009618234 | 4.58652E-5 | 0.030884687 | 0.13351512 | REACTOME\_G\_ALPHA\_I\_SIGNALLING\_EVENTS |
| 0.0 | 0.020532541 | 0.044786237 | 0.036907617 | KEGG\_GRAFT\_VERSUS\_HOST\_DISEASE |
| 4.0434443E-6 | 0.0 | 0.048219085 | 0.015176189 | INFLAMMATORY\_RESPONSE |
| 0.0 | 0.0027369289 | 0.033668406 | 0.0 | EXTRACELLULAR\_SPACE |
| 4.004787E-5 | 0.18083072 | 0.062482 | 6.125904E-5 | EXTRACELLULAR\_REGION\_PART |
| 1.4610786E-4 | 0.0061623864 | 0.14613497 | 0.08491679 | KEGG\_INTESTINAL\_IMMUNE\_NETWORK\_FOR\_IGA\_PRODUCTION |
| 0.0 | 6.501296E-4 | 0.19927236 | 0.003802986 | NCI\_IL23PATHWAY |
| 0.002521776 | 0.16622809 | 0.14159183 | 0.15058221 | NETPATH\_WNT\_PATHWAY\_DOWN |
| 4.1889373E-4 | 0.033842232 | 0.09870169 | 0.13597828 | HUMORAL\_IMMUNE\_RESPONSE |
| 0.0 | 0.0072650104 | 0.04673168 | 0.035359737 | KEGG\_ALLOGRAFT\_REJECTION |
| 0.009271151 | 0.18600726 | 0.17358682 | 0.07972296 | BIOCARTA\_FIBRINOLYSIS\_PATHWAY |
| 0.0 | 0.09067089 | 0.041445535 | 0.018020527 | KEGG\_TYPE\_I\_DIABETES\_MELLITUS |
| 6.802776E-4 | 0.0031133108 | 0.041329965 | 0.09974431 | KEGG\_AUTOIMMUNE\_THYROID\_DISEASE |
| 0.0 | 0.024269812 | 0.14592804 | 5.4974487E-5 | BIOCARTA\_INFLAM\_PATHWAY |
