# Supplementary material for: Computational approaches for discovery of common immunomodulators in fungal infections: towards broad-spectrum immunotherapeutic interventions
Source: BMC Microbiol. 2013 Oct 7;13:224. doi: 10.1186/1471-2180-13-224 (PMC3853472; doi:10.1186/1471-2180-13-224)
Supplement: Additional file 1 — Details of up- and down- regulated biclusters. [file 1471-2180-13-224-S1.zip › 2013-kidane-bmc/details-of-biclusters/upreg-biclust-20.html]

**BICLUSTER\_ID** : UPREG-20  
**PATHOGENS** /2/ : a. alternata,c. albicans  
**KNOWN DRUG TARGETS** /24/ : SMOX, PLAT, CCL2, SRD5A1, TARS, CCND1, PLAU, ASNS, GCH1, PIM1, CD55, CFB, FGF2, HDAC9, GARS, IFNGR1, JAK2, NP, TNFRSF1A, IL6, F3, ICAM1, IL8, PLAUR  

| Gene Set | Leading Edge Genes |
| --- | --- |
| NETPATH IL 2 PATHWAY UP | DDX21, PFKFB3, SRD5A1, TARS, PMAIP1, ASNS, FOS, CREM, BHLHB2, CFLAR, B4GALT5, MFHAS1, GARS, DENND3, VEGF, NFIL3, BAG1, ATF4, ICAM1, IRF1, MYC, UPP1, ZNF267, MMD, FRMD4B, CCND1, SLC2A3, RELA, OPCML, PLAU, PRKCD, PIM1, IER3, DUSP5, ETS2, USP15, SAMHD1, JUND, IL8, SLC7A5, CHSY1, PLAUR, MX1 |
| RESPONSE TO EXTERNAL STIMULUS | CCL2, PLAT, RELA, STC1, PLAU, FOSL1, ASNS, STC2, RIPK2, FOS, CXCL2, PTX3, FGF2, HDAC9, CXCL1, CEBPB, CXCL6, TNFRSF1A, CHST2, IL8, PLAUR |
| NETPATH TNF ALPHA PATHWAY UP | EMP1, TP53BP2, OPTN, BDNF, PMAIP1, NFKBIA, EGR1, SMG1, GCH1, SF3A3, RIPK2, CXCL2, KLF7, RELB, SOD2, CFLAR, CASP4, TNIP1, CXCL1, CSF1, TRAF3, JUNB, BCL3, ATF4, EFNA1, TNFAIP2, TNFAIP8, TNFAIP3, PTRH2, ICAM1, IRF1, TFPI2, IER2, GALNAC4S-6ST, SMOX, CCL2, NFKBIE, IL32, BIRC2, RRS1, PLAU, VEGFC, SDC4, PTX3, IER3, AFF1, BIRC3, NR4A1, KLF10, PPP1R15A, NRIP1, SMAD3, ETS2, ZFP36, IL8, PLAUR, MX1 |
| NETPATH IL 1 PATHWAY UP | NFKBIE, CCL2, RELA, FOSL1, NFKBIA, PRKCD, SLC25A28, CXCL2, SOD2, CFB, BIRC3, CXCL1, GARS, NP, ICAM1, ZFP36, IL8, MYC |
| KEGG CYTOKINE CYTOKINE RECEPTOR INTERACTION | PDGFB, PDGFA, IFNGR1, CXCL1, CCL2, CXCL6, VEGFC, IL11, TNFSF15, IL6, TNFRSF1A, IL8, CXCL2 |
| LOCOMOTORY BEHAVIOR | CXCL1, CCL2, CXCL6, PLAU, FOSL1, IL8, CXCL2, PLAUR, FGF2 |
| KEGG COMPLEMENT AND COAGULATION CASCADES | PLAU, F3, CD55, PLAUR, CFB |
| BEHAVIOR | CXCL1, FOSB, CCL2, CXCL6, PLAU, FOSL1, CXCL2, IL8, PLAUR, FGF2 |
| NETPATH IL 5 PATHWAY UP | IRF2, MMD, CCL2, NFKBIE, RELA, EGR1, CD55, RELB, IER3, CASP4, BIRC3, DUSP5, TRAF3, NFIL3, ATF4, ICAM1, IL8, UPP1, IER2 |
| NETPATH EGFR1 PATHWAY UP | DDX21, EMP1, ZFP36L2, IL32, NP, CXCL6, CCND1, PLAU, VEGF, EGR1, TNFAIP3, SDC4, MYC, PLAUR, IER3 |
| NETPATH IL 4 PATHWAY DOWN | LITAF, CCL2, DDIT4, NFKBIA, ICAM1, IRF1, CXCL2, BCL6, IL8 |
| NETPATH KIT RECEPTOR PATHWAY UP | GDF15, MCL1, JUNB, CCL2, ETV5, CCND1, VEGF, JUND, ATF4, TNFRSF1A, EGR1, FOS, MYC, IER3 |
| KEGG CHEMOKINE SIGNALING PATHWAY | CXCL1, JAK2, CCL2, CXCL6, RELA, NFKBIA, PRKCD, IL8, CXCL2, GRK5 |
| DEFENSE RESPONSE | MICB, IL32, RELA, FOSL1, VEZF1, RIPK2, FOS, CXCL2, PTX3, HDAC9, TNIP1, CXCL1, CEBPB, CXCL6, TNFRSF1A, CHST2, IL8, MX1, HLA-G, BNIP3 |
| INFLAMMATORY RESPONSE | HDAC9, CXCL1, CEBPB, CXCL6, TNFRSF1A, RIPK2, IL8, CXCL2 |
| EXTRACELLULAR SPACE | LOXL2, CXCL1, CCL2, IL32, TNFAIP2, CXCL2, IL8, FGF2 |
| NETPATH TNF ALPHA PATHWAY DOWN | CCND1, TARS, JMJD1A, NFKBIA, EGR1, EXT1, SDC4, CXCL2, BHLHB2, IER3, NR4A1, CXCL1, KLF10, PPP1R15A, JUNB, FOSB, MNT, VEGF, DDX3X, TNFAIP2, MAFF, TNFAIP3, ZFP36, IRF1 |
| NETPATH IL 3 PATHWAY UP | NFIL3, MCL1, CCL2, FOS, IL8, MYC |
| REACTOME PEPTIDE LIGAND BINDING RECEPTORS | CXCR7, CXCL1, CCL2, CXCL2, IL8, CXCL6 |
| CYTOKINE ACTIVITY | GDF15, CXCL1, CCL2, CXCL2, IL8, CXCL6 |
| CHEMOKINE ACTIVITY | CXCL1, CCL2, IL8, CXCL2, CXCL6 |
| NCI DISSOLUTION OF FIBRIN CLOT | PLAU, PLAUR |
| BIOCARTA LAIR PATHWAY | ICAM1, IL8 |
| G PROTEIN COUPLED RECEPTOR BINDING | CXCL1, CCL2, IL8, CXCL2, CXCL6 |
| BIOCARTA GRANULOCYTES PATHWAY | ICAM1, IL8 |
| KEGG NOD LIKE RECEPTOR SIGNALING PATHWAY | BIRC3, CXCL1, CCL2, BIRC2, RELA, NFKBIA, TNFAIP3, CXCL2, RIPK2, IL8 |
| BIOCARTA LYM PATHWAY | ICAM1, IL8 |
| CHEMOKINE RECEPTOR BINDING | CXCL1, CCL2, IL8, CXCL2, CXCL6 |
| NCI REG GR PATHWAY | POU2F1, EGR1, NR4A1, ICAM1, IRF1, IL8, FOS |
| REACTOME CHEMOKINE RECEPTORS BIND CHEMOKINES | CXCL1, CCL2, CXCL2, IL8, CXCL6 |
| POSITIVE REGULATION OF DEFENSE RESPONSE | TNFRSF1A |
| NCI NFAT TFPATHWAY | FOSL1, EGR1, POU2F1, PTPRK, JUNB, IL8, FOS |
| BIOCARTA STEM PATHWAY | IL8 |
| NETPATH HEDGEHOG PATHWAY UP | VEGF, NR4A1, MYC, CCND1 |
| LEUKOCYTE MIGRATION | IL8 |
| BIOCARTA FIBRINOLYSIS PATHWAY | PLAU |
| REACTOME PHASE 1 FUNCTIONALIZATION OF COMPOUNDS | SMOX |
| LEUKOCYTE CHEMOTAXIS | IL8 |
| NCI IL23PATHWAY | NFKBIA, CXCL1, CCL2, RELA |
| BIOCARTA INFLAM PATHWAY | PDGFA, IL8 |
| REACTOME G ALPHA I SIGNALLING EVENTS | CXCR7, CXCL1, CXCL2, IL8, CXCL6 |
| HORMONE ACTIVITY | STC2, STC1 |
| NCI CHEMOKINE RECEPTORS BIND CHEMOKINES |  |
| REGULATION OF INTERFERON GAMMA BIOSYNTHETIC PROCESS |  |
| HORMONE SECRETION | IL11 |
| NETPATH IL 6 PATHWAY UP | CXCL1, CEBPD, MCL1, JUNB, CEBPB, BCL3, SLC2A3, MAFF, ZFP36, IRF1, BHLHB2 |
| INTERFERON GAMMA PRODUCTION |  |
| NCI CD40 PATHWAY | NFKBIA, BIRC3, TNFAIP3, TRAF3, MYC, BIRC2, RELA |
| POSITIVE REGULATION OF TRANSLATION | BCL3 |
| RESPONSE TO VIRUS | FOSL1, TRIM22, BCL3, BNIP3 |
| REGULATION OF CYTOKINE BIOSYNTHETIC PROCESS | BCL3 |
| NCI IL12 2PATHWAY | RIPK2, RELB, FOS |
| REACTOME VIRAL DSRNA TLR3 TRIF COMPLEX ACTIVATES RIP1 | NFKBIA, TICAM1, RELA |
| CYTOKINE METABOLIC PROCESS | TNFSF15, BCL3 |
| CYTOKINE BIOSYNTHETIC PROCESS | BCL3 |

| Color legend | | | | | | | | | | | |
| --- | --- | --- | --- | --- | --- | --- | --- | --- | --- | --- | --- |
| q-value | 1 | 0.2 | 0.05 | 0.01 | 0.001 | 0.0001 |
| Color |  | |  |  |  | |

TABLE OF Q-VALUES

| candida albicans huvec | alternaria alternata beas2b | Gene Set |
| --- | --- | --- |
| 0.009711463 | 0.0 | NETPATH\_IL\_2\_PATHWAY\_UP |
| 0.19306602 | 1.715488E-5 | RESPONSE\_TO\_EXTERNAL\_STIMULUS |
| 0.17408594 | 0.06082179 | NETPATH\_TNF\_ALPHA\_PATHWAY\_UP |
| 0.0020151997 | 0.0014360275 | NETPATH\_IL\_1\_PATHWAY\_UP |
| 0.0076698544 | 4.8502272E-5 | KEGG\_CYTOKINE\_CYTOKINE\_RECEPTOR\_INTERACTION |
| 5.4281012E-5 | 0.0 | LOCOMOTORY\_BEHAVIOR |
| 0.060249306 | 0.0 | KEGG\_COMPLEMENT\_AND\_COAGULATION\_CASCADES |
| 7.269528E-5 | 1.2214923E-5 | BEHAVIOR |
| 5.682608E-4 | 0.0012581189 | NETPATH\_IL\_5\_PATHWAY\_UP |
| 0.0052840365 | 0.0482491 | NETPATH\_EGFR1\_PATHWAY\_UP |
| 0.13495818 | 0.0 | NETPATH\_IL\_4\_PATHWAY\_DOWN |
| 0.009533154 | 0.026568137 | NETPATH\_KIT\_RECEPTOR\_PATHWAY\_UP |
| 0.09618854 | 0.029972985 | KEGG\_CHEMOKINE\_SIGNALING\_PATHWAY |
| 0.104973674 | 0.0 | DEFENSE\_RESPONSE |
| 0.14119071 | 0.0 | INFLAMMATORY\_RESPONSE |
| 0.12368768 | 0.0027369289 | EXTRACELLULAR\_SPACE |
| 6.3579535E-4 | 0.09455213 | NETPATH\_TNF\_ALPHA\_PATHWAY\_DOWN |
| 0.04616931 | 0.0027465345 | NETPATH\_IL\_3\_PATHWAY\_UP |
| 0.015921747 | 0.0 | REACTOME\_PEPTIDE\_LIGAND\_BINDING\_RECEPTORS |
| 0.0049860743 | 0.0 | CYTOKINE\_ACTIVITY |
| 0.0024792356 | 0.0 | CHEMOKINE\_ACTIVITY |
| 0.10528463 | 0.059493493 | NCI\_DISSOLUTION\_OF\_FIBRIN\_CLOT |
| 0.026148528 | 0.022626787 | BIOCARTA\_LAIR\_PATHWAY |
| 0.08719925 | 9.6749085E-5 | G\_PROTEIN\_COUPLED\_RECEPTOR\_BINDING |
| 0.08973177 | 0.006535667 | BIOCARTA\_GRANULOCYTES\_PATHWAY |
| 0.035266537 | 0.0015941259 | KEGG\_NOD\_LIKE\_RECEPTOR\_SIGNALING\_PATHWAY |
| 0.17139763 | 0.02800103 | BIOCARTA\_LYM\_PATHWAY |
| 0.0022271401 | 0.0 | CHEMOKINE\_RECEPTOR\_BINDING |
| 0.059264697 | 0.058216974 | NCI\_REG\_GR\_PATHWAY |
| 0.0 | 0.0 | REACTOME\_CHEMOKINE\_RECEPTORS\_BIND\_CHEMOKINES |
| 0.10804863 | 0.032808315 | POSITIVE\_REGULATION\_OF\_DEFENSE\_RESPONSE |
| 0.031072352 | 0.0028159413 | NCI\_NFAT\_TFPATHWAY |
| 0.14155315 | 0.048412904 | BIOCARTA\_STEM\_PATHWAY |
| 0.044318005 | 0.1578012 | NETPATH\_HEDGEHOG\_PATHWAY\_UP |
| 0.10777622 | 0.023273164 | LEUKOCYTE\_MIGRATION |
| 0.08608703 | 0.18600726 | BIOCARTA\_FIBRINOLYSIS\_PATHWAY |
| 0.10817072 | 0.19245763 | REACTOME\_PHASE\_1\_FUNCTIONALIZATION\_OF\_COMPOUNDS |
| 0.0823816 | 0.036746003 | LEUKOCYTE\_CHEMOTAXIS |
| 0.0014167366 | 6.501296E-4 | NCI\_IL23PATHWAY |
| 0.023040734 | 0.024269812 | BIOCARTA\_INFLAM\_PATHWAY |
| 0.14138973 | 4.58652E-5 | REACTOME\_G\_ALPHA\_I\_SIGNALLING\_EVENTS |
| 0.04535491 | 0.032127097 | HORMONE\_ACTIVITY |
| 0.004932953 | 0.09035489 | NCI\_CHEMOKINE\_RECEPTORS\_BIND\_CHEMOKINES |
| 0.05996359 | 0.06865664 | REGULATION\_OF\_INTERFERON\_GAMMA\_BIOSYNTHETIC\_PROCESS |
| 0.05812768 | 0.14520921 | HORMONE\_SECRETION |
| 0.0011679985 | 0.004494796 | NETPATH\_IL\_6\_PATHWAY\_UP |
| 0.1398962 | 0.098003834 | INTERFERON\_GAMMA\_PRODUCTION |
| 0.022629568 | 0.039924555 | NCI\_CD40\_PATHWAY |
| 0.06905987 | 0.02836 | POSITIVE\_REGULATION\_OF\_TRANSLATION |
| 0.13223031 | 7.040857E-5 | RESPONSE\_TO\_VIRUS |
| 0.09263205 | 0.0027807166 | REGULATION\_OF\_CYTOKINE\_BIOSYNTHETIC\_PROCESS |
| 0.0036812855 | 0.0064716395 | NCI\_IL12\_2PATHWAY |
| 0.15074268 | 0.1594682 | REACTOME\_VIRAL\_DSRNA\_TLR3\_TRIF\_COMPLEX\_ACTIVATES\_RIP1 |
| 0.033609606 | 0.0048122746 | CYTOKINE\_METABOLIC\_PROCESS |
| 0.08593028 | 0.010602886 | CYTOKINE\_BIOSYNTHETIC\_PROCESS |
