# Supplementary material for: Computational approaches for discovery of common immunomodulators in fungal infections: towards broad-spectrum immunotherapeutic interventions
Source: BMC Microbiol. 2013 Oct 7;13:224. doi: 10.1186/1471-2180-13-224 (PMC3853472; doi:10.1186/1471-2180-13-224)
Supplement: Additional file 1 — Details of up- and down- regulated biclusters. [file 1471-2180-13-224-S1.zip › 2013-kidane-bmc/details-of-biclusters/upreg-biclust-22.html]

**BICLUSTER\_ID** : UPREG-22  
**PATHOGENS** /2/ : a. fumigatus,c. albicans  
**KNOWN DRUG TARGETS** /28/ : ATP2C1, JUN, CD44, CCL2, RAF1, PPP5C, CCND1, CASP3, PLAU, CYP1B1, PRKCA, FXYD2, PTK2, ATP1A1, MAP2K1, FGF2, EGFR, NFKB1, NP, GSK3B, PDPK1, PTGS2, PPAT, MAPKAPK2, IL8, IL1R1, PLAUR, GRB2  

| Gene Set | Leading Edge Genes |
| --- | --- |
| KEGG MAPK SIGNALING PATHWAY | NFKB2, BDNF, SOS1, DUSP6, PPP5C, MAPK8IP3, CASP3, RELA, NRAS, MAPKAPK5, PRKCA, RPS6KA1, RASA2, RRAS2, MAP3K2, RELB, FGF2, EGFR, MAP2K1, DUSP5, PDGFA, PPP3CB, CHUK, MAPKAPK2, DAXX, MAP4K4, CDC42, DUSP1 |
| NCI LYSOPHOSPHOLIPID PATHWAY | NFKB1, GNA11, GNB1, JUN, HBEGF, GSK3B, GNA13, CASP3, RELA, NFKBIA, PTK2, IL8, CRK, EGFR |
| KEGG CHEMOKINE SIGNALING PATHWAY | NFKBIB, GNB1, PARD3, CCL2, RAF1, SOS1, RELA, NRAS, CXCL3, PIK3CD, NFKBIA, PIK3CA, PTK2, GRK5, CXCL2, CRK, MAP2K1, NFKB1, CXCL1, GSK3B, CHUK, IL8, CDC42 |
| LOCOMOTORY BEHAVIOR | CXCL1, CCL2, PLAU, FOSL1, IL8, PLAUR, MAP2K1, FGF2 |
| NETPATH EGFR1 PATHWAY UP | EMP1, RRM2, CDKN1A, HBEGF, DUSP6, PHLDA2, CCND1, PTHLH, PLAU, SDC4, ARL4C, IER3, NAV3, NP, SPRY2, PTGS2, PPAT, TNFAIP3, NUP205, PLAUR, DUSP1 |
| NCI MET PATHWAY | JUN, RAF1, SOS1, PDPK1, PIK3CA, PTK2, STAT3, CRK, MAP2K1, GRB2 |
| BIOCARTA MET PATHWAY | JUN, RAF1, SOS1, PIK3CA, PTK2, STAT3, CRK, GRB2, MAP2K1 |
| BIOCARTA CDMAC PATHWAY | PRKCA, NFKBIA, JUN, RAF1, MAP2K1, RELA |
| KEGG T CELL RECEPTOR SIGNALING PATHWAY | NFKBIB, JUN, NCK2, NFKBIE, RAF1, SOS1, NRAS, RELA, PIK3CD, NFKBIA, PIK3CA, CBLB, MAP2K1, PPP3CB, CHUK, GSK3B, CDC42 |
| REACTOME TOLL LIKE RECEPTOR 3 CASCADE | NFKB1, NFKBIB, JUN, NFKB2, DUSP3, CHUK, DUSP6, RELA, RPS6KA1, NFKBIA, PPP2R1B, MAPKAPK2, TICAM1, MAP2K1 |
| KEGG B CELL RECEPTOR SIGNALING PATHWAY | NFKBIB, JUN, NFKBIE, PPP3CB, RAF1, GSK3B, SOS1, NRAS, RELA, PIK3CD, NFKBIA, PIK3CA, MAP2K1 |
| REACTOME TRAF6 MEDIATED INDUCTION OF THE ANTIVIRAL CYTOKINE IFN ALPHA BETA CASCADE | NFKB1, NFKBIB, JUN, NFKB2, DUSP3, CHUK, DUSP6, RELA, RPS6KA1, NFKBIA, PPP2R1B, MAPKAPK2, TICAM1, MAP2K1 |
| KEGG CYTOKINE CYTOKINE RECEPTOR INTERACTION | TNFRSF10B, PDGFA, CXCL1, CCL2, TNFRSF12A, VEGFC, CXCL3, IL8, CXCL2, EGFR |
| NCI NFAT TFPATHWAY | FOSL1, PTGS2, JUNB, IL8, ITCH, CASP3 |
| ATPASE ACTIVITY COUPLED TO TRANSMEMBRANE MOVEMENT OF IONS | ATP1B1, ATP2C1, ATP2B1, FXYD2, ATP1A1, ATP6V1B2, ATP6V1C1 |
| NCI AVB3 OPN PATHWAY | PLAU, NFKBIA, ITGAV, PIK3CA, JUN, CD44, CDC42, RELA |
| BIOCARTA GLEEVEC PATHWAY | PIK3CA, JUN, RAF1, SOS1, MAP2K1 |
| NETPATH IL 1 PATHWAY | NFKB1, NFKBIB, CHUK, MAP3K7IP2, RELA, SQSTM1, NFKBIA, IL1R1 |
| CHEMOKINE ACTIVITY | CXCL3, CXCL1, CCL2, IL8 |
| NCI DISSOLUTION OF FIBRIN CLOT | PLAU, PLAUR |
| KEGG NOD LIKE RECEPTOR SIGNALING PATHWAY | NFKBIB, BIRC3, CXCL1, CCL2, BIRC2, RELA, NFKBIA, TNFAIP3, CXCL2, IL8 |
| CHEMOKINE RECEPTOR BINDING | CXCL3, CXCL1, CCL2, IL8 |
| ATPASE ACTIVITY COUPLED TO TRANSMEMBRANE MOVEMENT OF IONS PHOSPHORYLATIVE MECHANISM | ATP1B1, ATP2C1, ATP2B1, ATP1A1 |
| REACTOME CHEMOKINE RECEPTORS BIND CHEMOKINES | CXCL3, CXCR7, CXCL1, CCL2, IL8 |
| ST B CELL ANTIGEN RECEPTOR | PIK3CD, NFKBIA, NFKBIB, PIK3CA, NFKB2, NFKBIE, SOS1, MAP2K1 |
| BIOCARTA STEM PATHWAY | IL8 |
| NCI PDGFRAPATHWAY | PIK3CA, ITGAV, PDGFA, JUN, SHB, CRK, SOS1 |
| REACTOME ENDOGENOUS STEROLS | CYP1B1 |
| LEUKOCYTE CHEMOTAXIS | IL8 |
| BIOCARTA INFLAM PATHWAY | PDGFA, IL8 |
| BIOCARTA IL17 PATHWAY | CD58, IL8 |
| CORUM CHUK-NFKB2-REL-IKBKG-SPAG9-NFKB1-NFKBIE-COPB2-TNIP1-NFKBIA-RELA-TNIP2 COMPLEX | NFKB1, SPAG9, REL, TNIP1, NFKB2, NFKBIE, CHUK, RELA, NFKBIA |
| BIOCARTA LYM PATHWAY | IL8 |
| NCI ENDOGENOUS STEROLS | CYP1B1 |
| BIOCARTA CD40 PATHWAY | NFKBIA, TNFAIP3, TRAF3, DUSP1, RELA |
| POSITIVE REGULATION OF DEFENSE RESPONSE |  |
| BIOCARTA ERYTH PATHWAY |  |
| SIG CD40PATHWAYMAP | NFKBIB, NFKB2, NFKBIE, TRAF3, MAPK8IP3, PIK3CD, MAPKAPK5, NFKBIA, PIK3CA, DUSP1 |
| CORUM TNF-ALPHA/NF-KAPPA B SIGNALING COMPLEX CHUK KPNA3 NFKB2 NFKBIB REL IKBKG NFKB1 NFKBIE RELB NFKBIA RELA TNIP2 |  |
| NCI CD40 PATHWAY | NFKBIA, BIRC3, TNFAIP3, TRAF3, BIRC2, RELA |
| ST T CELL SIGNAL TRANSDUCTION | NFKBIA, NFKB2, NFKBIE, SOS1 |
| TISSUE MORPHOGENESIS | ERCC3, KLF4 |
| REACTOME SPHINGOLIPID METABOLISM | COL4A3BP, PPAP2B, SPHK1, SGPP1, LASS2 |
| CORUM TNF-ALPHA/NF-KAPPA B SIGNALING COMPLEX RPL6 RPL30 RPS13 CHUK DDX3X NFKB2 NFKBIB REL IKBKG NFKB1 MAP3K8 RELB GLG1 NFKBIA RELA TNIP2 GTF2I |  |
| BIOCARTA TNFR2 PATHWAY | NFKBIA, TNFAIP3, TRAF3, DUSP1, RELA |

| Color legend | | | | | | | | | | | |
| --- | --- | --- | --- | --- | --- | --- | --- | --- | --- | --- | --- |
| q-value | 1 | 0.2 | 0.05 | 0.01 | 0.001 | 0.0001 |
| Color |  | |  |  |  | |

TABLE OF Q-VALUES

| candida albicans huvec | aspergillus fumigatus cluture filtrates a549 | Gene Set |
| --- | --- | --- |
| 0.19037609 | 0.120740704 | KEGG\_MAPK\_SIGNALING\_PATHWAY |
| 0.19033894 | 0.084783584 | NCI\_LYSOPHOSPHOLIPID\_PATHWAY |
| 0.09618854 | 0.10088094 | KEGG\_CHEMOKINE\_SIGNALING\_PATHWAY |
| 5.4281012E-5 | 0.064572826 | LOCOMOTORY\_BEHAVIOR |
| 0.0052840365 | 8.3157216E-4 | NETPATH\_EGFR1\_PATHWAY\_UP |
| 0.17205246 | 0.13343371 | NCI\_MET\_PATHWAY |
| 0.096628375 | 0.11091478 | BIOCARTA\_MET\_PATHWAY |
| 0.07875448 | 0.19136539 | BIOCARTA\_CDMAC\_PATHWAY |
| 0.046388313 | 0.13845338 | KEGG\_T\_CELL\_RECEPTOR\_SIGNALING\_PATHWAY |
| 0.17478716 | 0.08971636 | REACTOME\_TOLL\_LIKE\_RECEPTOR\_3\_CASCADE |
| 0.11673039 | 0.13446142 | KEGG\_B\_CELL\_RECEPTOR\_SIGNALING\_PATHWAY |
| 0.14167826 | 0.10125844 | REACTOME\_TRAF6\_MEDIATED\_INDUCTION\_OF\_THE\_ANTIVIRAL\_CYTOKINE\_IFN\_ALPHA\_BETA\_CASCADE |
| 0.0076698544 | 0.050987493 | KEGG\_CYTOKINE\_CYTOKINE\_RECEPTOR\_INTERACTION |
| 0.031072352 | 0.14430721 | NCI\_NFAT\_TFPATHWAY |
| 0.1215003 | 0.10697836 | ATPASE\_ACTIVITY\_COUPLED\_TO\_TRANSMEMBRANE\_MOVEMENT\_OF\_IONS |
| 0.077203505 | 0.14755891 | NCI\_AVB3\_OPN\_PATHWAY |
| 0.1752864 | 0.14519274 | BIOCARTA\_GLEEVEC\_PATHWAY |
| 0.117814705 | 0.13162808 | NETPATH\_IL\_1\_PATHWAY |
| 0.0024792356 | 0.13061193 | CHEMOKINE\_ACTIVITY |
| 0.10528463 | 0.11790499 | NCI\_DISSOLUTION\_OF\_FIBRIN\_CLOT |
| 0.035266537 | 0.04048064 | KEGG\_NOD\_LIKE\_RECEPTOR\_SIGNALING\_PATHWAY |
| 0.0022271401 | 0.12956315 | CHEMOKINE\_RECEPTOR\_BINDING |
| 0.09259346 | 0.15583727 | ATPASE\_ACTIVITY\_COUPLED\_TO\_TRANSMEMBRANE\_MOVEMENT\_OF\_IONS\_PHOSPHORYLATIVE\_MECHANISM |
| 0.0 | 0.0 | REACTOME\_CHEMOKINE\_RECEPTORS\_BIND\_CHEMOKINES |
| 0.045818783 | 0.074949 | ST\_B\_CELL\_ANTIGEN\_RECEPTOR |
| 0.14155315 | 0.014165326 | BIOCARTA\_STEM\_PATHWAY |
| 0.1407947 | 0.13217598 | NCI\_PDGFRAPATHWAY |
| 0.09555835 | 0.106379606 | REACTOME\_ENDOGENOUS\_STEROLS |
| 0.0823816 | 0.10609597 | LEUKOCYTE\_CHEMOTAXIS |
| 0.023040734 | 0.0013572491 | BIOCARTA\_INFLAM\_PATHWAY |
| 0.045749582 | 0.001628699 | BIOCARTA\_IL17\_PATHWAY |
| 0.08393664 | 0.17240939 | CORUM\_CHUK-NFKB2-REL-IKBKG-SPAG9-NFKB1-NFKBIE-COPB2-TNIP1-NFKBIA-RELA-TNIP2\_COMPLEX |
| 0.17139763 | 0.1187666 | BIOCARTA\_LYM\_PATHWAY |
| 0.09456388 | 0.10544677 | NCI\_ENDOGENOUS\_STEROLS |
| 0.13549858 | 0.13739029 | BIOCARTA\_CD40\_PATHWAY |
| 0.10804863 | 0.08528607 | POSITIVE\_REGULATION\_OF\_DEFENSE\_RESPONSE |
| 0.08735396 | 0.14564127 | BIOCARTA\_ERYTH\_PATHWAY |
| 0.09512491 | 0.15359046 | SIG\_CD40PATHWAYMAP |
| 0.08624834 | 0.15513363 | CORUM\_TNF-ALPHA/NF-KAPPA\_B\_SIGNALING\_COMPLEX\_CHUK\_KPNA3\_NFKB2\_NFKBIB\_REL\_IKBKG\_\_NFKB1\_NFKBIE\_RELB\_\_NFKBIA\_RELA\_TNIP2 |
| 0.022629568 | 0.08014807 | NCI\_CD40\_PATHWAY |
| 0.004998011 | 0.11773539 | ST\_T\_CELL\_SIGNAL\_TRANSDUCTION |
| 0.09528401 | 0.14050001 | TISSUE\_MORPHOGENESIS |
| 0.19353037 | 0.09959404 | REACTOME\_SPHINGOLIPID\_METABOLISM |
| 0.08620508 | 0.13089982 | CORUM\_TNF-ALPHA/NF-KAPPA\_B\_SIGNALING\_COMPLEX\_RPL6\_RPL30\_RPS13\_CHUK\_DDX3X\_NFKB2\_NFKBIB\_REL\_IKBKG\_NFKB1\_MAP3K8\_RELB\_GLG1\_NFKBIA\_RELA\_TNIP2\_\_GTF2I |
| 0.10791961 | 0.092164285 | BIOCARTA\_TNFR2\_PATHWAY |
