# Supplementary material for: Computational approaches for discovery of common immunomodulators in fungal infections: towards broad-spectrum immunotherapeutic interventions
Source: BMC Microbiol. 2013 Oct 7;13:224. doi: 10.1186/1471-2180-13-224 (PMC3853472; doi:10.1186/1471-2180-13-224)
Supplement: Additional file 1 — Details of up- and down- regulated biclusters. [file 1471-2180-13-224-S1.zip › 2013-kidane-bmc/details-of-biclusters/upreg-biclust-24.html]

**BICLUSTER\_ID** : UPREG-24  
**PATHOGENS** /2/ : p. jirovecii,s. chartarum  
**KNOWN DRUG TARGETS** /31/ : ORM1, MMP8, CCR5, MMP12, CTSD, CCL2, CD40, LGALS3, PLAU, SFTPD, CCL5, IFNG, CXCL10, FCGR2B, APOE, ANXA1, C1QB, MMP9, IFNGR1, CFD, CTSB, CTSS, IL6, F10, IL1B, ACSL4, F3, CASP1, PTGER2, CXCR4, PLAUR  

| Gene Set | Leading Edge Genes |
| --- | --- |
| RESPONSE TO EXTERNAL STIMULUS | CCR5, ORM1, CDKN1A, CCL2, CD40, PLAU, SFTPD, CCL5, CXCL2, CXCL10, CCL4, ANXA1, CXCL1, IL1A, CCR1, F10, CCL3, PGLYRP1, CXCR4, PLAUR |
| BEHAVIOR | APOE, CCR5, CXCL1, CCL2, PLAU, SFTPD, ACSL4, CCL3, CCL5, CXCL2, CXCR4, CXCL10, PLAUR |
| KEGG CYTOKINE CYTOKINE RECEPTOR INTERACTION | CCR5, IL1RAP, CCL2, CD40, CCL5, CXCL2, CXCL10, CCL4, CXCL1, IFNGR1, IL1A, CCR1, IL6, CCL3, IL1B, CXCR4 |
| LOCOMOTORY BEHAVIOR | CCR5, CXCL1, CCL2, PLAU, SFTPD, CCL3, CCL5, CXCL2, CXCL10, CXCR4, PLAUR |
| ENDOPEPTIDASE ACTIVITY | C1R, CTSD, MMP12, MMP14, MMP9, CFD, CTSE, CTSK, CTSB, CTSS, F10, CASP1, PDIA3 |
| RESPONSE TO WOUNDING | ANXA1, CCR5, ORM1, CXCL1, CD40, IL1A, CCR1, F10, CCL3, CCL5, CXCL2, CXCR4, CXCL10, CCL4 |
| PEPTIDASE ACTIVITY | C1R, CTSD, MMP12, MMP14, CFD, CTSK, CTSS, CTSB, F10, CASP1, PDIA3 |
| DEFENSE RESPONSE | CCR5, ORM1, SFTPD, CCL5, CXCL2, CXCL10, CCL4, ANXA1, CXCL1, IL1A, CCR1, LGALS3BP, CCL3, PGLYRP1, CXCR4, MX1 |
| INFLAMMATORY RESPONSE | ANXA1, CCR5, ORM1, CXCL1, CD40, IL1A, CCR1, CCL3, CCL5, CXCL2, CXCR4, CXCL10, CCL4 |
| REACTOME CLASS A1 RHODOPSIN LIKE RECEPTORS | CCR5, CXCL1, P2RY6, CCL2, CCR1, C3, CCL3, CCL5, PTGER2, CXCL2, CXCR4, CXCL10, CCL4 |
| REACTOME GPCR LIGAND BINDING | CCR5, CXCL1, P2RY6, CCL2, CCR1, C3, CCL3, CCL5, PTGER2, CXCL2, CXCR4, CXCL10, CCL4 |
| REACTOME PEPTIDE LIGAND BINDING RECEPTORS | CCR5, CXCL1, CCL2, CCR1, C3, CCL3, C3AR1, CCL5, CXCL2, CXCR4, CXCL10, CCL4 |
| KEGG COMPLEMENT AND COAGULATION CASCADES | PLAU, C1QB, F10, C3, C1R, C3AR1, F3, PLAUR |
| EXTRACELLULAR SPACE | C1QB, MMP8, ORM1, CXCL1, CCL2, IL1A, LGALS3BP, IL1RN, IL1B, CXCL2, CCL4 |
| REACTOME G ALPHA I SIGNALLING EVENTS | CCR5, CXCL1, CCR1, C3, CCL5, CXCL2, CXCR4, CXCL10 |
| REACTOME CHEMOKINE RECEPTORS BIND CHEMOKINES | CXCL1, CCL2, CCR1, CCL3, CCL5, CXCL2, CXCL10, CXCR4, CCL4 |
| KEGG GRAFT VERSUS HOST DISEASE | IL6, IL1B, IFNG, IL1A |
| CYTOKINE ACTIVITY | IL1RN, CCL3, CXCL1, CCL5, CCL2, CXCL2, CXCL10, CCL4 |
| BIOCARTA NKT PATHWAY | CCR5, CCL3, IFNG, CXCR4, CCL4, CCR1 |
| CHEMOKINE ACTIVITY | CCL3, CXCL1, CCL2, CCL5, CXCL2, CXCL10, CCL4 |
| G PROTEIN COUPLED RECEPTOR BINDING | CCL3, CXCL1, CCL2, CCL5, CXCL2, CXCL10, CCL4 |
| CHEMOKINE RECEPTOR BINDING | CCL3, CXCL1, CCL2, CCL5, CXCL2, CXCL10, CCL4 |
| KEGG SYSTEMIC LUPUS ERYTHEMATOSUS | FCGR2B, C1QB, C3, C1R, CD40 |
| KEGG INTESTINAL IMMUNE NETWORK FOR IGA PRODUCTION | IL6, CD40, PIGR, CXCR4, ITGB7 |
| REACTOME IMMUNOREGULATORY INTERACTIONS BETWEEN A LYMPHOID AND A NON LYMPHOID CELL | FCGR2B, C3, CD40, ITGB7 |
| KEGG HEMATOPOIETIC CELL LINEAGE | IL6, ITGAM, IL1B, CD14, IL1A |
| BIOCARTA EXTRINSIC PATHWAY | F10, F3 |
| SUGAR BINDING | SFTPD, LGALS3 |
| REACTOME FORMATION OF FIBRIN CLOT CLOTTING CASCADE | F10, F3 |
| HUMORAL IMMUNE RESPONSE | CCL2 |
| HEMOSTASIS | F10 |
| BIOCARTA ASBCELL PATHWAY | CD40 |
| NCI IMMUNOREGULATORY INTERACTIONS BETWEEN A LYMPHOID AND A NON LYMPHOID CELL | CD200, CD8B, C3, CD40, CD3D, ITGB7 |
| COAGULATION | F10 |
| BLOOD COAGULATION | F10 |
| REGULATION OF BODY FLUID LEVELS | F10 |
| HEMATOPOIETIN INTERFERON CLASSD200 DOMAIN CYTOKINE RECEPTOR ACTIVITY | IFNGR1 |
| KEGG ASTHMA |  |
| EXCITATORY EXTRACELLULAR LIGAND GATED ION CHANNEL ACTIVITY | P2RX4 |
| EXTRACELLULAR LIGAND GATED ION CHANNEL ACTIVITY | P2RX4 |
| DIGESTION | CTSE |

| Color legend | | | | | | | | | | | |
| --- | --- | --- | --- | --- | --- | --- | --- | --- | --- | --- | --- |
| q-value | 1 | 0.2 | 0.05 | 0.01 | 0.001 | 0.0001 |
| Color |  | |  |  |  | |

TABLE OF Q-VALUES

| pneumocystis carinnii macrophage | stachybotrys chartarum lung | Gene Set |
| --- | --- | --- |
| 0.14160725 | 0.0030316834 | RESPONSE\_TO\_EXTERNAL\_STIMULUS |
| 0.15872823 | 0.004897092 | BEHAVIOR |
| 0.016364018 | 8.667609E-4 | KEGG\_CYTOKINE\_CYTOKINE\_RECEPTOR\_INTERACTION |
| 0.108142614 | 8.301471E-4 | LOCOMOTORY\_BEHAVIOR |
| 0.040358678 | 0.030025851 | ENDOPEPTIDASE\_ACTIVITY |
| 0.04277774 | 0.003132014 | RESPONSE\_TO\_WOUNDING |
| 0.13859202 | 0.107635975 | PEPTIDASE\_ACTIVITY |
| 0.04436318 | 4.4228967E-5 | DEFENSE\_RESPONSE |
| 0.048219085 | 3.9669833E-4 | INFLAMMATORY\_RESPONSE |
| 1.738751E-4 | 2.021267E-4 | REACTOME\_CLASS\_A1\_RHODOPSIN\_LIKE\_RECEPTORS |
| 0.014167708 | 0.068581946 | REACTOME\_GPCR\_LIGAND\_BINDING |
| 2.594948E-4 | 8.2969455E-5 | REACTOME\_PEPTIDE\_LIGAND\_BINDING\_RECEPTORS |
| 0.0145290345 | 5.516338E-5 | KEGG\_COMPLEMENT\_AND\_COAGULATION\_CASCADES |
| 0.033668406 | 0.17841046 | EXTRACELLULAR\_SPACE |
| 0.030884687 | 0.0064382344 | REACTOME\_G\_ALPHA\_I\_SIGNALLING\_EVENTS |
| 0.016418632 | 5.5177166E-5 | REACTOME\_CHEMOKINE\_RECEPTORS\_BIND\_CHEMOKINES |
| 0.044786237 | 0.16922897 | KEGG\_GRAFT\_VERSUS\_HOST\_DISEASE |
| 1.1591674E-4 | 0.012438191 | CYTOKINE\_ACTIVITY |
| 0.106201544 | 0.10779688 | BIOCARTA\_NKT\_PATHWAY |
| 0.041768454 | 7.375062E-5 | CHEMOKINE\_ACTIVITY |
| 0.017283333 | 1.8657849E-4 | G\_PROTEIN\_COUPLED\_RECEPTOR\_BINDING |
| 0.04107923 | 7.93557E-5 | CHEMOKINE\_RECEPTOR\_BINDING |
| 0.034138743 | 0.005506665 | KEGG\_SYSTEMIC\_LUPUS\_ERYTHEMATOSUS |
| 0.14613497 | 0.0012814513 | KEGG\_INTESTINAL\_IMMUNE\_NETWORK\_FOR\_IGA\_PRODUCTION |
| 0.025689289 | 0.0013304139 | REACTOME\_IMMUNOREGULATORY\_INTERACTIONS\_BETWEEN\_A\_LYMPHOID\_AND\_A\_NON\_LYMPHOID\_CELL |
| 0.14391063 | 0.012549701 | KEGG\_HEMATOPOIETIC\_CELL\_LINEAGE |
| 0.17286684 | 0.1152361 | BIOCARTA\_EXTRINSIC\_PATHWAY |
| 0.15740086 | 0.19939512 | SUGAR\_BINDING |
| 0.03150514 | 0.17213066 | REACTOME\_FORMATION\_OF\_FIBRIN\_CLOT\_CLOTTING\_CASCADE |
| 0.09870169 | 3.5422304E-4 | HUMORAL\_IMMUNE\_RESPONSE |
| 0.18397155 | 0.17265753 | HEMOSTASIS |
| 0.1606871 | 0.14346233 | BIOCARTA\_ASBCELL\_PATHWAY |
| 0.08373454 | 0.003889722 | NCI\_IMMUNOREGULATORY\_INTERACTIONS\_BETWEEN\_A\_LYMPHOID\_AND\_A\_NON\_LYMPHOID\_CELL |
| 0.19515085 | 0.13898392 | COAGULATION |
| 0.18718082 | 0.15122637 | BLOOD\_COAGULATION |
| 0.15098336 | 0.12395742 | REGULATION\_OF\_BODY\_FLUID\_LEVELS |
| 0.17504902 | 0.15185583 | HEMATOPOIETIN\_INTERFERON\_CLASSD200\_DOMAIN\_CYTOKINE\_RECEPTOR\_ACTIVITY |
| 0.0478591 | 0.09246591 | KEGG\_ASTHMA |
| 0.18625905 | 1.3019271E-4 | EXCITATORY\_EXTRACELLULAR\_LIGAND\_GATED\_ION\_CHANNEL\_ACTIVITY |
| 0.1777229 | 1.4321198E-4 | EXTRACELLULAR\_LIGAND\_GATED\_ION\_CHANNEL\_ACTIVITY |
| 0.08366786 | 0.08851697 | DIGESTION |
