# Supplementary material for: Computational approaches for discovery of common immunomodulators in fungal infections: towards broad-spectrum immunotherapeutic interventions
Source: BMC Microbiol. 2013 Oct 7;13:224. doi: 10.1186/1471-2180-13-224 (PMC3853472; doi:10.1186/1471-2180-13-224)
Supplement: Additional file 1 — Details of up- and down- regulated biclusters. [file 1471-2180-13-224-S1.zip › 2013-kidane-bmc/details-of-biclusters/upreg-biclust-32.html]

**BICLUSTER\_ID** : UPREG-32  
**PATHOGENS** /3/ : c. albicans,a. fumigatus,s. chartarum  
**KNOWN DRUG TARGETS** /3/ : CCL2, PPARG, PLAUR  

| Gene Set | Leading Edge Genes |
| --- | --- |
| LOCOMOTORY BEHAVIOR | CXCL1, CCL2, PLAUR |
| KEGG CYTOKINE CYTOKINE RECEPTOR INTERACTION | OSMR, CXCL5, TNFSF9, CXCL1, CCL2, CXCL2 |
| CHEMOKINE ACTIVITY | CXCL1, CCL2 |
| NCI NFAT TFPATHWAY | PPARG |
| KEGG CHEMOKINE SIGNALING PATHWAY | CXCL5, NFKBIA, CXCL1, CCL2, CXCL2 |
| KEGG NOD LIKE RECEPTOR SIGNALING PATHWAY | NFKBIA, TNFAIP3, CXCL1, CCL2, CXCL2 |
| CHEMOKINE RECEPTOR BINDING | CXCL1, CCL2 |
| REACTOME CHEMOKINE RECEPTORS BIND CHEMOKINES | CXCL1, CCL2 |
| BIOCARTA IL17 PATHWAY |  |
| KEGG HEMATOPOIETIC CELL LINEAGE |  |
| REGULATION OF IMMUNE SYSTEM PROCESS | MALT1 |
| BIOCARTA TNFR2 PATHWAY | NFKBIA, TNFAIP3, TRAF3 |
| NCI CD40 PATHWAY | NFKBIA, BIRC3, TNFAIP3, TRAF3, BIRC2 |
| BIOCARTA HIVNEF PATHWAY |  |
| NETPATH IL 7 PATHWAY UP | CXCL5, CXCL1, TRAF3, CXCL2 |
| KEGG T CELL RECEPTOR SIGNALING PATHWAY | NFKBIA, PPP3CC, MALT1 |

| Color legend | | | | | | | | | | | |
| --- | --- | --- | --- | --- | --- | --- | --- | --- | --- | --- | --- |
| q-value | 1 | 0.2 | 0.05 | 0.01 | 0.001 | 0.0001 |
| Color |  | |  |  |  | |

TABLE OF Q-VALUES

| aspergillus fumigatus conidia a549 | candida albicans moddc135 | aspergillus fumigatus cluture filtrates a549 | stachybotrys chartarum lung | Gene Set |
| --- | --- | --- | --- | --- |
| 2.2720385E-5 | 3.2139455E-6 | 0.064572826 | 8.301471E-4 | LOCOMOTORY\_BEHAVIOR |
| 2.5672338E-5 | 0.0 | 0.050987493 | 8.667609E-4 | KEGG\_CYTOKINE\_CYTOKINE\_RECEPTOR\_INTERACTION |
| 2.8400484E-5 | 0.0 | 0.13061193 | 7.375062E-5 | CHEMOKINE\_ACTIVITY |
| 1.4434879E-4 | 5.8961764E-4 | 0.14430721 | 0.06361532 | NCI\_NFAT\_TFPATHWAY |
| 0.028497338 | 6.846009E-5 | 0.10088094 | 0.003478014 | KEGG\_CHEMOKINE\_SIGNALING\_PATHWAY |
| 7.929928E-5 | 2.5522509E-6 | 0.04048064 | 0.06922398 | KEGG\_NOD\_LIKE\_RECEPTOR\_SIGNALING\_PATHWAY |
| 0.0 | 0.0 | 0.12956315 | 7.93557E-5 | CHEMOKINE\_RECEPTOR\_BINDING |
| 0.0 | 0.0 | 0.0 | 5.5177166E-5 | REACTOME\_CHEMOKINE\_RECEPTORS\_BIND\_CHEMOKINES |
| 0.045313723 | 1.6053132E-4 | 0.001628699 | 0.08465764 | BIOCARTA\_IL17\_PATHWAY |
| 0.14205706 | 0.0 | 0.17998308 | 0.012549701 | KEGG\_HEMATOPOIETIC\_CELL\_LINEAGE |
| 0.09243208 | 4.633083E-4 | 0.12798534 | 0.179525 | REGULATION\_OF\_IMMUNE\_SYSTEM\_PROCESS |
| 0.011185336 | 9.2645256E-5 | 0.092164285 | 0.1395269 | BIOCARTA\_TNFR2\_PATHWAY |
| 3.4622008E-5 | 1.14572584E-4 | 0.08014807 | 0.10266698 | NCI\_CD40\_PATHWAY |
| 0.029508216 | 0.19104342 | 0.08937066 | 0.13998525 | BIOCARTA\_HIVNEF\_PATHWAY |
| 3.284682E-5 | 0.0 | 0.0386872 | 0.038418543 | NETPATH\_IL\_7\_PATHWAY\_UP |
| 0.0076145586 | 0.002180355 | 0.13845338 | 0.0064237956 | KEGG\_T\_CELL\_RECEPTOR\_SIGNALING\_PATHWAY |
