# Supplementary material for: Computational approaches for discovery of common immunomodulators in fungal infections: towards broad-spectrum immunotherapeutic interventions
Source: BMC Microbiol. 2013 Oct 7;13:224. doi: 10.1186/1471-2180-13-224 (PMC3853472; doi:10.1186/1471-2180-13-224)
Supplement: Additional file 1 — Details of up- and down- regulated biclusters. [file 1471-2180-13-224-S1.zip › 2013-kidane-bmc/details-of-biclusters/upreg-biclust-52.html]

**BICLUSTER\_ID** : UPREG-52  
**PATHOGENS** /3/ : p. jirovecii,a. fumigatus,c. albicans  
**KNOWN DRUG TARGETS** /1/ : CCL2  

| Gene Set | Leading Edge Genes |
| --- | --- |
| CYTOKINE ACTIVITY | CCL2, CXCL2 |
| NEUROPEPTIDE BINDING |  |
| BIOCARTA INFLAM PATHWAY | IL1A |
| NEUROPEPTIDE RECEPTOR ACTIVITY |  |

| Color legend | | | | | | | | | | | |
| --- | --- | --- | --- | --- | --- | --- | --- | --- | --- | --- | --- |
| q-value | 1 | 0.2 | 0.05 | 0.01 | 0.001 | 0.0001 |
| Color |  | |  |  |  | |

TABLE OF Q-VALUES

| candida albicans huvec | aspergillus fumigatus monocytes | pneumocystis carinnii macrophage | aspergillus fumigatus dendritic | Gene Set |
| --- | --- | --- | --- | --- |
| 0.0049860743 | 0.010335351 | 1.1591674E-4 | 0.0 | CYTOKINE\_ACTIVITY |
| 0.06798881 | 0.1554835 | 0.120724805 | 0.030479442 | NEUROPEPTIDE\_BINDING |
| 0.023040734 | 0.11185193 | 0.14592804 | 5.4974487E-5 | BIOCARTA\_INFLAM\_PATHWAY |
| 0.070704155 | 0.15771186 | 0.12307232 | 0.031832173 | NEUROPEPTIDE\_RECEPTOR\_ACTIVITY |
