# Supplementary material for: Computational approaches for discovery of common immunomodulators in fungal infections: towards broad-spectrum immunotherapeutic interventions
Source: BMC Microbiol. 2013 Oct 7;13:224. doi: 10.1186/1471-2180-13-224 (PMC3853472; doi:10.1186/1471-2180-13-224)
Supplement: Additional file 1 — Details of up- and down- regulated biclusters. [file 1471-2180-13-224-S1.zip › 2013-kidane-bmc/details-of-biclusters/dnreg-biclust-4.html]

**BICLUSTER\_ID** : DNREG-4  
**PATHOGENS** /2/ : a. fumigatus,c. albicans  
**KNOWN DRUG TARGETS** /11/ : NDUFB7, NDUFS6, NDUFS7, NDUFS4, NDUFA3, NDUFA5, NDUFV1, NDUFS2, NDUFB5, SDHD, NDUFC2  

| Gene Set | Leading Edge Genes |
| --- | --- |
| CORUM RESPIRATORY CHAIN COMPLEX I HOLOENZYME MITOCHONDRIAL | NDUFB7, NDUFS6, NDUFS4, NDUFA3, NDUFS7, NDUFA5, NDUFV1, NDUFS2, NDUFB5, NDUFB11, NDUFC2 |
| REACTOME ELECTRON TRANSPORT CHAIN | COX5A, NDUFS7, NDUFA3, NDUFA5, NDUFV1, COX5B, NDUFB5, NDUFB7, ETFB, NDUFS6, NDUFS4, COX4I1, UQCRQ, NDUFS2, UQCRC2, SDHD, ETFA, NDUFB11 |
| INTERLEUKIN 8 BIOSYNTHETIC PROCESS |  |
| KEGG PEROXISOME |  |
| KEGG BUTANOATE METABOLISM |  |
| COENZYME BINDING |  |
| CORUM MULTISYNTHETASE COMPLEX |  |
| REACTOME BRANCHED CHAIN AMINO ACID CATABOLISM |  |
| NCI CYTOSOLIC TRNA AMINOACYLATION |  |
| REACTOME PEPTIDE CHAIN ELONGATION |  |
| REACTOME CYTOSOLIC TRNA AMINOACYLATION |  |
| KEGG VALINE LEUCINE AND ISOLEUCINE DEGRADATION |  |
| KEGG ONE CARBON POOL BY FOLATE |  |
| LYASE ACTIVITY |  |
| INTERLEUKIN 8 PRODUCTION |  |
| COFACTOR BINDING |  |
| KEGG PORPHYRIN AND CHLOROPHYLL METABOLISM |  |

| Color legend | | | | | | | | | | | |
| --- | --- | --- | --- | --- | --- | --- | --- | --- | --- | --- | --- |
| q-value | -1 | -0.2 | -0.05 | -0.01 | -0.001 | -0.0001 |
| Color |  |  |  |  |  |  |

TABLE OF Q-VALUES

| candida albicans moddc135 | aspergillus fumigatus cluture filtrates a549 | Gene Set |
| --- | --- | --- |
| -0.0023720537 | -0.14497164 | CORUM\_RESPIRATORY\_CHAIN\_COMPLEX\_I\_HOLOENZYME\_MITOCHONDRIAL |
| -0.0042326762 | -0.14915366 | REACTOME\_ELECTRON\_TRANSPORT\_CHAIN |
| -0.01802164 | -0.109336585 | INTERLEUKIN\_8\_BIOSYNTHETIC\_PROCESS |
| -0.022583239 | -0.07615189 | KEGG\_PEROXISOME |
| -0.1300732 | -0.007829288 | KEGG\_BUTANOATE\_METABOLISM |
| -0.08973619 | -0.1941193 | COENZYME\_BINDING |
| -0.05327022 | -0.17334767 | CORUM\_MULTISYNTHETASE\_COMPLEX |
| -0.16647543 | -0.047852423 | REACTOME\_BRANCHED\_CHAIN\_AMINO\_ACID\_CATABOLISM |
| -0.027549418 | -0.007089334 | NCI\_CYTOSOLIC\_TRNA\_AMINOACYLATION |
| -0.10028489 | -0.19745262 | REACTOME\_PEPTIDE\_CHAIN\_ELONGATION |
| -0.026814407 | -0.005755359 | REACTOME\_CYTOSOLIC\_TRNA\_AMINOACYLATION |
| -0.037278395 | -0.0014862076 | KEGG\_VALINE\_LEUCINE\_AND\_ISOLEUCINE\_DEGRADATION |
| -0.030288681 | -0.07640898 | KEGG\_ONE\_CARBON\_POOL\_BY\_FOLATE |
| -0.16954342 | -0.15795115 | LYASE\_ACTIVITY |
| -0.06704181 | -0.07360196 | INTERLEUKIN\_8\_PRODUCTION |
| -0.12496564 | -0.119193695 | COFACTOR\_BINDING |
| -0.09468565 | -0.17071082 | KEGG\_PORPHYRIN\_AND\_CHLOROPHYLL\_METABOLISM |
