# Supplementary material for: Computational approaches for discovery of common immunomodulators in fungal infections: towards broad-spectrum immunotherapeutic interventions
Source: BMC Microbiol. 2013 Oct 7;13:224. doi: 10.1186/1471-2180-13-224 (PMC3853472; doi:10.1186/1471-2180-13-224)
Supplement: Additional file 1 — Details of up- and down- regulated biclusters. [file 1471-2180-13-224-S1.zip › 2013-kidane-bmc/details-of-biclusters/dnreg-biclust-6.html]

**BICLUSTER\_ID** : DNREG-6  
**PATHOGENS** /2/ : c. albicans,a. fumigatus  
**KNOWN DRUG TARGETS** /7/ : NDUFB7, NDUFS6, NDUFS7, NDUFS4, NDUFB5, NDUFA3, SDHD  

| Gene Set | Leading Edge Genes |
| --- | --- |
| REACTOME ELECTRON TRANSPORT CHAIN | ETFB, NDUFB7, NDUFS6, NDUFS4, NDUFS7, NDUFA3, COX4I1, UQCRQ, NDUFB5, COX5B, SDHD, NDUFB11 |
| KEGG BUTANOATE METABOLISM |  |
| KEGG PEROXISOME |  |
| KEGG PORPHYRIN AND CHLOROPHYLL METABOLISM |  |
| REACTOME PEPTIDE CHAIN ELONGATION |  |

| Color legend | | | | | | | | | | | |
| --- | --- | --- | --- | --- | --- | --- | --- | --- | --- | --- | --- |
| q-value | -1 | -0.2 | -0.05 | -0.01 | -0.001 | -0.0001 |
| Color |  |  |  |  |  |  |

TABLE OF Q-VALUES

| aspergillus fumigatus conidia a549 | candida albicans moddc135 | aspergillus fumigatus cluture filtrates a549 | Gene Set |
| --- | --- | --- | --- |
| -0.0 | -0.0042326762 | -0.14915366 | REACTOME\_ELECTRON\_TRANSPORT\_CHAIN |
| -0.15030144 | -0.1300732 | -0.007829288 | KEGG\_BUTANOATE\_METABOLISM |
| -0.017353417 | -0.022583239 | -0.07615189 | KEGG\_PEROXISOME |
| -0.14548959 | -0.09468565 | -0.17071082 | KEGG\_PORPHYRIN\_AND\_CHLOROPHYLL\_METABOLISM |
| -0.0 | -0.10028489 | -0.19745262 | REACTOME\_PEPTIDE\_CHAIN\_ELONGATION |
