# Supplementary material for: Computational approaches for discovery of common immunomodulators in fungal infections: towards broad-spectrum immunotherapeutic interventions
Source: BMC Microbiol. 2013 Oct 7;13:224. doi: 10.1186/1471-2180-13-224 (PMC3853472; doi:10.1186/1471-2180-13-224)
Supplement: Additional file 1 — Details of up- and down- regulated biclusters. [file 1471-2180-13-224-S1.zip › 2013-kidane-bmc/details-of-biclusters/upreg-biclust-34.html]

**BICLUSTER\_ID** : UPREG-34  
**PATHOGENS** /2/ : p. jirovecii,a. fumigatus  
**KNOWN DRUG TARGETS** /9/ : PLAU, NFKB1, STAT1, IL6, SERPINE1, IFNGR1, CCL2, TFPI, PLAUR  

| Gene Set | Leading Edge Genes |
| --- | --- |
| RESPONSE TO EXTERNAL STIMULUS | NFKB1, IL1RAP, CXCL1, PIK3CB, CCL2, TFPI, IL1A, PLAU, SERPINE1, CXCL2, PLAUR |
| NCI IL23PATHWAY | NFKB1, NFKBIA, STAT1, IL6, CXCL1, CCL2 |
| KEGG CYTOKINE CYTOKINE RECEPTOR INTERACTION | IL6, IFNGR1, IL1RAP, CXCL1, CCL2, CXCL2, IL1A |
| BEHAVIOR | PLAU, CXCL1, CCL2, CXCL2, PLAUR |
| LOCOMOTORY BEHAVIOR | PLAU, CXCL1, CCL2, CXCL2, PLAUR |
| RESPONSE TO WOUNDING | NFKB1, SERPINE1, CXCL1, IL1RAP, CXCL2, IL1A, TFPI |
| CHEMOKINE ACTIVITY | CXCL1, CCL2, CXCL2 |
| PEPTIDYL TYROSINE PHOSPHORYLATION | STAT1 |
| G PROTEIN COUPLED RECEPTOR BINDING | CXCL1, CCL2, CXCL2 |
| REACTOME CLASS A1 RHODOPSIN LIKE RECEPTORS | CXCL1, CCL2, EDN1, CXCL2 |
| DEFENSE RESPONSE | NFKB1, CXCL1, IL1RAP, CXCL2, IL1A |
| INFLAMMATORY RESPONSE | NFKB1, CXCL1, IL1RAP, CXCL2, IL1A |
| REACTOME PEPTIDE LIGAND BINDING RECEPTORS | CXCL1, CCL2, EDN1, CXCL2 |
| PEPTIDYL TYROSINE MODIFICATION | STAT1, TPST2 |
| REACTOME GPCR LIGAND BINDING | CXCL1, EDN1, CCL2, CXCL2 |
| EXTRACELLULAR SPACE | CXCL1, CCL2, EDN1, CXCL2 |
| CHEMOKINE RECEPTOR BINDING | CXCL1, CCL2, CXCL2 |
| CYTOKINE ACTIVITY | CXCL1, CCL2, CXCL2 |
| BIOCARTA INFLAM PATHWAY | IL6, IL1A |
| BIOCARTA NKT PATHWAY | IFNGR1 |
| REACTOME CHEMOKINE RECEPTORS BIND CHEMOKINES | CXCL1, CCL2, CXCL2 |
| HEART DEVELOPMENT |  |
| KEGG HEMATOPOIETIC CELL LINEAGE |  |
| REACTOME NA CL DEPENDENT NEUROTRANSMITTER TRANSPORTERS |  |
| REACTOME G ALPHA I SIGNALLING EVENTS | CXCL1, CXCL2 |
| PROTEIN TYROSINE KINASE ACTIVITY |  |
| EXTRACELLULAR REGION PART |  |

| Color legend | | | | | | | | | | | |
| --- | --- | --- | --- | --- | --- | --- | --- | --- | --- | --- | --- |
| q-value | 1 | 0.2 | 0.05 | 0.01 | 0.001 | 0.0001 |
| Color |  | |  |  |  | |

TABLE OF Q-VALUES

| aspergillus fumigatus conidia a549 | pneumocystis carinnii macrophage | Gene Set |
| --- | --- | --- |
| 6.960372E-4 | 0.14160725 | RESPONSE\_TO\_EXTERNAL\_STIMULUS |
| 1.4370484E-5 | 0.19927236 | NCI\_IL23PATHWAY |
| 2.5672338E-5 | 0.016364018 | KEGG\_CYTOKINE\_CYTOKINE\_RECEPTOR\_INTERACTION |
| 1.642341E-5 | 0.15872823 | BEHAVIOR |
| 2.2720385E-5 | 0.108142614 | LOCOMOTORY\_BEHAVIOR |
| 0.0013995847 | 0.04277774 | RESPONSE\_TO\_WOUNDING |
| 2.8400484E-5 | 0.041768454 | CHEMOKINE\_ACTIVITY |
| 0.0517779 | 0.15985331 | PEPTIDYL\_TYROSINE\_PHOSPHORYLATION |
| 1.8933655E-5 | 0.017283333 | G\_PROTEIN\_COUPLED\_RECEPTOR\_BINDING |
| 0.0015525026 | 1.738751E-4 | REACTOME\_CLASS\_A1\_RHODOPSIN\_LIKE\_RECEPTORS |
| 0.005702261 | 0.04436318 | DEFENSE\_RESPONSE |
| 6.0851693E-5 | 0.048219085 | INFLAMMATORY\_RESPONSE |
| 1.9160645E-5 | 2.594948E-4 | REACTOME\_PEPTIDE\_LIGAND\_BINDING\_RECEPTORS |
| 0.04039933 | 0.10861157 | PEPTIDYL\_TYROSINE\_MODIFICATION |
| 0.06493268 | 0.014167708 | REACTOME\_GPCR\_LIGAND\_BINDING |
| 0.07053614 | 0.033668406 | EXTRACELLULAR\_SPACE |
| 0.0 | 0.04107923 | CHEMOKINE\_RECEPTOR\_BINDING |
| 2.0902522E-5 | 1.1591674E-4 | CYTOKINE\_ACTIVITY |
| 0.01884623 | 0.14592804 | BIOCARTA\_INFLAM\_PATHWAY |
| 0.11278513 | 0.106201544 | BIOCARTA\_NKT\_PATHWAY |
| 0.0 | 0.016418632 | REACTOME\_CHEMOKINE\_RECEPTORS\_BIND\_CHEMOKINES |
| 0.13906007 | 0.14578117 | HEART\_DEVELOPMENT |
| 0.14205706 | 0.14391063 | KEGG\_HEMATOPOIETIC\_CELL\_LINEAGE |
| 0.13590625 | 0.046032816 | REACTOME\_NA\_CL\_DEPENDENT\_NEUROTRANSMITTER\_TRANSPORTERS |
| 0.017489815 | 0.030884687 | REACTOME\_G\_ALPHA\_I\_SIGNALLING\_EVENTS |
| 0.08512424 | 0.1764927 | PROTEIN\_TYROSINE\_KINASE\_ACTIVITY |
| 0.13260934 | 0.062482 | EXTRACELLULAR\_REGION\_PART |
