# Supplementary material for: Computational approaches for discovery of common immunomodulators in fungal infections: towards broad-spectrum immunotherapeutic interventions
Source: BMC Microbiol. 2013 Oct 7;13:224. doi: 10.1186/1471-2180-13-224 (PMC3853472; doi:10.1186/1471-2180-13-224)
Supplement: Additional file 1 — Details of up- and down- regulated biclusters. [file 1471-2180-13-224-S1.zip › 2013-kidane-bmc/details-of-biclusters/upreg-biclust-36.html]

**BICLUSTER\_ID** : UPREG-36  
**PATHOGENS** /5/ : p. jirovecii,a. alternata,a. fumigatus,c. albicans,s. chartarum  
**KNOWN DRUG TARGETS** /0/ :   

| Gene Set | Leading Edge Genes |
| --- | --- |
| CHEMOKINE ACTIVITY | CXCL1, CXCL2 |
| G PROTEIN COUPLED RECEPTOR BINDING | CXCL1, CXCL2 |
| CYTOKINE ACTIVITY | CXCL1, CXCL2 |
| CHEMOKINE RECEPTOR BINDING | CXCL1, CXCL2 |
| REACTOME CHEMOKINE RECEPTORS BIND CHEMOKINES | CXCL1, CXCL2 |
| REACTOME PEPTIDE LIGAND BINDING RECEPTORS | CXCL1, CXCL2 |

| Color legend | | | | | | | | | | | |
| --- | --- | --- | --- | --- | --- | --- | --- | --- | --- | --- | --- |
| q-value | 1 | 0.2 | 0.05 | 0.01 | 0.001 | 0.0001 |
| Color |  | |  |  |  | |

TABLE OF Q-VALUES

| candida albicans huvec | aspergillus fumigatus conidia a549 | candida albicans moddc135 | alternaria alternata beas2b | pneumocystis carinnii macrophage | candida albicans neutrophils | aspergillus fumigatus dendritic | stachybotrys chartarum lung | Gene Set |
| --- | --- | --- | --- | --- | --- | --- | --- | --- |
| 0.0024792356 | 2.8400484E-5 | 0.0 | 0.0 | 0.041768454 | 0.091335885 | 3.546737E-5 | 7.375062E-5 | CHEMOKINE\_ACTIVITY |
| 0.08719925 | 1.8933655E-5 | 0.0 | 9.6749085E-5 | 0.017283333 | 0.1173318 | 6.563468E-5 | 1.8657849E-4 | G\_PROTEIN\_COUPLED\_RECEPTOR\_BINDING |
| 0.0049860743 | 2.0902522E-5 | 0.0 | 0.0 | 1.1591674E-4 | 7.5262925E-4 | 0.0 | 0.012438191 | CYTOKINE\_ACTIVITY |
| 0.0022271401 | 0.0 | 0.0 | 0.0 | 0.04107923 | 0.08899127 | 2.8777304E-5 | 7.93557E-5 | CHEMOKINE\_RECEPTOR\_BINDING |
| 0.0 | 0.0 | 0.0 | 0.0 | 0.016418632 | 0.17955464 | 0.0 | 5.5177166E-5 | REACTOME\_CHEMOKINE\_RECEPTORS\_BIND\_CHEMOKINES |
| 0.015921747 | 1.9160645E-5 | 0.0 | 0.0 | 2.594948E-4 | 0.10786476 | 0.0 | 8.2969455E-5 | REACTOME\_PEPTIDE\_LIGAND\_BINDING\_RECEPTORS |
