# Supplementary material for: Computational approaches for discovery of common immunomodulators in fungal infections: towards broad-spectrum immunotherapeutic interventions
Source: BMC Microbiol. 2013 Oct 7;13:224. doi: 10.1186/1471-2180-13-224 (PMC3853472; doi:10.1186/1471-2180-13-224)
Supplement: Additional file 1 — Details of up- and down- regulated biclusters. [file 1471-2180-13-224-S1.zip › 2013-kidane-bmc/details-of-biclusters/upreg-biclust-38.html]

**BICLUSTER\_ID** : UPREG-38  
**PATHOGENS** /3/ : p. jirovecii,a. fumigatus,c. albicans  
**KNOWN DRUG TARGETS** /3/ : NFKB1, CCL2, PLAUR  

| Gene Set | Leading Edge Genes |
| --- | --- |
| LOCOMOTORY BEHAVIOR | CXCL1, CCL2, PLAUR |
| KEGG CYTOKINE CYTOKINE RECEPTOR INTERACTION | IL4R, CXCL1, CCL2, CXCL2 |
| CHEMOKINE ACTIVITY | CXCL1, CCL2 |
| CHEMOKINE RECEPTOR BINDING | CXCL1, CCL2 |
| KEGG CYTOSOLIC DNA SENSING PATHWAY | NFKB1, NFKBIA |
| REACTOME CHEMOKINE RECEPTORS BIND CHEMOKINES | CXCL1, CCL2 |
| BIOCARTA ASBCELL PATHWAY |  |
| KEGG HEMATOPOIETIC CELL LINEAGE | IL4R, CD38 |
| KEGG ECM RECEPTOR INTERACTION | SDC4 |
| BIOCARTA INFLAM PATHWAY |  |

| Color legend | | | | | | | | | | | |
| --- | --- | --- | --- | --- | --- | --- | --- | --- | --- | --- | --- |
| q-value | 1 | 0.2 | 0.05 | 0.01 | 0.001 | 0.0001 |
| Color |  | |  |  |  | |

TABLE OF Q-VALUES

| candida albicans moddc135 | pneumocystis carinnii macrophage | aspergillus fumigatus cluture filtrates a549 | aspergillus fumigatus dendritic | Gene Set |
| --- | --- | --- | --- | --- |
| 3.2139455E-6 | 0.108142614 | 0.064572826 | 0.04213685 | LOCOMOTORY\_BEHAVIOR |
| 0.0 | 0.016364018 | 0.050987493 | 0.0 | KEGG\_CYTOKINE\_CYTOKINE\_RECEPTOR\_INTERACTION |
| 0.0 | 0.041768454 | 0.13061193 | 3.546737E-5 | CHEMOKINE\_ACTIVITY |
| 0.0 | 0.04107923 | 0.12956315 | 2.8777304E-5 | CHEMOKINE\_RECEPTOR\_BINDING |
| 1.3989894E-4 | 0.16132888 | 0.13053004 | 0.15817112 | KEGG\_CYTOSOLIC\_DNA\_SENSING\_PATHWAY |
| 0.0 | 0.016418632 | 0.0 | 0.0 | REACTOME\_CHEMOKINE\_RECEPTORS\_BIND\_CHEMOKINES |
| 0.0067488668 | 0.1606871 | 0.1806629 | 0.16922034 | BIOCARTA\_ASBCELL\_PATHWAY |
| 0.0 | 0.14391063 | 0.17998308 | 0.001619189 | KEGG\_HEMATOPOIETIC\_CELL\_LINEAGE |
| 0.009347258 | 0.12158026 | 0.132169 | 0.0019129739 | KEGG\_ECM\_RECEPTOR\_INTERACTION |
| 0.0 | 0.14592804 | 0.0013572491 | 5.4974487E-5 | BIOCARTA\_INFLAM\_PATHWAY |
