# Supplementary material for: Computational approaches for discovery of common immunomodulators in fungal infections: towards broad-spectrum immunotherapeutic interventions
Source: BMC Microbiol. 2013 Oct 7;13:224. doi: 10.1186/1471-2180-13-224 (PMC3853472; doi:10.1186/1471-2180-13-224)
Supplement: Additional file 1 — Details of up- and down- regulated biclusters. [file 1471-2180-13-224-S1.zip › 2013-kidane-bmc/details-of-biclusters/upreg-biclust-40.html]

**BICLUSTER\_ID** : UPREG-40  
**PATHOGENS** /2/ : c. albicans,s. chartarum  
**KNOWN DRUG TARGETS** /19/ : PLA2G4A, HEXB, CTSD, CCL20, CD44, SERPINB2, GLA, PTGIR, CTNS, CCL5, PIM1, P2RY2, NEU1, PTGER4, NP, CTSB, IL1B, PTGER2, ALOX15  

| Gene Set | Leading Edge Genes |
| --- | --- |
| REACTOME CLASS A1 RHODOPSIN LIKE RECEPTORS | PTGER4, CCL20, CXCL1, CCR1, PTGIR, C3AR1, CCL5, PTGER2, CXCL2, P2RY2, CCL4 |
| KEGG LYSOSOME | HEXB, CD68, CTSD, CLN3, ATP6V0B, GNS, MCOLN1, GLA, CTSB, CTNS, HEXA, CLN5, NEU1, ATP6V0C |
| NETPATH IL 1 PATHWAY UP | CCL20, CXCL1, NFKBIE, NP, SERPINB2, CKS2, NFKBIA, IL1B, ZFP36, CXCL2, GJB1, CCL4 |
| CHEMOKINE ACTIVITY | CCL20, CXCL1, CCL5, CXCL2, CCL4 |
| NETPATH IL 3 PATHWAY UP | NFIL3, SOCS3, IL1B, CD69, FOS, PIM1, CCR1 |
| KEGG LINOLEIC ACID METABOLISM | PLA2G4A, ALOX15 |
| G PROTEIN COUPLED RECEPTOR BINDING | CCL20, CXCL1, CCL5, CXCL2, CCL4 |
| REACTOME PEPTIDE LIGAND BINDING RECEPTORS | CCL20, CXCL1, C3AR1, CCL5, CXCL2, CCR1, CCL4 |
| CHEMOKINE RECEPTOR BINDING | CCL20, CXCL1, CCL5, CXCL2, CCL4 |
| CYTOKINE ACTIVITY | IL1RN, CCL20, CXCL1, CCL5, CXCL2, CSF2, CCL4 |
| NETPATH IL 7 PATHWAY UP | LITAF, CXCL1, CCL5, CD44, CXCL2, CCL4 |
| REACTOME CHEMOKINE RECEPTORS BIND CHEMOKINES | CCL20, CXCL1, CCL5, CXCL2, CCL4, CCR1 |
| NEGATIVE REGULATION OF CELLULAR BIOSYNTHETIC PROCESS | BRCA1, GLA |
| NEGATIVE REGULATION OF BIOSYNTHETIC PROCESS | BRCA1, GLA |
| NCI NFAT TFPATHWAY | EGR3, EGR1, JUNB, FOS, EGR2 |
| KEGG ASTHMA |  |
| REGULATION OF HORMONE SECRETION |  |

| Color legend | | | | | | | | | | | |
| --- | --- | --- | --- | --- | --- | --- | --- | --- | --- | --- | --- |
| q-value | 1 | 0.2 | 0.05 | 0.01 | 0.001 | 0.0001 |
| Color |  | |  |  |  | |

TABLE OF Q-VALUES

| candida albicans neutrophils | stachybotrys chartarum lung | Gene Set |
| --- | --- | --- |
| 0.17791544 | 2.021267E-4 | REACTOME\_CLASS\_A1\_RHODOPSIN\_LIKE\_RECEPTORS |
| 0.17728922 | 2.7588583E-5 | KEGG\_LYSOSOME |
| 0.19693953 | 0.0027125722 | NETPATH\_IL\_1\_PATHWAY\_UP |
| 0.091335885 | 7.375062E-5 | CHEMOKINE\_ACTIVITY |
| 0.10933371 | 3.9366656E-4 | NETPATH\_IL\_3\_PATHWAY\_UP |
| 0.09112359 | 0.1388137 | KEGG\_LINOLEIC\_ACID\_METABOLISM |
| 0.1173318 | 1.8657849E-4 | G\_PROTEIN\_COUPLED\_RECEPTOR\_BINDING |
| 0.10786476 | 8.2969455E-5 | REACTOME\_PEPTIDE\_LIGAND\_BINDING\_RECEPTORS |
| 0.08899127 | 7.93557E-5 | CHEMOKINE\_RECEPTOR\_BINDING |
| 7.5262925E-4 | 0.012438191 | CYTOKINE\_ACTIVITY |
| 0.061718952 | 0.038418543 | NETPATH\_IL\_7\_PATHWAY\_UP |
| 0.17955464 | 5.5177166E-5 | REACTOME\_CHEMOKINE\_RECEPTORS\_BIND\_CHEMOKINES |
| 0.025412852 | 0.15733238 | NEGATIVE\_REGULATION\_OF\_CELLULAR\_BIOSYNTHETIC\_PROCESS |
| 0.022074154 | 0.15170847 | NEGATIVE\_REGULATION\_OF\_BIOSYNTHETIC\_PROCESS |
| 0.035003204 | 0.06361532 | NCI\_NFAT\_TFPATHWAY |
| 0.021450594 | 0.09246591 | KEGG\_ASTHMA |
| 0.10907822 | 0.16944346 | REGULATION\_OF\_HORMONE\_SECRETION |
