# Supplementary material for: Computational approaches for discovery of common immunomodulators in fungal infections: towards broad-spectrum immunotherapeutic interventions
Source: BMC Microbiol. 2013 Oct 7;13:224. doi: 10.1186/1471-2180-13-224 (PMC3853472; doi:10.1186/1471-2180-13-224)
Supplement: Additional file 1 — Details of up- and down- regulated biclusters. [file 1471-2180-13-224-S1.zip › 2013-kidane-bmc/details-of-biclusters/upreg-biclust-42.html]

**BICLUSTER\_ID** : UPREG-42  
**PATHOGENS** /2/ : c. albicans,a. fumigatus  
**KNOWN DRUG TARGETS** /27/ : LDLR, BCL2, SLC1A4, CCL2, GLA, CDK4, SLC1A3, IL3RA, SLC7A11, UGCG, PIM1, IL2RA, IFNG, CXCL10, PTPN1, STAT1, IL12B, MMP9, ABCC1, CCL8, IL6R, PPARG, SMPD1, IL1B, MET, IL8, TNF  

| Gene Set | Leading Edge Genes |
| --- | --- |
| NETPATH IL 3 PATHWAY UP | BCL2, BCL2L1, CCL2, SPP1, SOCS3, IL3RA, MATK, CCL7, PIM1, CCL4, CSF1, IL6R, USP36, IL1B, OSM, IL8, SOCS2, TNF |
| NCI NFAT TFPATHWAY | PTPN1, GBP3, PPARG, CDK4, FOSL1, CBLB, IL8, IFNG, IL2RA, CSF2, TNF |
| NETPATH IL 6 PATHWAY UP | GADD45B, LDLR, SPP1, HBEGF, SLC39A14, SOCS3, RHOBTB3, PIM1, STAT1, ABCC1, MMP9, PPARG, MAFF, STAT3, EIF5, IRF1, SOCS2, TNF |
| CYTOKINE ACTIVITY | SDCBP, CCL2, CXCL3, CCL7, IL1RN, INHBA, CXCL2, CSF2, CXCL10, CCL4, CSF1, IL1F5, CCL8, CXCL5, OSM, IL8, TNF |
| KEGG SPHINGOLIPID METABOLISM | SMPD1, SPHK1, UGCG, NEU4, GLA, SGPP2 |
| REACTOME AMINO ACID AND OLIGOPEPTIDE SLC TRANSPORTERS | SLC1A3, SLC7A11, SLC6A6, SLC16A10, SLC1A4, SLC15A4 |
| BIOCARTA INFLAM PATHWAY | IL12B, PDGFA, CSF1, IL8, CSF2, IL1A, TNF |
| KEGG EPITHELIAL CELL SIGNALING IN HELICOBACTER PYLORI INFECTION | ATP6V1H, TCIRG1, HBEGF, ATP6V1D, ADAM17, SRC, ATP6V0A1, MET, MAPK13, IL8, ATP6V0D2, MAPK8 |
| HEMATOPOIETIN INTERFERON CLASSD200 DOMAIN CYTOKINE RECEPTOR BINDING | SDCBP, IL12B, IFNG, OSM, CSF2 |
| MONOOXYGENASE ACTIVITY |  |
| AMINO ACID TRANSMEMBRANE TRANSPORTER ACTIVITY |  |
| BIOCARTA PLATELETAPP PATHWAY |  |
| REACTOME ENDOGENOUS STEROLS |  |
| AMINO SUGAR METABOLIC PROCESS |  |
| AMINO ACID TRANSPORT |  |
| AMINE TRANSMEMBRANE TRANSPORTER ACTIVITY |  |
| NCI ENDOGENOUS STEROLS |  |

| Color legend | | | | | | | | | | | |
| --- | --- | --- | --- | --- | --- | --- | --- | --- | --- | --- | --- |
| q-value | 1 | 0.2 | 0.05 | 0.01 | 0.001 | 0.0001 |
| Color |  | |  |  |  | |

TABLE OF Q-VALUES

| aspergillus fumigatus monocytes | candida albicans moddc135 | Gene Set |
| --- | --- | --- |
| 0.009785512 | 0.0 | NETPATH\_IL\_3\_PATHWAY\_UP |
| 0.19765195 | 5.8961764E-4 | NCI\_NFAT\_TFPATHWAY |
| 0.11932011 | 0.0 | NETPATH\_IL\_6\_PATHWAY\_UP |
| 0.010335351 | 0.0 | CYTOKINE\_ACTIVITY |
| 0.10197773 | 0.048068393 | KEGG\_SPHINGOLIPID\_METABOLISM |
| 0.08670611 | 0.01380212 | REACTOME\_AMINO\_ACID\_AND\_OLIGOPEPTIDE\_SLC\_TRANSPORTERS |
| 0.11185193 | 0.0 | BIOCARTA\_INFLAM\_PATHWAY |
| 0.15699872 | 0.002267632 | KEGG\_EPITHELIAL\_CELL\_SIGNALING\_IN\_HELICOBACTER\_PYLORI\_INFECTION |
| 0.04804973 | 8.233354E-5 | HEMATOPOIETIN\_INTERFERON\_CLASSD200\_DOMAIN\_CYTOKINE\_RECEPTOR\_BINDING |
| 0.09482384 | 0.07396551 | MONOOXYGENASE\_ACTIVITY |
| 0.092043035 | 0.117709376 | AMINO\_ACID\_TRANSMEMBRANE\_TRANSPORTER\_ACTIVITY |
| 0.14451481 | 0.098546006 | BIOCARTA\_PLATELETAPP\_PATHWAY |
| 0.1503794 | 0.1172772 | REACTOME\_ENDOGENOUS\_STEROLS |
| 0.11079641 | 0.055010613 | AMINO\_SUGAR\_METABOLIC\_PROCESS |
| 0.16387926 | 0.13234565 | AMINO\_ACID\_TRANSPORT |
| 0.15825975 | 0.061027322 | AMINE\_TRANSMEMBRANE\_TRANSPORTER\_ACTIVITY |
| 0.16779399 | 0.19913463 | NCI\_ENDOGENOUS\_STEROLS |
