# Supplementary material for: Computational approaches for discovery of common immunomodulators in fungal infections: towards broad-spectrum immunotherapeutic interventions
Source: BMC Microbiol. 2013 Oct 7;13:224. doi: 10.1186/1471-2180-13-224 (PMC3853472; doi:10.1186/1471-2180-13-224)
Supplement: Additional file 1 — Details of up- and down- regulated biclusters. [file 1471-2180-13-224-S1.zip › 2013-kidane-bmc/details-of-biclusters/upreg-biclust-44.html]

**BICLUSTER\_ID** : UPREG-44  
**PATHOGENS** /2/ : p. jirovecii,c. albicans  
**KNOWN DRUG TARGETS** /10/ : PF4, ANXA1, AGT, PLAU, HTR4, IL1B, CCL5, PTGER2, ADORA2A, TNF  

| Gene Set | Leading Edge Genes |
| --- | --- |
| REACTOME CLASS A1 RHODOPSIN LIKE RECEPTORS | ANXA1, PF4, CXCL1, EDN1, C5AR1, AGT, CCR1, CCL5, PTGER2, CXCL2, ADORA2A, CCL4 |
| REACTOME PEPTIDE LIGAND BINDING RECEPTORS | ANXA1, PF4, CXCL1, C5AR1, EDN1, AGT, CCR1, C3AR1, CCL5, CXCL2, CCL4 |
| CYTOKINE ACTIVITY | PF4, CSF1, CXCL1, IL1RN, CCL5, CXCL2, TNF, CCL4 |
| CHEMOKINE ACTIVITY | PF4, CXCL1, CCL5, CXCL2, CCL4 |
| CHEMOKINE RECEPTOR BINDING | PF4, CXCL1, CCL5, CXCL2, CCL4 |
| G PROTEIN COUPLED RECEPTOR BINDING | PF4, CXCL1, CCL5, CXCL2, CCL4 |
| RHODOPSIN LIKE RECEPTOR ACTIVITY | HTR4, C3AR1, C5AR1, PTGER2 |
| REACTOME CHEMOKINE RECEPTORS BIND CHEMOKINES | PF4, CXCL1, CCL5, CXCL2, CCL4, CCR1 |
| REACTOME AMINE LIGAND BINDING RECEPTORS | HTR4 |
| NETPATH WNT PATHWAY DOWN | CXCL1, IL1B |
| NCI PEPTIDE LIGAND BINDING RECEPTORS | C5AR1, AGT |
| AMINE RECEPTOR ACTIVITY | HTR4 |
| BIOCARTA FIBRINOLYSIS PATHWAY | PLAU |
| KEGG ASTHMA | HLA-DMA, TNF |
| BIOCARTA AMI PATHWAY |  |

| Color legend | | | | | | | | | | | |
| --- | --- | --- | --- | --- | --- | --- | --- | --- | --- | --- | --- |
| q-value | 1 | 0.2 | 0.05 | 0.01 | 0.001 | 0.0001 |
| Color |  | |  |  |  | |

TABLE OF Q-VALUES

| pneumocystis carinnii macrophage | candida albicans neutrophils | Gene Set |
| --- | --- | --- |
| 1.738751E-4 | 0.17791544 | REACTOME\_CLASS\_A1\_RHODOPSIN\_LIKE\_RECEPTORS |
| 2.594948E-4 | 0.10786476 | REACTOME\_PEPTIDE\_LIGAND\_BINDING\_RECEPTORS |
| 1.1591674E-4 | 7.5262925E-4 | CYTOKINE\_ACTIVITY |
| 0.041768454 | 0.091335885 | CHEMOKINE\_ACTIVITY |
| 0.04107923 | 0.08899127 | CHEMOKINE\_RECEPTOR\_BINDING |
| 0.017283333 | 0.1173318 | G\_PROTEIN\_COUPLED\_RECEPTOR\_BINDING |
| 0.04085593 | 0.03802937 | RHODOPSIN\_LIKE\_RECEPTOR\_ACTIVITY |
| 0.016418632 | 0.17955464 | REACTOME\_CHEMOKINE\_RECEPTORS\_BIND\_CHEMOKINES |
| 0.12571035 | 0.048742972 | REACTOME\_AMINE\_LIGAND\_BINDING\_RECEPTORS |
| 0.14159183 | 0.053037744 | NETPATH\_WNT\_PATHWAY\_DOWN |
| 0.17807059 | 0.14927945 | NCI\_PEPTIDE\_LIGAND\_BINDING\_RECEPTORS |
| 0.12090592 | 0.033233523 | AMINE\_RECEPTOR\_ACTIVITY |
| 0.17358682 | 0.17570677 | BIOCARTA\_FIBRINOLYSIS\_PATHWAY |
| 0.0478591 | 0.021450594 | KEGG\_ASTHMA |
| 0.17694005 | 0.13676883 | BIOCARTA\_AMI\_PATHWAY |
