# Supplementary material for: Computational approaches for discovery of common immunomodulators in fungal infections: towards broad-spectrum immunotherapeutic interventions
Source: BMC Microbiol. 2013 Oct 7;13:224. doi: 10.1186/1471-2180-13-224 (PMC3853472; doi:10.1186/1471-2180-13-224)
Supplement: Additional file 1 — Details of up- and down- regulated biclusters. [file 1471-2180-13-224-S1.zip › 2013-kidane-bmc/details-of-biclusters/upreg-biclust-46.html]

**BICLUSTER\_ID** : UPREG-46  
**PATHOGENS** /4/ : a. alternata,a. fumigatus,c. albicans,s. chartarum  
**KNOWN DRUG TARGETS** /1/ : CCL2  

| Gene Set | Leading Edge Genes |
| --- | --- |
| CYTOKINE ACTIVITY | CCL2, CXCL2 |
| NETPATH IL 3 PATHWAY UP | CCL2 |
| NETPATH IL 6 PATHWAY UP | MAFF |
| NCI NFAT TFPATHWAY |  |

| Color legend | | | | | | | | | | | |
| --- | --- | --- | --- | --- | --- | --- | --- | --- | --- | --- | --- |
| q-value | 1 | 0.2 | 0.05 | 0.01 | 0.001 | 0.0001 |
| Color |  | |  |  |  | |

TABLE OF Q-VALUES

| candida albicans huvec | aspergillus fumigatus conidia a549 | aspergillus fumigatus monocytes | candida albicans moddc135 | alternaria alternata beas2b | aspergillus fumigatus dendritic | stachybotrys chartarum lung | Gene Set |
| --- | --- | --- | --- | --- | --- | --- | --- |
| 0.0049860743 | 2.0902522E-5 | 0.010335351 | 0.0 | 0.0 | 0.0 | 0.012438191 | CYTOKINE\_ACTIVITY |
| 0.04616931 | 2.8740968E-5 | 0.009785512 | 0.0 | 0.0027465345 | 5.743034E-5 | 3.9366656E-4 | NETPATH\_IL\_3\_PATHWAY\_UP |
| 0.0011679985 | 3.6444217E-5 | 0.11932011 | 0.0 | 0.004494796 | 5.405209E-5 | 0.010593823 | NETPATH\_IL\_6\_PATHWAY\_UP |
| 0.031072352 | 1.4434879E-4 | 0.19765195 | 5.8961764E-4 | 0.0028159413 | 1.962291E-4 | 0.06361532 | NCI\_NFAT\_TFPATHWAY |
