# Supplementary material for: Computational approaches for discovery of common immunomodulators in fungal infections: towards broad-spectrum immunotherapeutic interventions
Source: BMC Microbiol. 2013 Oct 7;13:224. doi: 10.1186/1471-2180-13-224 (PMC3853472; doi:10.1186/1471-2180-13-224)
Supplement: Additional file 1 — Details of up- and down- regulated biclusters. [file 1471-2180-13-224-S1.zip › 2013-kidane-bmc/details-of-biclusters/upreg-biclust-48.html]

**BICLUSTER\_ID** : UPREG-48  
**PATHOGENS** /2/ : a. fumigatus,c. albicans  
**KNOWN DRUG TARGETS** /1/ : IL8  

| Gene Set | Leading Edge Genes |
| --- | --- |
| NCI NFAT TFPATHWAY | FOSL1, IL8 |
| BIOCARTA INFLAM PATHWAY | PDGFA, IL8 |
| NCI ENDOGENOUS STEROLS |  |
| REACTOME ENDOGENOUS STEROLS |  |

| Color legend | | | | | | | | | | | |
| --- | --- | --- | --- | --- | --- | --- | --- | --- | --- | --- | --- |
| q-value | 1 | 0.2 | 0.05 | 0.01 | 0.001 | 0.0001 |
| Color |  | |  |  |  | |

TABLE OF Q-VALUES

| candida albicans huvec | aspergillus fumigatus monocytes | candida albicans moddc135 | aspergillus fumigatus cluture filtrates a549 | Gene Set |
| --- | --- | --- | --- | --- |
| 0.031072352 | 0.19765195 | 5.8961764E-4 | 0.14430721 | NCI\_NFAT\_TFPATHWAY |
| 0.023040734 | 0.11185193 | 0.0 | 0.0013572491 | BIOCARTA\_INFLAM\_PATHWAY |
| 0.09456388 | 0.16779399 | 0.19913463 | 0.10544677 | NCI\_ENDOGENOUS\_STEROLS |
| 0.09555835 | 0.1503794 | 0.1172772 | 0.106379606 | REACTOME\_ENDOGENOUS\_STEROLS |
