# Supplementary material for: Computational approaches for discovery of common immunomodulators in fungal infections: towards broad-spectrum immunotherapeutic interventions
Source: BMC Microbiol. 2013 Oct 7;13:224. doi: 10.1186/1471-2180-13-224 (PMC3853472; doi:10.1186/1471-2180-13-224)
Supplement: Additional file 1 — Details of up- and down- regulated biclusters. [file 1471-2180-13-224-S1.zip › 2013-kidane-bmc/details-of-biclusters/upreg-biclust-4.html]

**BICLUSTER\_ID** : UPREG-4  
**PATHOGENS** /2/ : a. alternata,c. albicans  
**KNOWN DRUG TARGETS** /46/ : PLAT, BCAT1, SLC7A11, GFPT2, ISG20, MC1R, TGM2, CXCL10, CFB, GPRC5A, PPIF, IL6R, SLC22A4, CHRNB2, SERPINE1, MET, IL1B, F3, ICAM1, SMOX, EPHA2, CCL2, CD40, CASP7, CCND1, CCL5, CD55, ADORA2B, GP1BA, PIM1, SERPINA1, STAT1, NRP1, WARS, TAP1, INPP1, NP, NR3C1, TOP1, TFPI, IFNB1, KCNN4, IL6, PTGER2, IL8, PLAUR  

| Gene Set | Leading Edge Genes |
| --- | --- |
| SIGNAL TRANSDUCTION | BAI1, RRAGC, EREG, OPTN, HOMER1, PMAIP1, OSMR, HIPK2, MC1R, IRAK2, CXCL10, CFLAR, PDGFA, CXCL1, IL6R, IFI6, CD274, CHRNB2, RRAD, TNFAIP3, IL15RA, IL12A, MAP4K4, NBN, BID, HGS, BNIP3, SPHK1, EPHA2, DUSP10, CCL2, TNFRSF10A, RELA, CASP7, SQSTM1, TRIP10, AKAP12, CCL5, GP1BA, CXCL11, STAT1, KLF10, INPP1, TGFA, IFNB1, IL6, TICAM1, MX1, SCG2, TRAF4, SIAH2, TP53BP2, GTPBP2, EXT1, RIPK2, CREM, GNG4, IFITM1, GPRC5A, IL27RA, TRAF3, RIT1, RGS20, SRC, SERPINB9, IL1B, MRAS, DUSP4, CD40, BIRC2, NOTCH2, BIRC3, NRP1, NR3C1, LY6E, CXCL5, SKIL, IL8, PTGER2 |
| NETPATH TNF ALPHA PATHWAY UP | ELF3, EMP1, TRAF4, TP53BP2, OPTN, IFIT5, PMAIP1, GFPT2, NFKBIA, EGR1, PDLIM4, RIPK2, CXCL2, KLF7, RELB, SOD2, CXCL10, CFLAR, IFITM1, TNIP1, CXCL1, CSF1, TRAF3, SLC22A4, GBP2, IFITM3, EFNA1, TNFAIP2, TNFAIP8, SERPINB9, IL1B, WDR4, TNFAIP3, IL15RA, ICAM1, IRF1, BID, SMOX, CCL2, CASP7, DEXI, CCL5, SDC4, CXCL11, IFIT2, PTX3, IER3, BIRC3, WARS, KLF10, PPP1R15A, TRIM21, ADAM17, ETS2, SLCO3A1, IFNB1, IFI27, IL8, PLAUR, MX1 |
| NETPATH IL 2 PATHWAY UP | DDX21, PFKFB3, PMAIP1, IFI44, FOS, CREM, CFLAR, IFIT1, B4GALT5, MFHAS1, CSF1, DENND3, IFITM3, IL1B, SERPINE1, ICAM1, IRF1, MYC, UPP1, DUSP4, GBP1, RELA, CCND1, PIM1, IER3, STAT1, DUSP5, KLF6, LIF, WARS, TOP1, TRIM21, ETS2, IL8, PTGER2, SLC7A5, CHSY1, PLAUR, MX1 |
| NETPATH IL 1 PATHWAY UP | CCL2, RELA, FOSL1, NFKBIA, MT2A, CXCL2, SOD2, PTX3, CFB, BMP1, BIRC3, LIF, CXCL1, WARS, NP, TFPI, CXCL5, IL1B, SERPINE1, ICAM1, IL8, MYC |
| KEGG CYTOKINE CYTOKINE RECEPTOR INTERACTION | CCL2, CD40, OSMR, IL28RA, CCL5, CXCL2, CXCL11, CXCL10, PDGFB, PDGFA, CXCL1, CSF1, CXCL5, IFNB1, IL6, IL15RA, IL1B, MET, IL12A, IL8 |
| RESPONSE TO EXTERNAL STIMULUS | ELF3, IL1RAP, EREG, CCL2, PLAT, CD40, RELA, FOSL1, CCL5, RIPK2, IRAK2, FOS, CXCL2, CXCL11, ITGA2, CXCL10, PTX3, CXCL1, TFPI, CXCL5, SERPINE1, CHST2, IL8, PLAUR, SCG2 |
| CELL PROLIFERATION GO 0008283 | BAI1, EMP1, EREG, BCAT1, SPEG, ISG20, RUNX3, CXCL10, IFITM1, LAMP3, PDGFA, CXCL1, CSF1, CD274, IL1B, IL15RA, MYC, HGS, TCIRG1, SPHK1, TRIB1, EIF2AK2, CD276, FOSL1, NOTCH2, PIM1, ICOSLG, NRP1, ARHGEF2, LIF, WARS, KLF10, TGFA, CXCL5, IL8, SCG2 |
| POSITIVE REGULATION OF BIOLOGICAL PROCESS | EREG, TP53BP2, BCL2L11, PMAIP1, EGR1, HIPK2, RIPK2, TGM2, RUNX3, CXCL10, CFLAR, CDC42EP1, PDGFA, PML, CSF1, ELF4, TRAF3, HIVEP3, IL12A, BID, MYC, BNIP3, TCIRG1, SPHK1, CD40, BIRC2, CCND1, CD276, RELA, SQSTM1, FOSL1, NUFIP1, NOTCH2, NRP1, LIF, NRIP1, TGFA, IFNB1, CXCL5, IL8, TICAM1, SCG2, MX1 |
| KEGG COMPLEMENT AND COAGULATION CASCADES | BDKRB1, SERPINA1, PLAT, SERPINE1, F3, CD55, PLAUR, CFB |
| RESPONSE TO WOUNDING | ELF3, IL1RAP, EREG, PLAT, CD40, RELA, CCL5, RIPK2, IRAK2, FOS, CXCL2, CXCL11, ITGA2, CXCL10, PTX3, CXCL1, TFPI, SERPINE1, CHST2, IL8, SCG2 |
| NETPATH IL 5 PATHWAY UP | PLSCR1, DUSP5, PPIF, CCL2, TRAF3, RELA, EGR1, IL1B, ICAM1, IL8, PIM1, CD55, RELB, UPP1, IER3 |
| REACTOME GPCR LIGAND BINDING | BDKRB1, CXCL1, CCL2, CXCL5, CCL5, PTGER2, IL8, ADORA2B, FZD7, CXCL2, GNG4, CXCL10, CXCL11 |
| KEGG TOLL LIKE RECEPTOR SIGNALING PATHWAY | TRAF3, CD40, MAP3K8, RELA, IFNB1, NFKBIA, IL1B, IL12A, CCL5, IL8, IRF7, TICAM1, CXCL10, CXCL11 |
| POSITIVE REGULATION OF CELLULAR PROCESS | EREG, TP53BP2, BCL2L11, PMAIP1, EGR1, HIPK2, RIPK2, TGM2, RUNX3, CXCL10, CDC42EP1, CFLAR, PDGFA, CSF1, ELF4, TRAF3, HIVEP3, IL12A, MYC, BID, BNIP3, TCIRG1, SPHK1, CD40, BIRC2, CCND1, CD276, RELA, SQSTM1, FOSL1, NUFIP1, NOTCH2, NRP1, LIF, TGFA, CXCL5, IFNB1, TICAM1, SCG2, MX1 |
| KEGG JAK STAT SIGNALING PATHWAY | STAT1, LIF, IL6R, CCND1, OSMR, IL28RA, IFNB1, IL6, IL15RA, IL12A, OSM, PIM1, SPRY1, MYC |
| NETPATH EGFR1 PATHWAY UP | DDX21, EMP1, EREG, CCND1, PDLIM4, EGR1, AKAP12, SDC4, MT1M, TGM2, ITGA2, IER3, GPRC5A, LIF, NP, TGFA, CXCL5, TNFAIP3, MET, TFPI2, MYC, PLAUR |
| REACTOME CLASS A1 RHODOPSIN LIKE RECEPTORS | CXCL1, CCL2, CXCL5, CCL5, IL8, CXCL2, PTGER2, CXCL10, CXCL11 |
| DEFENSE RESPONSE | ELF3, TCIRG1, MX2, IL1RAP, EREG, CD40, RELA, FOSL1, VEZF1, CCL5, RIPK2, CXCL2, IRAK2, FOS, CXCL11, CXCL10, PTX3, TNIP1, CXCL1, KCNN4, CHST2, RSAD2, IL12A, IL8, MX1, SCG2, BNIP3 |
| NETPATH KIT RECEPTOR PATHWAY UP | DUSP4, CCL2, TAP1, CCND1, RELA, EGR1, CCL5, PIM1, FOS, MYC, IER3 |
| BEHAVIOR | CXCL1, FOSB, CCL2, FOSL1, CXCL5, CCL5, IL8, CXCL2, CXCL11, CXCL10, PLAUR, SCG2 |
| NETPATH B CELL RECEPTOR PATHWAY UP | EPHA2, EREG, CCL2, DDIT3, TP53BP2, BCL2L11, EIF2AK2, EGR1, CCL5, PIM1, FOS, CFLAR, IFITM1, BIRC3, ELF4, TNFAIP3, ICAM1, MYC |
| LOCOMOTORY BEHAVIOR | CXCL1, CCL2, FOSL1, CXCL5, CCL5, CXCL2, IL8, CXCL11, CXCL10, PLAUR, SCG2 |
| KEGG CHEMOKINE SIGNALING PATHWAY | STAT1, CXCL1, CCL2, RELA, CXCL5, NFKBIA, CCL5, IL8, CXCL2, CXCL11, GNG4, CXCL10 |
| KEGG CYTOSOLIC DNA SENSING PATHWAY | RELA, NFKBIA, IFNB1, IL6, IL1B, CCL5, IRF7, CXCL10, DDX58 |
| IMMUNE SYSTEM PROCESS | EREG, CCL2, CD276, IL28RA, NOTCH2, CCL5, ICOSLG, CSF1, IFI6, CD274, GBP2, IFITM3, SEMA4D, IL6, RSAD2, IFITM2, IL12A, IL8, SCG2 |
| REACTOME PEPTIDE LIGAND BINDING RECEPTORS | CXCL1, CCL2, CXCL5, CCL5, CXCL2, IL8, CXCL11, CXCL10 |
| EXTRACELLULAR REGION PART | SERPINA1, CXCL1, EREG, CCL2, TNFAIP2, IL1B, IL12A, CXCL2, IL8, SCG2 |
| CYTOKINE ACTIVITY | CXCL1, CSF1, CCL2, CXCL5, IL12A, CCL5, IL8, CXCL2, CXCL10, CXCL11, SCG2 |
| CHEMOKINE ACTIVITY | CXCL1, CCL2, CXCL5, CCL5, CXCL2, IL8, CXCL11, CXCL10 |
| REACTOME SIGNALING IN IMMUNE SYSTEM | DUSP4, TRIB3, CD40, MAP3K8, RELA, MAP3K14, SLC7A11, NFKBIA, RIPK2, FOS, HLA-C, CFB, IFITM1, ICOSLG, CD58, CD274, PVRL2, ICAM1, SLC7A5, IRF7, TICAM1, HLA-G |
| KEGG NOD LIKE RECEPTOR SIGNALING PATHWAY | CXCL1, CCL2, NFKBIA, IL1B, TNFAIP3, CCL5, IL8, RIPK2, CXCL2 |
| REGULATION OF CELL PROLIFERATION | TCIRG1, SPHK1, EREG, EIF2AK2, CD276, FOSL1, SPEG, NOTCH2, CXCL10, IFITM1, ICOSLG, ARHGEF2, NRP1, PDGFA, CSF1, CXCL1, KLF10, TGFA, CXCL5, IL1B, IL8, MYC, SCG2 |
| REACTOME CHEMOKINE RECEPTORS BIND CHEMOKINES | CXCL1, CCL2, CXCL5, CCL5, CXCL2, IL8, CXCL11, CXCL10 |
| CELL CELL SIGNALING | NRP1, PDGFA, LIF, EREG, KLF10, CXCL5, EFNA1, IL1B, CCL5, CXCL10, CXCL11 |
| EXTRACELLULAR REGION | SERPINA1, CXCL1, EREG, CCL2, TNFAIP2, IL1B, IL12A, DMD, IL8, CXCL2, SCG2, PTX3 |
| EXTRACELLULAR SPACE | SERPINA1, CXCL1, EREG, CCL2, TNFAIP2, IL1B, IL12A, CXCL2, IL8, SCG2 |
| RECEPTOR BINDING | CSF1, CXCL1, EREG, CCL2, TGFA, CXCL5, EFNA1, OASL, IL12A, CCL5, IL8, CXCL2, CXCL11, CXCL10, SCG2 |
| CHEMOKINE RECEPTOR BINDING | CXCL1, CCL2, CXCL5, CCL5, CXCL2, IL8, CXCL11, CXCL10 |
| NETPATH IL 3 PATHWAY UP | TGFA, IL1B, CSF1, CCL2, IL8, PIM1, MYC |
| G PROTEIN COUPLED RECEPTOR BINDING | CXCL1, CCL2, CXCL5, CCL5, CXCL2, IL8, CXCL11, CXCL10 |
| NCI DISSOLUTION OF FIBRIN CLOT | SERPINE1, PLAT, PLAUR |
| CHEMICAL HOMEOSTASIS | BDKRB1, CCL5, MT2A, CCL2, IFI6, CD55, MYC, BNIP3 |
| NCI REG GR PATHWAY | STAT1, EGR1, ICAM1, IRF1, IL8, FOS, RELA |
| CELLULAR CATION HOMEOSTASIS | BDKRB1, CCL5, MT2A, CCL2, CD55, MYC |
| NCI IL23PATHWAY | STAT1, NFKBIA, IL1B, CXCL1, CCL2, RELA |
| REACTOME G ALPHA I SIGNALLING EVENTS | BDKRB1, CXCL1, CXCL5, CXCL16, CCL5, CXCL2, IL8, GNG4, CXCL10, CXCL11 |
| ION HOMEOSTASIS | BDKRB1, CCL5, MT2A, CCL2, IFI6, CD55, MYC, BNIP3 |
| CELLULAR HOMEOSTASIS | BDKRB1, CCL5, MT2A, CCL2, DDIT3, IFI6, CD55, MYC, BNIP3 |
| NETPATH IL 4 PATHWAY DOWN | TNS1, CCL2, GBP2, NFKBIA, NINJ1, ICAM1, IRF1, CXCL2, IL8, MX1 |
| CATION HOMEOSTASIS | BDKRB1, CCL5, MT2A, CCL2, CD55, MYC |
| NETPATH B CELL RECEPTOR PATHWAY DOWN | TRAF4, DUSP5, ARHGEF2, PDGFA, CXCL1, GBP2, CASP7, EIF2AK2, MAP3K14, NFKBIA, ISG20, IL8, IRF1, IRF7, MYC |
| KEGG RIG I LIKE RECEPTOR SIGNALING PATHWAY | ISG15, IFIH1, TRAF3, DDX3Y, AZI2, RELA, DDX3X, IFNB1, TRIM25, NFKBIA, IL12A, IL8, IRF7, CXCL10, DDX58 |
| NCI IL27PATHWAY | STAT1, IL6, IL1B, IL27RA, IL12A |
| POSITIVE REGULATION OF CELL PROLIFERATION | ICOSLG, TCIRG1, NRP1, SPHK1, PDGFA, LIF, CSF1, EREG, CD276, TGFA, FOSL1, CXCL5, IL6, CXCL10, MYC, SCG2 |
| INFLAMMATORY RESPONSE | ELF3, CXCL1, RELA, CCL5, IL8, RIPK2, IRAK2, CXCL2, CXCL10, CXCL11, SCG2, PTX3 |
| KEGG GRAFT VERSUS HOST DISEASE | IL6, IL1B |
| NETPATH IL 7 PATHWAY UP | CXCL5, CXCL1, TRAF3, CCL5, IL8, CXCL2, MYC |
| BIOCARTA LAIR PATHWAY | ICAM1, IL8 |
| VIRAL REPRODUCTION | TNIP1, CCL2, IL8 |
| BIOCARTA LYM PATHWAY | ICAM1, IL8 |
| KEGG INTESTINAL IMMUNE NETWORK FOR IGA PRODUCTION | MAP3K14, ICOSLG, IL6, IL15RA, CD40 |
| RESPONSE TO OTHER ORGANISM | FOSL1, IFI44, RSAD2, IL12A, CCL5, ISG20, IRF7 |
| BIOCARTA FIBRINOLYSIS PATHWAY | SERPINE1, PLAT |
| KEGG CELL ADHESION MOLECULES CAMS | CD58, ICOSLG, CD40, CD274, CD276, PVRL2, ICAM1, SDC4, PTPRF, HLA-C, CLDN3, CLDN1, HLA-G |
| RESPONSE TO BIOTIC STIMULUS | FOSL1, RSAD2, IFI44, IL12A, CCL5, ISG20, IRF7 |
| NCI IL12 2PATHWAY | STAT1, IL1B, IL12A, FOS, RELB, RIPK2, RELA |
| KEGG APOPTOSIS | BIRC3, IL1RAP, BIRC2, TNFRSF10A, CASP7, RELA, MAP3K14, NFKBIA, IL1B, IRAK2, BID, CFLAR |
| RESPONSE TO VIRUS | FOSL1, IFI44, RSAD2, CCL5, ISG20, IRF7 |
| VIRAL GENOME REPLICATION | TNIP1, CCL2, IL8 |
| JAK STAT CASCADE | STAT1, IL12A, CCL2, HGS |
| PROTEIN KINASE CASCADE | DUSP4, CCL2, BIRC2, RELA, SQSTM1, HIPK2, RIPK2, IRAK2, CFLAR, STAT1, TGFA, TNFAIP3, IL12A, MAP4K4, TICAM1, HGS, SCG2 |
| KEGG LEISHMANIA INFECTION | STAT1, NFKBIA, IL1B, IL12A, FOS, RELA |
| NETPATH IL 6 PATHWAY UP | MAFF, STAT1, CXCL1, IRF1, PIM1, MX1 |
| REGULATION OF CELL ADHESION | IL12A, IL8, TGM2, ALOX12 |
| IMMUNE RESPONSE | EREG, CCL2, IFI6, CD274, GBP2, IFITM3, RSAD2, IFITM2, IL12A, CCL5 |
| MULTI ORGANISM PROCESS | TNIP1, FOSL1, MAFF, RSAD2, IFI44, ISG20, CCL5, IL12A, IRF7 |
| BIOCARTA GRANULOCYTES PATHWAY | ICAM1, IL8 |
| BIOCARTA STEM PATHWAY | CSF1, IL8 |
| NCI TNFPATHWAY | SQSTM1, STAT1, BIRC3, TNFAIP3, MAP4K4, BIRC2, ADAM17, RELA |
| BIOCARTA IL22BP PATHWAY | STAT1 |
| NCI NFAT TFPATHWAY | FOSL1, EGR1, GBP3, IL8, FOS |
| NCI CHEMOKINE RECEPTORS BIND CHEMOKINES | CCL5 |
| NETPATH HEDGEHOG PATHWAY UP | MYC, CCND1 |
| TYROSINE PHOSPHORYLATION OF STAT PROTEIN | STAT1, IL12A, SOCS1 |
| KEGG TYPE I DIABETES MELLITUS | IL1B, IL12A |
| NETPATH EGFR1 PATHWAY DOWN | IFITM1, IFIT1, IFI44L, CSF1, EREG, SQSTM1, TIMP3, NINJ1, IFI27, SPOCD1, CLDN1, CXCL10, IFIT2, IER3 |
| HUMORAL IMMUNE RESPONSE | CCL2 |
| SA MMP CYTOKINE CONNECTION | CSF1, IL1B |
| RECEPTOR SIGNALING PROTEIN ACTIVITY | IFITM1, SKIL, STAT1, IRAK2 |
| BIOCARTA CYTOKINE PATHWAY | IL12A, IL8 |
| KEGG AUTOIMMUNE THYROID DISEASE | HLA-C, CD40, HLA-G |
| NETPATH TNF ALPHA PATHWAY DOWN | IFIT5, CCND1, MAP3K14, NFKBIA, EGR1, EXT1, SDC4, CXCL2, IER3, KLF6, CXCL1, PPP1R15A, FOSB, KLF10, DDX3X, TNFAIP2, MAFF, TNFAIP3, IRF1, SYNJ2 |
| NETPATH WNT PATHWAY DOWN | IL1B, CXCL1, FZD7 |
| BIOCARTA CTL PATHWAY | ICAM1 |
| NCI CD40 PATHWAY | BIRC3, TRAF3, CD40, BIRC2, RELA, MAP3K14, NFKBIA, TNFAIP3, MYC |
| KEGG ALLOGRAFT REJECTION | IL12A, HLA-C, CD40, HLA-G |
| POSITIVE REGULATION OF CELL ADHESION | IL12A, TGM2, ALOX12 |
| REGULATION OF RESPONSE TO STIMULUS | EREG, IL12A, IL8, SCG2 |
| BIOCARTA INFLAM PATHWAY | PDGFA, CSF1, IL12A, IL8 |
| BIOCARTA NFKB PATHWAY | MAP3K14, NFKBIA, TNFAIP3, RELA |
| BIOCARTA NO2IL12 PATHWAY | IL12A |
| REGULATION OF DEFENSE RESPONSE | EREG, IL12A |
| INTERFERON GAMMA PRODUCTION | IL12A, CD276 |
| HEMOPOIETIC OR LYMPHOID ORGAN DEVELOPMENT |  |
| IMMUNE EFFECTOR PROCESS | RSAD2, IL12A |
| I KAPPAB KINASE NF KAPPAB CASCADE |  |
| LEUKOCYTE DIFFERENTIATION | CSF1 |
| REGULATION OF TYROSINE PHOSPHORYLATION OF STAT PROTEIN | IL12A, SOCS1 |
| POSITIVE REGULATION OF CYTOKINE BIOSYNTHETIC PROCESS |  |
| REGULATION OF I KAPPAB KINASE NF KAPPAB CASCADE |  |
| MYELOID CELL DIFFERENTIATION |  |
| POSITIVE REGULATION OF RESPONSE TO STIMULUS |  |
| PATTERN RECOGNITION RECEPTOR ACTIVITY |  |
| REGULATION OF PEPTIDYL TYROSINE PHOSPHORYLATION | IL12A |
| INNATE IMMUNE RESPONSE |  |
| CALCIUM MEDIATED SIGNALING |  |
| NETPATH TGFBETA RECEPTOR PATHWAY DOWN |  |
| REACTOME IMMUNOREGULATORY INTERACTIONS BETWEEN A LYMPHOID AND A NON LYMPHOID CELL |  |
| CELLULAR DEFENSE RESPONSE |  |
| ST TUMOR NECROSIS FACTOR PATHWAY |  |
| POSITIVE REGULATION OF PEPTIDYL TYROSINE PHOSPHORYLATION | IL12A |
| REACTOME VIRAL DSRNA TLR3 TRIF COMPLEX ACTIVATES RIP1 | NFKBIA, TICAM1, RELA |
| POSITIVE REGULATION OF SIGNAL TRANSDUCTION |  |
| CYTOKINE BIOSYNTHETIC PROCESS | EREG, CD276 |
| HORMONE SECRETION | LIF, OSM |
| REACTOME PHASE 1 FUNCTIONALIZATION OF COMPOUNDS |  |
| DEFENSE RESPONSE TO VIRUS | RSAD2 |
| CYTOKINE PRODUCTION |  |
| CYTOKINE METABOLIC PROCESS | EREG, CD276 |
| REGULATION OF JAK STAT CASCADE | IL12A, SOCS1, HGS |
| NEGATIVE REGULATION OF CELL PROLIFERATION |  |
| REACTOME STEROID HORMONES |  |
| POSITIVE REGULATION OF DEFENSE RESPONSE |  |
| INTRACELLULAR SIGNALING CASCADE |  |
| HORMONE ACTIVITY |  |
| IMMUNE SYSTEM DEVELOPMENT |  |
| REGULATION OF INTERFERON GAMMA BIOSYNTHETIC PROCESS | CD276 |
| HEMOPOIESIS |  |
| GENERATION OF A SIGNAL INVOLVED IN CELL CELL SIGNALING |  |
| BIOCARTA DEATH PATHWAY |  |
| POSITIVE REGULATION OF TRANSLATION | EREG, CD276 |
| REACTOME TOLL RECEPTOR CASCADES |  |
| REGULATION OF CYTOKINE BIOSYNTHETIC PROCESS | EREG, CD276 |
| REACTOME P75NTR SIGNALS VIA NFKB | SQSTM1, NFKBIA, RIPK2, RELA |
| BIOCARTA TOLL PATHWAY |  |

| Color legend | | | | | | | | | | | |
| --- | --- | --- | --- | --- | --- | --- | --- | --- | --- | --- | --- |
| q-value | 1 | 0.2 | 0.05 | 0.01 | 0.001 | 0.0001 |
| Color |  | |  |  |  | |

TABLE OF Q-VALUES

| candida albicans moddc135 | alternaria alternata beas2b | Gene Set |
| --- | --- | --- |
| 0.0151228625 | 0.15549001 | SIGNAL\_TRANSDUCTION |
| 2.4793292E-6 | 0.06082179 | NETPATH\_TNF\_ALPHA\_PATHWAY\_UP |
| 0.0 | 0.0 | NETPATH\_IL\_2\_PATHWAY\_UP |
| 0.0 | 0.0014360275 | NETPATH\_IL\_1\_PATHWAY\_UP |
| 0.0 | 4.8502272E-5 | KEGG\_CYTOKINE\_CYTOKINE\_RECEPTOR\_INTERACTION |
| 2.43421E-4 | 1.715488E-5 | RESPONSE\_TO\_EXTERNAL\_STIMULUS |
| 0.006118008 | 0.08443198 | CELL\_PROLIFERATION\_GO\_0008283 |
| 0.025013098 | 0.08685422 | POSITIVE\_REGULATION\_OF\_BIOLOGICAL\_PROCESS |
| 0.0020074716 | 0.0 | KEGG\_COMPLEMENT\_AND\_COAGULATION\_CASCADES |
| 2.1165008E-6 | 6.9193807E-6 | RESPONSE\_TO\_WOUNDING |
| 2.7117665E-6 | 0.0012581189 | NETPATH\_IL\_5\_PATHWAY\_UP |
| 0.0016598669 | 0.023852112 | REACTOME\_GPCR\_LIGAND\_BINDING |
| 2.892551E-6 | 2.0601391E-4 | KEGG\_TOLL\_LIKE\_RECEPTOR\_SIGNALING\_PATHWAY |
| 0.030061496 | 0.08361499 | POSITIVE\_REGULATION\_OF\_CELLULAR\_PROCESS |
| 2.9922942E-6 | 0.13215114 | KEGG\_JAK\_STAT\_SIGNALING\_PATHWAY |
| 0.0 | 0.0482491 | NETPATH\_EGFR1\_PATHWAY\_UP |
| 1.4066127E-4 | 7.181068E-4 | REACTOME\_CLASS\_A1\_RHODOPSIN\_LIKE\_RECEPTORS |
| 2.1694132E-6 | 0.0 | DEFENSE\_RESPONSE |
| 3.7335965E-5 | 0.026568137 | NETPATH\_KIT\_RECEPTOR\_PATHWAY\_UP |
| 2.2250392E-6 | 1.2214923E-5 | BEHAVIOR |
| 0.0030833506 | 0.11155586 | NETPATH\_B\_CELL\_RECEPTOR\_PATHWAY\_UP |
| 3.2139455E-6 | 0.0 | LOCOMOTORY\_BEHAVIOR |
| 6.846009E-5 | 0.029972985 | KEGG\_CHEMOKINE\_SIGNALING\_PATHWAY |
| 1.3989894E-4 | 4.208835E-4 | KEGG\_CYTOSOLIC\_DNA\_SENSING\_PATHWAY |
| 2.410459E-6 | 4.841327E-5 | IMMUNE\_SYSTEM\_PROCESS |
| 0.0 | 0.0 | REACTOME\_PEPTIDE\_LIGAND\_BINDING\_RECEPTORS |
| 4.004787E-5 | 0.18083072 | EXTRACELLULAR\_REGION\_PART |
| 0.0 | 0.0 | CYTOKINE\_ACTIVITY |
| 0.0 | 0.0 | CHEMOKINE\_ACTIVITY |
| 0.002677061 | 0.02860721 | REACTOME\_SIGNALING\_IN\_IMMUNE\_SYSTEM |
| 2.5522509E-6 | 0.0015941259 | KEGG\_NOD\_LIKE\_RECEPTOR\_SIGNALING\_PATHWAY |
| 0.00510492 | 0.014974086 | REGULATION\_OF\_CELL\_PROLIFERATION |
| 0.0 | 0.0 | REACTOME\_CHEMOKINE\_RECEPTORS\_BIND\_CHEMOKINES |
| 4.9217895E-4 | 0.068371326 | CELL\_CELL\_SIGNALING |
| 1.07919106E-4 | 0.17699227 | EXTRACELLULAR\_REGION |
| 0.0 | 0.0027369289 | EXTRACELLULAR\_SPACE |
| 0.0 | 0.00988802 | RECEPTOR\_BINDING |
| 0.0 | 0.0 | CHEMOKINE\_RECEPTOR\_BINDING |
| 0.0 | 0.0027465345 | NETPATH\_IL\_3\_PATHWAY\_UP |
| 0.0 | 9.6749085E-5 | G\_PROTEIN\_COUPLED\_RECEPTOR\_BINDING |
| 0.034454864 | 0.059493493 | NCI\_DISSOLUTION\_OF\_FIBRIN\_CLOT |
| 0.005975001 | 0.08715195 | CHEMICAL\_HOMEOSTASIS |
| 0.005379269 | 0.058216974 | NCI\_REG\_GR\_PATHWAY |
| 0.003281799 | 0.18564403 | CELLULAR\_CATION\_HOMEOSTASIS |
| 0.0 | 6.501296E-4 | NCI\_IL23PATHWAY |
| 0.009618234 | 4.58652E-5 | REACTOME\_G\_ALPHA\_I\_SIGNALLING\_EVENTS |
| 0.0042384043 | 0.057703048 | ION\_HOMEOSTASIS |
| 0.018229602 | 0.13101315 | CELLULAR\_HOMEOSTASIS |
| 0.0 | 0.0 | NETPATH\_IL\_4\_PATHWAY\_DOWN |
| 0.0040113498 | 0.17835741 | CATION\_HOMEOSTASIS |
| 0.015502429 | 0.0489315 | NETPATH\_B\_CELL\_RECEPTOR\_PATHWAY\_DOWN |
| 1.6542599E-5 | 0.0 | KEGG\_RIG\_I\_LIKE\_RECEPTOR\_SIGNALING\_PATHWAY |
| 9.398161E-5 | 0.06640709 | NCI\_IL27PATHWAY |
| 4.1946248E-4 | 0.02215129 | POSITIVE\_REGULATION\_OF\_CELL\_PROLIFERATION |
| 4.0434443E-6 | 0.0 | INFLAMMATORY\_RESPONSE |
| 0.0 | 0.020532541 | KEGG\_GRAFT\_VERSUS\_HOST\_DISEASE |
| 0.0 | 0.08906518 | NETPATH\_IL\_7\_PATHWAY\_UP |
| 0.0023013013 | 0.022626787 | BIOCARTA\_LAIR\_PATHWAY |
| 0.019178493 | 0.18524252 | VIRAL\_REPRODUCTION |
| 0.033600047 | 0.02800103 | BIOCARTA\_LYM\_PATHWAY |
| 1.4610786E-4 | 0.0061623864 | KEGG\_INTESTINAL\_IMMUNE\_NETWORK\_FOR\_IGA\_PRODUCTION |
| 6.656398E-5 | 0.0 | RESPONSE\_TO\_OTHER\_ORGANISM |
| 0.009271151 | 0.18600726 | BIOCARTA\_FIBRINOLYSIS\_PATHWAY |
| 0.0032991811 | 0.18163502 | KEGG\_CELL\_ADHESION\_MOLECULES\_CAMS |
| 3.6631514E-5 | 0.005413289 | RESPONSE\_TO\_BIOTIC\_STIMULUS |
| 0.0 | 0.0064716395 | NCI\_IL12\_2PATHWAY |
| 0.019864451 | 0.14210635 | KEGG\_APOPTOSIS |
| 2.2835927E-6 | 7.040857E-5 | RESPONSE\_TO\_VIRUS |
| 0.001417204 | 0.12211427 | VIRAL\_GENOME\_REPLICATION |
| 0.004624408 | 0.07725537 | JAK\_STAT\_CASCADE |
| 0.016131835 | 0.057919584 | PROTEIN\_KINASE\_CASCADE |
| 3.0991619E-6 | 0.031793345 | KEGG\_LEISHMANIA\_INFECTION |
| 0.0 | 0.004494796 | NETPATH\_IL\_6\_PATHWAY\_UP |
| 0.033861402 | 0.006116781 | REGULATION\_OF\_CELL\_ADHESION |
| 0.0 | 4.6192636E-5 | IMMUNE\_RESPONSE |
| 0.003974432 | 6.4580886E-6 | MULTI\_ORGANISM\_PROCESS |
| 0.029836837 | 0.006535667 | BIOCARTA\_GRANULOCYTES\_PATHWAY |
| 3.7597125E-5 | 0.048412904 | BIOCARTA\_STEM\_PATHWAY |
| 0.013837767 | 0.15428899 | NCI\_TNFPATHWAY |
| 0.04441359 | 0.024500562 | BIOCARTA\_IL22BP\_PATHWAY |
| 5.8961764E-4 | 0.0028159413 | NCI\_NFAT\_TFPATHWAY |
| 3.2052063E-4 | 0.09035489 | NCI\_CHEMOKINE\_RECEPTORS\_BIND\_CHEMOKINES |
| 0.044520505 | 0.1578012 | NETPATH\_HEDGEHOG\_PATHWAY\_UP |
| 0.011830762 | 0.15143469 | TYROSINE\_PHOSPHORYLATION\_OF\_STAT\_PROTEIN |
| 0.0 | 0.09067089 | KEGG\_TYPE\_I\_DIABETES\_MELLITUS |
| 0.0025017355 | 0.024863789 | NETPATH\_EGFR1\_PATHWAY\_DOWN |
| 4.1889373E-4 | 0.033842232 | HUMORAL\_IMMUNE\_RESPONSE |
| 0.0035063634 | 0.17880398 | SA\_MMP\_CYTOKINE\_CONNECTION |
| 0.039638337 | 0.11427313 | RECEPTOR\_SIGNALING\_PROTEIN\_ACTIVITY |
| 0.0 | 0.06687076 | BIOCARTA\_CYTOKINE\_PATHWAY |
| 6.802776E-4 | 0.0031133108 | KEGG\_AUTOIMMUNE\_THYROID\_DISEASE |
| 2.3453117E-6 | 0.09455213 | NETPATH\_TNF\_ALPHA\_PATHWAY\_DOWN |
| 0.002521776 | 0.16622809 | NETPATH\_WNT\_PATHWAY\_DOWN |
| 0.02464002 | 0.14031996 | BIOCARTA\_CTL\_PATHWAY |
| 1.14572584E-4 | 0.039924555 | NCI\_CD40\_PATHWAY |
| 0.0 | 0.0072650104 | KEGG\_ALLOGRAFT\_REJECTION |
| 0.029370287 | 0.034463257 | POSITIVE\_REGULATION\_OF\_CELL\_ADHESION |
| 0.033170246 | 0.039942924 | REGULATION\_OF\_RESPONSE\_TO\_STIMULUS |
| 0.0 | 0.024269812 | BIOCARTA\_INFLAM\_PATHWAY |
| 0.01410618 | 0.10728212 | BIOCARTA\_NFKB\_PATHWAY |
| 0.0077500087 | 0.015852578 | BIOCARTA\_NO2IL12\_PATHWAY |
| 0.03576746 | 0.01955003 | REGULATION\_OF\_DEFENSE\_RESPONSE |
| 0.0012447159 | 0.098003834 | INTERFERON\_GAMMA\_PRODUCTION |
| 0.17016491 | 0.09094638 | HEMOPOIETIC\_OR\_LYMPHOID\_ORGAN\_DEVELOPMENT |
| 4.1547208E-4 | 0.089186 | IMMUNE\_EFFECTOR\_PROCESS |
| 0.12882927 | 0.0026909143 | I\_KAPPAB\_KINASE\_NF\_KAPPAB\_CASCADE |
| 0.0046358434 | 0.18000008 | LEUKOCYTE\_DIFFERENTIATION |
| 0.031802375 | 0.17055395 | REGULATION\_OF\_TYROSINE\_PHOSPHORYLATION\_OF\_STAT\_PROTEIN |
| 0.073085286 | 0.0104400525 | POSITIVE\_REGULATION\_OF\_CYTOKINE\_BIOSYNTHETIC\_PROCESS |
| 0.17607056 | 0.010700843 | REGULATION\_OF\_I\_KAPPAB\_KINASE\_NF\_KAPPAB\_CASCADE |
| 0.15994087 | 0.07277227 | MYELOID\_CELL\_DIFFERENTIATION |
| 0.06268975 | 0.06788228 | POSITIVE\_REGULATION\_OF\_RESPONSE\_TO\_STIMULUS |
| 0.124737374 | 0.026693936 | PATTERN\_RECOGNITION\_RECEPTOR\_ACTIVITY |
| 0.008728356 | 0.06715995 | REGULATION\_OF\_PEPTIDYL\_TYROSINE\_PHOSPHORYLATION |
| 0.07530166 | 0.0063103526 | INNATE\_IMMUNE\_RESPONSE |
| 0.13157527 | 0.056881335 | CALCIUM\_MEDIATED\_SIGNALING |
| 0.052799206 | 0.18161221 | NETPATH\_TGFBETA\_RECEPTOR\_PATHWAY\_DOWN |
| 0.08234739 | 0.0027055622 | REACTOME\_IMMUNOREGULATORY\_INTERACTIONS\_BETWEEN\_A\_LYMPHOID\_AND\_A\_NON\_LYMPHOID\_CELL |
| 0.109449334 | 0.15413347 | CELLULAR\_DEFENSE\_RESPONSE |
| 0.055568002 | 0.11110564 | ST\_TUMOR\_NECROSIS\_FACTOR\_PATHWAY |
| 0.011540983 | 0.15405402 | POSITIVE\_REGULATION\_OF\_PEPTIDYL\_TYROSINE\_PHOSPHORYLATION |
| 0.0073752627 | 0.1594682 | REACTOME\_VIRAL\_DSRNA\_TLR3\_TRIF\_COMPLEX\_ACTIVATES\_RIP1 |
| 0.082406156 | 0.023551568 | POSITIVE\_REGULATION\_OF\_SIGNAL\_TRANSDUCTION |
| 0.020800248 | 0.010602886 | CYTOKINE\_BIOSYNTHETIC\_PROCESS |
| 0.013864454 | 0.14520921 | HORMONE\_SECRETION |
| 0.11710141 | 0.19245763 | REACTOME\_PHASE\_1\_FUNCTIONALIZATION\_OF\_COMPOUNDS |
| 0.023920348 | 0.18122093 | DEFENSE\_RESPONSE\_TO\_VIRUS |
| 0.059200067 | 0.09149431 | CYTOKINE\_PRODUCTION |
| 0.020787546 | 0.0048122746 | CYTOKINE\_METABOLIC\_PROCESS |
| 0.031942807 | 0.09628014 | REGULATION\_OF\_JAK\_STAT\_CASCADE |
| 0.06107344 | 0.053820435 | NEGATIVE\_REGULATION\_OF\_CELL\_PROLIFERATION |
| 0.15807852 | 0.048446074 | REACTOME\_STEROID\_HORMONES |
| 0.17313954 | 0.032808315 | POSITIVE\_REGULATION\_OF\_DEFENSE\_RESPONSE |
| 0.09532635 | 0.10679764 | INTRACELLULAR\_SIGNALING\_CASCADE |
| 0.011306912 | 0.032127097 | HORMONE\_ACTIVITY |
| 0.18702486 | 0.090045236 | IMMUNE\_SYSTEM\_DEVELOPMENT |
| 0.0028675264 | 0.06865664 | REGULATION\_OF\_INTERFERON\_GAMMA\_BIOSYNTHETIC\_PROCESS |
| 0.17024256 | 0.13300456 | HEMOPOIESIS |
| 0.095168166 | 0.06720909 | GENERATION\_OF\_A\_SIGNAL\_INVOLVED\_IN\_CELL\_CELL\_SIGNALING |
| 0.18593393 | 0.036749456 | BIOCARTA\_DEATH\_PATHWAY |
| 0.00871612 | 0.02836 | POSITIVE\_REGULATION\_OF\_TRANSLATION |
| 0.17623867 | 0.08390498 | REACTOME\_TOLL\_RECEPTOR\_CASCADES |
| 0.0108003775 | 0.0027807166 | REGULATION\_OF\_CYTOKINE\_BIOSYNTHETIC\_PROCESS |
| 0.039409634 | 0.1787799 | REACTOME\_P75NTR\_SIGNALS\_VIA\_NFKB |
| 0.17945796 | 0.023981113 | BIOCARTA\_TOLL\_PATHWAY |
