# Supplementary material for: Computational approaches for discovery of common immunomodulators in fungal infections: towards broad-spectrum immunotherapeutic interventions
Source: BMC Microbiol. 2013 Oct 7;13:224. doi: 10.1186/1471-2180-13-224 (PMC3853472; doi:10.1186/1471-2180-13-224)
Supplement: Additional file 1 — Details of up- and down- regulated biclusters. [file 1471-2180-13-224-S1.zip › 2013-kidane-bmc/details-of-biclusters/upreg-biclust-50.html]

**BICLUSTER\_ID** : UPREG-50  
**PATHOGENS** /2/ : c. albicans,a. fumigatus  
**KNOWN DRUG TARGETS** /17/ : HEXB, CTSD, JUN, GLA, CDK4, PLAU, SLC7A11, CTNS, PIM1, NEU1, PIK3R1, CTSB, SLC25A13, SMPD1, IL1B, IL8, TNF  

| Gene Set | Leading Edge Genes |
| --- | --- |
| KEGG LYSOSOME | ATP6V1H, HEXB, NPC2, CTSD, GGA1, GLA, CD63, NPC1, CTNS, CLTA, LIPA, SGSH, ATP6V0C, NEU1, M6PR, LAMP1, CLTC, ATP6V0D1, CD68, ATP6V0B, MCOLN1, ATP6AP1, CTSB, SMPD1, HEXA, CLN5 |
| NCI NFAT TFPATHWAY | FOSL1, SLC3A2, JUN, IL8, TNF, EGR2, CDK4 |
| NETPATH IL 3 PATHWAY UP | CSF1, CD69, CCR1, USP36, SOCS3, IL1B, SOCS1, IL8, OSM, PIM1, TNF, CCL4 |
| CYTOKINE ACTIVITY | CSF1, PIK3R1, CXCL3, IL1RN, IL8, CXCL2, OSM, CSF2, TNF, CCL4 |
| AMINO ACID TRANSMEMBRANE TRANSPORTER ACTIVITY | CTNS, SLC25A13, SLC7A11 |
| BIOCARTA PLATELETAPP PATHWAY | PLAU |
| HEMATOPOIETIN INTERFERON CLASSD200 DOMAIN CYTOKINE RECEPTOR BINDING | OSM, CSF2 |
| KEGG VIBRIO CHOLERAE INFECTION | ATP6V1E1, ATP6V1H, ATP6V0D1, ATP6V1D, ATP6AP1, ATP6V1C1, ATP6V1F, ATP6V1G1, ATP6V0C |

| Color legend | | | | | | | | | | | |
| --- | --- | --- | --- | --- | --- | --- | --- | --- | --- | --- | --- |
| q-value | 1 | 0.2 | 0.05 | 0.01 | 0.001 | 0.0001 |
| Color |  | |  |  |  | |

TABLE OF Q-VALUES

| aspergillus fumigatus monocytes | candida albicans neutrophils | Gene Set |
| --- | --- | --- |
| 0.014264559 | 0.17728922 | KEGG\_LYSOSOME |
| 0.19765195 | 0.035003204 | NCI\_NFAT\_TFPATHWAY |
| 0.009785512 | 0.10933371 | NETPATH\_IL\_3\_PATHWAY\_UP |
| 0.010335351 | 7.5262925E-4 | CYTOKINE\_ACTIVITY |
| 0.092043035 | 0.13208017 | AMINO\_ACID\_TRANSMEMBRANE\_TRANSPORTER\_ACTIVITY |
| 0.14451481 | 0.1780624 | BIOCARTA\_PLATELETAPP\_PATHWAY |
| 0.04804973 | 0.037406106 | HEMATOPOIETIN\_INTERFERON\_CLASSD200\_DOMAIN\_CYTOKINE\_RECEPTOR\_BINDING |
| 0.008837998 | 0.08450937 | KEGG\_VIBRIO\_CHOLERAE\_INFECTION |
