# Supplementary material for: Computational approaches for discovery of common immunomodulators in fungal infections: towards broad-spectrum immunotherapeutic interventions
Source: BMC Microbiol. 2013 Oct 7;13:224. doi: 10.1186/1471-2180-13-224 (PMC3853472; doi:10.1186/1471-2180-13-224)
Supplement: Additional file 1 — Details of up- and down- regulated biclusters. [file 1471-2180-13-224-S1.zip › 2013-kidane-bmc/details-of-biclusters/upreg-biclust-6.html]

**BICLUSTER\_ID** : UPREG-6  
**PATHOGENS** /2/ : c. albicans,s. chartarum  
**KNOWN DRUG TARGETS** /52/ : ACPP, MMP12, SLC7A11, RBP4, IFNG, CD86, CXCL10, CFB, PTPN1, NFKB1, PTGER4, PPIF, GPR109A, CCL8, SLC22A4, PPARG, IL1B, F3, CXCR4, ABCG1, CCR5, CCL20, CTPS, LDLR, CCL2, CD40, TNFRSF1B, SERPINB2, CD80, PTGIR, VCAM1, IL3RA, AMPD3, CCL5, PIM1, ADORA2B, CD55, PRKCQ, CTLA4, ANXA1, IL12B, MMP9, TAP1, NP, IL2RG, KCNN4, ANPEP, F5, IL6, PTGER2, TLR2, PLAUR  

| Gene Set | Leading Edge Genes |
| --- | --- |
| KEGG CYTOKINE CYTOKINE RECEPTOR INTERACTION | CCL22, IL7R, CCL24, OSMR, INHBA, CXCL2, TNFRSF18, CXCL10, CCR7, CXCL1, CCL8, IL1A, TNFSF9, IL10RA, IL1B, CCL3, TNFRSF9, CCR5, CCL20, CCL1, CCL2, CD40, TNFRSF1B, TNFRSF4, CXCR3, IL3RA, CCL7, TNFRSF11B, CCL5, CXCL11, CSF2, CCL4, TNFSF14, IL12B, CXCL5, IL6 |
| REACTOME GPCR LIGAND BINDING | GPR68, NMB, CCL22, CCR5, CCL20, CXCR6, CCL2, PTGIR, CXCR3, CCL7, CCL5, ADORA2B, CXCL2, CXCL10, CCL4, PTGER4, CCR7, CXCL1, P2RY1, GPR109A, CXCL5, CCL3, PTGER2, CXCR4 |
| NETPATH IL 1 PATHWAY UP | ACPP, CCL20, MMP12, CCL2, SERPINB2, CCL7, NFKBIA, AMPD3, PIM2, TNFRSF11B, MT2A, INHBA, CXCL2, CSF2, SOD2, CCL4, CFB, BIRC3, BMP1, CXCL1, CCL8, NP, IL1A, TNFAIP6, CKS2, CXCL5, IL6, IL1B, MYC |
| RESPONSE TO EXTERNAL STIMULUS | GPR68, CCL22, ELF3, CCR5, CCL20, PLA2G7, CXCL14, CCL2, CCL1, CD40, CCL24, CXCR3, CCL7, CCL5, INHBA, FOS, CXCL2, CXCL11, CXCL10, CCL4, ANXA1, CCR7, CXCL1, CCL8, PPARG, IL1A, TNFAIP6, CXCL5, F5, CCL3, PLAUR |
| RESPONSE TO CHEMICAL STIMULUS | ABCG1, CCL22, CCR5, CCL20, CTPS, CCL2, CCL1, CCL24, HERPUD1, CXCR3, PMAIP1, CCL7, CCL5, CXCL2, CLEC7A, CXCL11, SOD2, CXCL10, CCR7, CXCL1, CCL8, PPARG, AQP9, CXCL5, CCL3, PLAUR |
| NETPATH IL 2 PATHWAY UP | PTPN22, STAT5A, IRF4, PMAIP1, IFI44, FOS, CREM, B4GALT5, GADD45G, LCP2, RGS1, IL1B, CCL3, MYC, SOCS2, SACS, CCR5, CTPS, STAT4, LDLR, TNFRSF1B, TNFRSF4, SOCS3, PIM1, CYP7B1, SLAMF1, CSF2, CCL4, PPRC1, EZH2, KLF6, IL2RG, IL6, ID2, PTGER2, PLAUR, MX1 |
| IMMUNE SYSTEM PROCESS | CCL22, CST7, IL7R, CCL24, INHBA, LAT, CLEC7A, CD86, EBI3, IRF8, PTGER4, CD274, PDCD1, AQP9, SEMA4D, LCP2, RGS1, GEM, CD83, CCR5, CCL20, CCL2, TNFRSF4, CCL5, CCL4, ICOSLG, IL12B, MMP9, CTSE, MALT1, SEMA7A, TM7SF4, IL6 |
| REACTOME SIGNALING IN IMMUNE SYSTEM | OLR1, NFKB2, CD40, MAP3K8, CD80, IRAK3, VCAM1, SLC7A11, NFKBIA, LAT, FOS, CD86, RPS6KA3, CFB, PRKCQ, CTLA4, IFITM1, ICOSLG, CD48, C1R, CD84, ITGB7, CD274, MALT1, PDCD1, LCP2, TLR2, TICAM1 |
| KEGG TOLL LIKE RECEPTOR SIGNALING PATHWAY | CD40, MAP3K8, CD80, NFKBIA, CCL5, CD86, CXCL10, CXCL11, CCL4, IL12B, TRAF3, PIK3R5, IL6, CCL3, IL1B, TICAM1, TLR2 |
| REACTOME CLASS A1 RHODOPSIN LIKE RECEPTORS | GPR68, NMB, CCL22, CCR5, CCL20, CCL2, CXCR3, CCL7, CCL5, CXCL2, CXCL10, CCL4, PTGER4, CCR7, CXCL1, P2RY1, GPR109A, CXCL5, CCL3, PTGER2 |
| NETPATH T CELL RECEPTOR PATHWAY UP | CCR5, IL1RAP, DDIT3, CH25H, NFKBIA, EGR1, PIM2, CCL5, IFNG, CD86, CSF2, PSTPIP1, NFKB1, IL27RA, NP, TAP1, IL1A, TAF9, SLC5A3, IQGAP2, TNFAIP8, SEMA4D, IL6, RHOC, TFPI2, MYC |
| DEFENSE RESPONSE | CD83, ELF3, GPR68, TCIRG1, CCL22, CCR5, CCL20, PLA2G7, CCL24, NCF1, CCL5, INHBA, CXCL2, FOS, CXCL10, CCL4, CD48, ANXA1, IL12B, CCR7, TNIP1, CXCL1, CD84, IL1A, TNFAIP6, KCNN4, CCL3, MX1 |
| NETPATH IL 4 PATHWAY UP | CCL22, ETV3, CCL2, ELL2, CH25H, SOCS3, VCAM1, IL1RN, INHBA, FOS, CD86, AIM1, CREM, RGS2, SLC22A4, RASGRP1, IL6, IL10RA, EBI2, F3, PTGER2, MYC |
| EXTRACELLULAR SPACE | CCL22, CCL20, CCL2, CCL1, CCL7, IL1RN, INHBA, RBP4, CXCL2, EBI3, CCL4, IL12B, MMP9, CXCL1, IL1A, TNFAIP2, IL6, IL1B |
| BEHAVIOR | CCL22, CCR5, CCR7, CCL20, CXCL1, CCL8, CCL1, CCL2, CCL24, CXCR3, CXCL5, CCL7, CCL3, CCL5, CXCL2, CXCL10, PLAUR |
| RESPONSE TO WOUNDING | GPR68, CCL22, ELF3, CCR5, CCL20, PLA2G7, CD40, CCL24, CCL5, FOS, CXCL2, CXCL11, CXCL10, CCL4, ANXA1, CCR7, CXCL1, IL1A, TNFAIP6, F5, CCL3 |
| LOCOMOTORY BEHAVIOR | CCL22, CCR5, CCL20, CCL1, CCL2, CCL24, CXCR3, CCL7, CCL5, CXCL2, CXCL10, CXCL11, CCR7, CXCL1, CCL8, CXCL5, CCL3, PLAUR |
| IMMUNE RESPONSE | CD83, CCL22, CCL20, CST7, IL7R, CCL2, CCL24, TNFRSF4, CCL5, LAT, CD86, EBI3, CCL4, IRF8, PTGER4, IL12B, MALT1, SEMA7A, CD274, PDCD1, AQP9, LCP2, IL6, RGS1, GEM |
| CYTOKINE ACTIVITY | CCL22, CCL20, CCL2, CCL1, CCL24, CCL7, IL1RN, TNFRSF11B, CCL5, INHBA, CXCL2, CXCL11, CSF2, CXCL10, CCL4, IL12B, CXCL1, CCL8, CXCL5, CCL3 |
| REACTOME G ALPHA I SIGNALLING EVENTS | CCR5, CCL20, CCR7, CXCL1, CXCR6, GPR109A, CXCR3, CXCL5, CXCL16, CCL5, CXCL2, CXCL10, CXCR4, CXCL13 |
| REACTOME HEMOSTASIS | PTPN1, OLR1, CD48, P2RY1, PLEK, CD84, SERPINB2, SLC7A11, PIK3R5, LCP2, F5, F3, PLAUR |
| NETPATH IL 5 PATHWAY UP | NFKBIB, NFKB2, CCL2, GADD45A, IL3RA, EGR1, PIM1, CD55, RELB, REL, PPIF, TRAF3, IL1A, EGR3, LCP2, IDI1, IL1B, CCL3 |
| KEGG CHEMOKINE SIGNALING PATHWAY | CCL22, CCR5, CCL20, CXCL14, CXCR6, CCL2, CCL1, CCL24, CXCR3, NCF1, CCL7, NFKBIA, CCL5, CXCL2, CXCL11, CXCL10, CCL4, CCR7, CXCL1, CCL8, FGR, TIAM1, CXCL5, PIK3R5, CCL3 |
| INFLAMMATORY RESPONSE | GPR68, CCL22, ELF3, CCR5, CCL20, PLA2G7, CD40, CCL24, CCL5, FOS, CXCL2, CXCL11, CXCL10, CCL4, ANXA1, CCR7, CXCL1, IL1A, TNFAIP6, CCL3 |
| REACTOME PEPTIDE LIGAND BINDING RECEPTORS | CCL22, NMB, CCR5, CCR7, CCL20, CXCL1, CCL2, CXCR3, CXCL5, CCL7, CCL3, CCL5, CXCL2, CXCL10, CCL4 |
| CHEMOKINE ACTIVITY | CCL22, CCL20, CXCL1, CCL8, CCL1, CCL2, CCL24, CXCL5, CCL7, CCL3, CCL5, CXCL2, CXCL10, CXCL11, CCL4 |
| KEGG NOD LIKE RECEPTOR SIGNALING PATHWAY | CXCL1, CCL8, CCL2, NFKBIA, CCL7, IL6, TNFAIP3, IL1B, CCL5, CXCL2 |
| REACTOME CHEMOKINE RECEPTORS BIND CHEMOKINES | CCL22, CCR5, CCR7, CCL20, CXCL1, CCL2, CXCR3, CXCL5, CCL7, CCL3, CCL5, CXCL2, CXCL10, CCL4 |
| NCI NFAT TFPATHWAY | CTLA4, PRKCQ, PTPN1, PPARG, IRF4, EGR3, EGR1, IFNG, FOS, CSF2 |
| KEGG JAK STAT SIGNALING PATHWAY | IL12B, STAT4, IL7R, STAT5A, IL2RG, IL3RA, SOCS3, OSMR, PIK3R5, IL6, IL10RA, PIM1, CSF2, SOCS2, MYC |
| HOMEOSTATIC PROCESS | ABCG1, CCR5, CCR7, CCL2, CCL1, FXN, CXCR3, CCL7, IL6, CCL3, MT2A, CCL5, INHBA, MYC |
| CHEMOKINE RECEPTOR BINDING | CCL22, CCL20, CXCL1, CCL8, CCL1, CCL2, CCL24, CXCL5, CCL7, CCL3, CCL5, CXCL2, CXCL10, CXCL11, CCL4 |
| G PROTEIN COUPLED RECEPTOR BINDING | CCL22, CCL20, CXCL1, CCL8, CCL1, CCL2, CCL24, CXCL5, CCL7, CCL3, CCL5, CXCL2, CXCL10, CXCL11, CCL4 |
| KEGG GRAFT VERSUS HOST DISEASE | IL6, IL1B, CD86, CD80, IL1A |
| NETPATH IL 7 PATHWAY UP | CCL22, CXCL1, CCL8, TRAF3, STAT5A, CD80, CXCL5, CCL3, CCL5, CXCL2, CD86, MYC, CCL4 |
| KEGG COMPLEMENT AND COAGULATION CASCADES | F5, C1R, F3, CFB, PLAUR |
| CHEMICAL HOMEOSTASIS | ABCG1, CCR5, CCR7, CCL1, CCL2, FXN, CXCR3, CCL7, CCL3, CCL5, MT2A, MYC |
| KEGG INTESTINAL IMMUNE NETWORK FOR IGA PRODUCTION | ICOSLG, IL6, CD86, CD40, CD80 |
| CELLULAR CATION HOMEOSTASIS | CCR5, CCR7, CCL1, CCL2, FXN, CXCR3, CCL7, CCL3, MT2A, CCL5, CXCR4, MYC |
| NCI IL12 2PATHWAY | CCR5, IL12B, NFKB2, STAT4, STAT5A, GADD45G, IL2RG, IL1B, CCL3, RELB, FOS, CCL4 |
| CATION HOMEOSTASIS | CCR5, CCR7, CCL1, CCL2, FXN, CXCR3, CCL7, CCL3, MT2A, CCL5, CXCR4, MYC |
| NETPATH IL 3 PATHWAY UP | CCL2, SOCS3, MATK, CCL7, CCL3, IL1B, PIM1, CD86, MYC, SOCS2 |
| NETPATH IL 6 PATHWAY UP | MUC5AC, MMP9, CXCL1, LDLR, MAP3K8, SLC39A14, PPARG, SOCS3, MAFF, TNFRSF11B, PIM2, ID2, FOS, PIM1, SOCS2, MX1 |
| KEGG HEMATOPOIETIC CELL LINEAGE | IL3RA, ANPEP, IL6, IL1B, IL7R, IL1A, CSF2 |
| NCI CD8TCRPATHWAY | PRKCQ, MAP3K8, MALT1, CD80, LCP2, RASSF5, CD86, LAT |
| RESPONSE TO BIOTIC STIMULUS | CCL22, IL12B, CCL8, FGR, HERPUD1, IFI44, CCL5, CCL4 |
| ION HOMEOSTASIS | CCR5, CCR7, CCL1, CCL2, FXN, CXCR3, CCL7, CCL3, MT2A, CCL5, MYC |
| CELLULAR HOMEOSTASIS | CCR5, CCR7, CCL1, CCL2, FXN, CXCR3, CCL7, CCL3, MT2A, CCL5, MYC |
| CYTOKINE BINDING | IL7R, TNFRSF1B, TNFRSF4, IL2RG, IL3RA, IL10RA, TNFRSF18, EBI3 |
| KEGG PRION DISEASES | IL6, IL1B, CCL5, IL1A |
| BIOCARTA DC PATHWAY | IL12B, CD40, CSF2, TLR2 |
| KEGG LEISHMANIA INFECTION | IL12B, IL1A, NCF1, NFKBIA, IL1B, FOS, MARCKSL1, TLR2 |
| NETPATH IL 9 PATHWAY UP | MUC5AC, CCL2, SOCS3, CCL7, CCL3, PIM1, SOCS2, MYC |
| BIOCARTA NO2IL12 PATHWAY | CCR5, IL12B, STAT4, CXCR3 |
| INTERLEUKIN BINDING | IL3RA, IL2RG, IL10RA, IL7R, EBI3 |
| BIOCARTA NKT PATHWAY | CCR5, CCR7, IL12B, CCL3, CSF2, CCL4, CXCR3 |
| RESPONSE TO OTHER ORGANISM | CCL22, IFI44, CCL5, CCL8, CCL4 |
| NCI CD8TCRDOWNSTREAMPATHWAY | IL2RG, PRKCQ, TNFRSF9, STAT4, FOS, TNFRSF18, TNFRSF4 |
| KEGG T CELL RECEPTOR SIGNALING PATHWAY | CTLA4, PRKCQ, TEC, MALT1, RASGRP1, PDCD1, LCP2, PIK3R5, NFKBIA, PPP3CC, LAT, FOS, CSF2 |
| CYTOKINE BIOSYNTHETIC PROCESS | IL6, IL12B, INHBA, EBI3, IRF4 |
| REACTOME FORMATION OF FIBRIN CLOT CLOTTING CASCADE | F5, F3 |
| HUMORAL IMMUNE RESPONSE | CD83, IL6, CCL2, EBI3, PDCD1 |
| BIOCARTA IL12 PATHWAY | CCR5, IL12B, STAT4, CXCR3 |
| RESPONSE TO VIRUS | CCL22, IFI44, CCL5, CCL8, CCL4 |
| BIOCARTA IL5 PATHWAY | IL6, IL1B |
| CYTOKINE METABOLIC PROCESS | IL6, IL12B, INHBA, EBI3, IRF4 |
| INTERLEUKIN RECEPTOR ACTIVITY | IL3RA, IL2RG, IL10RA, IL7R |
| REACTOME COSTIMULATION BY THE CD28 FAMILY | ICOSLG, CD86, MAP3K8, CD274, CD80, PDCD1 |
| MULTI ORGANISM PROCESS | CCL22, MAFF, TNIP1, IFI44, CCL5, CCL8, CCL4 |
| POSITIVE REGULATION OF TRANSLATION | IL6, IL12B, EBI3, IRF4 |
| BIOCARTA ASBCELL PATHWAY | CD40, CD80 |
| REGULATION OF CYTOKINE BIOSYNTHETIC PROCESS | IL6, IL12B, INHBA, EBI3, IRF4 |
| BIOCARTA IL10 PATHWAY | IL6, IL10RA, STAT4, STAT3, STAT5A, IL1A |
| NEGATIVE REGULATION OF CYTOKINE BIOSYNTHETIC PROCESS | IL6, INHBA |
| LEUKOCYTE ACTIVATION | ICOSLG, IL12B, INHBA, CLEC7A, LAT, EBI3 |
| BIOCARTA LAIR PATHWAY | IL6, IL1A |
| REGULATION OF T CELL PROLIFERATION | ICOSLG, IL12B, EBI3 |
| NCI CHEMOKINE RECEPTORS BIND CHEMOKINES | CCL20, CCR7 |
| REGULATION OF LYMPHOCYTE ACTIVATION | ICOSLG, IL12B, INHBA, LAT, EBI3 |
| REGULATION OF IMMUNE SYSTEM PROCESS | ICOSLG, IL12B, INHBA, LAT, EBI3, MALT1 |
| NCI IL2 1PATHWAY | IL2RG, SOCS3, FOS, STAT5A, SOCS2, MYC |
| BIOCARTA IL17 PATHWAY | IL6 |
| COAGULATION | F5 |
| T CELL PROLIFERATION | ICOSLG, IL12B, EBI3 |
| REGULATION OF T CELL ACTIVATION | ICOSLG, IL12B, LAT, EBI3 |
| CELL ACTIVATION | ICOSLG, IL12B, INHBA, CLEC7A, LAT, EBI3 |
| REACTOME CELL SURFACE INTERACTIONS AT THE VASCULAR WALL | OLR1, CD48, SLC7A11, CD84 |
| NCI DOWNSTREAM TCR SIGNALING | PRKCQ, NFKBIA, MALT1 |
| REGULATION OF INTERFERON GAMMA BIOSYNTHETIC PROCESS | IL12B, INHBA, EBI3 |
| LYMPHOCYTE ACTIVATION | ICOSLG, IL12B, INHBA, CLEC7A, LAT, EBI3 |
| NCI CD40 PATHWAY | BIRC3, TRAF3, CD40, STAT5A, BIRC2, TRAF1, NFKBIA, TNFAIP3, MYC |
| BIOCARTA TNFR2 PATHWAY | TRAF1, NFKBIA, TNFAIP3, TANK, TRAF3, TNFRSF1B |
| BLOOD COAGULATION | F5 |
| T CELL ACTIVATION | ICOSLG, IL12B, CLEC7A, LAT, EBI3 |
| REACTOME PD1 SIGNALING | CD274, PDCD1 |
| HEMOSTASIS |  |
| VACUOLAR PART |  |
| REACTOME ENDOGENOUS STEROLS |  |
| NCI PD 1 SIGNALING | PDCD1 |
| NETPATH T CELL RECEPTOR PATHWAY DOWN |  |
| NCI CELL SURFACE INTERACTIONS AT THE VASCULAR WALL | OLR1, CD48 |
| BIOCARTA EXTRINSIC PATHWAY |  |
| POSITIVE REGULATION OF CYTOKINE BIOSYNTHETIC PROCESS |  |
| BIOCARTA IL22BP PATHWAY | SOCS3, IL10RA, STAT4, STAT5A |
| HEMATOPOIETIN INTERFERON CLASSD200 DOMAIN CYTOKINE RECEPTOR ACTIVITY |  |
| NCI ENDOGENOUS STEROLS |  |
| GLYCOLIPID METABOLIC PROCESS |  |
| BIOCARTA HIVNEF PATHWAY |  |
| REACTOME IMMUNOREGULATORY INTERACTIONS BETWEEN A LYMPHOID AND A NON LYMPHOID CELL |  |
| NEGATIVE REGULATION OF CELLULAR BIOSYNTHETIC PROCESS |  |
| CELLULAR DEFENSE RESPONSE |  |
| KEGG SYSTEMIC LUPUS ERYTHEMATOSUS |  |
| NCI SYNTHESIS OF BILE ACIDS AND BILE SALTS | CH25H, CYP7B1, HSD3B7 |
| KEGG ASTHMA |  |
| NCI TRYPTOPHAN CATABOLISM |  |
| REACTOME DOWNSTREAM EVENTS IN GPCR SIGNALING |  |
| CYTOKINE PRODUCTION |  |
| REGULATION OF TRANSLATION |  |
| KEGG NATURAL KILLER CELL MEDIATED CYTOTOXICITY | CD48, SH3BP2, PIK3R5, LCP2, PPP3CC, LAT, CSF2, BID |
| REGULATION OF BIOLOGICAL QUALITY |  |
| NCI TCR PATHWAY |  |
| REGULATION OF BODY FLUID LEVELS |  |
| REGULATION OF HORMONE SECRETION | SCG5, INHBA |
| SPHINGOLIPID METABOLIC PROCESS |  |
| LIPID RAFT |  |
| REACTOME DOWNSTREAM TCR SIGNALING |  |
| ACTIVATION OF IMMUNE RESPONSE |  |
| BIOCARTA IL7 PATHWAY |  |
| NEGATIVE REGULATION OF BIOSYNTHETIC PROCESS |  |

| Color legend | | | | | | | | | | | |
| --- | --- | --- | --- | --- | --- | --- | --- | --- | --- | --- | --- |
| q-value | 1 | 0.2 | 0.05 | 0.01 | 0.001 | 0.0001 |
| Color |  | |  |  |  | |

TABLE OF Q-VALUES

| candida albicans moddc135 | stachybotrys chartarum lung | Gene Set |
| --- | --- | --- |
| 0.0 | 8.667609E-4 | KEGG\_CYTOKINE\_CYTOKINE\_RECEPTOR\_INTERACTION |
| 0.0016598669 | 0.068581946 | REACTOME\_GPCR\_LIGAND\_BINDING |
| 0.0 | 0.0027125722 | NETPATH\_IL\_1\_PATHWAY\_UP |
| 2.43421E-4 | 0.0030316834 | RESPONSE\_TO\_EXTERNAL\_STIMULUS |
| 0.0013125496 | 0.029791968 | RESPONSE\_TO\_CHEMICAL\_STIMULUS |
| 0.0 | 0.098460175 | NETPATH\_IL\_2\_PATHWAY\_UP |
| 2.410459E-6 | 1.1035433E-4 | IMMUNE\_SYSTEM\_PROCESS |
| 0.002677061 | 2.8009398E-4 | REACTOME\_SIGNALING\_IN\_IMMUNE\_SYSTEM |
| 2.892551E-6 | 0.1078629 | KEGG\_TOLL\_LIKE\_RECEPTOR\_SIGNALING\_PATHWAY |
| 1.4066127E-4 | 2.021267E-4 | REACTOME\_CLASS\_A1\_RHODOPSIN\_LIKE\_RECEPTORS |
| 0.0034272652 | 0.037194528 | NETPATH\_T\_CELL\_RECEPTOR\_PATHWAY\_UP |
| 2.1694132E-6 | 4.4228967E-5 | DEFENSE\_RESPONSE |
| 2.7103227E-4 | 0.018108694 | NETPATH\_IL\_4\_PATHWAY\_UP |
| 0.0 | 0.17841046 | EXTRACELLULAR\_SPACE |
| 2.2250392E-6 | 0.004897092 | BEHAVIOR |
| 2.1165008E-6 | 0.003132014 | RESPONSE\_TO\_WOUNDING |
| 3.2139455E-6 | 8.301471E-4 | LOCOMOTORY\_BEHAVIOR |
| 0.0 | 3.6784775E-5 | IMMUNE\_RESPONSE |
| 0.0 | 0.012438191 | CYTOKINE\_ACTIVITY |
| 0.009618234 | 0.0064382344 | REACTOME\_G\_ALPHA\_I\_SIGNALLING\_EVENTS |
| 0.006042191 | 0.1397149 | REACTOME\_HEMOSTASIS |
| 2.7117665E-6 | 0.012120066 | NETPATH\_IL\_5\_PATHWAY\_UP |
| 6.846009E-5 | 0.003478014 | KEGG\_CHEMOKINE\_SIGNALING\_PATHWAY |
| 4.0434443E-6 | 3.9669833E-4 | INFLAMMATORY\_RESPONSE |
| 0.0 | 8.2969455E-5 | REACTOME\_PEPTIDE\_LIGAND\_BINDING\_RECEPTORS |
| 0.0 | 7.375062E-5 | CHEMOKINE\_ACTIVITY |
| 2.5522509E-6 | 0.06922398 | KEGG\_NOD\_LIKE\_RECEPTOR\_SIGNALING\_PATHWAY |
| 0.0 | 5.5177166E-5 | REACTOME\_CHEMOKINE\_RECEPTORS\_BIND\_CHEMOKINES |
| 5.8961764E-4 | 0.06361532 | NCI\_NFAT\_TFPATHWAY |
| 2.9922942E-6 | 0.099357516 | KEGG\_JAK\_STAT\_SIGNALING\_PATHWAY |
| 0.011522901 | 0.003299158 | HOMEOSTATIC\_PROCESS |
| 0.0 | 7.93557E-5 | CHEMOKINE\_RECEPTOR\_BINDING |
| 0.0 | 1.8657849E-4 | G\_PROTEIN\_COUPLED\_RECEPTOR\_BINDING |
| 0.0 | 0.16922897 | KEGG\_GRAFT\_VERSUS\_HOST\_DISEASE |
| 0.0 | 0.038418543 | NETPATH\_IL\_7\_PATHWAY\_UP |
| 0.0020074716 | 5.516338E-5 | KEGG\_COMPLEMENT\_AND\_COAGULATION\_CASCADES |
| 0.005975001 | 3.4941523E-4 | CHEMICAL\_HOMEOSTASIS |
| 1.4610786E-4 | 0.0012814513 | KEGG\_INTESTINAL\_IMMUNE\_NETWORK\_FOR\_IGA\_PRODUCTION |
| 0.003281799 | 3.452314E-4 | CELLULAR\_CATION\_HOMEOSTASIS |
| 0.0 | 0.10351314 | NCI\_IL12\_2PATHWAY |
| 0.0040113498 | 3.4417826E-4 | CATION\_HOMEOSTASIS |
| 0.0 | 3.9366656E-4 | NETPATH\_IL\_3\_PATHWAY\_UP |
| 0.0 | 0.010593823 | NETPATH\_IL\_6\_PATHWAY\_UP |
| 0.0 | 0.012549701 | KEGG\_HEMATOPOIETIC\_CELL\_LINEAGE |
| 0.035413742 | 0.012081878 | NCI\_CD8TCRPATHWAY |
| 3.6631514E-5 | 0.01000818 | RESPONSE\_TO\_BIOTIC\_STIMULUS |
| 0.0042384043 | 2.7618292E-4 | ION\_HOMEOSTASIS |
| 0.018229602 | 6.074107E-4 | CELLULAR\_HOMEOSTASIS |
| 0.0010598439 | 0.0390746 | CYTOKINE\_BINDING |
| 3.6304154E-5 | 0.06267426 | KEGG\_PRION\_DISEASES |
| 0.002013036 | 0.13948603 | BIOCARTA\_DC\_PATHWAY |
| 3.0991619E-6 | 0.013637285 | KEGG\_LEISHMANIA\_INFECTION |
| 0.002537575 | 0.009742402 | NETPATH\_IL\_9\_PATHWAY\_UP |
| 0.0077500087 | 0.029798996 | BIOCARTA\_NO2IL12\_PATHWAY |
| 5.676862E-6 | 0.09190098 | INTERLEUKIN\_BINDING |
| 2.6295918E-6 | 0.10779688 | BIOCARTA\_NKT\_PATHWAY |
| 6.656398E-5 | 0.0020648958 | RESPONSE\_TO\_OTHER\_ORGANISM |
| 2.160868E-5 | 0.1276452 | NCI\_CD8TCRDOWNSTREAMPATHWAY |
| 0.002180355 | 0.0064237956 | KEGG\_T\_CELL\_RECEPTOR\_SIGNALING\_PATHWAY |
| 0.020800248 | 0.0037536372 | CYTOKINE\_BIOSYNTHETIC\_PROCESS |
| 0.013854358 | 0.17213066 | REACTOME\_FORMATION\_OF\_FIBRIN\_CLOT\_CLOTTING\_CASCADE |
| 4.1889373E-4 | 3.5422304E-4 | HUMORAL\_IMMUNE\_RESPONSE |
| 0.009150396 | 0.19903007 | BIOCARTA\_IL12\_PATHWAY |
| 2.2835927E-6 | 0.012842769 | RESPONSE\_TO\_VIRUS |
| 0.0012734811 | 0.16566963 | BIOCARTA\_IL5\_PATHWAY |
| 0.020787546 | 0.0035099161 | CYTOKINE\_METABOLIC\_PROCESS |
| 4.4912662E-4 | 0.037428394 | INTERLEUKIN\_RECEPTOR\_ACTIVITY |
| 0.028571257 | 0.047260914 | REACTOME\_COSTIMULATION\_BY\_THE\_CD28\_FAMILY |
| 0.003974432 | 0.1695661 | MULTI\_ORGANISM\_PROCESS |
| 0.00871612 | 5.324376E-4 | POSITIVE\_REGULATION\_OF\_TRANSLATION |
| 0.0067488668 | 0.14346233 | BIOCARTA\_ASBCELL\_PATHWAY |
| 0.0108003775 | 0.001354138 | REGULATION\_OF\_CYTOKINE\_BIOSYNTHETIC\_PROCESS |
| 2.3067395E-4 | 0.09661621 | BIOCARTA\_IL10\_PATHWAY |
| 0.050513387 | 0.10721472 | NEGATIVE\_REGULATION\_OF\_CYTOKINE\_BIOSYNTHETIC\_PROCESS |
| 0.029801909 | 0.025527647 | LEUKOCYTE\_ACTIVATION |
| 0.0023013013 | 0.03715054 | BIOCARTA\_LAIR\_PATHWAY |
| 0.024630219 | 0.1436165 | REGULATION\_OF\_T\_CELL\_PROLIFERATION |
| 3.2052063E-4 | 0.037954282 | NCI\_CHEMOKINE\_RECEPTORS\_BIND\_CHEMOKINES |
| 0.0015481856 | 0.09924895 | REGULATION\_OF\_LYMPHOCYTE\_ACTIVATION |
| 4.633083E-4 | 0.179525 | REGULATION\_OF\_IMMUNE\_SYSTEM\_PROCESS |
| 0.0128200315 | 0.09984908 | NCI\_IL2\_1PATHWAY |
| 1.6053132E-4 | 0.08465764 | BIOCARTA\_IL17\_PATHWAY |
| 0.002985779 | 0.13898392 | COAGULATION |
| 0.025794454 | 0.11078743 | T\_CELL\_PROLIFERATION |
| 0.015937276 | 0.09062176 | REGULATION\_OF\_T\_CELL\_ACTIVATION |
| 0.02619427 | 0.030370101 | CELL\_ACTIVATION |
| 0.035054013 | 0.13950141 | REACTOME\_CELL\_SURFACE\_INTERACTIONS\_AT\_THE\_VASCULAR\_WALL |
| 0.0038455338 | 0.093958445 | NCI\_DOWNSTREAM\_TCR\_SIGNALING |
| 0.0028675264 | 0.1687969 | REGULATION\_OF\_INTERFERON\_GAMMA\_BIOSYNTHETIC\_PROCESS |
| 0.01019916 | 0.030079752 | LYMPHOCYTE\_ACTIVATION |
| 1.14572584E-4 | 0.10266698 | NCI\_CD40\_PATHWAY |
| 9.2645256E-5 | 0.1395269 | BIOCARTA\_TNFR2\_PATHWAY |
| 0.0028893442 | 0.15122637 | BLOOD\_COAGULATION |
| 0.029343517 | 0.05428354 | T\_CELL\_ACTIVATION |
| 0.005299703 | 0.095099404 | REACTOME\_PD1\_SIGNALING |
| 0.19500217 | 0.17265753 | HEMOSTASIS |
| 0.117716365 | 0.12727696 | VACUOLAR\_PART |
| 0.1172772 | 0.0929073 | REACTOME\_ENDOGENOUS\_STEROLS |
| 0.014557132 | 0.123761244 | NCI\_PD\_1\_SIGNALING |
| 0.15405947 | 0.0912635 | NETPATH\_T\_CELL\_RECEPTOR\_PATHWAY\_DOWN |
| 0.023967756 | 0.08937411 | NCI\_CELL\_SURFACE\_INTERACTIONS\_AT\_THE\_VASCULAR\_WALL |
| 0.07859949 | 0.1152361 | BIOCARTA\_EXTRINSIC\_PATHWAY |
| 0.073085286 | 0.0047633494 | POSITIVE\_REGULATION\_OF\_CYTOKINE\_BIOSYNTHETIC\_PROCESS |
| 0.04441359 | 0.1804909 | BIOCARTA\_IL22BP\_PATHWAY |
| 0.07319358 | 0.15185583 | HEMATOPOIETIN\_INTERFERON\_CLASSD200\_DOMAIN\_CYTOKINE\_RECEPTOR\_ACTIVITY |
| 0.19913463 | 0.1238783 | NCI\_ENDOGENOUS\_STEROLS |
| 0.088080995 | 0.15965135 | GLYCOLIPID\_METABOLIC\_PROCESS |
| 0.19104342 | 0.13998525 | BIOCARTA\_HIVNEF\_PATHWAY |
| 0.08234739 | 0.0013304139 | REACTOME\_IMMUNOREGULATORY\_INTERACTIONS\_BETWEEN\_A\_LYMPHOID\_AND\_A\_NON\_LYMPHOID\_CELL |
| 0.06449941 | 0.15733238 | NEGATIVE\_REGULATION\_OF\_CELLULAR\_BIOSYNTHETIC\_PROCESS |
| 0.109449334 | 0.0013074436 | CELLULAR\_DEFENSE\_RESPONSE |
| 0.08947626 | 0.005506665 | KEGG\_SYSTEMIC\_LUPUS\_ERYTHEMATOSUS |
| 0.024552073 | 0.068042584 | NCI\_SYNTHESIS\_OF\_BILE\_ACIDS\_AND\_BILE\_SALTS |
| 0.0061685643 | 0.09246591 | KEGG\_ASTHMA |
| 0.18769778 | 0.1546794 | NCI\_TRYPTOPHAN\_CATABOLISM |
| 0.057645433 | 0.1596031 | REACTOME\_DOWNSTREAM\_EVENTS\_IN\_GPCR\_SIGNALING |
| 0.059200067 | 0.004944692 | CYTOKINE\_PRODUCTION |
| 0.10621182 | 0.045171976 | REGULATION\_OF\_TRANSLATION |
| 0.032976635 | 0.015356489 | KEGG\_NATURAL\_KILLER\_CELL\_MEDIATED\_CYTOTOXICITY |
| 0.104142815 | 0.09474054 | REGULATION\_OF\_BIOLOGICAL\_QUALITY |
| 0.17708118 | 0.002480163 | NCI\_TCR\_PATHWAY |
| 0.19393124 | 0.12395742 | REGULATION\_OF\_BODY\_FLUID\_LEVELS |
| 0.020191431 | 0.16944346 | REGULATION\_OF\_HORMONE\_SECRETION |
| 0.052274436 | 0.10375146 | SPHINGOLIPID\_METABOLIC\_PROCESS |
| 0.090893894 | 0.11064813 | LIPID\_RAFT |
| 0.16647865 | 0.13418262 | REACTOME\_DOWNSTREAM\_TCR\_SIGNALING |
| 0.16686626 | 0.09125618 | ACTIVATION\_OF\_IMMUNE\_RESPONSE |
| 0.153053 | 0.13623825 | BIOCARTA\_IL7\_PATHWAY |
| 0.06904809 | 0.15170847 | NEGATIVE\_REGULATION\_OF\_BIOSYNTHETIC\_PROCESS |
