# Supplementary material for: Computational approaches for discovery of common immunomodulators in fungal infections: towards broad-spectrum immunotherapeutic interventions
Source: BMC Microbiol. 2013 Oct 7;13:224. doi: 10.1186/1471-2180-13-224 (PMC3853472; doi:10.1186/1471-2180-13-224)
Supplement: Additional file 1 — Details of up- and down- regulated biclusters. [file 1471-2180-13-224-S1.zip › 2013-kidane-bmc/details-of-biclusters/upreg-biclust-8.html]

**BICLUSTER\_ID** : UPREG-8  
**PATHOGENS** /2/ : a. fumigatus,c. albicans  
**KNOWN DRUG TARGETS** /42/ : RB1, CYP1B1, ASNS, PTK2, UGCG, MAP2K1, EGFR, NFKB1, IGF1R, GARS, GSK3B, CDK7, PDPK1, FYN, ICAM1, CCL20, UAP1, JUN, LDLR, PDE4B, CCL2, CASP3, PLAU, ABL1, SLC7A1, PIM1, CD55, ADRBK1, FGF2, IFNGR1, CHD1, IFNGR2, GJA1, IL6, PTGS2, PPAT, CLK1, CREB1, MAPKAPK2, IL8, PLAUR, GRB2  

| Gene Set | Leading Edge Genes |
| --- | --- |
| NETPATH TGFBETA RECEPTOR PATHWAY UP | BDNF, HBEGF, ASNS, NFKBIA, EIF2C2, INHBA, ZNF143, TDG, STAM, RGS2, APBB2, PTPRK, PDGFA, GOLGA4, JUNB, MAPK6, TNFRSF12A, TMEPAI, NFIL3, JAG1, HIVEP2, RELA, FOSL1, PTHLH, PLAU, IER3, FGF2, GDF15, RAPGEF2, DUSP5, NUP153, KLF10, SPRY2, ATP6V0A2, SMAD3, IL6, ATF3, PTGS2, PMP22, JUP, SLC7A5, SERPINB8, DDX21, TRIB3, MAP3K8, CHST11, PHLDA2, CYP1B1, GTPBP2, EXT1, PTPN2, RELB, BHLHB2, MAP2K1, EGFR, CAB39, SGK, SMAD2, GARS, CBFB, VEGF, TOPORS, FYN, RYBP, FOXO1A, FST, CRY1, SMURF2, ICAM1, MYC, MXI1, GADD45B, UAP1, DVL3, CASP3, VEGFC, SLC7A1, MAP3K2, SRPX2, NEDD9, DSCR1, KIAA0247, NRIP1, DDIT4, ATP1B1, ETS2, CLK1, TANK, SNF1LK, SMURF1, MAPKAPK2, DAXX, PLAUR |
| NETPATH IL 2 PATHWAY UP | DDX21, PFKFB3, ABCE1, NOLC1, BTG3, DUSP6, PMAIP1, ASNS, RRAS2, FOS, CREM, BHLHB2, CFLAR, NFKB1, SGK, APBB2, MFHAS1, GARS, CBFB, MAP3K7IP2, DENND3, OXSR1, VEGF, NFIL3, FYN, ICAM1, IRF1, MYC, AHR, ZNF267, LDLR, JUN, PDE4B, MMD, FRMD4B, SOS1, CASP3, SLC2A3, RELA, NR4A2, PLAU, PRKCD, PIM1, FOSL2, PPRC1, IER3, TTK, DUSP5, ATP1B1, ETS2, USP15, SAMHD1, JUND, IL6, MAPKAPK2, DAXX, IL8, SLC7A5, CHSY1, PLAUR |
| NCI LYSOPHOSPHOLIPID PATHWAY | NFKB1, JUN, HBEGF, GSK3B, CASP3, RELA, NFKBIA, IL6, PRKCD, PTK2, IL8, FOS, GNAI1, EGFR |
| RESPONSE TO EXTERNAL STIMULUS | CCL20, CCL2, RELA, STC1, PLAU, FOSL1, STC2, RIPK2, FOS, CXCL2, FGF2, MAP2K1, NFKB1, CXCL1, CEBPB, IL1A, IL8, ADM, PLAUR |
| KEGG CHEMOKINE SIGNALING PATHWAY | CCL20, CCL2, SOS1, RELA, CXCL3, NFKBIA, PIK3CA, PRKCD, PTK2, CXCL2, ADRBK1, GNAI1, MAP2K1, GNB5, NFKB1, CXCL1, GSK3B, PLCB1, IL8 |
| KEGG CYTOKINE CYTOKINE RECEPTOR INTERACTION | TNFRSF10B, CCL20, CCL2, VEGFC, CXCL3, LEPR, INHBA, CXCL2, EGFR, PDGFB, PDGFA, CXCL1, IFNGR1, TNFRSF12A, IL1A, IFNGR2, IL6, IL8 |
| NEGATIVE REGULATION OF PROGRAMMED CELL DEATH | MCL1, CCL2, CASP3, RELA, BCL2L2, PIM1, IER3, CFLAR, NFKB1, BIRC3, IGF1R, BCL3, IL1A, ANGPTL4, GSK3B, BCL10, TNFAIP8, IL6, SFRP1, TAX1BP1, PSEN1, BNIP3 |
| NEGATIVE REGULATION OF APOPTOSIS | MCL1, CCL2, CASP3, RELA, BCL2L2, PIM1, IER3, CFLAR, NFKB1, BIRC3, IGF1R, BCL3, IL1A, ANGPTL4, GSK3B, BCL10, TNFAIP8, IL6, SFRP1, TAX1BP1, PSEN1, BNIP3 |
| KEGG MAPK SIGNALING PATHWAY | GADD45B, BDNF, MAP3K8, SOS1, DUSP6, CASP3, RELA, RASA2, MAP4K3, RRAS2, MAP3K2, RELB, FOS, FGF2, EGFR, MAP2K1, PDGFB, NFKB1, DUSP5, RAPGEF2, PDGFA, NR4A1, STK3, PPP3CB, IL1A, JUND, PPM1B, MAPKAPK2, DAXX, MYC, DUSP1 |
| NETPATH IL 4 PATHWAY DOWN | PDGFB, CCL20, GADD45B, KLHL18, CCL2, DDIT4, NFKBIA, IL6, GEM, PTGS2, IRF1, IL8, CXCL2, EGFR |
| BEHAVIOR | CCL20, CXCL1, FOSB, CCL2, PLAU, FOSL1, IL8, CXCL2, PLAUR, FGF2 |
| NETPATH TNF ALPHA PATHWAY DOWN | JMJD1A, NFKBIA, ZNF131, EGR1, CXCL2, BHLHB2, NFKB1, CXCL1, ZHX2, JUNB, FOSB, MAPK6, GSK3B, TIPARP, VEGF, MAFF, TNFAIP2, GEM, TNFAIP3, IRF1, CCL20, MTMR2, CXCL3, SDC4, FOSL2, IER3, REL, NR4A1, PPP1R15A, KLF10, MNT, DDX3X, IL6, CLK1, PTGS2, ZFP36 |
| LOCOMOTORY BEHAVIOR | CCL20, CXCL1, CCL2, PLAU, FOSL1, IL8, CXCL2, PLAUR, FGF2 |
| NETPATH IL 5 PATHWAY UP | IRF2, PDE4B, MMD, CCL2, NFKBIE, HBEGF, DUSP6, RELA, EGR1, MAP3K2, FOSL2, CD55, RELB, IER3, SGK, NFKB1, BIRC3, DUSP5, REL, TRAF3, MAPK6, IL1A, RAB21, NFIL3, PMP22, ICAM1, IL8, IER2 |
| NETPATH IL 1 PATHWAY UP | NR4A3, CCL20, NFKBIE, CCL2, DUSP6, RELA, FOSL1, CXCL3, NFKBIA, PRKCD, CXCL2, SOD2, NFKB1, TTK, BIRC3, CXCL1, JMJD3, IL6, PTGS2, ZFP36, IL8, MYC |
| TRANSCRIPTION FROM RNA POLYMERASE II PROMOTER | RB1, SNW1, NFAT5, ELL2, E2F3, ZNF143, INHBA, TAF2, KLF7, FOS, SOD2, NFKB1, SMAD2, KLF11, CEBPZ, CBFB, RBBP8, TAF1A, FOSB, ELF4, CEBPB, JUNB, CDK7, NFIL3, MAFF, HSF2, FST, RYBP, ZBTB38, IRF1, MYC, IRF2, AHR, ETV1, TIAL1, COPS2, CREB5, ABL1, TCEB3, SQSTM1, FOSL1, PLAGL1, FOXK2, VEZF1, ELF2, FOSL2, E2F6, CEBPD, CHD1, SP2, KLF10, MNT, NRIP1, SMAD3, GTF2H1, RNF4, TAF5, PBX3, JUND, ARID4A, ZMYND11 |
| NETPATH IL 4 PATHWAY UP | AHR, SLC38A1, CCL2, ELL2, SOS1, DUSP6, FJX1, CASP3, RELA, CYP1B1, RASA2, ARL4C, RIPK2, FOS, CREM, GNAI1, PMCH, GATA6, RGS2, NFKB1, REL, PSCD1, CEBPB, BCL3, NFIL3, IL6, JAG1, MYC |
| KEGG T CELL RECEPTOR SIGNALING PATHWAY | JUN, NFAT5, NFKBIE, MAP3K8, SOS1, RELA, NFKBIA, PIK3CA, PDK1, CBLB, FOS, MAP2K1, NFKB1, PPP3CB, GSK3B, BCL10, FYN |
| NCI MET PATHWAY | MAP3K5, JUN, SOS1, PDPK1, PIK3CA, PTK2, FOS, MAP2K1, GRB2 |
| NETPATH IL 2 PATHWAY | NFKB1, IRS2, SHB, SOS1, RELA, ETS2, FYN, PIK3CA, CREB1, PLCB1, GAB2, GRB2, STAM, MAP2K1 |
| EXTRACELLULAR SPACE | CCL20, CXCL1, CCL2, FJX1, CXCL3, TNFAIP2, IL6, ADM, IL8, CXCL2, FGF2 |
| BIOCARTA MET PATHWAY | PIK3CA, PTK2, JUN, FOS, SOS1, MAP2K1, GRB2 |
| KEGG NOD LIKE RECEPTOR SIGNALING PATHWAY | NFKB1, BIRC3, CXCL1, CCL2, BIRC2, RELA, NFKBIA, IL6, TNFAIP3, CXCL2, IL8, RIPK2 |
| REACTOME TOLL LIKE RECEPTOR 3 CASCADE | NFKB1, ATF1, JUN, NFKB2, DUSP3, RIPK1, DUSP6, TBK1, RELA, NFKBIA, PPP2R1B, MAPKAPK2, FOS, TICAM1, MAP2K1 |
| KEGG B CELL RECEPTOR SIGNALING PATHWAY | NFKB1, JUN, NFAT5, NFKBIE, PPP3CB, GSK3B, SOS1, BCL10, RELA, NFKBIA, PIK3CA, FOS, MAP2K1 |
| NCI REG GR PATHWAY | NFKB1, SGK, NR4A1, GSK3B, EGR1, IL6, IRF1, FOS, IL8 |
| NETPATH EGFR1 PATHWAY UP | EMP1, NR4A3, DUSP6, PHLDA2, PTHLH, PLAU, EGR1, SDC4, IER3, NAV3, SPRY2, VEGF, TNFAIP3, PPAT, PTGS2, MYC, PLAUR, DUSP1 |
| NCI AMB2 NEUTROPHILS PATHWAY | PLAU, NFKB1, IL6, PLAUR |
| NCI IL2 1PATHWAY | IRS2, JUN, SOS1, PIK3CA, FYN, MAPKAPK2, FOS, MYC, MAP2K1, STAM |
| BIOCARTA EGF PATHWAY | SRF, JUN, SOS1, PIK3CA, FOS, EGFR, GRB2, MAP2K1 |
| BIOCARTA KERATINOCYTE PATHWAY | NFKB1, JUN, RELA, ETS2, NFKBIA, PRKCD, DAXX, FOS, EGFR, MAP2K1 |
| REACTOME PEPTIDE LIGAND BINDING RECEPTORS | CXCL3, CCL20, CXCL1, CCL2, CXCL2, IL8 |
| BIOCARTA IL1R PATHWAY | NFKB1, NFKBIA, IL6, JUN, IL1A, RELA |
| NCI MAPKTRKPATHWAY | SRF, NRAS, EHD4, PRKCD, EGR1, KRAS, MAPKAPK2, CREB1, FOS, MAP3K2, MAP2K1 |
| NCI FGF PATHWAY | SOS1, SPRY2, PLAU, PIK3CA, FOS, SSH1, FGF2, PLAUR |
| BIOCARTA CDMAC PATHWAY | NFKB1, NFKBIA, JUN, FOS, MYC, MAP2K1, RELA |
| CYTOKINE ACTIVITY | CXCL3, GDF15, CCL20, CXCL1, CCL2, CXCL2, IL8 |
| BIOCARTA IGF1 PATHWAY | SRF, PIK3CA, IGF1R, JUN, FOS, SOS1, MAP2K1 |
| CHEMOKINE ACTIVITY | CXCL3, CCL20, CXCL1, CCL2, IL8, CXCL2 |
| DEFENSE RESPONSE | CD83, CCL20, RELA, FOSL1, VEZF1, INHBA, RIPK2, FOS, CXCL2, NFKB1, TNIP1, CXCL1, CEBPB, IL1A, BCL10, IL8, BNIP3 |
| CHEMOKINE RECEPTOR BINDING | CXCL3, CCL20, CXCL1, CCL2, IL8, CXCL2 |
| REACTOME TRAF6 MEDIATED INDUCTION OF THE ANTIVIRAL CYTOKINE IFN ALPHA BETA CASCADE | NFKB1, ATF1, JUN, DUSP3, DUSP6, RELA, NFKBIA, FOS, TICAM1, MAP2K1 |
| REACTOME CHEMOKINE RECEPTORS BIND CHEMOKINES | CXCL3, CCL20, CXCL1, CCL2, CXCL2, IL8 |
| BIOCARTA CARDIACEGF PATHWAY | NFKB1, JUN, FOS, MYC, EGFR, RELA |
| NCI IL23PATHWAY | NFKB1, NFKBIA, IL6, PIK3CA, CXCL1, CCL2, RELA |
| BIOCARTA PDGF PATHWAY | SRF, PDGFA, JUN, SOS1, PIK3CA, FOS, GRB2, MAP2K1 |
| G PROTEIN COUPLED RECEPTOR BINDING | CXCL3, CCL20, CXCL1, CCL2, IL8, CXCL2 |
| NCI AVB3 OPN PATHWAY | PLAU, NFKB1, NFKBIA, PIK3CA, JUN, FOS, RELA |
| INFLAMMATORY RESPONSE | NFKB1, CCL20, CXCL1, CEBPB, IL1A, CXCL2, RIPK2, IL8, FOS |
| BIOCARTA TPO PATHWAY | PIK3CA, JUN, FOS, SOS1, MAP2K1, GRB2 |
| NETPATH WNT PATHWAY UP | SIAH2, KLF10, BTG3, MAPK6, DUSP6, TRRAP, GJA1, IL8, FOS, CREM, DUSP1, PLAUR |
| ST B CELL ANTIGEN RECEPTOR | NFKB1, NFKBIA, PIK3CA, NFAT5, NFKBIE, SOS1, MAP2K1 |
| NCI ATF2 PATHWAY | PLAU, JUND, IL6, ATF3, DUSP5, FOS, DUSP1 |
| BIOCARTA STEM PATHWAY | IL6, IL8 |
| BIOCARTA INSULIN PATHWAY | SRF, PIK3CA, JUN, FOS, SOS1, MAP2K1 |
| BIOCARTA NKT PATHWAY | IFNGR1, CSF2, IFNGR2 |
| NCI IL6 7PATHWAY | JUN, MCL1, JUNB, CEBPB, SOS1, MITF, IL6, PRKCD, PIK3CA, IRF1, FOS, MYC |
| NCI DISSOLUTION OF FIBRIN CLOT | PLAU, PLAUR |
| NETPATH KIT RECEPTOR PATHWAY UP | GDF15, MCL1, JUNB, CCL2, PPP3CB, ETV5, VEGF, JUND, IL6, EGR1, FOS, MYC, IER3 |
| ST ERK1 ERK2 MAPK PATHWAY | NFKB1, ATF1, TRAF3, MAP3K8, DUSP6, SOS1, CREB5, MAP2K1 |
| BIOCARTA NTHI PATHWAY | SMAD3, NFKB1, NFKBIA, IL8, SMAD4, DUSP1, RELA |
| NCI NFAT TFPATHWAY | FOSL1, EGR1, CBLB, PTGS2, JUNB, IL8, FOS |
| NETPATH IL 3 PATHWAY UP | NFIL3, MCL1, CCL2, FOS, IL8, MYC |
| BIOCARTA INFLAM PATHWAY | IL6, PDGFA, IL8, CSF2, IL1A |
| BIOCARTA IL17 PATHWAY | IL6, IL8 |
| REACTOME G ALPHA I SIGNALLING EVENTS | CXCL3, OPN3, CCL20, CXCL1, CXCL2, IL8, GNAI1, GNB5 |
| BIOCARTA GRANULOCYTES PATHWAY | ICAM1, IL8, IL1A |
| BIOCARTA GLEEVEC PATHWAY | PIK3CA, JUN, FOS, MYC, SOS1, MAP2K1 |
| NCI TCRCALCIUMPATHWAY | FOSL1, PTGS2, JUNB, FOS, PPP3CB |
| NETPATH IL 9 PATHWAY UP | CCL2, JUNB, BCL3, MYC |
| NCI PDGFRAPATHWAY | PDGFB, SRF, PDGFA, JUN, SHB, SOS1, PIK3CA, FOS |
| NCI NFKAPPABATYPICALPATHWAY | NFKB1, REL, BCL3, CSNK2A2, RELA, NFKBIA, PIK3CA, FBXW11 |
| FEEDING BEHAVIOR | FYN, PMCH |
| CORUM CHUK-NFKB2-REL-IKBKG-SPAG9-NFKB1-NFKBIE-COPB2-TNIP1-NFKBIA-RELA-TNIP2 COMPLEX | NFKB1, NFKBIA, SPAG9, REL, TNIP1, NFKBIE, RELA |
| BIOCARTA CD40 PATHWAY | NFKB1, NFKBIA, TNFAIP3, TRAF3, DUSP1, RELA |
| BIOCARTA ERYTH PATHWAY | IL6, CSF2, IL1A |
| BIOCARTA RANKL PATHWAY | FOSL1, NFKB1, FOS, FOSL2, RELA |
| NCI CHEMOKINE RECEPTORS BIND CHEMOKINES | CCL20 |
| SIG CD40PATHWAYMAP | NFKB1, NFKBIA, PIK3CA, NFKBIE, TRAF3, DUSP1 |
| NETPATH IL 6 PATHWAY UP | SGK, GADD45B, CXCL1, LDLR, MCL1, JUNB, CEBPB, BCL3, SLC2A3, MAFF, ZFP36, IRF1, FOS, BHLHB2 |
| NCI CD40 PATHWAY | NFKB1, BIRC3, TRAF3, BIRC2, RELA, NFKBIA, TNFAIP3, CBLB, MYC |
| ST T CELL SIGNAL TRANSDUCTION | NFKB1, NFKBIA, NFAT5, NFKBIE, SOS1 |
| BIOCARTA IL2 PATHWAY | FOS, SOS1, MAP2K1 |
| POSITIVE REGULATION OF TRANSLATION | IL6, BCL3, SAMD4A, BCL10 |
| NCI CD8TCRDOWNSTREAMPATHWAY | FOSL1, EGR1, JUNB, FOS, PPP3CB, MAP2K1 |
| SPHINGOLIPID BIOSYNTHETIC PROCESS | UGCG |
| BIOCARTA TNFR2 PATHWAY | NFKB1, NFKBIA, TNFAIP3, TANK, TRAF3, DUSP1, RELA |
| NCI IL12 2PATHWAY | NFKB1, GADD45B, RIPK2, RELB, FOS, PPP3CB |
| NCI NFKAPPABCANONICALPATHWAY | NFKB1, CYLD, MALT1, BIRC2, BCL10, RELA, NFKBIA, TNFAIP3, RIPK2, FBXW11 |
| LEUKOCYTE ACTIVATION |  |
| BIOCARTA IL6 PATHWAY |  |
| BIOCARTA SPRY PATHWAY |  |
| NETPATH IL 1 PATHWAY |  |
| PROTEIN AMINO ACID DEPHOSPHORYLATION | PPM1D, PPM1B, DUSP5, DUSP3, PTPN12, MTMR3, DUSP6, SSH1 |
| NETPATH IL 7 PATHWAY |  |
| TISSUE MORPHOGENESIS | SMAD2 |
| BIOCARTA LAIR PATHWAY |  |
| BIOCARTA LYM PATHWAY |  |
| PHOSPHOPROTEIN PHOSPHATASE ACTIVITY | PPM1D, DUSP5, PTPRK, PPAP2B, DUSP3, MTMR3, DUSP6, PPM1B, PTPN2, PTPN12, DUSP1 |
| PROTEIN TYROSINE PHOSPHATASE ACTIVITY | DUSP5, DUSP3, PTPN12, MTMR3, DUSP6, DUSP1 |
| CORUM TNF-ALPHA/NF-KAPPA B SIGNALING COMPLEX CHUK KPNA3 NFKB2 NFKBIB REL IKBKG NFKB1 NFKBIE RELB NFKBIA RELA TNIP2 |  |
| NETPATH HEDGEHOG PATHWAY UP | VEGF, NR4A1, PMP22, MYC |
| NEGATIVE REGULATION OF RNA METABOLIC PROCESS | IRF2, E2F6, SMAD2, SIRT1, KLF11, ZHX2, FOSB, KLF10, NRIP1, SMAD3, FST, RYBP, DAXX, SMAD4, ARID4A, ZMYND11 |
| LEUKOCYTE MIGRATION |  |
| LEUKOCYTE CHEMOTAXIS |  |
| BIOCARTA 41BB PATHWAY |  |
| CORUM TNF-ALPHA/NF-KAPPA B SIGNALING COMPLEX RPL6 RPL30 RPS13 CHUK DDX3X NFKB2 NFKBIB REL IKBKG NFKB1 MAP3K8 RELB GLG1 NFKBIA RELA TNIP2 GTF2I |  |
| NCI IL12 STAT4PATHWAY | FOS, IRF1, PPP3CB, ETV5 |
| NCI PDGFRBPATHWAY |  |
| NEGATIVE REGULATION OF TRANSCRIPTION DNA DEPENDENT | IRF2, E2F6, SMAD2, SIRT1, KLF11, ZHX2, FOSB, KLF10, NRIP1, SMAD3, FST, RYBP, DAXX, SMAD4, ARID4A, ZMYND11 |

| Color legend | | | | | | | | | | | |
| --- | --- | --- | --- | --- | --- | --- | --- | --- | --- | --- | --- |
| q-value | 1 | 0.2 | 0.05 | 0.01 | 0.001 | 0.0001 |
| Color |  | |  |  |  | |

TABLE OF Q-VALUES

| candida albicans huvec | aspergillus fumigatus conidia a549 | Gene Set |
| --- | --- | --- |
| 0.04716648 | 0.014604702 | NETPATH\_TGFBETA\_RECEPTOR\_PATHWAY\_UP |
| 0.009711463 | 2.9144692E-4 | NETPATH\_IL\_2\_PATHWAY\_UP |
| 0.19033894 | 0.009436423 | NCI\_LYSOPHOSPHOLIPID\_PATHWAY |
| 0.19306602 | 6.960372E-4 | RESPONSE\_TO\_EXTERNAL\_STIMULUS |
| 0.09618854 | 0.028497338 | KEGG\_CHEMOKINE\_SIGNALING\_PATHWAY |
| 0.0076698544 | 2.5672338E-5 | KEGG\_CYTOKINE\_CYTOKINE\_RECEPTOR\_INTERACTION |
| 0.08243739 | 0.075307034 | NEGATIVE\_REGULATION\_OF\_PROGRAMMED\_CELL\_DEATH |
| 0.07932325 | 0.07917507 | NEGATIVE\_REGULATION\_OF\_APOPTOSIS |
| 0.19037609 | 0.003983041 | KEGG\_MAPK\_SIGNALING\_PATHWAY |
| 0.13495818 | 8.290948E-4 | NETPATH\_IL\_4\_PATHWAY\_DOWN |
| 7.269528E-5 | 1.642341E-5 | BEHAVIOR |
| 6.3579535E-4 | 0.0 | NETPATH\_TNF\_ALPHA\_PATHWAY\_DOWN |
| 5.4281012E-5 | 2.2720385E-5 | LOCOMOTORY\_BEHAVIOR |
| 5.682608E-4 | 1.0097658E-4 | NETPATH\_IL\_5\_PATHWAY\_UP |
| 0.0020151997 | 1.5328516E-5 | NETPATH\_IL\_1\_PATHWAY\_UP |
| 0.16540709 | 0.04315536 | TRANSCRIPTION\_FROM\_RNA\_POLYMERASE\_II\_PROMOTER |
| 0.006071951 | 2.0300957E-5 | NETPATH\_IL\_4\_PATHWAY\_UP |
| 0.046388313 | 0.0076145586 | KEGG\_T\_CELL\_RECEPTOR\_SIGNALING\_PATHWAY |
| 0.17205246 | 0.085733145 | NCI\_MET\_PATHWAY |
| 0.14932056 | 0.07182297 | NETPATH\_IL\_2\_PATHWAY |
| 0.12368768 | 0.07053614 | EXTRACELLULAR\_SPACE |
| 0.096628375 | 0.05619789 | BIOCARTA\_MET\_PATHWAY |
| 0.035266537 | 7.929928E-5 | KEGG\_NOD\_LIKE\_RECEPTOR\_SIGNALING\_PATHWAY |
| 0.17478716 | 0.008573098 | REACTOME\_TOLL\_LIKE\_RECEPTOR\_3\_CASCADE |
| 0.11673039 | 0.049602754 | KEGG\_B\_CELL\_RECEPTOR\_SIGNALING\_PATHWAY |
| 0.059264697 | 0.003923119 | NCI\_REG\_GR\_PATHWAY |
| 0.0052840365 | 2.5547526E-5 | NETPATH\_EGFR1\_PATHWAY\_UP |
| 0.14123398 | 0.0056939363 | NCI\_AMB2\_NEUTROPHILS\_PATHWAY |
| 0.059428915 | 0.024652105 | NCI\_IL2\_1PATHWAY |
| 0.11742049 | 0.02654651 | BIOCARTA\_EGF\_PATHWAY |
| 0.09448266 | 0.031115418 | BIOCARTA\_KERATINOCYTE\_PATHWAY |
| 0.015921747 | 1.9160645E-5 | REACTOME\_PEPTIDE\_LIGAND\_BINDING\_RECEPTORS |
| 0.13068527 | 0.070430465 | BIOCARTA\_IL1R\_PATHWAY |
| 0.09780717 | 0.094538905 | NCI\_MAPKTRKPATHWAY |
| 0.14202769 | 0.017533444 | NCI\_FGF\_PATHWAY |
| 0.07875448 | 0.050761063 | BIOCARTA\_CDMAC\_PATHWAY |
| 0.0049860743 | 2.0902522E-5 | CYTOKINE\_ACTIVITY |
| 0.13522848 | 0.04326642 | BIOCARTA\_IGF1\_PATHWAY |
| 0.0024792356 | 2.8400484E-5 | CHEMOKINE\_ACTIVITY |
| 0.104973674 | 0.005702261 | DEFENSE\_RESPONSE |
| 0.0022271401 | 0.0 | CHEMOKINE\_RECEPTOR\_BINDING |
| 0.14167826 | 0.008140157 | REACTOME\_TRAF6\_MEDIATED\_INDUCTION\_OF\_THE\_ANTIVIRAL\_CYTOKINE\_IFN\_ALPHA\_BETA\_CASCADE |
| 0.0 | 0.0 | REACTOME\_CHEMOKINE\_RECEPTORS\_BIND\_CHEMOKINES |
| 0.09701839 | 0.012665072 | BIOCARTA\_CARDIACEGF\_PATHWAY |
| 0.0014167366 | 1.4370484E-5 | NCI\_IL23PATHWAY |
| 0.123684034 | 0.026266055 | BIOCARTA\_PDGF\_PATHWAY |
| 0.08719925 | 1.8933655E-5 | G\_PROTEIN\_COUPLED\_RECEPTOR\_BINDING |
| 0.077203505 | 0.058076452 | NCI\_AVB3\_OPN\_PATHWAY |
| 0.14119071 | 6.0851693E-5 | INFLAMMATORY\_RESPONSE |
| 0.16690448 | 0.048348818 | BIOCARTA\_TPO\_PATHWAY |
| 0.068515584 | 6.440544E-4 | NETPATH\_WNT\_PATHWAY\_UP |
| 0.045818783 | 0.044324104 | ST\_B\_CELL\_ANTIGEN\_RECEPTOR |
| 0.026835954 | 2.2992774E-5 | NCI\_ATF2\_PATHWAY |
| 0.14155315 | 0.02162187 | BIOCARTA\_STEM\_PATHWAY |
| 0.1247653 | 0.034434684 | BIOCARTA\_INSULIN\_PATHWAY |
| 5.1760615E-4 | 0.11278513 | BIOCARTA\_NKT\_PATHWAY |
| 0.043364815 | 0.009613878 | NCI\_IL6\_7PATHWAY |
| 0.10528463 | 0.01466968 | NCI\_DISSOLUTION\_OF\_FIBRIN\_CLOT |
| 0.009533154 | 0.003725802 | NETPATH\_KIT\_RECEPTOR\_PATHWAY\_UP |
| 0.14128143 | 0.09501592 | ST\_ERK1\_ERK2\_MAPK\_PATHWAY |
| 0.085064895 | 0.017831605 | BIOCARTA\_NTHI\_PATHWAY |
| 0.031072352 | 1.4434879E-4 | NCI\_NFAT\_TFPATHWAY |
| 0.04616931 | 2.8740968E-5 | NETPATH\_IL\_3\_PATHWAY\_UP |
| 0.023040734 | 0.01884623 | BIOCARTA\_INFLAM\_PATHWAY |
| 0.045749582 | 0.045313723 | BIOCARTA\_IL17\_PATHWAY |
| 0.14138973 | 0.017489815 | REACTOME\_G\_ALPHA\_I\_SIGNALLING\_EVENTS |
| 0.08973177 | 0.04560868 | BIOCARTA\_GRANULOCYTES\_PATHWAY |
| 0.1752864 | 0.0937287 | BIOCARTA\_GLEEVEC\_PATHWAY |
| 0.08715484 | 0.0031213323 | NCI\_TCRCALCIUMPATHWAY |
| 0.14094914 | 1.0158219E-4 | NETPATH\_IL\_9\_PATHWAY\_UP |
| 0.1407947 | 0.002269441 | NCI\_PDGFRAPATHWAY |
| 0.04089652 | 0.106404856 | NCI\_NFKAPPABATYPICALPATHWAY |
| 0.0021286034 | 0.028019777 | FEEDING\_BEHAVIOR |
| 0.08393664 | 0.02733804 | CORUM\_CHUK-NFKB2-REL-IKBKG-SPAG9-NFKB1-NFKBIE-COPB2-TNIP1-NFKBIA-RELA-TNIP2\_COMPLEX |
| 0.13549858 | 0.019903738 | BIOCARTA\_CD40\_PATHWAY |
| 0.08735396 | 0.09741977 | BIOCARTA\_ERYTH\_PATHWAY |
| 0.097271964 | 0.00397121 | BIOCARTA\_RANKL\_PATHWAY |
| 0.004932953 | 0.009898863 | NCI\_CHEMOKINE\_RECEPTORS\_BIND\_CHEMOKINES |
| 0.09512491 | 0.042247143 | SIG\_CD40PATHWAYMAP |
| 0.0011679985 | 3.6444217E-5 | NETPATH\_IL\_6\_PATHWAY\_UP |
| 0.022629568 | 3.4622008E-5 | NCI\_CD40\_PATHWAY |
| 0.004998011 | 0.01957019 | ST\_T\_CELL\_SIGNAL\_TRANSDUCTION |
| 0.13862748 | 0.10931956 | BIOCARTA\_IL2\_PATHWAY |
| 0.06905987 | 0.02029611 | POSITIVE\_REGULATION\_OF\_TRANSLATION |
| 0.042483065 | 0.009731392 | NCI\_CD8TCRDOWNSTREAMPATHWAY |
| 0.19508676 | 0.077864 | SPHINGOLIPID\_BIOSYNTHETIC\_PROCESS |
| 0.10791961 | 0.011185336 | BIOCARTA\_TNFR2\_PATHWAY |
| 0.0036812855 | 0.0054974956 | NCI\_IL12\_2PATHWAY |
| 0.031076742 | 0.02079193 | NCI\_NFKAPPABCANONICALPATHWAY |
| 0.19483066 | 0.12753585 | LEUKOCYTE\_ACTIVATION |
| 0.09606069 | 0.11664504 | BIOCARTA\_IL6\_PATHWAY |
| 0.19034094 | 0.14502573 | BIOCARTA\_SPRY\_PATHWAY |
| 0.117814705 | 0.17969474 | NETPATH\_IL\_1\_PATHWAY |
| 0.08500121 | 0.019513937 | PROTEIN\_AMINO\_ACID\_DEPHOSPHORYLATION |
| 0.19357568 | 0.15138073 | NETPATH\_IL\_7\_PATHWAY |
| 0.09528401 | 0.06765847 | TISSUE\_MORPHOGENESIS |
| 0.026148528 | 0.118695535 | BIOCARTA\_LAIR\_PATHWAY |
| 0.17139763 | 0.12109292 | BIOCARTA\_LYM\_PATHWAY |
| 0.084417686 | 0.04253726 | PHOSPHOPROTEIN\_PHOSPHATASE\_ACTIVITY |
| 0.04342165 | 0.051919185 | PROTEIN\_TYROSINE\_PHOSPHATASE\_ACTIVITY |
| 0.08624834 | 0.03207342 | CORUM\_TNF-ALPHA/NF-KAPPA\_B\_SIGNALING\_COMPLEX\_CHUK\_KPNA3\_NFKB2\_NFKBIB\_REL\_IKBKG\_\_NFKB1\_NFKBIE\_RELB\_\_NFKBIA\_RELA\_TNIP2 |
| 0.044318005 | 0.006852322 | NETPATH\_HEDGEHOG\_PATHWAY\_UP |
| 0.11734353 | 0.06537605 | NEGATIVE\_REGULATION\_OF\_RNA\_METABOLIC\_PROCESS |
| 0.10777622 | 0.14390759 | LEUKOCYTE\_MIGRATION |
| 0.0823816 | 0.16236398 | LEUKOCYTE\_CHEMOTAXIS |
| 0.10678873 | 0.14658783 | BIOCARTA\_41BB\_PATHWAY |
| 0.08620508 | 0.07160611 | CORUM\_TNF-ALPHA/NF-KAPPA\_B\_SIGNALING\_COMPLEX\_RPL6\_RPL30\_RPS13\_CHUK\_DDX3X\_NFKB2\_NFKBIB\_REL\_IKBKG\_NFKB1\_MAP3K8\_RELB\_GLG1\_NFKBIA\_RELA\_TNIP2\_\_GTF2I |
| 0.1331315 | 0.0014031419 | NCI\_IL12\_STAT4PATHWAY |
| 0.18987893 | 0.11617209 | NCI\_PDGFRBPATHWAY |
| 0.11676378 | 0.06835922 | NEGATIVE\_REGULATION\_OF\_TRANSCRIPTION\_DNA\_DEPENDENT |
