# Supplementary material for: Computational approaches for discovery of common immunomodulators in fungal infections: towards broad-spectrum immunotherapeutic interventions
Source: BMC Microbiol. 2013 Oct 7;13:224. doi: 10.1186/1471-2180-13-224 (PMC3853472; doi:10.1186/1471-2180-13-224)
Supplement: Additional file 1 — Details of up- and down- regulated biclusters. [file 1471-2180-13-224-S1.zip › 2013-kidane-bmc/details-of-biclusters/dnreg-biclust-0.html]

**BICLUSTER\_ID** : DNREG-0  
**PATHOGENS** /2/ : c. albicans,a. fumigatus  
**KNOWN DRUG TARGETS** /21/ : NDUFV2, PDHB, SDHB, NDUFS7, NDUFA3, ATP5D, PSMB1, NDUFB5, NDUFS8, UQCRC1, NDUFB7, NDUFS6, PSMB5, NDUFS4, DCPS, NDUFB1, PPIH, NDUFAB1, SDHD, NDUFS3, NDUFC1  

| Gene Set | Leading Edge Genes |
| --- | --- |
| REACTOME ELECTRON TRANSPORT CHAIN | NDUFS7, SDHB, NDUFA3, COX5B, NDUFB5, UQCRFS1, NDUFS8, NDUFB7, ETFB, NDUFS6, NDUFS4, COX4I1, NDUFB1, UQCRQ, NDUFAB1, SDHD, NDUFS3, NDUFB11, NDUFC1 |
| ORGANELLE ENVELOPE | HTRA2, ABCB6, NDUFS7, ATP5D, ATP5G2, TOMM22, SLC25A11, MPV17, PHB, NUTF2, TIMM13, ATP5O, PPOX, NDUFS4, FIS1, TIMM23, NDUFAB1, SDHD, NDUFS3 |
| MITOCHONDRIAL RESPIRATORY CHAIN | UQCRC1, NDUFAB1, BCS1L, NDUFS4, NDUFS7, NDUFS3, NDUFS8, SURF1 |
| ENVELOPE | HTRA2, ABCB6, NDUFS7, ATP5D, ATP5G2, TOMM22, SLC25A11, MPV17, PHB, NUTF2, TIMM13, ATP5O, PPOX, NDUFS4, FIS1, TIMM23, NDUFAB1, SDHD, NDUFS3 |
| CORUM RESPIRATORY CHAIN COMPLEX I INCOMPLETE INTERMEDIATE MITOCHONDRIAL | NDUFS6, NDUFV2, NDUFS4, NDUFS7 |
| CORUM RESPIRATORY CHAIN COMPLEX I LAMBDA SUBUNIT MITOCHONDRIAL | NDUFS6, NDUFS4, NDUFS7, NDUFS3 |
| AEROBIC RESPIRATION | UQCRC1, PDHB, SDHB, SDHD, OXA1L, SLC25A14 |
| NCI ORC1 REMOVAL FROM CHROMATIN | PSMB5, PSMB8, PSMD10, PSMA2, PSMA5, PSMB10, PSMB6, PSMB1, PSMB9, PSMA4, PSMB3, PSMF1, MCM4, PSME1 |
| REACTOME CDT1 ASSOCIATION WITH THE CDC6 ORC ORIGIN COMPLEX | PSMB5, GMNN, PSMB8, PSMD10, PSMA2, PSMA5, PSMB10, PSMB6, PSMB1, PSMB9, PSMA4, PSMB3, PSMF1, PSME1 |
| NCI APC C CDH1 MEDIATED DEGRADATION OF CDC20 AND OTHER APC C CDH1 TARGETED PROTEINS IN LATE MITOSIS EARLY G1 | PSMB5, PSMB8, PSMD10, PSMA2, PSMA5, PSMB10, PSMB6, PSMB1, PSMB9, PSMA4, PSMB3, ANAPC2, PSMF1, PSME1 |
| NCI AUTODEGRADATION OF CDH1 BY CDH1 APC C | PSMB5, PSMB8, PSMD10, PSMA2, PSMA5, PSMB10, PSMB6, PSMB1, PSMB9, PSMA4, PSMB3, ANAPC2, PSMF1, PSME1 |
| NCI CDT1 ASSOCIATION WITH THE CDC6 ORC ORIGIN COMPLEX | PSMB5, GMNN, PSMB8, PSMD10, PSMA2, PSMA5, PSMB10, PSMB6, PSMB1, PSMB9, PSMA4, PSMB3, PSMF1, PSME1 |
| NCI REMOVAL OF LICENSING FACTORS FROM ORIGINS | PSMB5, GMNN, PSMB8, PSMD10, PSMA2, PSMA5, PSMB10, PSMB6, PSMB1, PSMB9, PSMA4, PSMB3, PSMF1, MCM4, PSME1 |
| NCI CDC20 PHOSPHO APC C MEDIATED DEGRADATION OF CYCLIN A | PSMB5, PSMB8, PSMD10, MAD2L1, PSMA2, PSMA5, PSMB10, PSMB6, PSMB1, PSMB9, PSMA4, PSMB3, ANAPC2, PSMF1, PSME1 |
| REACTOME AUTODEGRADATION OF CDH1 BY CDH1 APC | PSMB5, PSMB8, PSMD10, PSMA2, PSMA5, PSMB10, PSMB6, PSMB1, PSMB9, PSMA4, PSMB3, ANAPC2, PSMF1, PSME1 |
| REACTOME M G1 TRANSITION | PSMB5, GMNN, PSMB8, PSMD10, PSMA2, PSMA5, PSMB10, PSMB6, PSMB1, PSMB9, PSMA4, PSMB3, PSMF1, MCM4, PSME1 |
| NCI SWITCHING OF ORIGINS TO A POST REPLICATIVE STATE | PSMB5, PSMB8, PSMD10, PSMA2, PSMA5, PSMB10, PSMB6, PSMB1, PSMB9, PSMA4, PSMB3, PSMF1, MCM4, PSME1 |
| REACTOME MRNA DECAY BY 3 TO 5 EXORIBONUCLEASE | EXOSC5, DCPS, EXOSC4, EXOSC7, EXOSC2 |
| RIBONUCLEOPROTEIN COMPLEX | RPP40, SNRPC, MRPS16, MRPS12, MRPS11, POP7, SERP1, PPIH, MRPL12, LSM4, MRPL40, LSM6, MRPS28, ZMAT5, MRPS15 |
| ESTABLISHMENT OF VESICLE LOCALIZATION | SNAP29, AP1M2 |
| ORGANELLAR SMALL RIBOSOMAL SUBUNIT |  |
| VESICLE LOCALIZATION | SNAP29, AP1M2 |
| REACTOME RNA POL II CTD PHOSPHORYLATION AND INTERACTION WITH CE | POLR2H, POLR2F, POLR2I, POLR2G |
| PEROXISOME |  |
| NCI RNA POLYMERASE II TRANSCRIPTION PRE INITIATION | POLR2H, TAF12, POLR2F, GTF2H4, POLR2I, GTF2A2, POLR2G |
| REACTOME NUCLEOTIDE EXCISION REPAIR | POLR2H, POLD4, XAB2, POLR2F, ERCC5, POLR2I, POLR2G |
| CORUM F1F0-ATP SYNTHASE EC 3.6.3.14 MITOCHONDRIAL |  |
| NCI HOST INTERACTIONS OF HIV FACTORS |  |
| REACTOME GLUCOSE REGULATION OF INSULIN SECRETION |  |
| CORUM RNA POLYMERASE II RNAPII |  |
| KEGG RNA POLYMERASE | POLR2H, POLR3K, POLR2F, POLR2I, POLR2G |
| NCI FORMATION OF THE EARLY ELONGATION COMPLEX | POLR2H, POLR2F, POLR2I, POLR2G |
| REACTOME SCF BETA TRCP MEDIATED DEGRADATION OF EMI1 |  |
| NCI RNA POLYMERASE II TRANSCRIPTION ELONGATION |  |
| OXIDOREDUCTASE ACTIVITY ACTING ON THE CH CH GROUP OF DONORS |  |
| OXIDOREDUCTASE ACTIVITY ACTING ON NADH OR NADPH |  |
| MITOCHONDRIAL SMALL RIBOSOMAL SUBUNIT |  |
| FATTY ACID METABOLIC PROCESS |  |
| CORUM EXOSOME |  |
| NCI VPU MEDIATED DEGRADATION OF CD4 |  |
| RNA POLYMERASE ACTIVITY |  |
| KEGG PEROXISOME |  |
| CORUM RESPIRATORY CHAIN COMPLEX I INTERMEDIATE VII/650KD MITOCHONDRIAL |  |
| RIBOSOMAL SUBUNIT |  |
| REACTOME TELOMERE MAINTENANCE |  |
| CORUM LSM1-7 COMPLEX | LSM4, LSM6, LSM2, LSM7 |
| KEGG OXIDATIVE PHOSPHORYLATION |  |
| NCI VIRAL MRNA TRANSLATION |  |
| CELLULAR RESPIRATION |  |
| CORUM RNA POLYMERASE II CORE COMPLEX |  |
| REACTOME VIRAL MRNA TRANSLATION |  |
| REACTOME MICRORNA BIOGENESIS |  |
| REACTOME HIV1 TRANSCRIPTION INITIATION | POLR2H, TAF12, POLR2F, GTF2H4, POLR2I, GTF2A2, POLR2G |
| REACTOME P53 INDEPENDENT DNA DAMAGE RESPONSE |  |
| NCI PEPTIDE CHAIN ELONGATION |  |
| NADH DEHYDROGENASE COMPLEX |  |
| NCI RNA POLYMERASE II TRANSCRIPTION INITIATION | POLR2H, TAF12, POLR2F, GTF2H4, POLR2I, GTF2A2, POLR2G |
| NCI RNA POLYMERASE II HIV 1 PROMOTER ESCAPE | POLR2H, TAF12, POLR2F, GTF2H4, POLR2I, GTF2A2, POLR2G |
| KEGG RIBOSOME |  |
| NCI MRNA SPLICING MAJOR PATHWAY |  |
| REACTOME HIV1 TRANSCRIPTION ELONGATION |  |
| NCI SCF BETA TRCP MEDIATED DEGRADATION OF EMI1 |  |
| NCI REGULATION OF ACTIVATED PAK 2P34 BY PROTEASOME MEDIATED DEGRADATION1 |  |
| KEGG PROTEASOME |  |
| NCI VIF MEDIATED DEGRADATION OF APOBEC3G |  |
| CORUM LSM2-8 COMPLEX |  |
| REACTOME FORMATION AND MATURATION OF MRNA TRANSCRIPT | NHP2L1, TAF12, POLR2F, TCEB2, POLR2I, SF3B5, POLR2H, POLR2G, LSM2 |
| POSITIVE REGULATION OF CYTOKINE SECRETION |  |
| NCI HIV 1 TRANSCRIPTION INITIATION | POLR2H, TAF12, POLR2F, GTF2H4, POLR2I, GTF2A2, POLR2G |
| NCI RNA POL II CTD PHOSPHORYLATION AND INTERACTION WITH CE | POLR2H, POLR2F, POLR2I, POLR2G |
| NCI RNA POL II CTD PHOSPHORYLATION AND INTERACTION WITH CE1 | POLR2H, POLR2F, POLR2I, POLR2G |
| NCI TAT MEDIATED ELONGATION OF THE HIV 1 TRANSCRIPT |  |
| NCI FORMATION OF A POOL OF FREE 40S SUBUNITS |  |
| MICROBODY |  |
| CORUM BRCA1-RNA POLYMERASE II COMPLEX | POLR2H, POLR2F, GTF2H4, POLR2I, POLR2G, BRCA1 |
| REACTOME ABORTIVE ELONGATION OF HIV1 TRANSCRIPT IN THE ABSENCE OF TAT |  |
| MITOCHONDRIAL LUMEN |  |
| REACTOME MRNA SPLICING |  |
| NCI FORMATION OF THE HIV 1 EARLY ELONGATION COMPLEX | POLR2H, POLR2F, POLR2I, POLR2G |
| NCI APC C CDC20 MEDIATED DEGRADATION OF SECURIN |  |
| CYTOKINE SECRETION |  |
| REACTOME ELONGATION AND PROCESSING OF CAPPED TRANSCRIPTS | NHP2L1, POLR2F, POLR2I, TCEB2, SF3B5, POLR2H, POLR2G, LSM2 |
| MITOCHONDRIAL RIBOSOME |  |
| RESPIRATORY CHAIN COMPLEX I |  |
| NCI RNA POLYMERASE II TRANSCRIPTION | POLR2H, TAF12, POLR2F, GTF2H4, POLR2I, GTF2A2, POLR2G |
| MITOCHONDRIAL RESPIRATORY CHAIN COMPLEX I |  |
| REACTOME FORMATION OF ATP BY CHEMIOSMOTIC COUPLING |  |
| KEGG BUTANOATE METABOLISM |  |
| KEGG PYRIMIDINE METABOLISM | NUDT2, POLD4, CTPS2, NME3, POLE3, POLR2I, POLR3K, POLR2G |
| REACTOME VIRAL MESSENGER RNA SYNTHESIS |  |
| NCI HIV 1 TRANSCRIPTION ELONGATION | POLR2H, POLR2F, POLR2I, POLR2G |
| ORGANELLAR RIBOSOME |  |
| REGULATION OF CYTOKINE SECRETION |  |
| REACTOME FORMATION OF THE EARLY ELONGATION COMPLEX | POLR2H, POLR2F, POLR2I, POLR2G |
| KEGG PORPHYRIN AND CHLOROPHYLL METABOLISM |  |
| NCI L13A MEDIATED TRANSLATIONAL SILENCING OF CERULOPLASMIN EXPRESSION |  |
| REACTOME P2Y RECEPTORS |  |
| CORUM RESPIRATORY CHAIN COMPLEX I EARLY INTERMEDIATE NDUFAF1 ASSEMBLY MITOCHONDRIAL |  |
| NCI FORMATION OF HIV 1 ELONGATION COMPLEX CONTAINING HIV 1 TAT |  |
| STRUCTURAL CONSTITUENT OF RIBOSOME |  |
| REACTOME REGULATION OF APC ACTIVATORS BETWEEN G1 S AND EARLY ANAPHASE |  |
| REACTOME CDC20 PHOSPHO APC MEDIATED DEGRADATION OF CYCLIN A |  |
| REACTOME VIF MEDIATED DEGRADATION OF APOBEC3G |  |
| REACTOME GENE EXPRESSION |  |
| NCI FORMATION OF RNA POL II ELONGATION COMPLEX |  |
| KEGG PARKINSONS DISEASE |  |
| REACTOME PEPTIDE CHAIN ELONGATION |  |
| NCI ELONGATION OF INTRON CONTAINING TRANSCRIPTS AND CO TRANSCRIPTIONAL MRNA SPLICING |  |
| CORUM RESPIRATORY CHAIN COMPLEX I BETA SUBUNIT MITOCHONDRIAL |  |
| REACTOME MITOCHONDRIAL FATTY ACID BETA OXIDATION |  |
| MITOCHONDRIAL MATRIX |  |
| CORUM BRCA1-CORE RNA POLYMERASE II COMPLEX |  |
| NCI RNA POLYMERASE II PROMOTER ESCAPE | POLR2H, TAF12, POLR2F, GTF2H4, POLR2I, GTF2A2, POLR2G |
| NCI ABORTIVE ELONGATION OF HIV 1 TRANSCRIPT IN THE ABSENCE OF TAT |  |
| SMALL RIBOSOMAL SUBUNIT |  |
| REACTOME SIGNALING BY WNT |  |
| REACTOME ORC1 REMOVAL FROM CHROMATIN |  |
| CORUM RNA POLYMERASE II HOLOENZYME COMPLEX | POLR2H, POLR2F, GTF2H4, POLR2I, POLR2G |
| REACTOME DUAL INCISION REACTION IN TC NER | POLR2H, XAB2, POLR2F, ERCC5, POLR2I, POLR2G |
| CORUM 28S RIBOSOMAL SUBUNIT MITOCHONDRIAL | MRPS34, MRPS7, MRPS12, MRPS16, MRPS11, MRPS33, MRPS28, MRPS14, MRPS15 |
| REACTOME INFLUENZA VIRAL RNA TRANSCRIPTION AND REPLICATION |  |
| NCI FORMATION AND MATURATION OF MRNA TRANSCRIPT | POLR2H, TAF12, POLR2F, GTF2H4, POLR2I, GTF2A2, POLR2G |
| NCI RNA POLYMERASE II TRANSCRIPTION INITIATION AND PROMOTER CLEARANCE | POLR2H, TAF12, POLR2F, GTF2H4, POLR2I, GTF2A2, POLR2G |
| NCI HIV 1 TRANSCRIPTION PRE INITIATION | POLR2H, TAF12, POLR2F, GTF2H4, POLR2I, GTF2A2, POLR2G |
| NCI FORMATION OF ATP BY CHEMIOSMOTIC COUPLING |  |
| REACTOME FORMATION OF A POOL OF FREE 40S SUBUNITS |  |
| NCI REGULATION OF ACTIVATED PAK 2P34 BY PROTEASOME MEDIATED DEGRADATION |  |
| ORGANELLE MEMBRANE |  |
| NCI UBIQUITIN MEDIATED DEGRADATION OF PHOSPHORYLATED CDC25A |  |
| NCI TRANSCRIPTION OF THE HIV GENOME | POLR2H, TAF12, POLR2F, GTF2H4, POLR2I, GTF2A2, POLR2G |
| POSITIVE REGULATION OF PROTEIN SECRETION |  |
| DETECTION OF BIOTIC STIMULUS |  |
| HYDROGEN ION TRANSMEMBRANE TRANSPORTER ACTIVITY |  |

| Color legend | | | | | | | | | | | |
| --- | --- | --- | --- | --- | --- | --- | --- | --- | --- | --- | --- |
| q-value | -1 | -0.2 | -0.05 | -0.01 | -0.001 | -0.0001 |
| Color |  |  |  |  |  |  |

TABLE OF Q-VALUES

| aspergillus fumigatus conidia a549 | candida albicans moddc135 | Gene Set |
| --- | --- | --- |
| -0.0 | -0.0042326762 | REACTOME\_ELECTRON\_TRANSPORT\_CHAIN |
| -0.09963688 | -0.01787396 | ORGANELLE\_ENVELOPE |
| -0.0012278926 | -0.0029108513 | MITOCHONDRIAL\_RESPIRATORY\_CHAIN |
| -0.10145514 | -0.017450536 | ENVELOPE |
| -0.1488785 | -0.016759641 | CORUM\_RESPIRATORY\_CHAIN\_COMPLEX\_I\_INCOMPLETE\_INTERMEDIATE\_MITOCHONDRIAL |
| -0.0041144835 | -0.00528366 | CORUM\_RESPIRATORY\_CHAIN\_COMPLEX\_I\_LAMBDA\_SUBUNIT\_MITOCHONDRIAL |
| -0.14321724 | -0.011471048 | AEROBIC\_RESPIRATION |
| -0.05383398 | -0.0106720785 | NCI\_ORC1\_REMOVAL\_FROM\_CHROMATIN |
| -0.032371715 | -0.011758469 | REACTOME\_CDT1\_ASSOCIATION\_WITH\_THE\_CDC6\_ORC\_ORIGIN\_COMPLEX |
| -0.0048236074 | -0.015324515 | NCI\_APC\_C\_CDH1\_MEDIATED\_DEGRADATION\_OF\_CDC20\_AND\_OTHER\_APC\_C\_CDH1\_TARGETED\_PROTEINS\_IN\_LATE\_MITOSIS\_EARLY\_G1 |
| -0.0055047623 | -0.015378352 | NCI\_AUTODEGRADATION\_OF\_CDH1\_BY\_CDH1\_APC\_C |
| -0.024426209 | -0.011714172 | NCI\_CDT1\_ASSOCIATION\_WITH\_THE\_CDC6\_ORC\_ORIGIN\_COMPLEX |
| -0.06528577 | -0.0011531919 | NCI\_REMOVAL\_OF\_LICENSING\_FACTORS\_FROM\_ORIGINS |
| -0.03827526 | -0.008206246 | NCI\_CDC20\_PHOSPHO\_APC\_C\_MEDIATED\_DEGRADATION\_OF\_CYCLIN\_A |
| -0.006735072 | -0.01617904 | REACTOME\_AUTODEGRADATION\_OF\_CDH1\_BY\_CDH1\_APC |
| -0.1432032 | -0.001409413 | REACTOME\_M\_G1\_TRANSITION |
| -0.004438449 | -0.010109279 | NCI\_SWITCHING\_OF\_ORIGINS\_TO\_A\_POST\_REPLICATIVE\_STATE |
| -0.050533775 | -0.015392404 | REACTOME\_MRNA\_DECAY\_BY\_3\_TO\_5\_EXORIBONUCLEASE |
| -0.1088028 | -0.0031472717 | RIBONUCLEOPROTEIN\_COMPLEX |
| -0.18917029 | -0.0026966596 | ESTABLISHMENT\_OF\_VESICLE\_LOCALIZATION |
| -0.06994355 | -0.060189236 | ORGANELLAR\_SMALL\_RIBOSOMAL\_SUBUNIT |
| -0.17951778 | -0.008146862 | VESICLE\_LOCALIZATION |
| -0.17016739 | -0.0018440568 | REACTOME\_RNA\_POL\_II\_CTD\_PHOSPHORYLATION\_AND\_INTERACTION\_WITH\_CE |
| -0.13291328 | -0.01980183 | PEROXISOME |
| -0.1823957 | -0.0030244656 | NCI\_RNA\_POLYMERASE\_II\_TRANSCRIPTION\_PRE\_INITIATION |
| -0.10654625 | -0.0 | REACTOME\_NUCLEOTIDE\_EXCISION\_REPAIR |
| -0.0024710582 | -0.037789445 | CORUM\_F1F0-ATP\_SYNTHASE\_EC\_3.6.3.14\_MITOCHONDRIAL |
| -0.0016155674 | -0.078787155 | NCI\_HOST\_INTERACTIONS\_OF\_HIV\_FACTORS |
| -0.0 | -0.08464909 | REACTOME\_GLUCOSE\_REGULATION\_OF\_INSULIN\_SECRETION |
| -0.068763465 | -0.054278184 | CORUM\_RNA\_POLYMERASE\_II\_RNAPII |
| -0.08501885 | -0.0021060598 | KEGG\_RNA\_POLYMERASE |
| -0.043655038 | -0.0029766958 | NCI\_FORMATION\_OF\_THE\_EARLY\_ELONGATION\_COMPLEX |
| -0.013872726 | -0.06707726 | REACTOME\_SCF\_BETA\_TRCP\_MEDIATED\_DEGRADATION\_OF\_EMI1 |
| -0.120242715 | -0.037391946 | NCI\_RNA\_POLYMERASE\_II\_TRANSCRIPTION\_ELONGATION |
| -0.058844544 | -0.16770418 | OXIDOREDUCTASE\_ACTIVITY\_ACTING\_ON\_THE\_CH\_CH\_GROUP\_OF\_DONORS |
| -0.012355938 | -0.18792583 | OXIDOREDUCTASE\_ACTIVITY\_ACTING\_ON\_NADH\_OR\_NADPH |
| -0.08102552 | -0.059933983 | MITOCHONDRIAL\_SMALL\_RIBOSOMAL\_SUBUNIT |
| -0.13949311 | -0.1681935 | FATTY\_ACID\_METABOLIC\_PROCESS |
| -0.118870884 | -0.036283128 | CORUM\_EXOSOME |
| -0.003946924 | -0.09194465 | NCI\_VPU\_MEDIATED\_DEGRADATION\_OF\_CD4 |
| -0.1513847 | -0.019761499 | RNA\_POLYMERASE\_ACTIVITY |
| -0.017353417 | -0.022583239 | KEGG\_PEROXISOME |
| -0.025246581 | -0.019329578 | CORUM\_RESPIRATORY\_CHAIN\_COMPLEX\_I\_INTERMEDIATE\_VII/650KD\_MITOCHONDRIAL |
| -0.010294335 | -0.04456721 | RIBOSOMAL\_SUBUNIT |
| -0.19928153 | -0.039459545 | REACTOME\_TELOMERE\_MAINTENANCE |
| -0.083885916 | -0.017430348 | CORUM\_LSM1-7\_COMPLEX |
| -0.0 | -0.08093818 | KEGG\_OXIDATIVE\_PHOSPHORYLATION |
| -0.0 | -0.09010949 | NCI\_VIRAL\_MRNA\_TRANSLATION |
| -0.10660171 | -0.06740698 | CELLULAR\_RESPIRATION |
| -0.046077378 | -0.062596954 | CORUM\_RNA\_POLYMERASE\_II\_CORE\_COMPLEX |
| -0.0 | -0.09003957 | REACTOME\_VIRAL\_MRNA\_TRANSLATION |
| -0.037430074 | -0.1584127 | REACTOME\_MICRORNA\_BIOGENESIS |
| -0.18300086 | -0.0043030553 | REACTOME\_HIV1\_TRANSCRIPTION\_INITIATION |
| -0.004877203 | -0.0883461 | REACTOME\_P53\_INDEPENDENT\_DNA\_DAMAGE\_RESPONSE |
| -0.0 | -0.10015844 | NCI\_PEPTIDE\_CHAIN\_ELONGATION |
| -0.0021898218 | -0.043524224 | NADH\_DEHYDROGENASE\_COMPLEX |
| -0.18270484 | -0.0030064138 | NCI\_RNA\_POLYMERASE\_II\_TRANSCRIPTION\_INITIATION |
| -0.18564335 | -0.0030264389 | NCI\_RNA\_POLYMERASE\_II\_HIV\_1\_PROMOTER\_ESCAPE |
| -0.0 | -0.08162497 | KEGG\_RIBOSOME |
| -0.038492735 | -0.04465357 | NCI\_MRNA\_SPLICING\_\_\_MAJOR\_PATHWAY |
| -0.120618254 | -0.026729085 | REACTOME\_HIV1\_TRANSCRIPTION\_ELONGATION |
| -0.0041450756 | -0.09627981 | NCI\_SCF\_BETA\_TRCP\_MEDIATED\_DEGRADATION\_OF\_EMI1 |
| -0.0061084223 | -0.06351998 | NCI\_REGULATION\_OF\_ACTIVATED\_PAK\_2P34\_BY\_PROTEASOME\_MEDIATED\_DEGRADATION1 |
| -0.0047522634 | -0.15251054 | KEGG\_PROTEASOME |
| -0.0014645411 | -0.14592344 | NCI\_VIF\_MEDIATED\_DEGRADATION\_OF\_APOBEC3G |
| -0.04684002 | -0.019852318 | CORUM\_LSM2-8\_COMPLEX |
| -0.16962957 | -0.009274207 | REACTOME\_FORMATION\_AND\_MATURATION\_OF\_MRNA\_TRANSCRIPT |
| -0.14983578 | -0.10229519 | POSITIVE\_REGULATION\_OF\_CYTOKINE\_SECRETION |
| -0.18829085 | -0.0031642471 | NCI\_HIV\_1\_TRANSCRIPTION\_INITIATION |
| -0.15599461 | -0.0013294893 | NCI\_RNA\_POL\_II\_CTD\_PHOSPHORYLATION\_AND\_INTERACTION\_WITH\_CE |
| -0.14895299 | -0.0013565404 | NCI\_RNA\_POL\_II\_CTD\_PHOSPHORYLATION\_AND\_INTERACTION\_WITH\_CE1 |
| -0.103672884 | -0.054964002 | NCI\_TAT\_MEDIATED\_ELONGATION\_OF\_THE\_HIV\_1\_TRANSCRIPT |
| -0.0 | -0.11395516 | NCI\_FORMATION\_OF\_A\_POOL\_OF\_FREE\_40S\_SUBUNITS |
| -0.14333291 | -0.018453468 | MICROBODY |
| -0.18918294 | -0.0018487591 | CORUM\_BRCA1-RNA\_POLYMERASE\_II\_COMPLEX |
| -0.023509752 | -0.023703527 | REACTOME\_ABORTIVE\_ELONGATION\_OF\_HIV1\_TRANSCRIPT\_IN\_THE\_ABSENCE\_OF\_TAT |
| -0.006375424 | -0.07335171 | MITOCHONDRIAL\_LUMEN |
| -0.032766946 | -0.027123883 | REACTOME\_MRNA\_SPLICING |
| -0.03677051 | -0.0035150482 | NCI\_FORMATION\_OF\_THE\_HIV\_1\_EARLY\_ELONGATION\_COMPLEX |
| -0.012765669 | -0.026127843 | NCI\_APC\_C\_CDC20\_MEDIATED\_DEGRADATION\_OF\_SECURIN |
| -0.16820036 | -0.08537631 | CYTOKINE\_SECRETION |
| -0.08497858 | -0.013949721 | REACTOME\_ELONGATION\_AND\_PROCESSING\_OF\_CAPPED\_TRANSCRIPTS |
| -0.0016940758 | -0.029169844 | MITOCHONDRIAL\_RIBOSOME |
| -0.0023094467 | -0.03968679 | RESPIRATORY\_CHAIN\_COMPLEX\_I |
| -0.18876565 | -0.0033233832 | NCI\_RNA\_POLYMERASE\_II\_TRANSCRIPTION |
| -0.0021812555 | -0.037207037 | MITOCHONDRIAL\_RESPIRATORY\_CHAIN\_COMPLEX\_I |
| -0.0048473706 | -0.05915291 | REACTOME\_FORMATION\_OF\_ATP\_BY\_CHEMIOSMOTIC\_COUPLING |
| -0.15030144 | -0.1300732 | KEGG\_BUTANOATE\_METABOLISM |
| -0.06037003 | -0.008173908 | KEGG\_PYRIMIDINE\_METABOLISM |
| -0.020137079 | -0.04817951 | REACTOME\_VIRAL\_MESSENGER\_RNA\_SYNTHESIS |
| -0.0407774 | -0.0029624666 | NCI\_HIV\_1\_TRANSCRIPTION\_ELONGATION |
| -0.0016211164 | -0.030870223 | ORGANELLAR\_RIBOSOME |
| -0.13797833 | -0.12778845 | REGULATION\_OF\_CYTOKINE\_SECRETION |
| -0.051903196 | -0.0021079895 | REACTOME\_FORMATION\_OF\_THE\_EARLY\_ELONGATION\_COMPLEX |
| -0.14548959 | -0.09468565 | KEGG\_PORPHYRIN\_AND\_CHLOROPHYLL\_METABOLISM |
| -0.0 | -0.19491725 | NCI\_L13A\_MEDIATED\_TRANSLATIONAL\_SILENCING\_OF\_CERULOPLASMIN\_EXPRESSION |
| -0.17341332 | -0.11692676 | REACTOME\_P2Y\_RECEPTORS |
| -0.089656524 | -0.13078342 | CORUM\_RESPIRATORY\_CHAIN\_COMPLEX\_I\_EARLY\_INTERMEDIATE\_NDUFAF1\_ASSEMBLY\_MITOCHONDRIAL |
| -0.11396314 | -0.033146307 | NCI\_FORMATION\_OF\_HIV\_1\_ELONGATION\_COMPLEX\_CONTAINING\_HIV\_1\_TAT |
| -0.0 | -0.12606873 | STRUCTURAL\_CONSTITUENT\_OF\_RIBOSOME |
| -0.111958295 | -0.114191785 | REACTOME\_REGULATION\_OF\_APC\_ACTIVATORS\_BETWEEN\_G1\_S\_AND\_EARLY\_ANAPHASE |
| -0.029512709 | -0.039681815 | REACTOME\_CDC20\_PHOSPHO\_APC\_MEDIATED\_DEGRADATION\_OF\_CYCLIN\_A |
| -0.0013596858 | -0.14123815 | REACTOME\_VIF\_MEDIATED\_DEGRADATION\_OF\_APOBEC3G |
| -0.09432287 | -0.04762433 | REACTOME\_GENE\_EXPRESSION |
| -0.13729998 | -0.033875223 | NCI\_FORMATION\_OF\_RNA\_POL\_II\_ELONGATION\_COMPLEX\_ |
| -0.0 | -0.01866694 | KEGG\_PARKINSONS\_DISEASE |
| -0.0 | -0.10028489 | REACTOME\_PEPTIDE\_CHAIN\_ELONGATION |
| -0.14888924 | -0.03086242 | NCI\_ELONGATION\_OF\_INTRON\_CONTAINING\_TRANSCRIPTS\_AND\_CO\_TRANSCRIPTIONAL\_MRNA\_SPLICING |
| -0.0036567838 | -0.17501329 | CORUM\_RESPIRATORY\_CHAIN\_COMPLEX\_I\_BETA\_SUBUNIT\_MITOCHONDRIAL |
| -0.1875582 | -0.07101309 | REACTOME\_MITOCHONDRIAL\_FATTY\_ACID\_BETA\_OXIDATION |
| -0.0063135265 | -0.07889756 | MITOCHONDRIAL\_MATRIX |
| -0.053434946 | -0.030560207 | CORUM\_BRCA1-CORE\_RNA\_POLYMERASE\_II\_COMPLEX |
| -0.19105646 | -0.0027085582 | NCI\_RNA\_POLYMERASE\_II\_PROMOTER\_ESCAPE |
| -0.020793166 | -0.056737807 | NCI\_ABORTIVE\_ELONGATION\_OF\_HIV\_1\_TRANSCRIPT\_IN\_THE\_ABSENCE\_OF\_TAT |
| -0.06787975 | -0.057359003 | SMALL\_RIBOSOMAL\_SUBUNIT |
| -0.16954629 | -0.19680882 | REACTOME\_SIGNALING\_BY\_WNT |
| -0.09401642 | -0.0369318 | REACTOME\_ORC1\_REMOVAL\_FROM\_CHROMATIN |
| -0.17117524 | -0.004446161 | CORUM\_RNA\_POLYMERASE\_II\_HOLOENZYME\_COMPLEX |
| -0.028032146 | -0.0018773903 | REACTOME\_DUAL\_INCISION\_REACTION\_IN\_TC\_NER |
| -0.0034792167 | -0.0030206852 | CORUM\_28S\_RIBOSOMAL\_SUBUNIT\_MITOCHONDRIAL |
| -0.0 | -0.033735447 | REACTOME\_INFLUENZA\_VIRAL\_RNA\_TRANSCRIPTION\_AND\_REPLICATION |
| -0.18883672 | -0.0027118528 | NCI\_FORMATION\_AND\_MATURATION\_OF\_MRNA\_TRANSCRIPT |
| -0.1795855 | -0.003271132 | NCI\_RNA\_POLYMERASE\_II\_TRANSCRIPTION\_INITIATION\_AND\_PROMOTER\_CLEARANCE |
| -0.18625917 | -0.0029841603 | NCI\_HIV\_1\_TRANSCRIPTION\_PRE\_INITIATION |
| -0.0046969065 | -0.05519277 | NCI\_FORMATION\_OF\_ATP\_BY\_CHEMIOSMOTIC\_COUPLING |
| -0.0 | -0.11682938 | REACTOME\_FORMATION\_OF\_A\_POOL\_OF\_FREE\_40S\_SUBUNITS |
| -0.0021843927 | -0.07666453 | NCI\_REGULATION\_OF\_ACTIVATED\_PAK\_2P34\_BY\_PROTEASOME\_MEDIATED\_DEGRADATION |
| -0.18811844 | -0.033378232 | ORGANELLE\_MEMBRANE |
| -0.004132616 | -0.08640822 | NCI\_UBIQUITIN\_MEDIATED\_DEGRADATION\_OF\_PHOSPHORYLATED\_CDC25A |
| -0.18213114 | -0.0031621137 | NCI\_TRANSCRIPTION\_OF\_THE\_HIV\_GENOME |
| -0.09398284 | -0.056834225 | POSITIVE\_REGULATION\_OF\_PROTEIN\_SECRETION |
| -0.11390469 | -0.16771029 | DETECTION\_OF\_BIOTIC\_STIMULUS |
| -0.012319426 | -0.1616828 | HYDROGEN\_ION\_TRANSMEMBRANE\_TRANSPORTER\_ACTIVITY |
