# Supplementary material for: Computational approaches for discovery of common immunomodulators in fungal infections: towards broad-spectrum immunotherapeutic interventions
Source: BMC Microbiol. 2013 Oct 7;13:224. doi: 10.1186/1471-2180-13-224 (PMC3853472; doi:10.1186/1471-2180-13-224)
Supplement: Additional file 1 — Details of up- and down- regulated biclusters. [file 1471-2180-13-224-S1.zip › 2013-kidane-bmc/details-of-biclusters/dnreg-biclust-12.html]

**BICLUSTER\_ID** : DNREG-12  
**PATHOGENS** /2/ : a. fumigatus,c. albicans  
**KNOWN DRUG TARGETS** /0/ :   

| Gene Set | Leading Edge Genes |
| --- | --- |
| NCI SYNTHESIS OF BILE ACIDS AND BILE SALTS VIA 24 HYDROXYCHOLESTEROL | AMACR |
| NCI SYNTHESIS OF BILE ACIDS AND BILE SALTS | AMACR |
| NCI PEPTIDE LIGAND BINDING RECEPTORS |  |
| REACTOME P2Y RECEPTORS | P2RY10 |
| REACTOME STEROID HORMONES | LGMN |

| Color legend | | | | | | | | | | | |
| --- | --- | --- | --- | --- | --- | --- | --- | --- | --- | --- | --- |
| q-value | -1 | -0.2 | -0.05 | -0.01 | -0.001 | -0.0001 |
| Color |  |  |  |  |  |  |

TABLE OF Q-VALUES

| candida albicans huvec | aspergillus fumigatus conidia a549 | Gene Set |
| --- | --- | --- |
| -0.13695282 | -0.024139855 | NCI\_SYNTHESIS\_OF\_BILE\_ACIDS\_AND\_BILE\_SALTS\_VIA\_24\_HYDROXYCHOLESTEROL |
| -0.16183066 | -0.038341194 | NCI\_SYNTHESIS\_OF\_BILE\_ACIDS\_AND\_BILE\_SALTS |
| -0.07648555 | -0.09421941 | NCI\_PEPTIDE\_LIGAND\_BINDING\_RECEPTORS |
| -0.04069343 | -0.17341332 | REACTOME\_P2Y\_RECEPTORS |
| -0.007715277 | -0.18254085 | REACTOME\_STEROID\_HORMONES |
