# Supplementary material for: Computational approaches for discovery of common immunomodulators in fungal infections: towards broad-spectrum immunotherapeutic interventions
Source: BMC Microbiol. 2013 Oct 7;13:224. doi: 10.1186/1471-2180-13-224 (PMC3853472; doi:10.1186/1471-2180-13-224)
Supplement: Additional file 1 — Details of up- and down- regulated biclusters. [file 1471-2180-13-224-S1.zip › 2013-kidane-bmc/details-of-biclusters/dnreg-biclust-14.html]

**BICLUSTER\_ID** : DNREG-14  
**PATHOGENS** /2/ : a. fumigatus,s. chartarum  
**KNOWN DRUG TARGETS** /4/ : GSTA4, GSTK1, MGST3, MAOA  

| Gene Set | Leading Edge Genes |
| --- | --- |
| KEGG DRUG METABOLISM CYTOCHROME P450 | GSTA4, GSTK1, MGST3, MAOA |
| NCI HORMONE LIGAND BINDING RECEPTORS | TSHB |
| REACTOME HORMONE LIGAND BINDING RECEPTORS | TSHB |

| Color legend | | | | | | | | | | | |
| --- | --- | --- | --- | --- | --- | --- | --- | --- | --- | --- | --- |
| q-value | -1 | -0.2 | -0.05 | -0.01 | -0.001 | -0.0001 |
| Color |  |  |  |  |  |  |

TABLE OF Q-VALUES

| aspergillus fumigatus conidia a549 | aspergillus fumigatus cluture filtrates a549 | stachybotrys chartarum lung | Gene Set |
| --- | --- | --- | --- |
| -0.0016370183 | -0.0155476285 | -0.043982726 | KEGG\_DRUG\_METABOLISM\_CYTOCHROME\_P450 |
| -0.01072094 | -0.0492003 | -0.10186097 | NCI\_HORMONE\_LIGAND\_BINDING\_RECEPTORS |
| -0.0041764076 | -0.024800204 | -0.06603967 | REACTOME\_HORMONE\_LIGAND\_BINDING\_RECEPTORS |
