# Supplementary material for: Computational approaches for discovery of common immunomodulators in fungal infections: towards broad-spectrum immunotherapeutic interventions
Source: BMC Microbiol. 2013 Oct 7;13:224. doi: 10.1186/1471-2180-13-224 (PMC3853472; doi:10.1186/1471-2180-13-224)
Supplement: Additional file 1 — Details of up- and down- regulated biclusters. [file 1471-2180-13-224-S1.zip › 2013-kidane-bmc/details-of-biclusters/dnreg-biclust-20.html]

**BICLUSTER\_ID** : DNREG-20  
**PATHOGENS** /2/ : c. albicans,a. fumigatus  
**KNOWN DRUG TARGETS** /0/ :   

| Gene Set | Leading Edge Genes |
| --- | --- |
| NEUROPEPTIDE BINDING |  |
| NEUROPEPTIDE RECEPTOR ACTIVITY |  |

| Color legend | | | | | | | | | | | |
| --- | --- | --- | --- | --- | --- | --- | --- | --- | --- | --- | --- |
| q-value | -1 | -0.2 | -0.05 | -0.01 | -0.001 | -0.0001 |
| Color |  |  |  |  |  |  |

TABLE OF Q-VALUES

| aspergillus fumigatus conidia a549 | candida albicans neutrophils | aspergillus fumigatus 16hbe14o | Gene Set |
| --- | --- | --- | --- |
| -0.14514485 | -0.029743437 | -0.14210184 | NEUROPEPTIDE\_BINDING |
| -0.13338187 | -0.025046814 | -0.1392568 | NEUROPEPTIDE\_RECEPTOR\_ACTIVITY |
