# Supplementary material for: Computational approaches for discovery of common immunomodulators in fungal infections: towards broad-spectrum immunotherapeutic interventions
Source: BMC Microbiol. 2013 Oct 7;13:224. doi: 10.1186/1471-2180-13-224 (PMC3853472; doi:10.1186/1471-2180-13-224)
Supplement: Additional file 1 — Details of up- and down- regulated biclusters. [file 1471-2180-13-224-S1.zip › 2013-kidane-bmc/details-of-biclusters/dnreg-biclust-2.html]

**BICLUSTER\_ID** : DNREG-2  
**PATHOGENS** /1/ : a. fumigatus  
**KNOWN DRUG TARGETS** /75/ : LRP2, GSTO1, GSTA4, FGA, NDUFA3, MGST3, PECR, GPX4, DHRS3, CYP2D6, ADH5, NDUFB7, NDUFS6, FGG, F8, UGDH, DHRS4, DECR1, NDUFS5, NDUFA13, NDUFS7, GPX2, GSTA1, ALDH7A1, HSD17B6, PLA2G6, EHHADH, NQO2, DCI, CYB5A, FTH1, MTHFR, NDUFS4, HMOX1, CFD, ALDH1B1, HADH, IDH1, BAAT, NDUFB6, NDUFA4, EGLN2, SDHD, HSD17B4, EPHX2, NDUFB8, MGST2, BLVRB, BDH1, GSTM4, NDUFB5, BLVRA, NDUFA7, RDH5, NDUFA6, UROD, UGT2B28, SULT1A1, GSTK1, PLA2G2A, AKR1C4, CYP2R1, MSRB2, PHYH, SULT2B1, MAOA, NDUFC2, PRDX2, NDUFB3, PYCR1, GSTZ1, ALDH2, HMGCL, F5, FGB  

| Gene Set | Leading Edge Genes |
| --- | --- |
| OXIDOREDUCTASE ACTIVITY | CYP1A2, CYP4F3, MGST3, GPX4, KMO, BLVRB, BDH1, BLVRA, NOX5, RDH5, MECR, SURF1, DECR1, COX5A, GPX2, L2HGDH, ALDH7A1, CYBA, AKR1C4, HSD17B6, MSRB2, BDH2, ADH6, EHHADH, NQO2, PRDX2, CYB5A, ETFB, HAO1, PYCR1, MTHFR, NOX1, GSTZ1, NDUFS4, HMOX1, HADH, AKR1A1, FDXR, EGLN2, SDHD |
| REACTOME ELECTRON TRANSPORT CHAIN | NDUFS5, NDUFA13, NDUFS7, NDUFA3, NDUFB8, COX5B, NDUFB5, NDUFA7, NDUFC2, NDUFB7, NDUFA6, ETFB, NDUFB3, NDUFS6, NDUFS4, COX4I1, UQCRQ, NDUFB6, NDUFA4, SDHD, NDUFB11 |
| KEGG DRUG METABOLISM CYTOCHROME P450 | CYP1A2, GSTA4, GSTO1, UGT2A1, GSTZ1, GSTK1, GSTA1, MGST3, MGST2, UGT2B15, ADH6, CYP2D6, GSTM4, UGT2B28, MAOA |
| KEGG GLUTATHIONE METABOLISM | GSTA4, GSTO1, GPX2, GSTK1, GSTZ1, GSTA1, MGST3, GPX4, MGST2, GSTM4 |
| KEGG METABOLISM OF XENOBIOTICS BY CYTOCHROME P450 | CYP1A2, GSTA4, GSTO1, UGT2A1, GSTZ1, GSTK1, GSTA1, MGST3, AKR1C4, MGST2, UGT2B15, EPHX1, ADH6, GSTM4, UGT2B28 |
| KEGG PEROXISOME | PXMP2, EPHX2, DECR2, PEX10, PEX7, PECR, PHYH, GNPAT, EHHADH, PEX16, MVK, HAO1, PEX11A, AMACR, BAAT, HMGCL, PXMP4, DHRS4, PEX12, IDH2, ECH1, HSD17B4, ABCD4 |
| OXIDOREDUCTASE ACTIVITY GO 0016616 | GRHPR, HADH, AKR1A1, UGDH, IDH1, AKR1C4, BDH1, EHHADH, ADH6, BDH2, HSD17B4, RDH5 |
| OXIDOREDUCTASE ACTIVITY ACTING ON CH OH GROUP OF DONORS | HAO1, GRHPR, HADH, AKR1A1, UGDH, L2HGDH, IDH1, AKR1C4, BDH1, EHHADH, ADH6, BDH2, HSD17B4, RDH5 |
| KEGG FATTY ACID METABOLISM | ALDH1B1, HADH, ALDH7A1, ALDH2, EHHADH, ADH6, ACAA2, DCI, ADH5 |
| KEGG BUTANOATE METABOLISM | ACSM3, ALDH1B1, ALDH7A1, HADH, HMGCS2, L2HGDH, ALDH2, HMGCL, BDH1, EHHADH, BDH2 |
| NCI EXOCYTOSIS OF ALPHA GRANULE | FGA, FGG, F5, F8, FGB, CFD |
| KEGG PORPHYRIN AND CHLOROPHYLL METABOLISM | PPOX, HMOX1, UGT2A1, BLVRB, UGT2B15, UROD, UROS, BLVRA, UGT2B28, FTH1 |
| REACTOME GLUTATHIONE CONJUGATION | GSTA4, GSTO1, GSTM4, GSTA1, MGST3, MGST2 |
| KEGG ASCORBATE AND ALDARATE METABOLISM | UGT2A1, ALDH1B1, UGT2B28, ALDH7A1, UGDH, ALDH2, UGT2B15 |
| GLUTATHIONE TRANSFERASE ACTIVITY | GSTA4, GSTM4, GSTZ1, MGST3, MGST2 |
| KEGG RETINOL METABOLISM | PNPLA4, CYP1A2, UGT2A1, BCMO1, DHRS3, UGT2B15, DHRS4, ADH6, UGT2B28, ADH5, RDH5 |
| REACTOME FORMATION OF FIBRIN CLOT CLOTTING CASCADE | F13B, FGA, FGG, F5, F8, FGB |
| KEGG PRIMARY BILE ACID BIOSYNTHESIS | AMACR, AKR1C4, BAAT |
| REACTOME SYNTHESIS OF BILE ACIDS AND BILE SALTS | AMACR, AKR1C4, BAAT |
| REACTOME CYTOSOLIC SULFONATION OF SMALL MOLECULES | SULT2B1, SULT1A1, SULT1A2, SULT1A3 |
| PHOSPHOLIPASE A2 ACTIVITY | PRDX6, PLA2G2A, PLA2G5, PLA2G6 |
| REACTOME STEROID HORMONES | LRP2, CYP24A1, LGMN, CYP2R1 |
| XENOBIOTIC METABOLIC PROCESS | UGT2B28, UGT2B15 |
| NCI SYNTHESIS OF BILE ACIDS AND BILE SALTS VIA 24 HYDROXYCHOLESTEROL | SLC27A2, SLC27A5, AMACR, AKR1C4 |
| NCI SYNTHESIS OF BILE ACIDS AND BILE SALTS | SLC27A2, SLC27A5, AMACR, AKR1C4 |
| REACTOME SYNTHESIS OF BILE ACIDS AND BILE SALTS VIA 24 HYDROXYCHOLESTEROL | SLC27A2, SLC27A5, AMACR, AKR1C4 |
| REACTOME HORMONE LIGAND BINDING RECEPTORS | TSHB |
| DETECTION OF CHEMICAL STIMULUS | STIM1, UGT2A1, SYT1 |
| REACTOME PEPTIDE CHAIN ELONGATION | RPS15A, RPS18, RPS4Y1, RPL38, RPS17, RPS14, RPS21, RPS5, RPS16, RPS28, RPL26L1, RPL22, RPS9, RPL12, RPL13, RPS12, RPL30, UBA52, RPS13, RPL19, RPS29, RPL28, RPL31, RPL13A, RPL29, RPS27, RPS20, RPL10A, RPS27A |
| TRIACYLGLYCEROL METABOLIC PROCESS | PNLIPRP2 |
| KEGG SYSTEMIC LUPUS ERYTHEMATOSUS | HIST3H2A, HIST1H3D, HLA-DQB1, HIST1H2BD, HIST1H3H, C7, H2AFZ, HIST1H2BH, H2AFV, HLA-DMB, HIST1H2AG, HIST1H2AC |
| SENSORY PERCEPTION OF TASTE | TAS2R14 |
| NCI HORMONE LIGAND BINDING RECEPTORS | TSHB |
| KEGG AUTOIMMUNE THYROID DISEASE | HLA-DOA, HLA-F, HLA-DQB1, HLA-DMB, HLA-C, HLA-G |

| Color legend | | | | | | | | | | | |
| --- | --- | --- | --- | --- | --- | --- | --- | --- | --- | --- | --- |
| q-value | -1 | -0.2 | -0.05 | -0.01 | -0.001 | -0.0001 |
| Color |  |  |  |  |  |  |

TABLE OF Q-VALUES

| aspergillus fumigatus conidia a549 | aspergillus fumigatus cluture filtrates a549 | Gene Set |
| --- | --- | --- |
| -0.0064010406 | -0.13529885 | OXIDOREDUCTASE\_ACTIVITY |
| -0.0 | -0.14915366 | REACTOME\_ELECTRON\_TRANSPORT\_CHAIN |
| -0.0016370183 | -0.0155476285 | KEGG\_DRUG\_METABOLISM\_CYTOCHROME\_P450 |
| -0.0036571922 | -0.15647686 | KEGG\_GLUTATHIONE\_METABOLISM |
| -0.0016399521 | -0.109704904 | KEGG\_METABOLISM\_OF\_XENOBIOTICS\_BY\_CYTOCHROME\_P450 |
| -0.017353417 | -0.07615189 | KEGG\_PEROXISOME |
| -0.12517942 | -0.18114749 | OXIDOREDUCTASE\_ACTIVITY\_GO\_0016616 |
| -0.16851543 | -0.1593284 | OXIDOREDUCTASE\_ACTIVITY\_ACTING\_ON\_CH\_OH\_GROUP\_OF\_DONORS |
| -0.18709704 | -0.016400568 | KEGG\_FATTY\_ACID\_METABOLISM |
| -0.15030144 | -0.007829288 | KEGG\_BUTANOATE\_METABOLISM |
| -0.09314135 | -0.046258703 | NCI\_EXOCYTOSIS\_OF\_ALPHA\_GRANULE\_ |
| -0.14548959 | -0.17071082 | KEGG\_PORPHYRIN\_AND\_CHLOROPHYLL\_METABOLISM |
| -0.01229048 | -0.11134606 | REACTOME\_GLUTATHIONE\_CONJUGATION |
| -0.1862089 | -0.04190696 | KEGG\_ASCORBATE\_AND\_ALDARATE\_METABOLISM |
| -0.0048513873 | -0.091617465 | GLUTATHIONE\_TRANSFERASE\_ACTIVITY |
| -0.11428769 | -0.10407206 | KEGG\_RETINOL\_METABOLISM |
| -0.19206469 | -0.048555158 | REACTOME\_FORMATION\_OF\_FIBRIN\_CLOT\_CLOTTING\_CASCADE |
| -0.009655746 | -0.15741973 | KEGG\_PRIMARY\_BILE\_ACID\_BIOSYNTHESIS |
| -0.004840797 | -0.14989902 | REACTOME\_SYNTHESIS\_OF\_BILE\_ACIDS\_AND\_BILE\_SALTS |
| -0.035657454 | -0.10491196 | REACTOME\_CYTOSOLIC\_SULFONATION\_OF\_SMALL\_MOLECULES |
| -0.09685158 | -0.19480094 | PHOSPHOLIPASE\_A2\_ACTIVITY |
| -0.18254085 | -0.008624122 | REACTOME\_STEROID\_HORMONES |
| -0.17024921 | -0.105185494 | XENOBIOTIC\_METABOLIC\_PROCESS |
| -0.024139855 | -0.16367477 | NCI\_SYNTHESIS\_OF\_BILE\_ACIDS\_AND\_BILE\_SALTS\_VIA\_24\_HYDROXYCHOLESTEROL |
| -0.038341194 | -0.15724574 | NCI\_SYNTHESIS\_OF\_BILE\_ACIDS\_AND\_BILE\_SALTS |
| -0.036712904 | -0.19669358 | REACTOME\_SYNTHESIS\_OF\_BILE\_ACIDS\_AND\_BILE\_SALTS\_VIA\_24\_HYDROXYCHOLESTEROL |
| -0.0041764076 | -0.024800204 | REACTOME\_HORMONE\_LIGAND\_BINDING\_RECEPTORS |
| -0.14939642 | -0.07668876 | DETECTION\_OF\_CHEMICAL\_STIMULUS |
| -0.0 | -0.19745262 | REACTOME\_PEPTIDE\_CHAIN\_ELONGATION |
| -0.024328705 | -0.09200811 | TRIACYLGLYCEROL\_METABOLIC\_PROCESS |
| -0.004912047 | -0.1211435 | KEGG\_SYSTEMIC\_LUPUS\_ERYTHEMATOSUS |
| -0.061281245 | -0.14412837 | SENSORY\_PERCEPTION\_OF\_TASTE |
| -0.01072094 | -0.0492003 | NCI\_HORMONE\_LIGAND\_BINDING\_RECEPTORS |
| -0.09871034 | -0.18785216 | KEGG\_AUTOIMMUNE\_THYROID\_DISEASE |
