# Supplementary material for: Computational approaches for discovery of common immunomodulators in fungal infections: towards broad-spectrum immunotherapeutic interventions
Source: BMC Microbiol. 2013 Oct 7;13:224. doi: 10.1186/1471-2180-13-224 (PMC3853472; doi:10.1186/1471-2180-13-224)
Supplement: Additional file 1 — Details of up- and down- regulated biclusters. [file 1471-2180-13-224-S1.zip › 2013-kidane-bmc/details-of-biclusters/dnreg-biclust-18.html]

**BICLUSTER\_ID** : DNREG-18  
**PATHOGENS** /2/ : c. albicans,a. fumigatus  
**KNOWN DRUG TARGETS** /0/ :   

| Gene Set | Leading Edge Genes |
| --- | --- |
| NCI PEPTIDE CHAIN ELONGATION |  |
| CORUM 28S RIBOSOMAL SUBUNIT MITOCHONDRIAL | MRPS34, MRPS12, MRPS16, MRPS11, MRPS33, MRPS28, MRPS14, MRPS15 |
| STRUCTURAL CONSTITUENT OF RIBOSOME |  |

| Color legend | | | | | | | | | | | |
| --- | --- | --- | --- | --- | --- | --- | --- | --- | --- | --- | --- |
| q-value | -1 | -0.2 | -0.05 | -0.01 | -0.001 | -0.0001 |
| Color |  |  |  |  |  |  |

TABLE OF Q-VALUES

| aspergillus fumigatus conidia a549 | aspergillus fumigatus monocytes | candida albicans moddc135 | Gene Set |
| --- | --- | --- | --- |
| -0.0 | -0.1741325 | -0.10015844 | NCI\_PEPTIDE\_CHAIN\_ELONGATION |
| -0.0034792167 | -0.1713698 | -0.0030206852 | CORUM\_28S\_RIBOSOMAL\_SUBUNIT\_MITOCHONDRIAL |
| -0.0 | -0.15857045 | -0.12606873 | STRUCTURAL\_CONSTITUENT\_OF\_RIBOSOME |
