# Supplementary material for: Computational approaches for discovery of common immunomodulators in fungal infections: towards broad-spectrum immunotherapeutic interventions
Source: BMC Microbiol. 2013 Oct 7;13:224. doi: 10.1186/1471-2180-13-224 (PMC3853472; doi:10.1186/1471-2180-13-224)
Supplement: Additional file 1 — Details of up- and down- regulated biclusters. [file 1471-2180-13-224-S1.zip › 2013-kidane-bmc/details-of-biclusters/dnreg-biclust-22.html]

**BICLUSTER\_ID** : DNREG-22  
**PATHOGENS** /1/ : a. fumigatus  
**KNOWN DRUG TARGETS** /8/ : EDNRA, CHRNA4, CHRM5, SCNN1G, CHRNA2, BDKRB2, CHRNB2, PLA2G6  

| Gene Set | Leading Edge Genes |
| --- | --- |
| LIGAND GATED CHANNEL ACTIVITY | CHRNA4, CHRND, CHRNB3, SCNN1G, CHRNA2, CNGB1, CHRNB2 |
| PHOSPHOLIPASE ACTIVITY | EDNRA, PLA1A, CHRM5, SMPD2, BDKRB2, PLA2G6 |
| DETECTION OF CHEMICAL STIMULUS | UGT2A1 |

| Color legend | | | | | | | | | | | |
| --- | --- | --- | --- | --- | --- | --- | --- | --- | --- | --- | --- |
| q-value | -1 | -0.2 | -0.05 | -0.01 | -0.001 | -0.0001 |
| Color |  |  |  |  |  |  |

TABLE OF Q-VALUES

| aspergillus fumigatus cluture filtrates a549 | aspergillus fumigatus 16hbe14o | Gene Set |
| --- | --- | --- |
| -0.11036019 | -0.003872479 | LIGAND\_GATED\_CHANNEL\_ACTIVITY |
| -0.022414522 | -0.19262274 | PHOSPHOLIPASE\_ACTIVITY |
| -0.07668876 | -0.1644083 | DETECTION\_OF\_CHEMICAL\_STIMULUS |
