# Supplementary material for: Computational approaches for discovery of common immunomodulators in fungal infections: towards broad-spectrum immunotherapeutic interventions
Source: BMC Microbiol. 2013 Oct 7;13:224. doi: 10.1186/1471-2180-13-224 (PMC3853472; doi:10.1186/1471-2180-13-224)
Supplement: Additional file 1 — Details of up- and down- regulated biclusters. [file 1471-2180-13-224-S1.zip › 2013-kidane-bmc/index.html]

  **Computational Approaches for Discovery of  
 Common Immunomodulators in Fungal Infections:**  
  Towards Broad-Spectrum Immunotherapeutic Interventions 
  Yared H. Kidane, Christopher Lawrence, and T. M. Murali

---

**Details of up- and down- regulated biclusters**

Table S1: Up-regulated biclusters click here

Table S2: Down-regulated biclusters click here
