# Supplementary material for: Computational approaches for discovery of common immunomodulators in fungal infections: towards broad-spectrum immunotherapeutic interventions
Source: BMC Microbiol. 2013 Oct 7;13:224. doi: 10.1186/1471-2180-13-224 (PMC3853472; doi:10.1186/1471-2180-13-224)
Supplement: Additional file 1 — Details of up- and down- regulated biclusters. [file 1471-2180-13-224-S1.zip › 2013-kidane-bmc/Additional_file_1:Table_S1.html]

**Summary of Up-regulated Biclusters and their Enrichment in drug targets** 

*Click on the "Bicluster ID" to view details. Statistically significant biclusters are highlighted in green*

| Bicluster ID | Num. Pathogens | Num. Pathogens | Num. Genesets | Bicluster Sig. (Pval) | List of Pathogens | Num. Lead Edg Genes | Num. Targets | Drg Target Enrichment (PVal) |
| --- | --- | --- | --- | --- | --- | --- | --- | --- |
| 2 | 2 | 2 | 174 | < 0.00001 | candida\_albicans\_moddc135 , aspergillus\_fumigatus\_dendritic | 493 | 111 | 0.00000 |
| 42 | 2 | 2 | 17 | 0.262865 | aspergillus\_fumigatus\_monocytes , candida\_albicans\_moddc135 | 73 | 27 | 0.00000 |
| 22 | 2 | 2 | 45 | 1.000000 | candida\_albicans\_huvec , aspergillus\_fumigatus\_cluture\_filtrates\_a549 | 111 | 28 | 0.00000 |
| 0 | 2 | 2 | 204 | < 0.00001 | aspergillus\_fumigatus\_conidia\_a549 , candida\_albicans\_moddc135 | 265 | 43 | 0.00006 |
| 30 | 2 | 2 | 33 | 1.000000 | alternaria\_alternata\_beas2b , aspergillus\_fumigatus\_cluture\_filtrates\_a549 | 82 | 17 | 0.00063 |
| 44 | 2 | 2 | 15 | 1.000000 | pneumocystis\_jirovecii\_macrophage , candida\_albicans\_neutrophils | 20 | 10 | 0.00000 |
| 6 | 2 | 2 | 129 | 1.000000 | candida\_albicans\_moddc135 , stachybotrys\_chartarum\_lung | 196 | 52 | 0.00000 |
| 50 | 2 | 2 | 8 | 1.000000 | aspergillus\_fumigatus\_monocytes , candida\_albicans\_neutrophils | 56 | 17 | 0.00000 |
| 28 | 2 | 2 | 34 | 1.000000 | aspergillus\_fumigatus\_conidia\_a549 , candida\_albicans\_neutrophils | 83 | 7 | 0.59715 |
| 40 | 2 | 2 | 17 | 0.262865 | candida\_albicans\_neutrophils , stachybotrys\_chartarum\_lung | 49 | 19 | 0.00000 |
| 20 | 2 | 2 | 55 | 1.000000 | candida\_albicans\_huvec , alternaria\_alternata\_beas2b | 137 | 24 | 0.00077 |
| 14 | 2 | 2 | 74 | 1.000000 | alternaria\_alternata\_beas2b , stachybotrys\_chartarum\_lung | 126 | 34 | 0.00000 |
| 8 | 2 | 2 | 110 | 1.000000 | candida\_albicans\_huvec , aspergillus\_fumigatus\_conidia\_a549 | 287 | 42 | 0.00066 |
| 4 | 2 | 2 | 145 | 1.000000 | candida\_albicans\_moddc135 , alternaria\_alternata\_beas2b | 220 | 46 | 0.00000 |
| 34 | 2 | 2 | 27 | 1.000000 | aspergillus\_fumigatus\_conidia\_a549 , pneumocystis\_jirovecii\_macrophage | 17 | 9 | 0.00000 |
| 24 | 2 | 2 | 41 | 1.000000 | pneumocystis\_jirovecii\_macrophage , stachybotrys\_chartarum\_lung | 59 | 31 | 0.00000 |
| 10 | 2 | 1 | 91 | 1.000000 | aspergillus\_fumigatus\_conidia\_a549 , aspergillus\_fumigatus\_cluture\_filtrates\_a549 | 213 | 39 | 0.00001 |
| 26 | 3 | 2 | 26 | 1.000000 | candida\_albicans\_moddc135 , candida\_albicans\_neutrophils , aspergillus\_fumigatus\_dendritic | 61 | 16 | 0.00005 |
| 16 | 3 | 2 | 42 | 1.000000 | candida\_albicans\_moddc135 , aspergillus\_fumigatus\_cluture\_filtrates\_a549 , aspergillus\_fumigatus\_dendritic | 92 | 22 | 0.00001 |
| 12 | 3 | 2 | 54 | 1.000000 | aspergillus\_fumigatus\_conidia\_a549 , alternaria\_alternata\_beas2b , aspergillus\_fumigatus\_dendritic | 86 | 17 | 0.00111 |
| 32 | 4 | 3 | 16 | 1.000000 | aspergillus\_fumigatus\_conidia\_a549 , candida\_albicans\_moddc135 , aspergillus\_fumigatus\_cluture\_filtrates\_a549 , stachybotrys\_chartarum\_lung | 15 | 3 | 0.13796 |
| 48 | 4 | 2 | 4 | 1.000000 | candida\_albicans\_huvec , aspergillus\_fumigatus\_monocytes , candida\_albicans\_moddc135 , aspergillus\_fumigatus\_cluture\_filtrates\_a549 | 3 | 1 | 0.24017 |
| 18 | 4 | 4 | 30 | 1.000000 | candida\_albicans\_moddc135 , alternaria\_alternata\_beas2b , pneumocystis\_jirovecii\_macrophage , aspergillus\_fumigatus\_dendritic | 19 | 11 | 0.00000 |
| 38 | 4 | 3 | 10 | 1.000000 | candida\_albicans\_moddc135 , pneumocystis\_jirovecii\_macrophage , aspergillus\_fumigatus\_cluture\_filtrates\_a549 , aspergillus\_fumigatus\_dendritic | 9 | 3 | 0.03758 |
| 52 | 4 | 3 | 4 | 1.000000 | candida\_albicans\_huvec , aspergillus\_fumigatus\_monocytes , pneumocystis\_jirovecii\_macrophage , aspergillus\_fumigatus\_dendritic | 3 | 1 | 0.24017 |
| 46 | 7 | 4 | 4 | 1.000000 | candida\_albicans\_huvec , aspergillus\_fumigatus\_conidia\_a549 , aspergillus\_fumigatus\_monocytes , candida\_albicans\_moddc135 , alternaria\_alternata\_beas2b , aspergillus\_fumigatus\_dendritic , stachybotrys\_chartarum\_lung | 3 | 1 | 0.24017 |
| 36 | 8 | 5 | 6 | 1.000000 | candida\_albicans\_huvec , aspergillus\_fumigatus\_conidia\_a549 , candida\_albicans\_moddc135 , alternaria\_alternata\_beas2b , pneumocystis\_jirovecii\_macrophage , candida\_albicans\_neutrophils , aspergillus\_fumigatus\_dendritic , stachybotrys\_chartarum\_lung | 2 | 0 | 1.00000 |
