# Supplementary material for: Computational approaches for discovery of common immunomodulators in fungal infections: towards broad-spectrum immunotherapeutic interventions
Source: BMC Microbiol. 2013 Oct 7;13:224. doi: 10.1186/1471-2180-13-224 (PMC3853472; doi:10.1186/1471-2180-13-224)
Supplement: Additional file 1 — Details of up- and down- regulated biclusters. [file 1471-2180-13-224-S1.zip › 2013-kidane-bmc/Additional_file_1:Table_S2.html]

**Summary of Down-regulated Biclusters and their Enrichment in drug targets** 

*Click on the "Bicluster ID" to view details. Statistically significant biclusters are highlighted in green*

| Bicluster ID | Num. Pathogens | Num. Pathogens | Num. Genesets | Bicluster Sig. (Pval) | List of Pathogens | Num. Lead Edg Genes | Num. Targets | Drg Target Enrichment (PVal) |
| --- | --- | --- | --- | --- | --- | --- | --- | --- |
| 2 | 2 | 1 | 34 | 1.000000 | aspergillus\_fumigatus\_conidia\_a549 , aspergillus\_fumigatus\_cluture\_filtrates\_a549 | 179 | 75 | 0.00000 |
| 12 | 2 | 2 | 5 | 0.583030 | candida\_albicans\_huvec , aspergillus\_fumigatus\_conidia\_a549 | 3 | 0 | 1.00000 |
| 22 | 2 | 1 | 3 | 1.000000 | aspergillus\_fumigatus\_cluture\_filtrates\_a549 , aspergillus\_fumigatus\_16hbe14o | 14 | 8 | 0.00001 |
| 8 | 2 | 1 | 6 | 1.000000 | aspergillus\_fumigatus\_conidia\_a549 , aspergillus\_fumigatus\_16hbe14o | 6 | 4 | 0.00076 |
| 4 | 2 | 2 | 17 | 0.262865 | candida\_albicans\_moddc135 , aspergillus\_fumigatus\_cluture\_filtrates\_a549 | 19 | 11 | 0.00000 |
| 0 | 2 | 2 | 133 | < 0.00001 | aspergillus\_fumigatus\_conidia\_a549 , candida\_albicans\_moddc135 | 104 | 21 | 0.00023 |
| 24 | 2 | 2 | 2 | 1.000000 | candida\_albicans\_moddc135 , aspergillus\_fumigatus\_dendritic | 0 | 0 | 1.00000 |
| 10 | 2 | 2 | 5 | 0.583030 | candida\_albicans\_huvec , aspergillus\_fumigatus\_cluture\_filtrates\_a549 | 4 | 0 | 1.00000 |
| 6 | 3 | 2 | 5 | 0.346411 | aspergillus\_fumigatus\_conidia\_a549 , candida\_albicans\_moddc135 , aspergillus\_fumigatus\_cluture\_filtrates\_a549 | 12 | 7 | 0.00002 |
| 14 | 3 | 2 | 3 | 0.623453 | aspergillus\_fumigatus\_conidia\_a549 , aspergillus\_fumigatus\_cluture\_filtrates\_a549 , stachybotrys\_chartarum\_lung | 5 | 4 | 0.00027 |
| 20 | 3 | 2 | 2 | 1.000000 | aspergillus\_fumigatus\_conidia\_a549 , candida\_albicans\_neutrophils , aspergillus\_fumigatus\_16hbe14o | 0 | 0 | 1.00000 |
| 18 | 3 | 2 | 3 | 0.623453 | aspergillus\_fumigatus\_conidia\_a549 , aspergillus\_fumigatus\_monocytes , candida\_albicans\_moddc135 | 8 | 0 | 1.00000 |
| 16 | 3 | 2 | 3 | 0.623453 | candida\_albicans\_huvec , aspergillus\_fumigatus\_conidia\_a549 , aspergillus\_fumigatus\_cluture\_filtrates\_a549 | 2 | 0 | 1.00000 |
